# Sleep-wake variation in body temperature regulates tau secretion and correlates with CSF and plasma tau

**DOI:** 10.1172/JCI182931

**Published:** 2025-02-04

**Authors:** Geoffrey Canet, Felipe Da Gama Monteiro, Emma Rocaboy, Sofia Diego-Diaz, Boutheyna Khelaifia, Kelly Godbout, Aymane Lachhab, Jessica Kim, Daphne I. Valencia, Audrey Yin, Hau-Tieng Wu, Jordan Howell, Emily Blank, Francis Laliberté, Nadia Fortin, Emmanuelle Boscher, Parissa Fereydouni-Forouzandeh, Stéphanie Champagne, Isabelle Guisle, Sébastien S. Hébert, Vincent Pernet, Haiyan Liu, William Lu, Ludovic Debure, David M. Rapoport, Indu Ayappa, Andrew W. Varga, Ankit Parekh, Ricardo S. Osorio, Steve Lacroix, Mark P. Burns, Brendan P. Lucey, Esther M. Blessing, Emmanuel Planel

**Affiliations:** 1 Centre de Recherche du CHU de Québec – Université Laval, Axe Neurosciences, Québec, Québec City, Canada.; 2 Université Laval, Faculté de Médecine, Département de Psychiatrie et Neurosciences, Québec, Québec City, Canada.; 3 Université Laval, Faculté de Médecine, Département de Médecine Moléculaire, Québec, Québec City, Canada.; 4 Department of Psychiatry, NYU Grossman School of Medicine, New York, New York, USA.; 5 Mount Sinai Integrative Sleep Center, Division of Pulmonary, Critical Care, and Sleep Medicine, Icahn School of Medicine at Mount Sinai, New York, New York, USA.; 6 Department of Neurology, Inselspital, and; 7 Center for Experimental Neurology (ZEN), Bern University Hospital, University of Bern, Bern, Switzerland.; 8 Department of Biomedical Research, University of Bern, Bern, Switzerland.; 9 Department of Neurology, Washington University School of Medicine, St. Louis, Missouri, USA.; 10 Laboratory for Brain Injury and Dementia, Department of Neuroscience, Georgetown University Medical Center, Washington, DC, USA.

**Keywords:** Cell biology, Neuroscience, Alzheimer disease, Proteoglycans

## Abstract

Sleep disturbance is bidirectionally associated with an increased risk of Alzheimer’s disease and other tauopathies. While the sleep-wake cycle regulates interstitial and cerebrospinal fluid (CSF) tau levels, the underlying mechanisms remain unknown. Understanding these mechanisms is crucial, given the evidence that tau pathology spreads through neuron-to-neuron transfer, involving the secretion and internalization of pathological tau forms. Here, we combined in vitro, in vivo, and clinical methods to reveal a pathway by which changes in body temperature (BT) over the sleep-wake cycle modulate extracellular tau levels. In mice, a higher BT during wakefulness and sleep deprivation increased CSF and plasma tau levels, while also upregulating unconventional protein secretion pathway I (UPS-I) events including (a) intracellular tau dephosphorylation, (b) caspase 3–mediated cleavage of tau (TauC3), and (c) membrane translocation of tau through binding to phosphatidylinositol 4,5-bisphosphate (PIP_2_) and syndecan 3. In humans, the increase in CSF and plasma tau levels observed after wakefulness correlated with BT increases during wakefulness. By demonstrating that sleep-wake variation in BT regulates extracellular tau levels, our findings highlight the importance of thermoregulation in linking sleep disturbances to tau-mediated neurodegeneration and the preventative potential of thermal interventions.

## Abstract 2

Wakefulness body temperature upregulates neuronal tau secretion and correlates with CSF and plasma tau levels, highlighting the importance of sleep and thermoregulation in Alzheimer’s disease.

## Graphical abstract

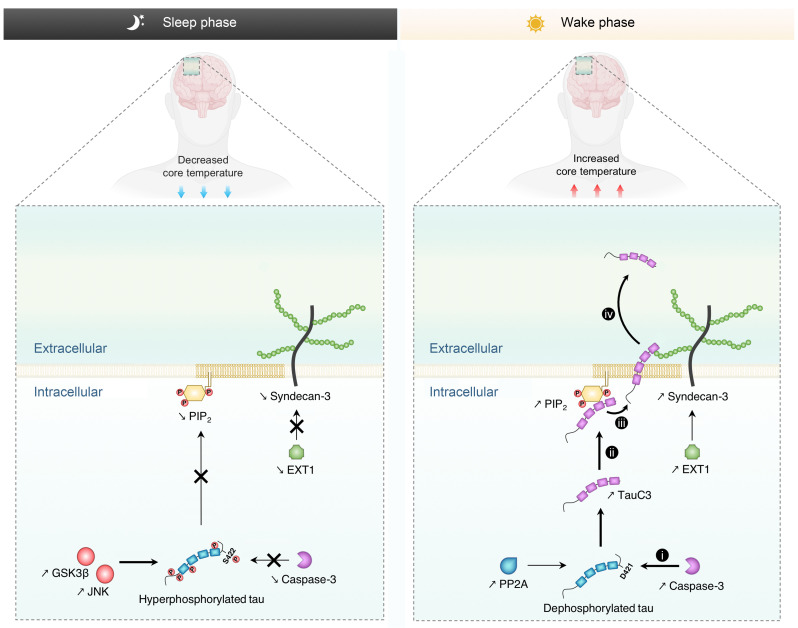

## Introduction

Neurodegenerative tauopathies, including Alzheimer’s disease (AD), are characterized by the intraneuronal accumulation and pathological neuron-to-neuron spread of hyperphosphorylated aggregated tau protein, along with elevated extracellular levels of soluble hyperphosphorylated tau, measurable in the cerebrospinal fluid (CSF) and plasma (1). Wakefulness is associated with increased CSF and interstitial fluid (ISF) tau levels, and sleep deprivation (SD) further increases these levels (2), while reducing CSF tau phosphorylation levels (3). Tau pathology may propagate through cell-to-cell transfer, which involves the secretion and internalization of pathological tau (4); yet the mechanisms by which the sleep-wake cycle modulates extracellular tau remain underexplored. Increased neuronal activity, characteristic of wakefulness, was shown to promote tau release (2, 5, 6), whereas sleep may enhance glymphatic system activity (7) and tau clearance (8). Increasing evidence suggests that age-related sleep and circadian rhythm disturbances exacerbate tau-mediated neurodegeneration (9), highlighting the importance of understanding how the sleep-wake cycle regulates processes that maintain tau proteostasis and limit tau propagation. Exploring these mechanisms could pave the way for clinical interventions to mitigate sleep and circadian disturbances in AD prevention.

Temperature, defined as the average speed of molecular kinetic energy, fundamentally affects chemical reactions rates, with biochemical processes being particularly sensitive to small temperature changes (10). Core body temperature (BT) variations during the sleep-wake cycle — up to 3°C in mice and 1.5°C in humans (11) — have been shown to influence tau processing. We previously demonstrated that sleep-wake variation in BT drives tau phosphorylation in mice (12), while SD prevented sleep-associated tau phosphorylation by disrupting the normal BT decrease during sleep (12). In older adults, we found that core BT during wakefulness was inversely associated with tau phosphorylation in CSF and plasma, as well as tau tracer uptake in early Braak stage areas (13). This suggests that age-related thermoregulatory abnormalities may contribute to tau hyperphosphorylation and early tau pathology.

In this study, we sought to investigate the role of core BT variation in modulating tau processing. Specifically, we tested whether sleep-wake variation in BT influences extracellular total tau levels in mice and in vitro. Additionally, we explored the molecular pathways underlying these temperature-dependent actions, focusing on tau secretion pathways. As a leaderless protein, tau is mainly secreted through the unconventional protein secretion pathway I (UPS-I), consistent with approximately 90% of extracellular tau being unbound to vesicular organelles (4, 14–18). Consequently, we investigated whether tau secretion is mediated by a temperature-dependent modulation of the UPS-I pathway. In humans, we examined whether plasma tau varies over the diurnal interval, as we previously showed for CSF, and tested whether BT variation predicts plasma and CSF changes. We hypothesized that increases in plasma and CSF tau levels following the day or the active waking interval would correlate with a BT increase during the same interval.

Here, we demonstrate in mice and in vitro that core BT variation across the sleep-wake cycle drove changes in extracellular tau levels. We show that higher BT — whether during wakefulness, SD, or induced by mild hyperthermia — promoted tau secretion into the CSF via the upregulation of a specific UPS-I–related pathway. We elucidated a temperature-dependent intracellular pathway involving (a) caspase 3–mediated TauC3 production, (b) TauC3 binding to phosphatidylinositol 4,5-bisphosphate (PIP_2_) at the plasma membrane, and (c) the transmembrane export of TauC3 facilitated by the HSPG family member syndecan 3 (SDC3). We found that higher BT during waking promoted the secretion of truncated and dephosphorylated tau species, which are less prone to aggregation. In humans, we found that plasma total tau levels were higher after wakefulness compared with after sleep, similar to previous findings for CSF (2). Furthermore, the increase in both CSF and plasma tau levels correlated positively with BT increases, supporting our in vivo and in vitro findings.

## Results

### Sleep-wake–like temperatures regulate tau extracellular levels.

**Figure 1:**
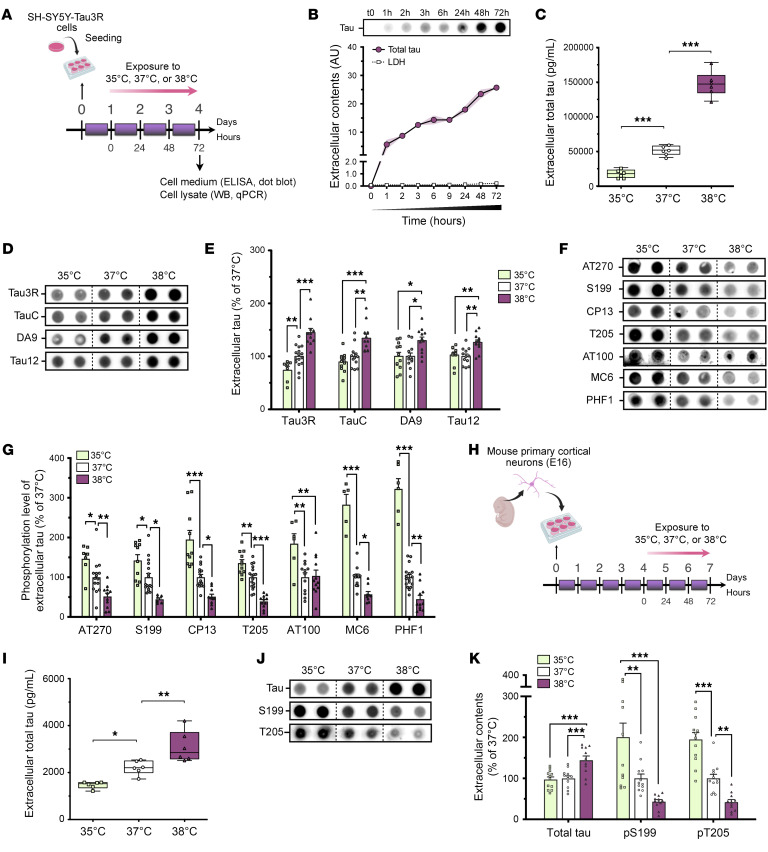
Tau secretion is temperature dependent in neuronal cells. (**A**) Twenty-four hours after seeding, SH-Tau3R cells were exposed to 35°C, 37°C, 38°C, or 39°C for 72 hours. (**B**) Extracellular accumulation of tau protein (Tau3R) (*n* = 6–12; mean ± SEM; error envelopes are shown in light purple) and LDH (*n* = 4–9; mean ± SEM) over time in cell medium of neurons cultured at 37°C. (**C**) The increase in extracellular tau levels (pg/mL) was temperature dependent in SH-Tau3R cells exposed to 35°C, 37°C, or 38°C for 72 hours (*n* = 6; Tukey’s test; box and whiskers show minimum to maximum and median). (**D** and **E**) The increase in extracellular tau levels was temperature dependent (Tau3R, TauC, DA9, and Tau12 antibodies) in SH-Tau3R cells exposed to 35°C, 37°C, or 38°C (*n* = 7–16; Tukey’s test; mean ± SEM). (**F** and **G**) The phosphorylation level of extracellular tau at AT270 (T181), S199, CP13 (S202), T205, AT100 (S212/S214), MC6 (S235), and PHF1 (S396/S404) was decreased at 38°C compared with 35°C or 37°C (*n* = 7–16; Tukey’s test; mean ± SEM. (**H**) Four days after seeding, mouse primary cortical neurons were exposed at 35°C, 37°C, or 38°C for 72 hours. (**I**) The increase in extracellular tau levels was temperature dependent in mouse primary neurons exposed to 35°C, 37°C, or 38°C (*n* = 6; Dunnett’s test; box and whiskers show minimum to maximum and median). (**J** and **K**) The increase in extracellular tau levels was temperature dependent (Tau3R antibody), whereas its phosphorylation level at S199 and T205 was decreased at 38°C compared with 35°C or 37°C (*n* = 11–12; Tukey’s test; mean ± SEM). (**K**) Data are from a minimum of 2 independent experiments. *P < 0.05, **P < 0.01, and ***P < 0.001.

We first tested whether temperatures mimicking BT variations during the sleep-wake cycle regulate neuronal tau secretion in human SH-SY5Y cells stably expressing human tau 3-repeat isoform (SH-Tau3R) (Figure 1A) and in primary mouse cortical cells (Figure 1G). We found that extracellular tau levels plateaued after 72 hours at 37°C (Figure 1B). Using ELISA and dot blotting, we observed an approximately 2-fold increase in tau secretion at 38°C compared with 35°C in both human (Figure 1, C–E, and Supplemental Figure 1A; supplemental material available online with this article; https://doi.org/10.1172/JCI182931DS1) and mouse neuron-like cells (Figure 1, G–J), without cytotoxicity except at 39°C (Supplemental Figure 1, C and D). These findings extend to 3R and 4R tau isoforms, as similar results were previously reported in HeLa cells expressing Tau4R (18). In order to more faithfully replicate physiological BT variations occurring during a full 24-hour sleep-wake cycle, we confirmed that shorter exposures (6, 24, and 48 hours) corroborated these findings (Supplemental Figure 1E). Our prior research demonstrated that elevated core BT during wakefulness or sauna-like conditions leads to tau dephosphorylation (12, 19). We extended these findings by demonstrating that higher temperatures reduce tau phosphorylation levels both intracellularly (Supplemental Figure 1, F and G) and extracellularly (Figure 1, F–K, and Supplemental Figure 1B) in SH-Tau3R and mouse primary cells. Overall, exposure to higher temperatures similar to those experienced during wakefulness promoted tau secretion, with the secreted tau species being dephosphorylated.

### Tau secretion pathways are particularly sensitive to temperature variations.

To assess whether temperature effects are specific to tau secretion, we compared extracellular contents of various proteins, including microtubule-associated protein 2 (MAP2), α-synuclein, fibroblast growth factor 2 (FGF2), caspase-1, and neurofilament light chain (NfL). MAP2 is another microtubule-associated protein, and NfL is a cytoskeletal protein often used as a negative control (NC) for extracellular tau (2, 20). FGF2, α-synuclein, and caspase-1 are known to be secreted via the UPS pathways (21, 22). However, temperature did not affect the secretion profiles or the intracellular expression of these proteins (Supplemental Figure 1, H–J).

Next, we conducted proteomics analyses of the extracellular medium of SH-Tau3R cells to assess the effect of temperature on the secretome. Out of 2,099 identified proteins, 1,536–1,620 were quantified (Supplemental Figure 2, A–C). A comparison of the secretome profiles at 38°C versus 35°C revealed significant changes, with 240 proteins downregulated and 222 upregulated, including tau (Supplemental Figure 2C). Notably, extracellular α-synuclein levels also showed temperature dependence (Supplemental Figure 2, B and C), consistent with previous reports (2), but contrasting with our dot blot results. To identify proteins relevant to tau secretion, we filtered for “extracellular” and “secretory pathway” proteins using the ComPPI database. Among the 29 detected proteins, several were upregulated at 38°C (Supplemental Figure 2, D–F). These results show that tau secretion pathways were particularly sensitive to temperature variation within the physiological range, among a minority of other signaling pathways.

### Wakefulness temperatures promote tau release through its caspase 3–mediated cleavage.

**Figure 2:**
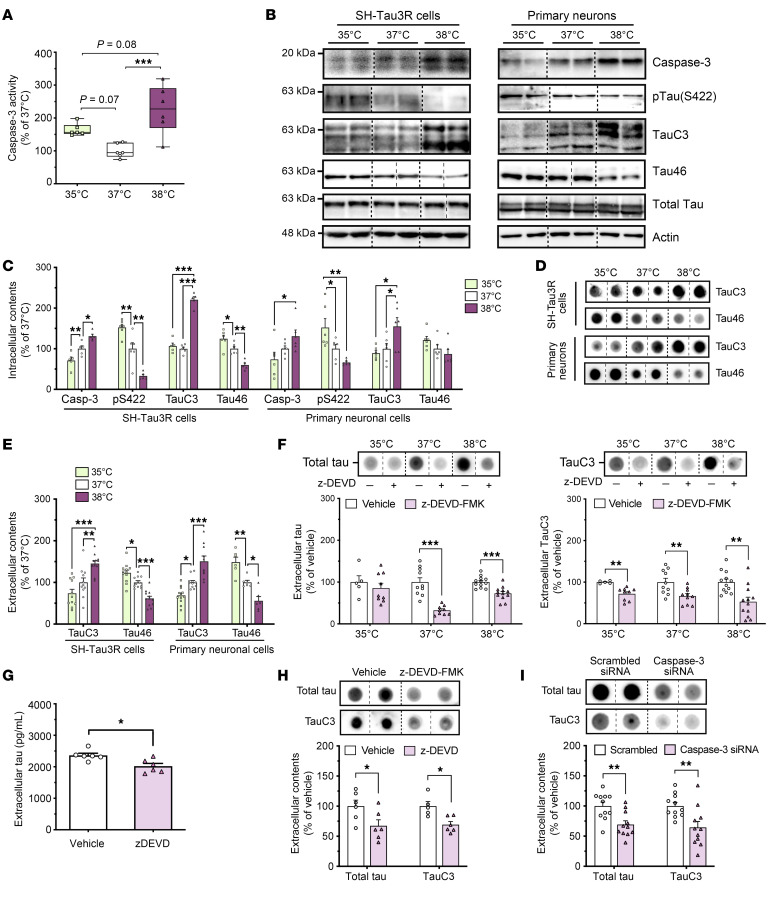
Wakefulness temperatures promote tau release through its caspase 3–mediated cleavage in neuronal cells. (**A**) The increase in the proteolytic activity of caspase 3 was temperature dependent in SH-Tau3R cells (*n* = 6; Tukey’s; box and whiskers show minimum to maximum and median). (**B** and **C**) The intracellular expression levels of caspase 3 (Casp-3), p-Tau (S422), TauC3, and Tau46 were temperature dependent in SH-Tau3R cells (left panels) or primary neurons (right panels) (*n* = 5–6; Tukey’s test; mean ± SEM). (**D** and **E**) Extracellular levels of TauC3 and Tau46 were oppositely temperature dependent in SH-Tau3R cells (left panels) or primary neurons (right panels) (*n* = 6–12; Tukey’s test; mean ± SEM). (**F**) The inhibition of caspase 3 with z-DEVD-FMK (20 μM) decreased total tau (Tau3R) (*n* = 5–12; unpaired, 2-tailed *t* test) and TauC3 (*n* = 3–8; Mann-Whitney *U* test) extracellular levels in SH-Tau3R cells exposed to 35°C, 37°C, or 38°C (mean ± SEM). The inhibition of caspase 3 with z-DEVD-FMK (20 μM) decreased the extracellular levels of (**G**) total tau (ELISA; *n* = 6; unpaired, 2-tailed *t* test) and (**H**) Tau3R and TauC3 (dot blots, *n* = 5–6; unpaired, 2-tailed *t* test; mean ± SEM. (**I**) The genetic knockdown of caspase 3 decreased total tau (Tau3R) and TauC3 extracellular levels in SH-Tau3R cells exposed to 37°C (*n* = 11; unpaired, 2-tailed *t* test; mean ± SEM). Data are from a minimum of 2 independent experiments. *P < 0.05, **P < 0.01, and ***P < 0.001.

We further sought to identify cellular pathways underlying temperature-dependent effects upon extracellular tau levels. We focused on caspase 3–mediated tau cleavage, as extracellular tau predominantly exists as the TauC3 proteolytic fragment in AD (3, 18, 23). Our findings revealed that wakefulness temperatures (38°C) increased caspase 3 activity and protein levels in both human and mouse cells compared with 35°C or 37°C (Figure 2, A–C), consistent with previous reports indicating that protease and caspase activities are temperature dependent (24, 25). Interestingly, this coincided with the intracellular dephosphorylation of tau at S422 (Figure 2, B and C), known to facilitate the caspase 3–mediated cleavage at D421 (26, 27). Consequently, we observed increased intra- and extracellular levels of TauC3 at 38°C (Figure 2, B–E), along with decreased levels of Tau46 (epitope 428–441) (Figure 2, B–E), which does not recognize C-terminal cleaved tau (23). To confirm the role of caspase 3 in tau secretion, we observed that its inhibition — either with the z-DEVD-FMK pharmacological inhibitor or caspase 3 mRNA targeting with a siRNA — significantly decreased extracellular tau release (Figure 2, F–I). Our data collectively demonstrate that wakefulness temperatures promoted caspase 3–mediated tau cleavage, driving its UPS-I–mediated secretion.

### Wakefulness temperatures drive TauC3 secretion through SDC3 upregulation.

**Figure 3:**
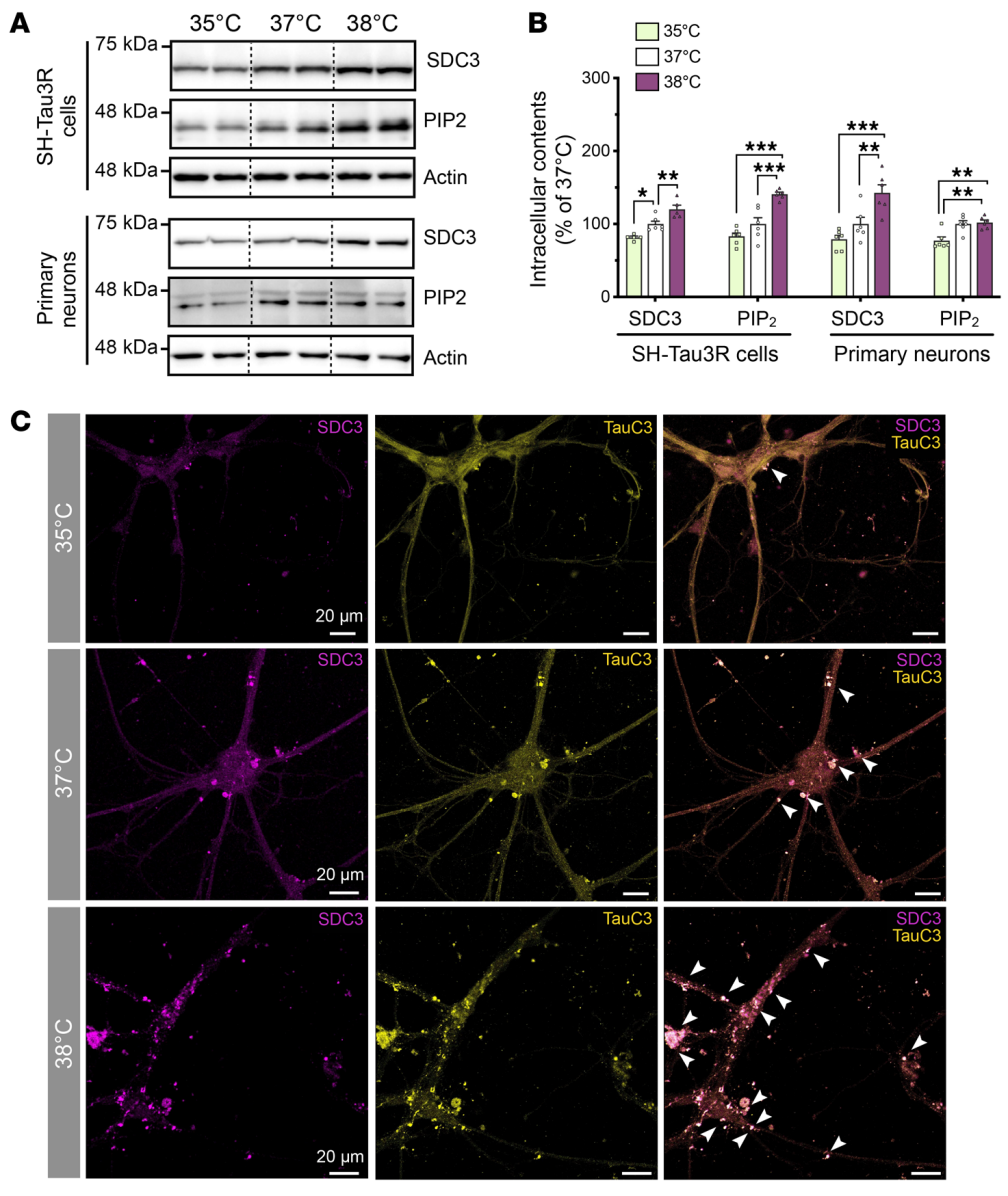
Wakefulness temperatures promote TauC3 interaction with SDC3 in neuronal cells. (**A** and **B**) Intracellular expression levels of SDC3 and PIP_2_ were temperature dependent in SH-Tau3R cells and in primary neurons (*n* = 6; Tukey’s test; mean ± SEM). Data are from a minimum of 2 independent experiments. *P < 0.05, **P < 0.01, and ***P < 0.001 versus the respective control condition. (**C**) Merged staining images show a temperature-dependent increase of colocalization between SDC3 (purple) and TauC3 (yellow) in primary mouse cortical neurons (marked with white arrowheads). Scale bars: 20 μm).

Heparan sulfate proteoglycans (HSPGs) are essential components of the UPS-I pathway, facilitating the direct export of intracellular proteins across the plasma membrane (28). Ubiquitously expressed on cell surfaces, HSPGs consist of a core proteoglycan with heparan sulfate chains, whose elongation is driven by the glycosyltransferase exostosin 1 (EXT1) in the brain (29). Among the family of HSPGs, neuronal SDC3 is particularly relevant to AD pathophysiology (30, 31). We hypothesized that wakefulness temperatures might promote the secretion of TauC3 by enhancing its interaction with SDC3. First, we found that wakefulness temperatures increased SDC3 and EXT1 protein and mRNA expression in SH-Tau3R cells and primary neuronal cells (Figure 3, A and B, and Supplemental Figure 3). Second, using confocal microscopy, we observed a temperature-dependent increase in the merged staining of SDC3 and TauC3, with numerous puncta (SDC3^+^ and TauC3^+^) mainly localized in the soma and in proximal neurites of primary neurons cultured at 38°C (Figure 3C and Supplemental Figure 4).

**Figure 4:**
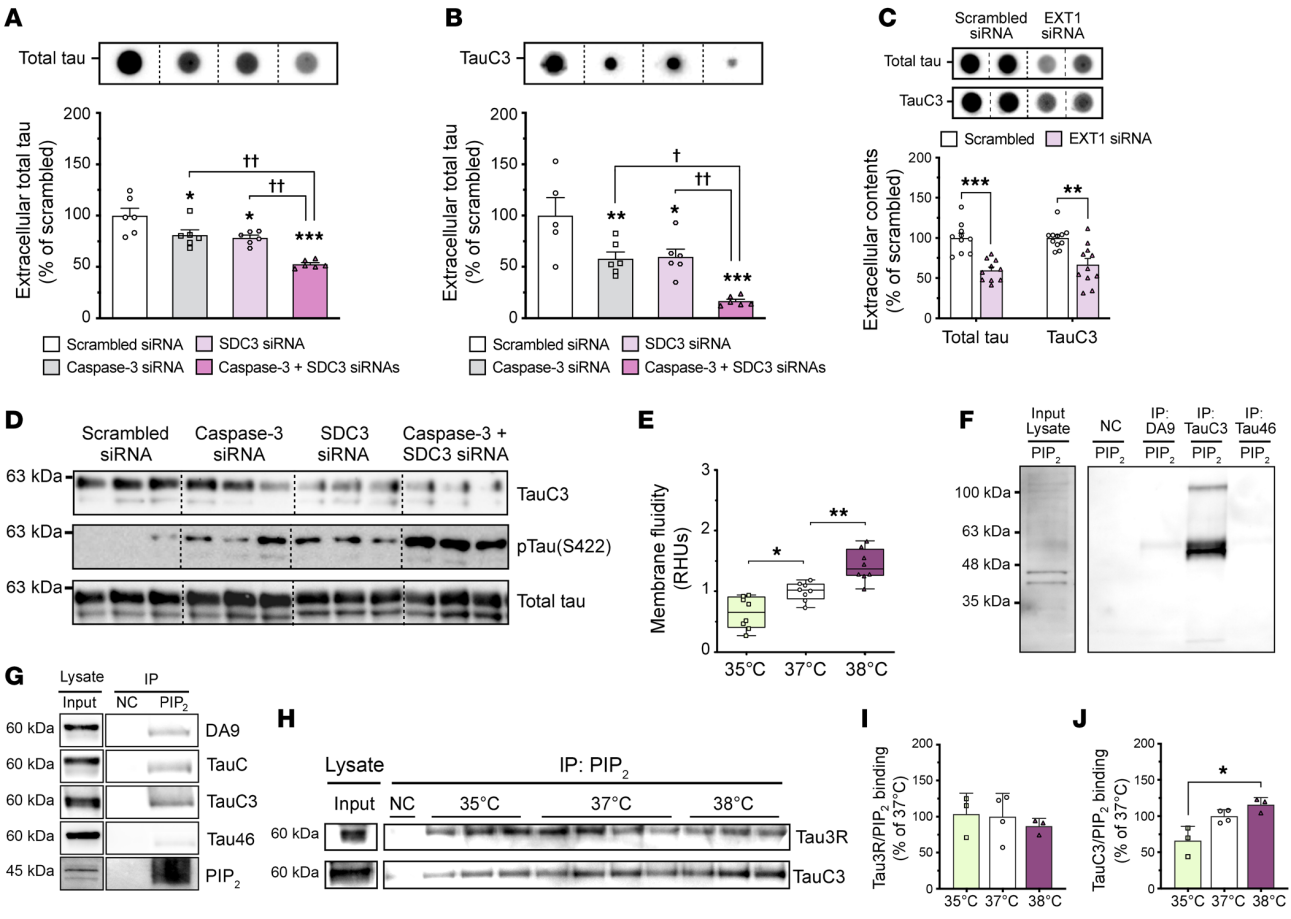
Wakefulness temperatures promote TauC3 secretion though its interaction with PIP_2_ and SDC3. (**A** and **B**) SDC3 knockdown decreased total tau (Tau3R) and TauC3 extracellular levels in SH-Tau3R cells exposed to 37°C, and the cotransfection of caspase 3 siRNA and SDC3 siRNA induced additive effects in the suppression of tau secretion (*n* = 5–6; Tukey’s test; mean ± SEM). ^†^*P* < 0.05 and ^††^*P* < 0.01 versus the indicated condition. (**C**) EXT1 knockdown decreased total tau (Tau3R) and TauC3 extracellular levels in SH-Tau3R cells exposed to 37°C (*n* = 11; unpaired, 2-tailed *t* test; mean ± SEM). (**D**) Knockdown of caspase 3 and SDC3 decreased the intracellular expression of TauC, while increasing tau phosphorylation at S422 (representative Western blot; *n* = 3). (**E**) The increase in membrane fluidity was temperature dependent in SH-Tau3R cells (*n* = 8; Tukey’s test; box and whiskers show minimum to maximum and median). (**F** and **G**) PIP_2_ showed a better binding affinity for TauC3 than for full-length tau (anti-DA9, anti-Tau46, and anti-TauC antibodies) in SH-Tau3R cells exposed to 37°C. (**H**–**J**) The increase in binding between PIP_2_ and TauC3 was temperature dependent (*n* = 3; Kruskal-Wallis test; mean ± SEM). Data are from a minimum of 2 independent experiments. *P < 0.05, **P < 0.01, and ***P < 0.001 versus the respective control condition.

To confirm the role of SDC3 and EXT1 in tau secretion via the UPS-I pathway, we conducted siRNA-mediated knockdown experiments targeting these proteins. Knockdown of either SDC3 or EXT1 led to a significant reduction in extracellular levels of total tau and TauC3 compared with cells transfected with scrambled siRNA (Figure 4, A–C). To further examine the interplay between caspase 3 and SDC3 in tau secretion, we performed simultaneous knockdown of both proteins, which resulted in almost complete suppression of intra- and extracellular TauC3 levels (Figure 4, A and B). Intriguingly, the inhibition of TauC3 expression was associated with an increase in tau hyperphosphorylation at S422 (Figure 4D). These observations collectively emphasize a complementary role of caspase 3 and SDC3 in mediating the extracellular export of TauC3 during wakefulness.

### Wakefulness temperatures facilitate tau recruitment and release at the plasma membrane.

Plasma membranes, given their lipidic composition, are particularly sensitive to temperature changes. Elevated temperatures enhance membrane fluidity and permeability in plant and animal cells (32, 33). On this basis, we hypothesized that wakefulness-like temperatures might drive tau secretion by optimizing membrane properties to facilitate its extracellular translocation. We found that SH-Tau3R cells exhibited increased membrane fluidity at wakefulness temperatures (Figure 4E). Previous studies have described that tau can bind to PIP_2_ at the inner plasma membrane layer (16). Therefore, we investigated whether temperature affects the interaction of tau with PIP_2_, potentially initiating its translocation across the membrane. We found that PIP_2_ expression was temperature dependent in both human and mouse neuron–like cells (Figure 3, A and B). Notably, similar temperature-dependent PIP_2_ increases have been previously reported in yeast and plant cells (34, 35), suggesting a highly conserved process.

To explore whether full-length tau (DA9) and TauC3 have the same affinity for PIP_2_, we performed co-IP using anti-DA9, anti-TauC3, or anti-Tau46 antibodies and probed for PIP_2_ by Western blotting (Figure 4F), or by co-IP using an anti-PIP_2_ antibody to assess the interaction with different tau antibodies (Figure 4G). Both approaches revealed that PIP_2_ preferentially binds TauC3 rather than full-length tau (Tau46 signal was barely detectable) (Figure 4, F and G). Finally, we demonstrated that an increase in temperature significantly promoted the binding of TauC3 to PIP_2_, while the binding of full-length tau to PIP_2_ tended to decrease (Figure 4, H–J). Altogether, these findings suggest that wakefulness temperatures promoted the UPS-I–mediated secretion of TauC3 by facilitating its interaction with PIP_2_ and SDC3 at the plasma membrane, thereby initiating vesicle-free tau release.

### Wakefulness and SD upregulate the UPS-I pathway by increasing core BT in mice.

**Figure 5:**
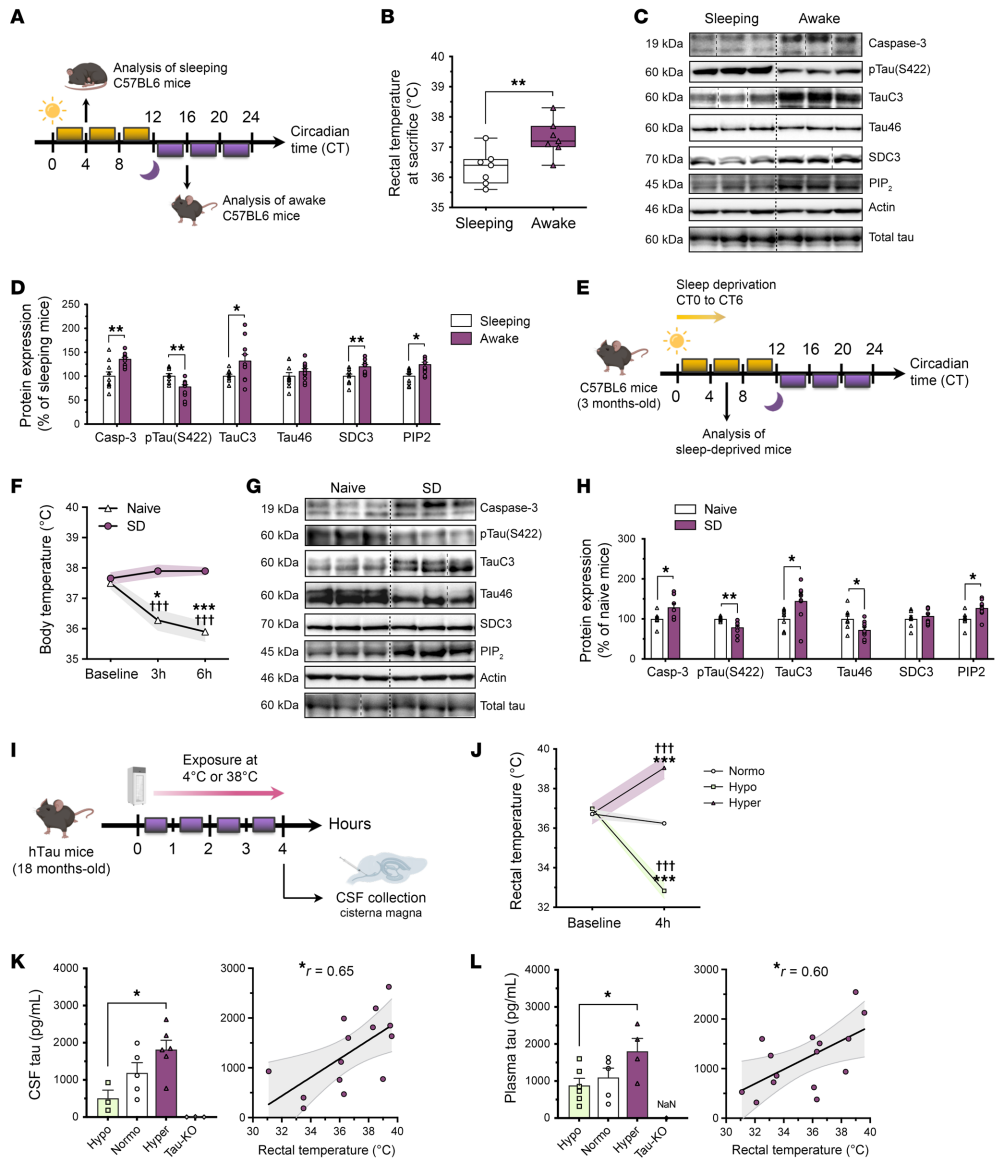
Wakefulness, SD, and mild hyperthermia promote UPS-I–dependent tau release in CSF in mice. (**A**) C57BL6 mice were euthanized during sleep (CT4, 10:00 am) or the active period (CT16, 8:45 pm). (**B**) Awake mice had higher rectal temperatures (°C) compared with sleeping mice (*n* = 7; unpaired, 2-tailed *t* test; box and whiskers with minimum to maximum and median). (**C** and **D**) Caspase 3, TauC3, SDC3, and PIP_2_ expression levels were increased in the cortices of awake mice compared with levels in sleeping mice, whereas p-Tau (S422) levels were decreased (*n* = 10; unpaired, 2-tailed *t* test; mean ± SEM). (**E**) C57BL6 mice were sleep deprived (*n* = 9) for 6 hours and then compared with naive mice (*n* = 7). (**F**) SD inhibited the drop in BT (°C) induced by sleep (*n* = 5; Tukey’s; mean ± SEM shown as error envelopes). (**G** and **H**) Caspase 3, TauC3, and PIP_2_ expression levels were increased in the cortices of sleep-deprived mice compared with naive mice, whereas p-Tau (S422) and Tau46 levels were decreased (unpaired, 2-tailed *t* test; mean ± SEM). (**I** and **J**) hTau mice were exposed for 4 hours to 4°C (hypo, *n* = 6) or 38°C (hyper, *n* = 6) and then compared with naive (normo) mice (normo, *n* = 5; Šidák’s test; mean ± SEM shown as error envelopes). ***P < 0.001 versus the naive (normo) group at baseline; ^†††^*P* < 0.001 versus the respective group at baseline. (**K**) Hyperthermic (hyper) mice had higher CSF tau levels than did hypothermic (hypo) mice (*n* = 5 normo; *n* = 3 hypo; *n* = 6 hyper; Kruskal-Wallis test; mean ± SEM). CSF tau levels were significantly correlated with rectal BT (°C) (Pearson’s test; error envelopes are shown in gray). (**L**) Hyperthermic mice had higher plasma tau levels than did hypothermic mice (*n* = 5 normo; *n* = 6 hypo; *n* = 5 hyper; Dunnett’s test; mean ± SEM). Plasma tau levels were significantly correlated with rectal BT (°C) (Pearson’s test; error envelopes are shown in gray). Data are from a minimum of 2 independent experiments. NaN, not a number. *P < 0.05, **P < 0.01, and ***P < 0.001.

To assess whether higher CSF and ISF tau levels during wakefulness and following SD (2) are related to naturally elevated BT (Figure 5, A and B) (12), we analyzed UPS-I–related protein expression in the cortex of WT mice across sleep versus wakefulness or following SD. Our findings revealed that the expression levels of caspase 3, TauC3, SDC3, and PIP_2_ were upregulated (Figure 5, C and D), along with tau dephosphorylation at S422 (Figure 5, C and D), in awake mice compared with sleeping mice. Moreover, the rectal temperature of mice at euthanasia was significantly correlated with the expression levels of caspase 3, phosphorylated tau (p-tau) at S422, SDC3, and PIP_2_ (Supplemental Figure 5, A–F). We further showed that 6 hours of SD (Figure 5E) prevented the natural decrease in core BT during sleep (Figure 5F) and triggered the upregulation of caspase 3, TauC3, and PIP_2_ levels associated with decreased S422 phosphorylation and Tau46 expression (Figure 5, G and H). These findings align with previous reports indicating that SD promotes tau dephosphorylation (12, 36). Altogether, our results suggest that conditions related to physiological elevation in BT promoted the expression of the UPS-I–related components involved in tau secretion.

### Mild hyperthermia increases CSF and plasma tau levels in hTau mice.

To determine whether induced changes in BT affect CSF and plasma tau levels, we subjected human tau (hTau)-expressing mice to hypo- or hyperthermic conditions for 4 hours and compared them with normothermic mice (Figure 5, I and J). After having confirmed that core BT and brain temperature closely mirror each other without significant differences (Supplemental Figure 5, A and B), we observed that hyperthermic mice had a comparable increase in both CSF and plasma tau concentrations (Figure 5, K and L), with concentrations significantly correlating with rectal temperatures measured immediately after exposure (Figure 5, K and L). The rise in circulating tau concentrations was associated with increased cortical expression of caspase 3, TauC3, SDC3, and PIP_2_, along with a reduction in tau phosphorylation at S422 and Tau46 expression (Supplemental Figure 6, C and D), all correlating with rectal temperature (Supplemental Figure 6, E–J). These findings collectively suggest that core BT variation influenced CSF and plasma tau levels through upregulation of the UPS-I pathway and emphasize the pivotal role of sleep-wake BT variation in regulating tau secretion and propagation.

### BT correlates with CSF and plasma tau but not CSF NfL in humans.

**Figure 6:**
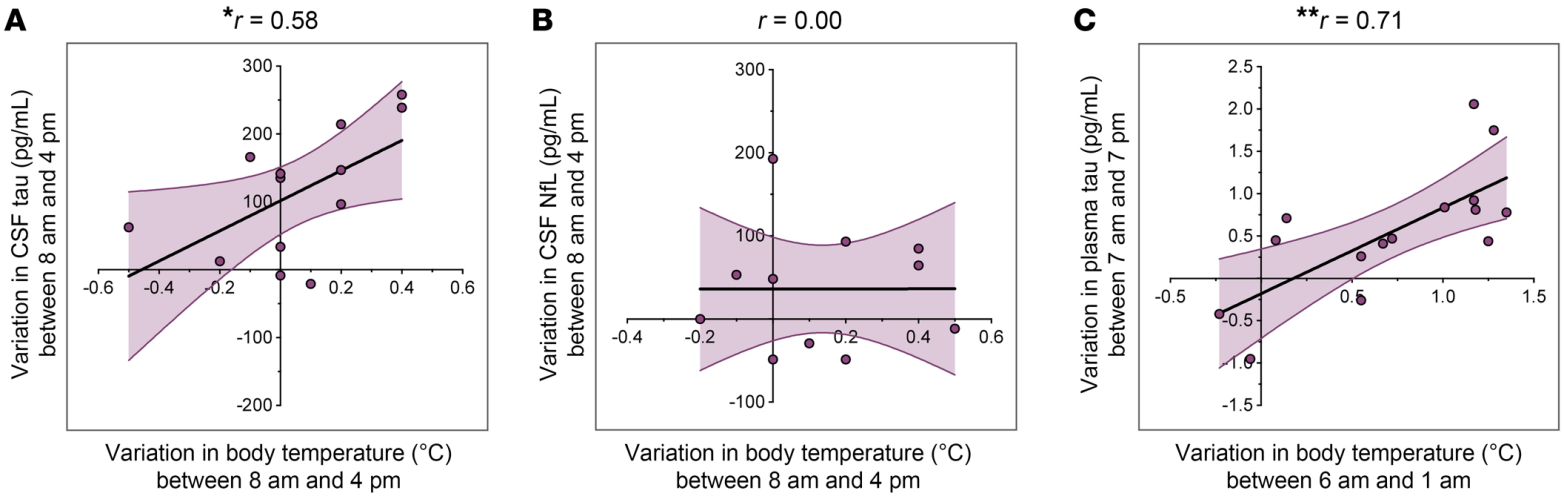
BT correlates with CSF and plasma tau levels in humans. (**A**) The variation in total CSF tau concentrations between 8 am and 4 pm was significantly correlated to the variation in oral temperature at the same times (*n* = 13; Pearson’s test), (**B**) but no correlation was observed for CSF NfL concentrations or oral temperature (*n* = 11; Pearson’s test). (**C**) The variation in total plasma tau concentrations between 7 am and 7 pm was significantly correlated to the variation in core BT between 6 pm and 1 am (*n* = 15; Pearson’s test). Standard error bars are shown as error envelopes in light purple. *P < 0.05 and **P < 0.01.

To test the relationship between BT and sleep-wake tau dynamics in humans, we analyzed 2 datasets from older adults in whom BT and tau levels (CSF or plasma) were measured simultaneously at multiple time points. We examined the correlation between the magnitude of change in tau levels after wakefulness (ΔTau) and the concurrent rise in BT during wakefulness (ΔBT). Predefined measurement times were selected within the constraints of the available datasets (see Methods). We found that plasma tau levels were, on average, approximately 15% higher in the evening than in the morning (Supplemental Tables 2 and 3), similar to previous findings in CSF (2, 3). This study is, to our knowledge, the first to compare diurnal (morning vs. evening) plasma tau levels in older adults. We observed a positive and significant correlation between ΔBT and ΔTau for both CSF and plasma tau, with no correlation for CSF NfL levels (Figure 6, A–C), and a consistent relationship across CSF and plasma data sets. Participants exhibiting a large positive ΔTau, i.e., higher afternoon-evening levels compared with morning, also showed a large positive ΔBT. Conversely, participants with negligible or negative ΔTau showed minimal or negative ΔBT (Figure 6, A–C).

The observed ΔBT values for the plasma data set agree with our previous study and, to our knowledge, represent the first report of correlations between BT and circulating tau in humans. Notably, our overall finding of approximately 15% higher plasma tau levels in the evening comprised a broad range of ΔTau values that were explained by ΔBT. Furthermore, this strong correlation was observed for the selected time interval of 6 pm to 1 am, as well as other adjacent time intervals surrounding the evening BT drop. Specifically, we observed the strongest correlation for the 6 pm to 12 am interval, with significant correlations also observed across 17 adjacent ΔBT intervals (5–9 pm to 9 pm–4 am) (Supplemental Figures 7 and 8). Overall, these results supported our in vivo and in vitro findings, in which higher BT during wakefulness drove increased tau secretion.

## Discussion

**Figure 7:**
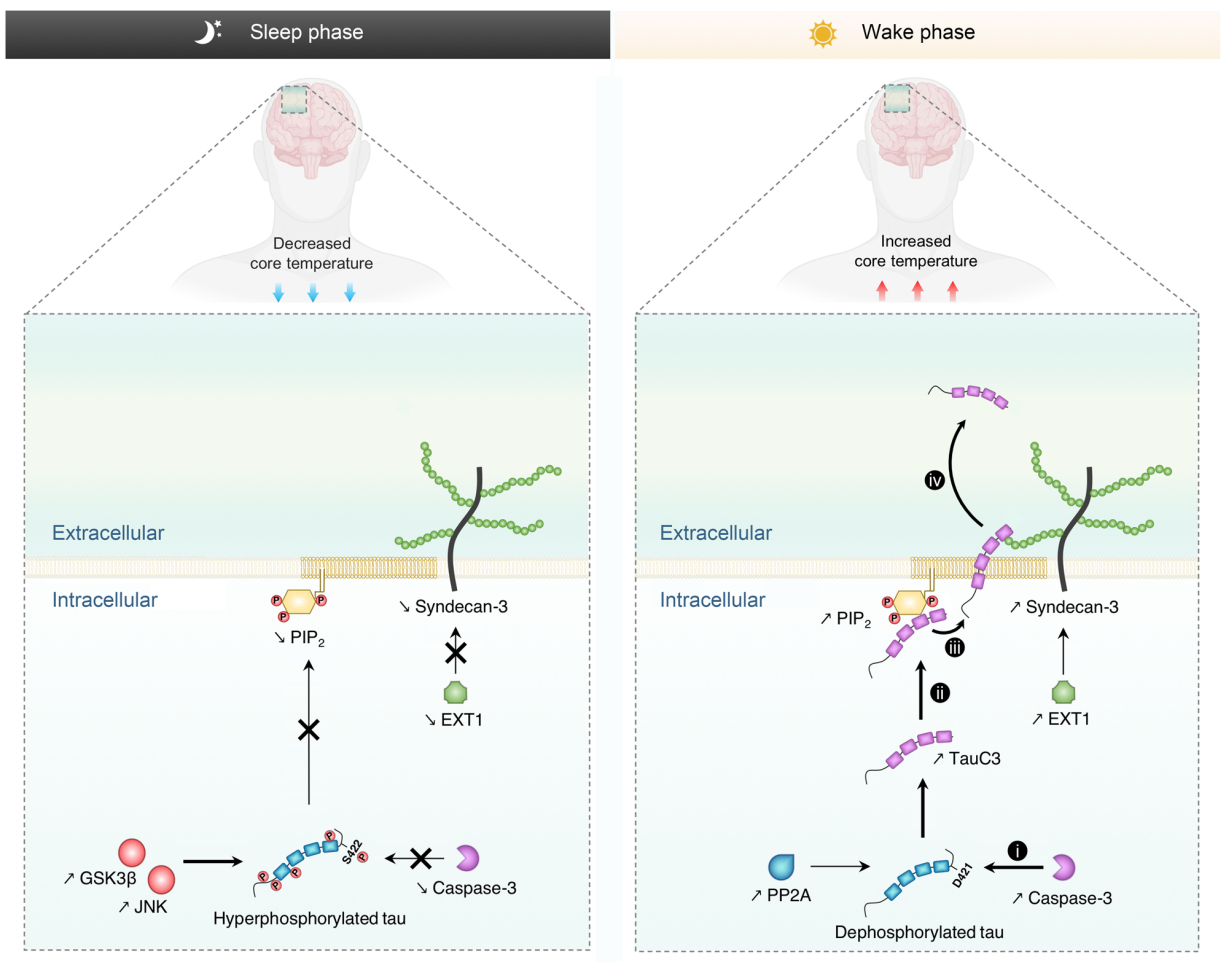
Proposed mechanism to elucidate the regulatory effect of BT on tau secretion via the UPS-I pathway during the sleep-wake cycle. During wakefulness, the physiological elevation in BT instigates a series of events triggering tau secretion. (i) There is an increase in caspase 3 activity, concomitant with tau dephosphorylation, especially at S422, leading to augmented cleavage of tau at D421 and yielding the TauC3 fragment. (ii) Subsequently, TauC3 is sequestered at the inner leaflet of the plasma membrane due to its strong affinity binding for PIP_2_. (iii and iv) The interplay between TauC3 and SDC3 initiates and facilitates the export process across the plasma membrane, which has heightened fluidity and permeability properties during wakefulness. In contrast, during sleep, the decrease in BT inhibits caspase 3 activity and promotes tau hyperphosphorylation at S422, preventing the generation of TauC3. The sleep phase also leads to reduced expression levels of both PIP_2_ and SDC3, along with lower membrane fluidity, resulting in diminished extracellular tau levels. GSK3β, glycogen synthase kinase 3β; JNK, c-Jun N-terminal kinase.

This study examined how BT variation during the sleep-wake cycle affect extracellular tau levels. We reveal a pathway in which physiological BT variation regulated both the quantity and type of secreted tau species. This regulation occurred through the modulation of temperature-dependent activity of multiple components of the UPS-I pathway (Figure 7). A higher BT during wakefulness increased extracellular tau secretion by (i) promoting its dephosphorylation at S422, (ii) enhancing caspase 3–mediated cleavage of tau into TauC3, (iii) facilitating TauC3 sequestration at the plasma membrane through PIP_2_ binding, and (iv) initiating the translocation of tau into the extracellular space via SDC3. Our findings in humans showed that plasma tau levels in older adults were higher after wakefulness compared with after sleep, similar to previous findings for CSF. This variation in plasma and CSF tau levels strongly correlated with BT changes. These findings support the hypothesis that BT regulates plasma and CSF tau levels by modulating tau secretion, among other potential temperature-dependent molecular and physiological mechanisms. An important clinical implication is that BT may be informative for understanding variability in plasma tau levels, which is relevant to its utility as a clinical biomarker.

The precise mechanisms behind the sleep-wake variation in extracellular tau levels and tau secretion remain unestablished. Our findings suggest that temperature-dependent tau release implicates core BT as a key regulator of tau secretion. Holth et al. previously reported a 2-fold increase in ISF and CSF tau levels during wakefulness compared with sleep (2). We replicated this observation by varying temperature alone within the physiological range, with extracellular tau doubling at 38°C compared with 35°C. Neuronal activity, which is potentiated during wakefulness and reduced during sleep (37), was one of the first biological processes identified as a driver of tau release (5, 6). Holth et al. demonstrated that inhibiting neuronal activity with tetrodotoxin (TTX) prevented tau release during SD (2). However, TTX also causes hypothermia (38, 39), pointing to a possible effect of temperature in these findings. Notably, even a 1°C change in brain temperature can alter neuronal excitability and activity (40–42), and we observed that such a change affects tau secretion. Given that SH-Tau3R cells and mouse primary neurons lack neuronal activity (43), our data strongly support BT as a direct regulator of UPS-I–mediated tau secretion. However, considering that neuronal activity alone can also drive tau release (5), the interplay between neuronal activity and BT in stimulating tau secretion requires further investigation.

Our study emphasizes tau cleavage into TauC3 as pivotal for secretion, with CSF and extracellular tau mainly present in C-terminally truncated form (18, 23). TauC3-specific antibodies have been shown to impede tau propagation and seeding (44), but how diurnal rhythms regulate these processes remains unexplored. We observed that wakefulness temperatures induced tau dephosphorylation at S422, enabling tau cleavage, and upregulated caspase 3–mediated TauC3 truncation, leading to its extracellular release. Our investigation revealed an inverse relationship between TauC3 and S422 phosphorylation, modulated by physiological sleep-wake BT variation. Wakefulness temperatures may drive these processes by increasing protein phosphatase 2A (PP2A) activity as we previously reported (19). PP2A dephosphorylates tau at S422 (45) and is bidirectionally linked to caspase 3, with each enzyme amplifying the activity of the other (46, 47), further enhancing TauC3 production. These findings also imply a physiological tau release, consistent with prior studies showing that tau secretion does not necessarily result in spreading of neuronal pathology (5, 6). In favor of this view, anti–p-S422 antibody treatment has been shown to reduce AD pathology while increasing plasma tau concentrations in AD mice (48), suggesting that TauC3 might be more prone to brain clearance. Although wakefulness temperatures induce tau dephosphorylation at multiple sites, the significance of other phosphorylation epitopes in regulating tau secretion remains to be explored.

The role of S422 phosphorylation and TauC3 continues to be debated. TauC3 is typically linked to the progression of tau pathology in AD, where it is associated with hyperphosphorylation and neurofibrillary tangle (NFT) formation (26, 49, 50). However, these findings often emerge from studies involving transgenic mice or patients with AD who have abnormal tau hyperphosphorylation. Conversely, emerging evidence suggests that TauC3 may have neuroprotective roles under physiological conditions. Some studies report no correlation between TauC3 and NFT formation or cognitive decline in AD (51, 52), whereas others have shown evidence that TauC3 may help eliminate toxic tau species or inhibit hyperphosphorylated tau accumulation (53, 54). Voss et al. (55) proposed that TauC3 is probably degraded by autophagy pathways under physiological conditions (56), reducing its seeding potential. However, in AD, hyperphosphorylation at S422 overwhelms neuronal degradation systems, leading to TauC3 accumulation. Our data support this model, showing that higher temperatures increased TauC3 levels while globally dephosphorylating tau, including at S422, a state unlikely to promote tau seeding. Furthermore, there is no compelling evidence that TauC3 is more prone to seeding than other hyperphosphorylated tau species. While TauC3 accumulation might occur under pathological conditions, its role in tau seeding remains uncertain and warrants further investigation.

We identified several mechanisms by which tau secretion is modulated through interactions with temperature-sensitive components of the UPS-I pathway. Our results suggest that the temperature-dependent cleavage of tau into TauC3 may serve as an initiating factor for finely modulating its secretion. The loss of the microtubule-binding capacity of TauC3 (50) likely enhances its availability for the secretion pathway, whereas wakefulness temperatures facilitate TauC3 binding to PIP_2_ at the inner plasma membrane. Prior research has demonstrated that the tau C-terminal domain contains a low-affinity site for phosphoinositides (57), which likely explains the preferential binding of PIP_2_ to TauC3, as the truncation removes part of the C-terminal. Altogether, these findings suggest that a higher core BT during wakefulness, SD, or mild-hyperthermia promotes TauC3 binding to PIP_2_ at the plasma membrane, initiating the export process.

The increase in BT during wakefulness promotes the extracellular release of TauC3 by enhancing its interaction with SDC3 and thus facilitating the membrane translocation process. Although SDC3 upregulation has been observed in AD mouse models (31) or following neuronal stimulation (58), its temperature-dependent expression and metabolism remain unexplored. Notably, 1 study showed that the glycosyltransferase activity of enzymes such as EXT1 — required for elongating SDC3 sulfate chains — increases with temperature (59). Our study extends these findings, showing that wakefulness temperatures enhance *EXT1* mRNA expression, potentially improving SDC3 functionality. We also observed a higher intracellular colocalization of SDC3 and TauC3 at wakefulness temperatures, furthering our understanding of the mechanisms underlying tau secretion during the sleep-wake cycle.

Our findings suggest that sleep-wake BT variation may modulate physiological CSF and plasma tau dynamics in humans. To our knowledge, this study is the first to report diurnal variation in plasma tau in older adults under naturalistic conditions similar to those found in clinical settings. A previous study in young men under sedentary conditions found no significant morning-evening differences in plasma tau levels (60). Our finding of, on average, approximately 15% higher plasma tau levels in the evening included some variability in ΔTau values, which was correlated with variability in ΔBT. An important clinical implication of this finding is that measuring BT may help explain variability in CSF and plasma tau levels, including both the time-of-day and inter-individual variability. However, systematic studies are needed to determine the optimal timing of BT measurements relative to blood or CSF sampling, given the uncertain delay between BT-driven brain processes and the appearance of tau in the circulation. It should not be assumed that BT at the time of blood or CSF sampling will be most highly correlated with tau outcomes. Further studies may also examine the influences of sleep quality, which is likely to interact with that of BT, and constant routine protocols in which light exposure is controlled for may examine circadian influences.

While the findings in humans support our in vitro and in vivo results, further research is needed to determine the explicit role of tau secretion among other temperature-dependent processes. Important candidates include neuronal activity and sleep, which are known to depend on body and brain temperature variation (61). For instance, chronic thermoneutral temperature exposure in AD mice reduced amyloid pathology by enhancing slow-wave sleep, showing how BT may influence AD biomarkers through sleep (62). Bidirectional effects might also explain the BT and tau dynamic relationship, as early tau pathology in thermoregulatory brain areas — a known feature of AD (13, 63) — can alter BT patterns, as shown in mice (64). Finally, in the setting of AD, further research is needed to distinguish between circulating tau derived from unconventional versus vesicular secretion (4), impaired degradation and clearance pathways (65), or release after neuronal death (66).

Altogether, our findings suggest that sleep-wake BT variation modulates tau secretion and phosphorylation, linking BT variation to CSF and plasma tau dynamics in humans. This implies that impaired thermoregulation or BT disruption caused by sleep disturbance may contribute to AD pathogenesis and related tauopathies. Understanding natural BT variation during the sleep-wake interval is crucial, particularly in patients with thermoregulatory or sleep deficits, as it may affect CSF and plasma tau levels used for AD diagnosis. We note that few previous studies involving patients with AD measured BT variation over the sleep versus wake interval; rather, most reported BT averaged over the sleep-wake cycle, and meta-analysis showed little difference (0.1°C) between patients with AD and controls (67). By contrast, our findings emphasize the importance of assessing BT dynamics over the sleep-wake interval to better understand how BT interacts with tau metabolism. Previously, we showed that lower waking BT predicts tau pathology, supporting hypotheses that an age-associated BT decline is an AD risk factor (13, 68, 69). Conversely, sleep fragmentation or deprivation (9, 70, 71) and increased nocturnal activity (72, 73) — both risk factors for, and observed in, AD — may prevent the nocturnal BT drop (12), increasing tau secretion and potentially accelerating tau pathogenesis (74, 75).

### Conclusions.

Our model (Figure 7) elucidates how core BT regulates tau secretion by driving UPS-I pathway activity during the sleep-wake cycle. We posit that by interacting with the distinct temperature dependence profiles of tau secretion versus phosphorylation, the physiological pattern of core body and brain temperature variation effectively regulates both the amount and type of tau species secreted (Figure 7 and Supplemental Figure 9) (48, 53, 54, 76). Core BT variation may also influence other processes involved in tau proteostasis, many of which are modulated by sleep-wake and circadian cycles (77). For example, a higher waking BT may coordinate increased tau secretion with heat shock tau chaperone activity to manage extracellular tau levels. This model points toward the importance of maintaining proper BT patterns during the sleep-wake cycle, as disruptions, whether age or AD related, could contribute to tau pathology. Interestingly, interventions that temporarily increase BT, like sauna bathing (78) or physical exercise (79), are beneficial in reducing AD risk (80, 81), enhancing deep sleep (62, 82), and lowering amyloid (62, 81) and tau (19, 81) pathologies in mice. Future studies may examine whether sauna use can delay tau-mediated neurodegeneration by correcting sleep and core BT misalignment associated with thermoregulatory and sleep disturbances in aging and early AD. Finally, while the physiological role of extracellular tau remains enigmatic, it may serve as a signaling molecule that potentially interacts with muscarinic receptors (83). Elucidating the physiological role of neuronal tau secretion (84) and understanding the normal function of extracellular tau could inform therapeutic strategies to impede tau pathology propagation.

## Methods

### Sex as a biological variable

Our study examined male and female animals, and similar findings are reported for both sexes.

### Cell culture

Human SH-SY5Y neuroblastoma cells stably expressing human tau 3 repeat isoform (SH-Tau3R cells, provided by Luc Buée, Université de Lille, Lille, France) were cultured as previously described (85). Briefly, the cells were grown in DMEM/high-glucose medium (11995-065, Thermo Fisher Scientific), supplemented with 10% bovine growth serum (BGS) (heat inactivated, F1051-500ML, MilliporeSigma), 1% glutamine (25030081, Thermo Fisher Scientific), and 1% penicillin/streptomycin (15140-122, Thermo Fisher Scientific). The cell cultures were maintained in a humidified incubator with 5% CO_2_ at 37°C. The cells were grown either in 10 cm Petri dishes or in 6-, 12-, or 96-well plates.

### Primary culturing of neurons

Mouse embryonic cortices (E15–E17) were obtained from transgenic B6.129S2Emx1tm1(Cre)Krj/J mice (Emx1-Cre crossed with Red Fluorescent Protein-Lox mice, The Jackson Laboratory). After dissection, meninges, choroid plexus, and hippocampi were removed, and the cortices were enzymatically and mechanically dissociated using 0.25% trypsin-EDTA (Gibco, Thermo Fisher Scientific) at 37°C for 20 minutes. The cell suspension was filtered through a 70 μm strainer and plated at 200,000 cells/well in 6-well plates precoated with 50 μg/mL poly-d-lysine (A3890401, Thermo Fisher Scientific), or at 150,000 cells/well on coverslips precoated with 1 μg/mL polyethylenimine (043896.03, Thermo Fisher Scientific) and 50 μg/mL poly-d-lysine in 24-well plates. The cells were firstly grown for 2 hours in DMEM/high-glucose medium, supplemented with 10% BGS and 1% of streptomycin/penicillin antibiotics in a 5% CO_2_ humidified incubator at 37°C. Then, the medium was replaced with neurobasal medium (21103-049, Thermo Fisher Scientific), supplemented with 1% glutamine (25030081, Thermo Fisher Scientific), 2% B-27 (17504044, Thermo Fisher Scientific), 1% N-2 (17502-048, Thermo Fisher Scientific), and 1% penicillin/streptomycin. The cultures were maintained at 37°C in a humidified 5% CO_2_ atmosphere, with experimental treatments conducted after a 4-day growth period.

### Temperature exposures and cell treatments

Before treatment, the medium was replaced with fresh DMEM (BGS-free) for the SH-Tau3R cells or neurobasal medium for the primary neuronal cultures. Cells were exposed to 35°C, 37°C, 38°C, or 39°C in CO_2_ incubators for 6–72 hours (Figure 1, A and G). Caspase 3 was inhibited by treating cells with 20 μM of the selective caspase 3 inhibitor z-DEVD-FMK (A13503, Adooq Biosciences) for 72 hours (18) dissolved in PBS containing 0.1% of DMSO. siRNA transfection was performed using Lipofectamine RNAiMAX (13778075, Thermo Fisher Scientific) according to the manufacturer’s instructions. Cells were cultured in Opti-MEM (Thermo Fisher Scientific) with 40 μL Lipofectamine and 100 nmol siRNAs for 72 hours. The following siRNAs were used: Silencer Predesigned EXT1 siRNA (ID116802, Thermo Fisher Scientific), Stealth RNAi^TI^ SDC3 siRNA (HSS145253, Thermo Fisher Scientific), and SignalSilence Caspase 3 siRNA (6466S, Cell Signaling Technology). The Silencer select NC siRNA (4390843, Thermo Fisher Scientific) was used as a scrambled control.

### Animals

Three-month-old male and female C57BL6 mice, 18-month-old male hTau mice, and tau-KO (TKO) littermates (86) were used in the experiments. hTau mice were generated by crossing 8c mice expressing the 6 nonmutated human tau isoforms (87) with TKO mice (88), both originating from mice on a C57BL6 background [B6.Cg-Mapttm1(EGFP)Klt-Tg(MAPT)8cPdav/J, The Jackson Laboratory]. Mice had ad libitum access to water and food, and were housed on a 12-hour light/12-hour dark cycle, with lights on at 7:15 am. At the end of each experiment, mice were euthanized by decapitation without anesthesia, which induces tau hyperphosphorylation (89, 90). The brains were promptly removed, and cortices were dissected on ice, frozen in liquid nitrogen, and stored at –80°C for further analysis.

### Rectal temperature measurements

Mice were handled using the “scruff method” and fitted with a rectal probe (RET-3, Brain Tree Scientific) connected to a digital thermometer (Thermalert TH5, Physitemp).

### Sleeping versus awake mice

Mice were subjected to a continuous period of darkness lasting for 3 days. The determination of subjective day was done as previously described (12). Briefly, sleeping C57BL6 mice (*n* = 5 males and *n* = 5 females) were euthanized between 10:30 am and 11:30 am local time (at circadian time 4 [CT4]), and active mice (*n* = 5 males and *n* = 5 females) were euthanized between 10:30 pm and 11:30 pm local time (CT16) (Figure 5A). Furthermore, the sleeping criterion corresponded to mice in the nest in a “resting posture,” as elucidated by Thoman and Carroll (91). The rectal BT of mice was recorded just before and after thermal exposure.

### SD experiment

As previously described (12), a subset of C57BL6 mice was intentionally kept awake for the first 6 hours of the light period (SD group, *n* = 4 males and *n* = 5 females), according to a gentle handling procedure. Naive mice (*n* = 3 males and *n* = 4 females) were allowed to sleep without any disturbance. All mice were euthanized at the end of the SD period (Figure 5E). Prior to the SD experiment, a subset of 5 mice from both groups was abdominally implanted with telemetric probes (BodyCap, Anipill) enabling continuous monitoring of their BT.

### Cold and heat exposures

On the day before the experiment, hTau mice were individually housed to prevent mutual heating. Throughout the study, mice in the naive group and the NC TKO mice remained at the standard temperature of the experimental room (22°C). The 2 other groups of mice underwent a 4-hour exposure period either at 4°C or 38°C. Experiments were started at 6:45 am, 30 minutes before the onset of the light phase, to avoid disturbing the sleep cycle of the mice. All temperature exposures were conducted in a dark environment to minimize the risk of the mice falling asleep. The wakefulness of the mice was visually controlled every hour during the 4-hour experiment. The rectal BT of the mice was recorded just before and after thermal exposure.

### CSF collection

The mice were anesthetized with isoflurane and placed in a stereotaxic instrument with core BT maintained using a water heating pad. Under a dissection microscope, subcutaneous tissues and muscles (musculus [m.] biventer cervicis and m. rectus capitis dorsalis major) were gently separated using blunt dissection with forceps, exposing the dura mater of the cisterna magna. A capillary tube was introduced through the dura mater into the cisterna magna to collect CSF.

### Protein extraction

All samples were homogenized by sonication in RIPA buffer and then centrifuged for 20 minutes at 20,000*g* at 4°C. The resulting supernatant was collected, and the total protein concentration was assayed (Pierce BCA Protein Assay Kits, 23225, Thermo Fisher Scientific). The samples were diluted in sample buffer (NuPAGE LDS, Invitrogen, Thermo Fisher Scientific) containing 5% of 2-ME, 1 mM Na_3_VO_4_, 1 mM NaF, 1 mM phenylmethylsulfonyl fluoride (PMSF, and 10 μL/mL Proteases Inhibitor Cocktail (P8340, MilliporeSigma). The samples were then denaturated at 95°C for 10 minutes.

### Western blotting

Western blot analysis was conducted as previously described (92). Samples (10–20 μg) were separated on an SDS-10% polyacrylamide gel and transferred onto nitrocellulose membranes (Amersham Biosciences). The membranes were saturated, hybridized with the appropriate antibodies, and revealed as previously described (92). For immunoblots targeting p-tau epitopes, the signal was normalized to the total tau protein. As a loading control, other proteins were normalized to β-actin. Representative lanes from the immunoblots are presented for each specific experimental condition. The dashed lines indicate segments where certain lanes from the same blot were excluded, and the remaining lanes were combined. Brightness levels were adjusted as necessary to enhance visualization and accuracy.

### Antibodies

Detailed information on the antibodies used is provided in Supplemental Table 3.

### Dot blotting

Cell media were centrifuged at 20,000*g* at 4°C for 10 minutes. The resulting supernatant (100 μL) was deposited onto nitrocellulose membranes (Amersham Biosciences) using a microfiltration blotting apparatus (1706545, Bio-Rad). The membranes were saturated, hybridized with the appropriate antibodies (Supplemental Table 3), and the signal was detected as described previously (92). For dot blots targeting p-tau epitopes, the signal was normalized to total tau protein. Other proteins were normalized to the respective extracellular lactate dehydrogenase (LDH) value (CytoTox96 Assay, Promega). All LDH results are displayed in Supplemental Figure 10. Representative dot signals are presented for each specific experimental condition. The dashed lines indicate segments where certain dots from the same blot were excluded, and the remaining dots were combined. Brightness levels were adjusted as necessary to enhance visualization and accuracy.

### Co-IP

Co-IP analyses were performed following the manufacturer’s instructions (Pierce Classic Magnetic IP/Co-IP Kit, 88804, Thermo Fisher Scientific). SH-Tau3R cells were lysed, incubated at 4°C for 5 minutes, and centrifuged at 13,000*g* to remove debris. The supernatants were adjusted to 500 μg proteins and incubated overnight at 4°C on a rotating device with primary antibodies (Supplemental Table 3), except for the NC sample. Protein A/G magnetic beads (25 μL) were added to each sample, incubated for 1 hour at room temperature, and washed 3 times after magnetic separation. The beads were eluted with elution buffer and separated magnetically, and 10 μL neutralization buffer was added to the supernatant. The resulting sample was diluted with sample buffer (NuPAGE LDS, Invitrogen, Thermo Fisher Scientific) containing 5% of 2-ME, 1 mM Na_3_VO_4_, 1 mM NaF, 1 mM PMSF, and 10 μL/mL Protease Inhibitor Cocktail and then boiled at 95°C for 5 minutes before Western blot analysis.

### Immunocytochemistry

Primary neurons were fixed in PBS (311-010-CL, Multicell) with 4% paraformaldehyde (19210 Electron Microscopy Sciences, Thermo Fisher Scientific) and 10% sucrose for 20 minutes at room temperature. Cells were washed 3 times with PBS, permeabilized with 0.2% Triton X-100 (T8787-100ML, MilliporeSigma) in PBS for 30 minutes, and blocked with 5% heat-inactivated goat serum (G6767, MilliporeSigma) in PBS for 1 hour at room temperature. Cells were incubated with primary antibodies (Supplemental Table 3) in 5% heat-inactivated goat serum in PBS at 4°C overnight. Following 3 PBS washes, the secondary antibodies anti–mouse Alexa Fluor 488 diluted at 1:1,000 (A-11029, Thermo Fisher Scientific) and goat anti–rabbit IgG Alexa Fluor 633 diluted at 1:1,000 (A-21070, Thermo Fisher Scientific) were added for 2 hours. Following 3 PBS washes, DAPI (Thermo Fisher Scientific, 3.5 μL in 25 mL PBS) was used to stain nuclei, and coverslips were mounted with Fluoromount-G (00-4958-02, Invitrogen, Thermo Fisher Scientific). Imaging was conducted using a Zeiss LSM800 confocal microscope, and images were processed with Zen Blue Edition software (version 2.3, Carl Zeiss).

### Caspase 3 activity assay kit

The SH-Tau3R cells were cultured in 96-well plates and treated according to the particular experimental conditions (Figure 1A). The Colorimetric Caspase 3 Assay Kit (ab39401, Abcam) was used to determine caspase 3 activity according to the manufacturer’s instructions.

### Membrane fluidity

SH-Tau3R cells were cultured in 96-well plates and treated according to the experimental conditions (Figure 1A). Membrane fluidity was assessed following the manufacturer’s instructions. The cells were incubated for 1 hour at 35°C, 37°C, or 38°C in medium containing 5 μM Fluorescent Lipid Reagent and 0.08% Pluronic F127, (ab189819, Abcam). The fluorescence intensity was measured (Infinite F200, Tecan) with excitation at 350 nm and emissions at 400 nm (monomer) and 470 nm (excimer). Fluorescence values were blank corrected, and the ratio of excimer to monomer emissions was calculated to determine membrane fluidity.

### ELISAs of extracellular tau

Total and p-tau concentrations within the cell medium were quantified using ELISA kits (Tau [total] Human KHB0041; Tau [pS199] Human KHB7041; Tau [pT231] Human KHB8051; Tau [pS396] Human KHB7031; Tau [total] Mouse KMB7001, Thermo Fisher Scientific). Prior to analysis, the samples were suitably diluted in diluent buffer (1:50 for human tau, 1:2 for mouse tau and p-tau). ELISAs were performed in accordance with the instructions provided by the manufacturer.

### Plasma tau assay

Blood samples were collected upon euthanasia (BD vacutainer EDTA), centrifuged at 4°C, and plasma was stored at –20°C until the tau assay. Total tau concentrations in the plasma of hTau mice were quantified using the Meso Scale Diagnostics R-Plex Human Tau (total) assay (K151AGTR-2, MSD). Prior to analysis, the samples were suitably diluted in diluent buffer (1:2). The assays were performed in accordance with the instructions provided by the manufacturer and read with a MESO QuickPLex SQ 120MM instrument.

### Quantitative PCR

Total RNA was extracted from SH-Tau3R cells using TRIzol reagent (Life Technologies, Thermo Fisher Scientific) in accordance with the manufacturer’s instructions. RNA quantification was performed, and 1 μg total RNA was used for cDNA synthesis using the iscript cDNA Synthesis Kit (Bio-Rad), containing an optimal blend of oligo-dT and random primers. For subsequent PCR amplification, 1 μL of the resultant cDNA was used as a template. The primer sequences are listed in Supplemental Table 3. The quantitative PCR (qPCR) mix was formulated with 18 μL per 2 μL of 20 ng cDNA. The mix consisted of 0.5 μL of both the forward and reverse primers, 10 μL SYBR Green PCR Master Mix (Applied Biosystems), and 7.5 μL nuclease-free water. The qPCR program began with a hot start at 95°C for 3 minutes, succeeded by 40 cycles at 95°C for 15 seconds, and then 60°C for 1 minute using a LightCycler 480 II apparatus (Roche). The melting curves were evaluated to ensure a single PCR product. To quantify cDNA levels, the comparative 2ΔΔCt method was used. Ct values corresponding to the target gene were normalized to the Ct values of the housekeeping gene *GAPDH*. The results were expressed as n-fold differences relative to the experimental control.

### Human studies

#### CSF temperature correlations.

Details regarding participants, CSF collection, and study design are described by Lucey et al. (93). Thirteen cognitively unimpaired participants from the placebo group of a clinical trial provided 6 mL CSF every 2 hours over 36 hours via lumbar catheterization (93). All participants were in good health, except for poor sleep efficiency (<85%) measured via actigraphy. BT was recorded every 4 hours using an oral thermometer (Welch Allyn SureTemp Plus, Model: 692). CSF tau forms (T181, S202, T217) were measured by immunoprecipitation/mass spectrometry (93), and NfL levels were quantified using the NF-light ELISA kit (UmanDiagnostics) following the manufacturer’s protocol. The assay’s measurement range is 100 pg/mL to 10,000 pg/mL, with a detection threshold of 33 pg/mL. Samples were diluted 1:100 with Sample Diluent (UmanDiagnostics) before analysis. Absorbance was measured at 450 nm with a reference wavelength of 620–650 nm. Positive control samples containing high NfL levels (“bloody CSF”) were diluted to 1:1,000 and used to ensure consistency across plates. CSF tau-181, tau-202, and tau-217 concentrations were averaged at 8 am, 4 pm, and 8 pm. Differences between temperature, CSF tau levels, and CSF NfL levels were calculated for analysis (Supplemental Table 1). NfL was selected as the control protein because its soluble concentration is not affected by sleep-wake activity (2). We selected time points of 8 am versus 4 pm for post-sleep versus post-wakefulness tau levels, which also corresponded to the minimum BT and maximum BT, respectively. These intervals were selected on the basis of the following rationale. First, we assumed little delay between tau secretion and the appearance of tau in the CSF, meaning that BT taken at the time of CSF collection would roughly reflect brain temperature at the time of tau secretion. Second, we selected 8 am versus 4 pm as the interval that maximized that difference in sleep versus wake temperatures, given that BT was not recorded during sleep and, for the majority of participants, had already begun to drop between 4 pm and 8 pm (Supplemental Table 1). Because of the BT drop at the end of the day, no significant correlation was observed between ΔBT and ΔTau in CSF between 8 am and 8 pm (Supplemental Figure 7).

#### Plasma temperature correlations.

Data were collected from 24 older adults aged 68.39 ± 5.25 years, including 17 women. The participants were enrolled in a cross-sectional study examining relationships between core BT and plasma and PET AD biomarkers. The participants were cognitively normal (*n* = 21) or had mild cognitive impairment (*n* = 3) based on the clinical dementia rating (CDR) scale and were medically healthy, with mild or no sleep apnea. The participants underwent screening through interviews and 1 week of home actigraphy for sleep-wake disorders including sleep duration of less that 6 hours per night and significant phase advance or phase delay. Additional exclusion criteria have been previously described in detail (13) and included AD dementia (CDR >0.5), medical comorbidities, medications that might affect sleep or thermoregulation, major psychiatric disorders, moderate-to-severe substance use disorders, shift work within the previous 6 months, or traveling across 1 or more time zones within 2 weeks of study participation.

The study design was a seminaturalistic protocol fully detailed previously (13). Participants underwent continuous core BT monitoring using an ingestible telemetric device (CorTemp), which sampled BT every 15 seconds with an accuracy of 0.2°C for a minimum of 36 hours spanning 2 nights. During this time, 2 in-laboratory nocturnal polysomnograms were measured, and participants were free to behave as they chose during the intervening day between the laboratory nocturnal recordings. The goal of this design was to capture data that most closely represented the typical BT for each participant. Blood draws for plasma tau were collected at 4 time points: morning (7 am) and evening (7 pm) on 2 consecutive days (Supplemental Table 2). Prior to analysis, the temperature data were preprocessed to exclude gaps and artifacts as detailed in a previous report (13). Data presented for BT-tau correlations comprised tau levels from night 2 and morning 2, given that BT was not always measured prior to the blood draw on night 1 (Supplemental Table 2). Paired tau levels and BT data were obtained for 15 participants (Supplemental Table 2). For BT-tau correlations, the difference between the average BT between 6–7 pm and 1–2 am was calculated. This interval was chosen because these times represented the sample average minimum and maximum BTs, and as such their difference maximized the diurnal BT difference, or ΔBT. Plasma sampling times were selected to maximize efficiency in collecting data. Food intake was not regulated, but the morning sample was typically obtained before the morning meal, whereas the evening sample was typically obtained before the evening meal. Concentrations of plasma tau were measured using the neurology 3-PLEX kit and Simoa HD-X instruments (Quanterix) at the NYU Alzheimer’s Disease Center Biomarker Core according to the manufacturer’s instructions. Plasma extraction was performed as described previously. Assays were run in duplicate to obtain interassay coefficient of variations (CVs). The inter-assay CV was under 20% for all samples.

### Proteomics and bioinformatics analyses

Detailed information is provided in the Supplemental Methods.

### Brain temperature recordings

Detailed information is provided in the Supplemental Methods.

### Generative AI program use

We used ChatGPT (o1 version) between January 1, and May 31, 2024, submitting all of the draft paragraphs we had written with the following prompt: “Please improve the scientific writing and readability of this text.”

### Statistics

A minimum of 2 distinct experiments were carried out for each experimental condition. Prior to conducting each analysis of variance, an assessment of Gaussian distribution was performed, and its validity was confirmed through a Kolmogorov-Smirnov test (using GraphPad Prism 9.0). Depending on the specific analysis, a 2-tailed *t* test (or Mann-Whitney *U* test), as well as 1- or 2-way ANOVAs (or Kruskal-Wallis tests) were applied. Post hoc analyses, involving either Tukey’s or Dunnett’s tests, were subsequently applied. A significance threshold of *P* < 0.05 was used to determine statistical significance. The presentation of data incorporated either box and whisker plots (illustrating the range from minimum to maximum values, encompassing the median) or the mean ± SEM. The scatter plots depicted on each graph provide an indication of the number of data points, and detailed statistical information is provided in Supplemental Table 4.

### Study approval

All procedures followed the protocols of the Animal Care Committee of Université Laval (CPAUL-3, approbation number: CHU-22-1027) under the guidelines of the Canadian Council on Animal Care.

### Data availability

The raw data supporting the findings of this study are reported in the Supporting Data Values file.

## Author contributions

GC and EP conceived of the preclinical studies, designed experiments, and analyzed the data. EMB conceived of and designed the human BT and plasma study. BPL, HL, and WL conceived of and designed the human CSF studies. FDGM performed dissection and seeding of primary cultures. GC, ER, SDD, FL, E. Boscher, BK, PFF, and SC performed WB and dot blot experiments. FL performed qPCR experiments. GC performed ELISA assays, co-IP, membrane fluidity assays, and caspase 3 activity assays. GC and FDGM performed IHC, microscopy imaging, and analysis. GC and IG designed and performed sleep-wake and SD experiments in WT mice and performed surgical implantation of abdominal probes for temperature recordings. GC and FL performed heat and cold exposures in hTau mice. NF performed mouse CSF collection. FL maintained animal colonies and genotyped the animals. GC, KG, and AL conducted the proteomics analysis. MPB performed the brain temperature recordings in mice. JK, JH, and DV conducted the human plasma studies. AY, HTW, and AP processed and analyzed temperature and tau data. E. Blank conducted cognitive screening, DMR, IA, AWV, and RSO supervised and conducted the sleep disorder and actigraphy screening. LD performed the tau SIMOA assays. GC, EP, and EMB conceived of the study model and wrote the manuscript. SL, SSH, and VP provided review and feedback on the drafts and final manuscript. All authors reviewed, corrected, and approved the final manuscript.

## Supplementary Material

Supplemental data

Unedited blot and gel images

Supporting data values

## Version 1 02/04/2025

In-Press Preview

## Version 2 04/01/2025

Electronic publication
