# Supplementary material for: Sleep-wake variation in body temperature regulates tau secretion and correlates with CSF and plasma tau
Source: J Clin Invest. 2025 Feb 4;135(7):e182931. doi: 10.1172/JCI182931 (PMC11957704; doi:10.1172/JCI182931)
Supplement: Supplemental data [file jci-135-182931-s040.pdf]

Supplementary Material for

Sleep-wake body temperature variation regulates tau secretion and correlates with CSF and plasma tau

Geoffrey Canet[1,2*], Felipe Da Gama Monteiro[1,3], Emma Rocaboy[2], Sofia Diego-Diaz[2], Boutheyna Khelaifia[1,2], Kelly Godbout[2], Aymane Lachhab[2], Jessica Kim[4], Daphne I. Valencia[5], Audrey Yin[4], Hau-Tieng Wu[4], Jordan Howell[4], Emily Blank[4], Francis Laliberté[1], Nadia Fortin[1], Emmanuelle Boscher[1,2], Parissa Fereydouni-Forouzandeh[2], Stéphanie Champagne[2], Isabelle Guisle[1,2], Sébastien S. Hébert[1,2], Vincent Pernet[1,3,6,7,8], Haiyan Liu[9], William Lu[9], Ludovic Debure[4], David M. Rapoport[5], Indu Ayappa[5], Andrew W. Varga[5], Ankit Parekh[5], Ricardo S. Osorio[4], Steve Lacroix[1,3], Mark P. Burns[10], Brendan P. Lucey[9], Esther M. Blessing[4*], Emmanuel Planel[1,2*]

***CORRESPONDING AUTHORS**

Dr. Geoffrey CANET	Dr. Emmanuel PLANEL	Dr. Esther M. BLESSING
Email: geoffrey.canet.1@ulaval.ca	Email: emmanuel@planel.org	Email: esther.blessing@nyulangone.org
Phone: 1-418-525-4444	Phone: 1-418-525-4444	Phone: 1-646-754-4808

This PDF file includes:

- Supplemental Methods
- Supplemental Figures 1 to 10
- Supplemental Tables 1 to 4

SUPPLEMENTAL METHODS

Proteomic and Bioinformatic analyses

Sample preparation: Extracellular proteins from 500 µL of SH-Tau3R cell supernatant were precipitated using cold acetone. After centrifugation, proteins were resolubilized in 50 mM ammonium bicarbonate with 1% sodium deoxycholate, denatured at 95°C for 5 minutes, reduced with 0.2 mM dithiothreitol at 37°C for 30 minutes, and alkylated with 0.8 mM iodoacetamide at 37°C for 30 minutes. Proteins were digested overnight at 37°C with 400 ng trypsin. Samples were acidified with 50% formic acid, dried under vacuum, desalted on C18 StageTips (Empore), and then solubilized in 0.1% formic acid. Peptides were quantified using a Nanodrop2000 (Thermo Fisher Scientific) and 0.1 µg was deposited on Evotip (Evosep, Odense, Denmark).

Mass spectrometry acquisitions: Samples were analyzed on an Evosep One system coupled with an Orbitrap Exploris 480 (Thermo Fisher Scientific). Peptides were eluted from Evotip using the pre-programmed Whisper method for 40 samples/day (33 min gradient) on a 15 cm x 75 µm ID capillary column (1.7 µm particle size, Ion Opticks Aurora Elite TS). Data-independent acquisition (DIA) was employed, with precursors acquired at 120,000 resolution over a 350-1500 m/z range, AGC target set at 300%, and automatic maximum injection time. Precursors were fragmented using HCD at 30%, with fragments acquired over a 350-877 m/z range using 17 windows of 31 m/z at 15,000 resolution. An AGC target of 800% and a maximum injection time of 38 ms were used for fragment ions.

Data analysis: Spectra were analyzed using DIA-NN (version 1.8.1) with an in-silico digested *Homo sapiens* sequence database (UniProt ID UP000005640). Trypsin/P was set as the enzyme parameter, with a maximum of 2 missed cleavages allowed. Carbamidomethylation was set as a fixed modification, with methionine excision and oxidation as variable modifications. Only 2+ to 4+ precursors were considered over a 350-877 m/z mass range, with fragments in the 100-2000 m/z range. Protein intensities were normalized using the MaxLFQ algorithm in R, applying a 1% q-value filter on both precursors and protein groups. Proteins were quantified if they had at least 2 identified peptides and intensity values in at least 75% of replicates in one group. Missing values

were imputed, and significant variations were determined using a limma t-test with a p-value < 0.01.

Bioinformatic: Tau protein interactions were analyzed using ComPPI website, focusing on "Extracellular" and "Secretory-pathway" proteins. Of the 95 matching proteins, 29 were detected in our samples.

Brain-implanted thermocouples and real-time temperature recordings

Brain temperature experiments were approved by the Georgetown University IACUC Committee. For thermocouple recordings of brain temperature, we used a modified thermocouple inserted into the striatum, as described by Lopez-Rodriguez et al. (1). Briefly, isoflurane anesthetized mice were implanted with MBR-5 intracerebral guide cannula (ID 457 µm, OD 635 µm; BASi Research Products) into the striatum at 0.3 mm A/P; ± 2.0 mm M/L; 3.0 mm D/V. The cannula was cemented into place using C & B metabond (Parkell Inc). Immediately following canula placement, an Anilogger core temperature capsule (Texas Scientific Instruments, Boerne, TX) was surgically placed into the intraperitoneal cavity and the site closed with sutures. Mice were administered post-surgery slow-release buprenorphine (Ethiqa-XR, Fidelis Animal Health) and allowed to recover for at least 2 hours in a heated surgical recovery area and in their home cage for at least 7 days prior to brain temperature measurements.

For the simultaneous testing of brain and body temperature, 3 female wild-type mice were recorded in three different environments. A cage at room temperature (20-22°C), a refrigerator (3-5°C) and an incubator (37-38°C). On the day of testing, mice had a modified thermocouple inserted into the guide cannula. The thermocouple was an ultrafast T-type implantable device (IT-23; ADInstruments Ltd.) modified to fit into a microdiaylsis probe using the specifications of Lopez-Rodriguez et al (1) with our only adaptation being that we expanded the opening of the microdialysis probe using a 0.5mm drill. The thermocouple was connected to a BAT-12 physitemp recorder (AD Instruments). Readings were captured from the thermocouple and from the core temperature capsule every 5 min for 4h. We set parameters to remove the mouse from their respective environments if body or brain temperature reached 30°C or 41°C. Two of the three mice

reached 30°C while in the cold environment, and were immediately removed to room temperature and recovered normal body and brain temperature within 45 min.

REFERENCES FOR SUPPLEMENTAL METHODS

1. Lopez-Rodriguez AB, et al. Hyperthermia elevates brain temperature and improves behavioural signs in animal models of autism spectrum disorder. *Molecular Autism*. 2023;14(1):43.

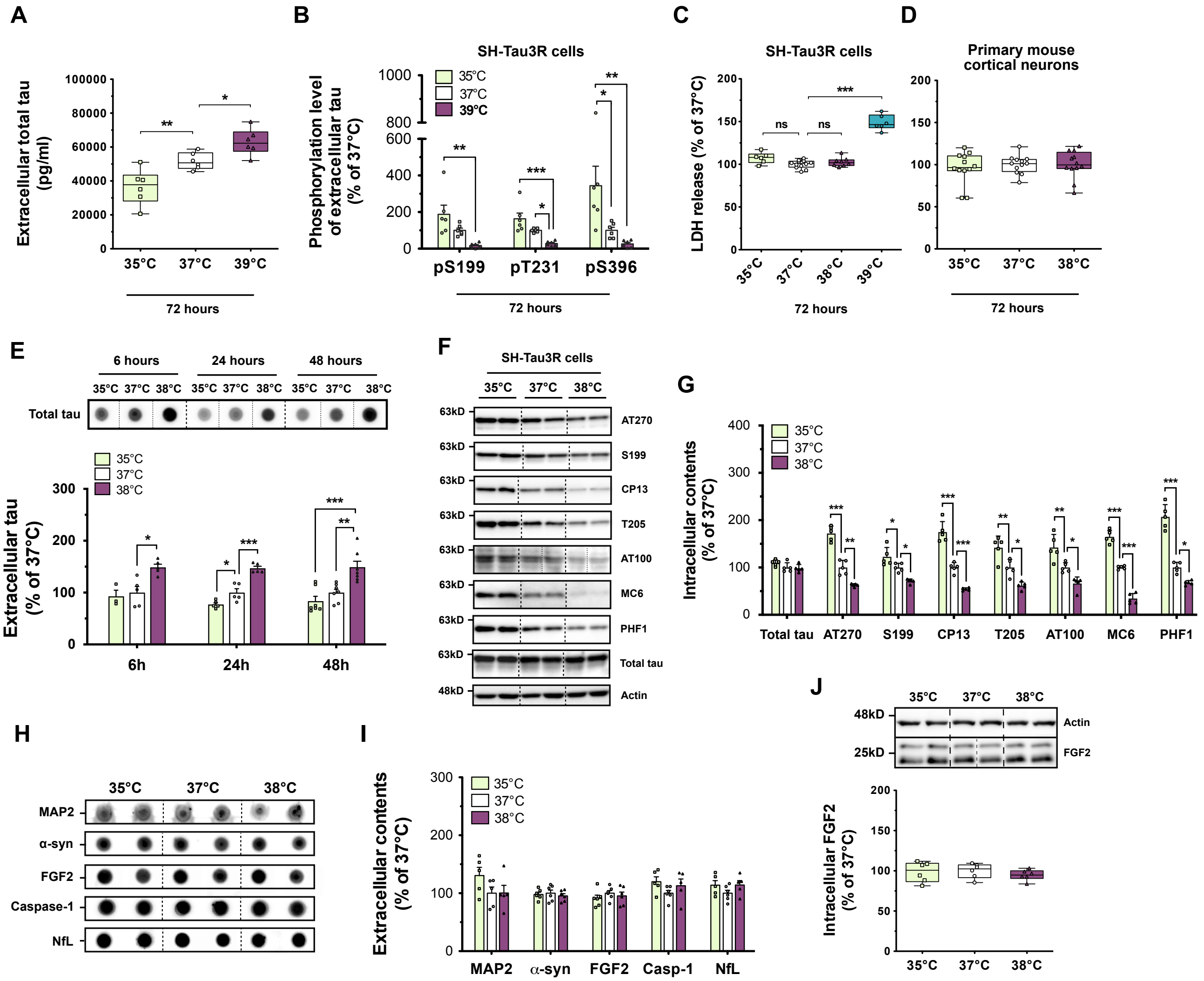

Supplemental Figure 1. Wakefulness temperatures increase tau secretion while decreasing intracellular tau phosphorylation level. (A) The increase in extracellular tau levels (pg/ml) is temperature-dependent in SH-Tau3R cells exposed to 35, 37 or 39°C (*n* = 6; Dunnett's; box and whiskers with minimum to maximum and median). (**B**) The phosphorylation level of extracellular tau at S199, T231 and S396 (ELISA) is decreased at 39°C compared to 35 or 37°C (*n* = 6; Tukey's; mean ± SEM). (**C**, **D**) 72 hours of exposure to temperatures between 35°C and 38°C did not induce cytotoxic LDH release in SH-Tau3R cells or primary mouse cortical (*n* = 6-12; Tukey's; box and whiskers with minimum to maximum and median). **(E)** The temperature-dependent increase in extracellular tau levels is comparable either after 6, 24 or 48 hours of exposure to 35-38°C (*n* = 3-5 for 6-hours; Dunn's; *n* = 5-6 for 24- and 48-hours; Tukey's; mean ± SEM.). (**F, G)** The intracellular phosphorylation level of tau at AT270, S199, CP13, T205, AT100, MC6 and PHF1 is decreased at 38°C compared to 35 or 37°C (*n* = 5; Tukey's; mean ± SEM.). (**H, I)** The extracellular levels of MAP2, α-synuclein, FGF2, Caspase-1 and NfL are not affected by temperature (*n* = 5-6; Dunnet's; mean ± SEM). (**J**) The intracellular expression of FGF2 is not affected by temperature (*n* = 6; Dunnet's; box and whiskers with minimum to maximum and median). *p<0.05, **p<0.01 and ***p<0.001. ns: non-significant.

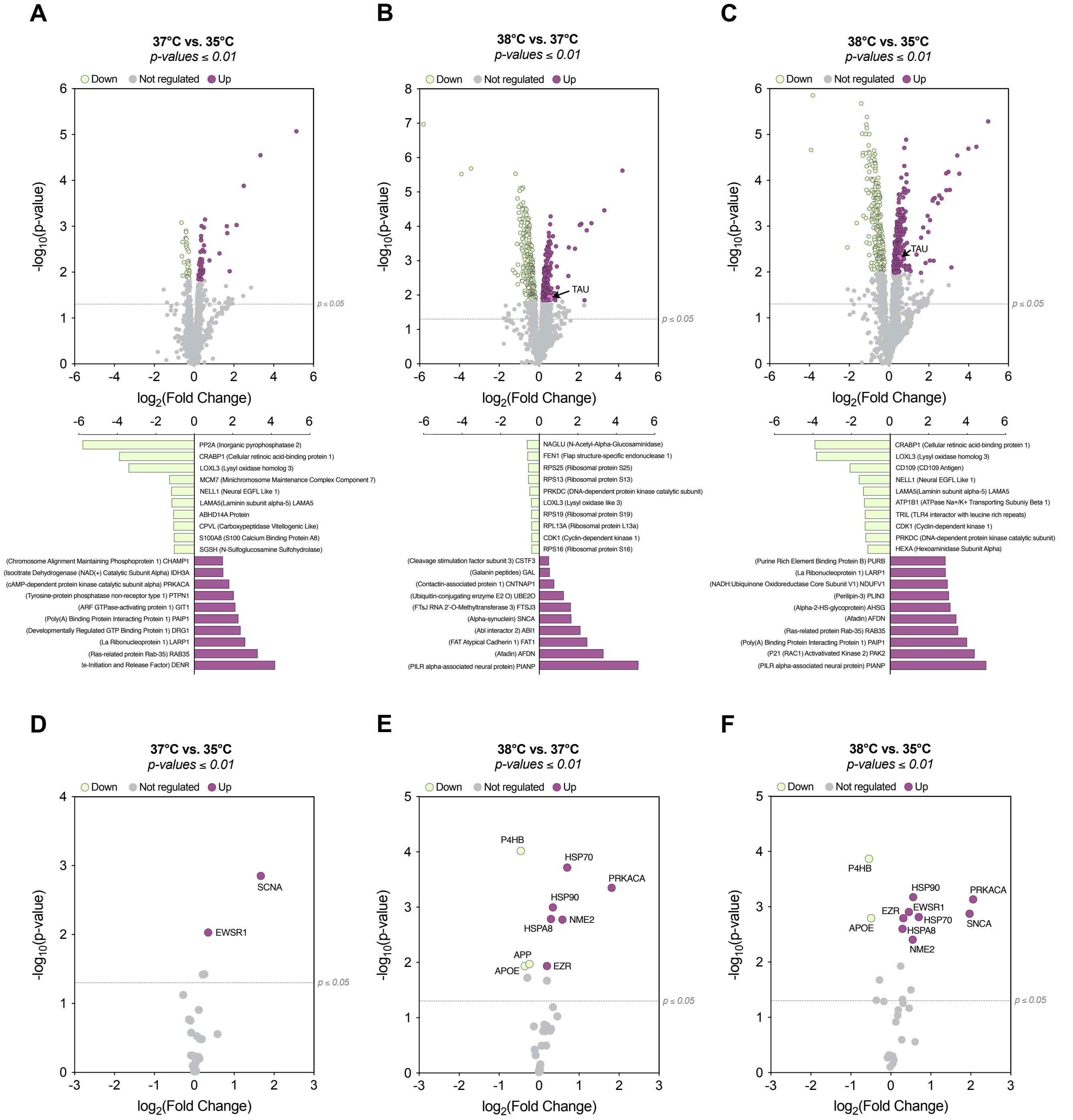

Supplemental Figure 2. Sleep-wake temperatures influence the secretome of SH-Tau3R cells.
Volcano plots of extracellular proteins (**A**) in 37°C condition relative to 35°C condition (34 were downregulated and 45 were upregulated), out of 1,538 total proteins detected; (**B**) in 38°C condition relative to 37°C condition (272 were downregulated and 175 were upregulated), out of 1,620 total proteins detected; (**C**) in 38°C condition relative to 35°C condition (240 were downregulated and 222 were upregulated), out of 1,617 total proteins detected. For each comparison, the 10 proteins that were the most downregulated, as well as the 10 proteins that were the most upregulated, are displayed below and expressed as $\log_2$(Fold Change). Volcano plots of extracellular proteins that are part of "extracellular" and "secretory-pathway" interactomes of tau (obtained with ComPPI website) (**D**) in 37°C condition relative to 35°C condition (2 were upregulated), out of 28 total proteins detected;

(**E**) in 38°C condition relative to 37°C condition (3 were downregulated and 6 were upregulated), out of 29 total proteins detected; (**F**) in 38°C condition relative to 35°C condition (2 were downregulated and 8 were upregulated), out of 29 total proteins detected. $p \leq 0.01$ was considered significant. Abbreviations: APOE (Apolipoprotein E); APP (Amyloid precursor protein); EWSR1 (RNA-binding protein EWS); EZR (Ezrin); HSP70 (Heat Shock protein 70); HSP90 (Heat Shock protein 90); HSPA8 (Heat shock cognate 71 kDa protein); NME2 (Nucleoside diphosphate kinase B); P4HB (Nucleoside diphosphate kinase B); PRKACA (cAMP-dependent protein kinase catalytic subunit alpha); SCNA (alpha-synuclein).

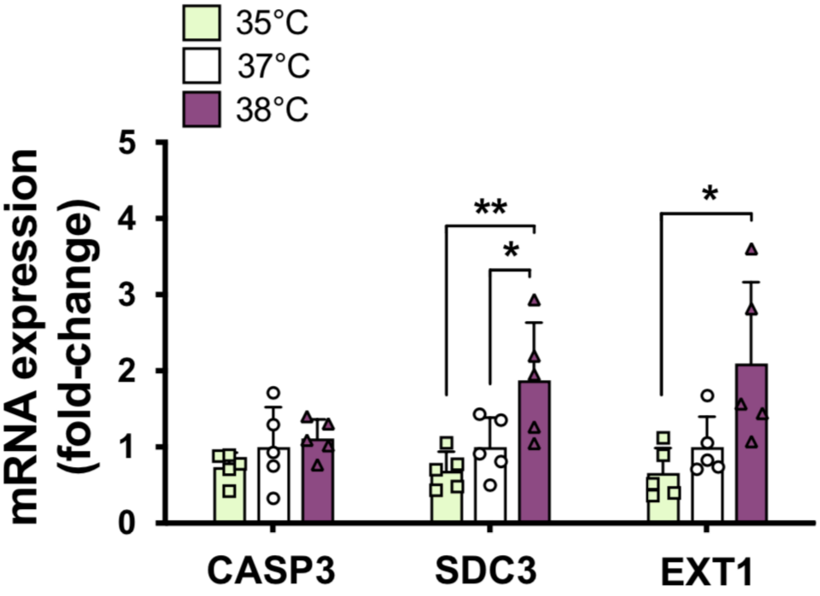

Supplemental Figure 3.
The mRNA expression of *SDC3* and *EXT1* genes are temperature-dependent in SH-Tau3R, and *CASP3* mRNA is unchanged (n = 5; Tukey's; mean ± SEM).

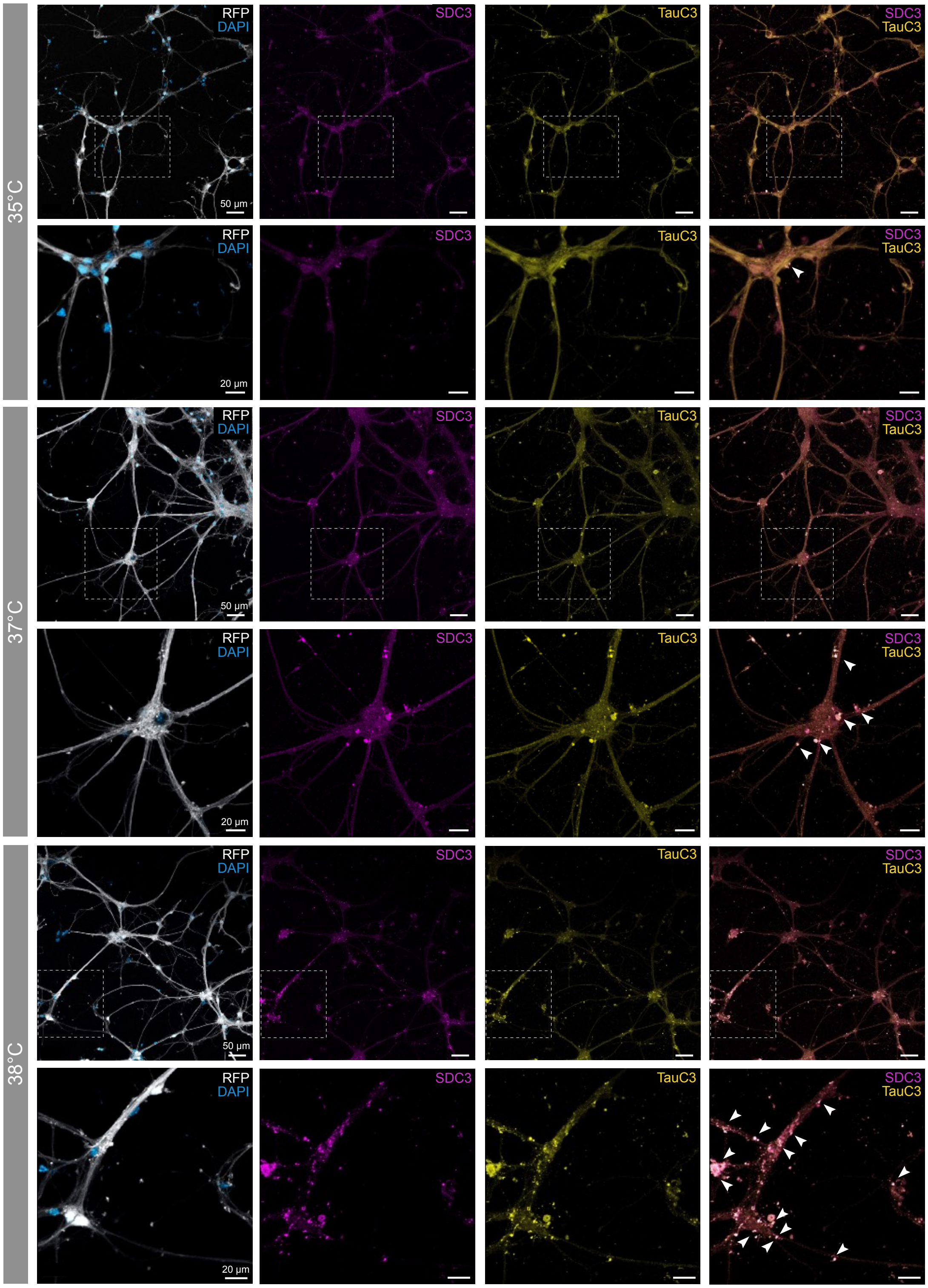

Supplemental Figure 4.

Representative confocal images of primary mouse cortical neurons stained for red fluorescent protein (RFP), DAPI (blue), SDC3 (purple) and TauC3 (yellow). Cells were exposed at 35, 37 or 38°C for 72 hours. A merged staining is displayed, showing a temperature-dependant increase of colocalization between SDC3 and TauC3, and marked with white arrows. Scale bar represents 50 µm for upper panels, and 20 µm for lower panels (magnification of dotted boxes). Data representative of *n* = 3 per condition, examined over 2 independent experiments. Disclosure: Some images in Supplemental Figure S4 were previously presented in Figure 3C.

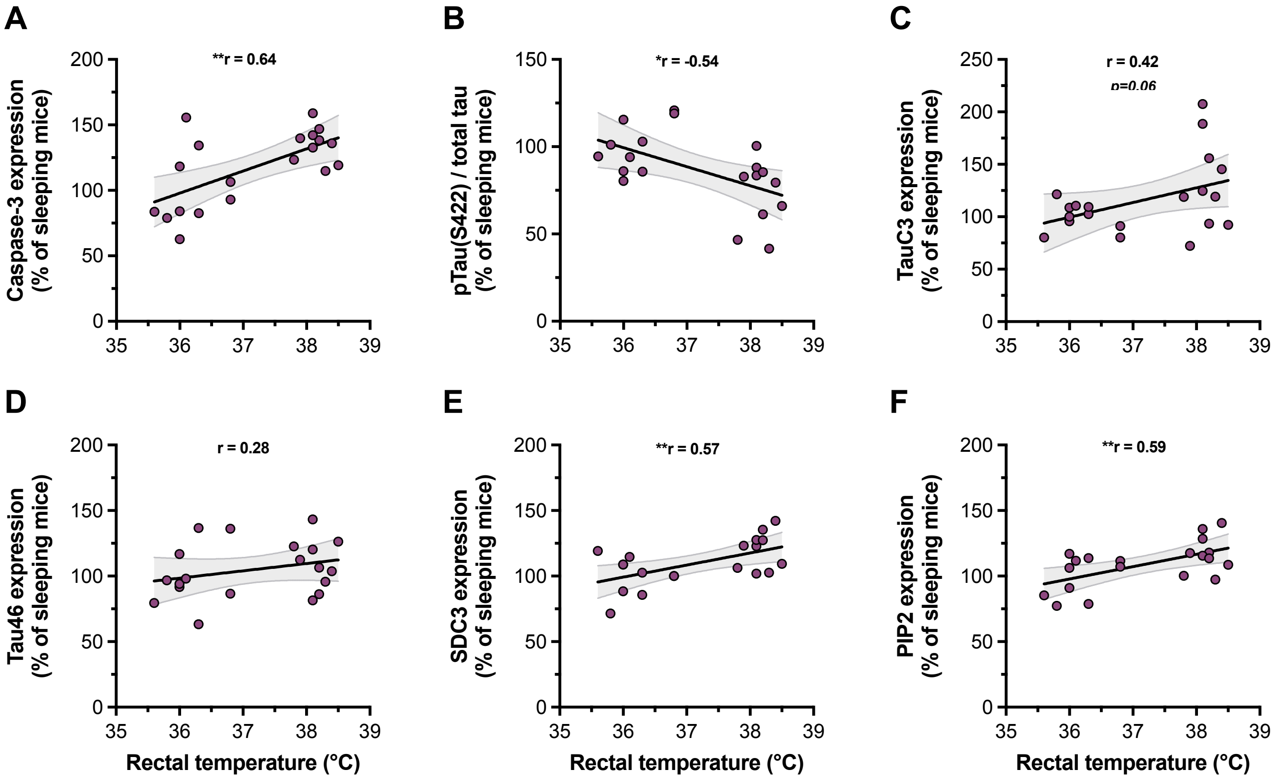

Supplemental Figure 5. UPS-I protein component expressions correlate with body temperature during the sleep-wake cycle in wild-type mice.

(A-F) The cortical expression of caspase-3, pTau(S422), Tau46, SDC3 and PIP_2 are significantly correlated with rectal temperature of mice (Pearson correlation; standard error bars displayed as error envelopes in light grey). *p<0.05 and **p<0.01.

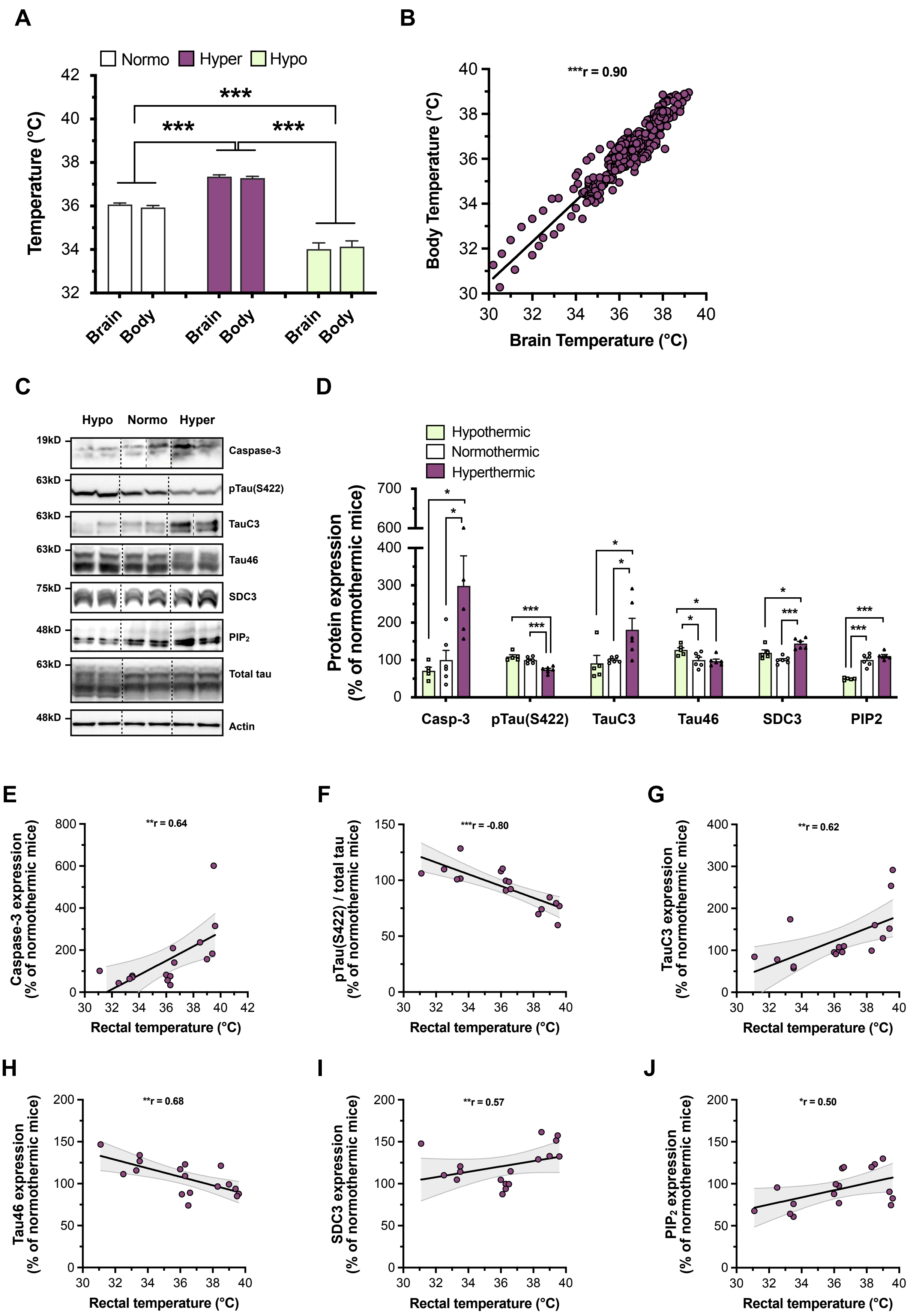
A
Normo
Hyper
Hypo
Temperature (°C)
Brain
Body
***
B
***r = 0.90
Body Temperature (°C)
Brain Temperature (°C)
C
Hypo
Normo
Hyper
19kD
Caspase-3
63kD
pTau(S422)
TauC3
Tau46
75kD
SDC3
48kD
PIP2
Total tau
Actin
D
Hypothermic
Normothermic
Hyperthermic
Protein expression
(% of normothermic mice)
Casp-3
pTau(S422)
TauC3
Tau46
SDC3
PIP2
E
**r = 0.64
Caspase-3 expression
(% of normothermic mice)
Rectal temperature (°C)
F
***r = -0.80
pTau(S422) / total tau
(% of normothermic mice)
Rectal temperature (°C)
G
**r = 0.62
TauC3 expression
(% of normothermic mice)
Rectal temperature (°C)
H
**r = 0.68
Tau46 expression
(% of normothermic mice)
Rectal temperature (°C)
I
**r = 0.57
SDC3 expression
(% of normothermic mice)
Rectal temperature (°C)
J
*r = 0.50
PIP2 expression
(% of normothermic mice)
Rectal temperature (°C)

Supplemental Figure 6. UPS-I protein component expressions correlate with body temperature in hTau mice.

(A) Simultaneous recording of core body temperature and brain temperature in wild-type mice exposed to 4°C (hypothermic) or 38°C (hyperthermic) compared to normothermic mice S422 (*n* = 3; Tukey's; mean ± SEM). (**B**) Core body temperature and brain temperature are significantly correlated (Pearson correlation). **(C, D)** The expressions of caspase-3, TauC3, SDC3 and PIP2 are increased in the cortices of hyperthermic mice compared to hypo- or normothermic mice, while tau phosphorylation is decreased at S422 (*n* = 5-6; Tukey's; mean ± SEM). **(E-J)** The cortical expression of caspase-3, pTau(S422), TauC3, Tau46, SDC3 and PIP_2 are significantly correlated with rectal temperature of mice (Pearson correlation; standard error bars displayed as error envelopes in light grey). *p<0.05, **p<0.01 and ***p<0.001.

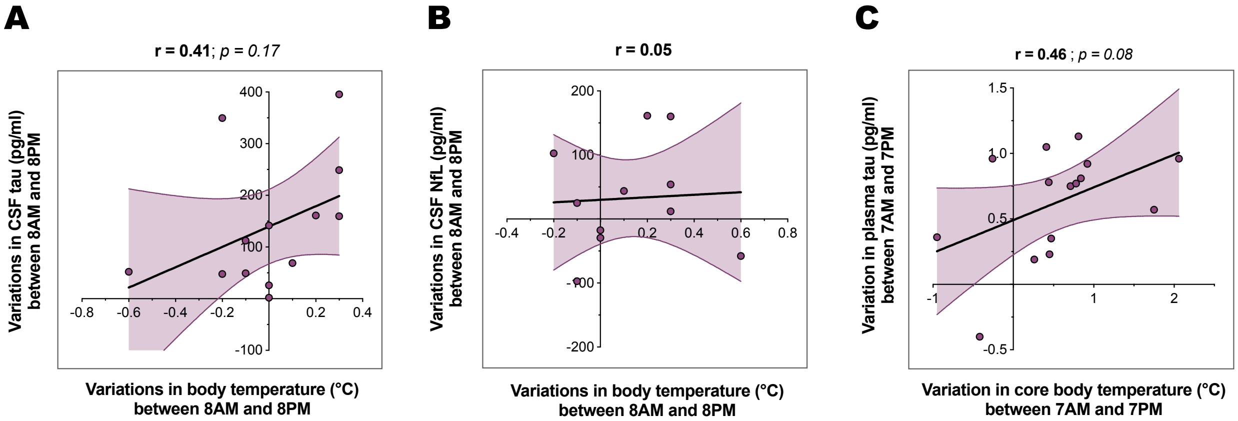

Supplemental Figure 7. Correlation between variations in body temperature and CSF/plasma tau levels in humans.

(A) The variation in total CSF tau concentrations between 8AM and 8PM is not significantly correlated to the variation in body temperature at the same times (n = 13, Pearson's correlation, p=0.17). **(B)** No correlation is observed for CSF NfL concentrations and body temperature (n = 11, Pearson's correlation). **(C)** The variation in total plasma tau concentrations between 7AM and 7PM is not significantly correlated to the variation in core body temperature between 7PM and 7AM (n = 15, Pearson's correlation, p=0.08). Standard error bars displayed as error envelopes in light purple.

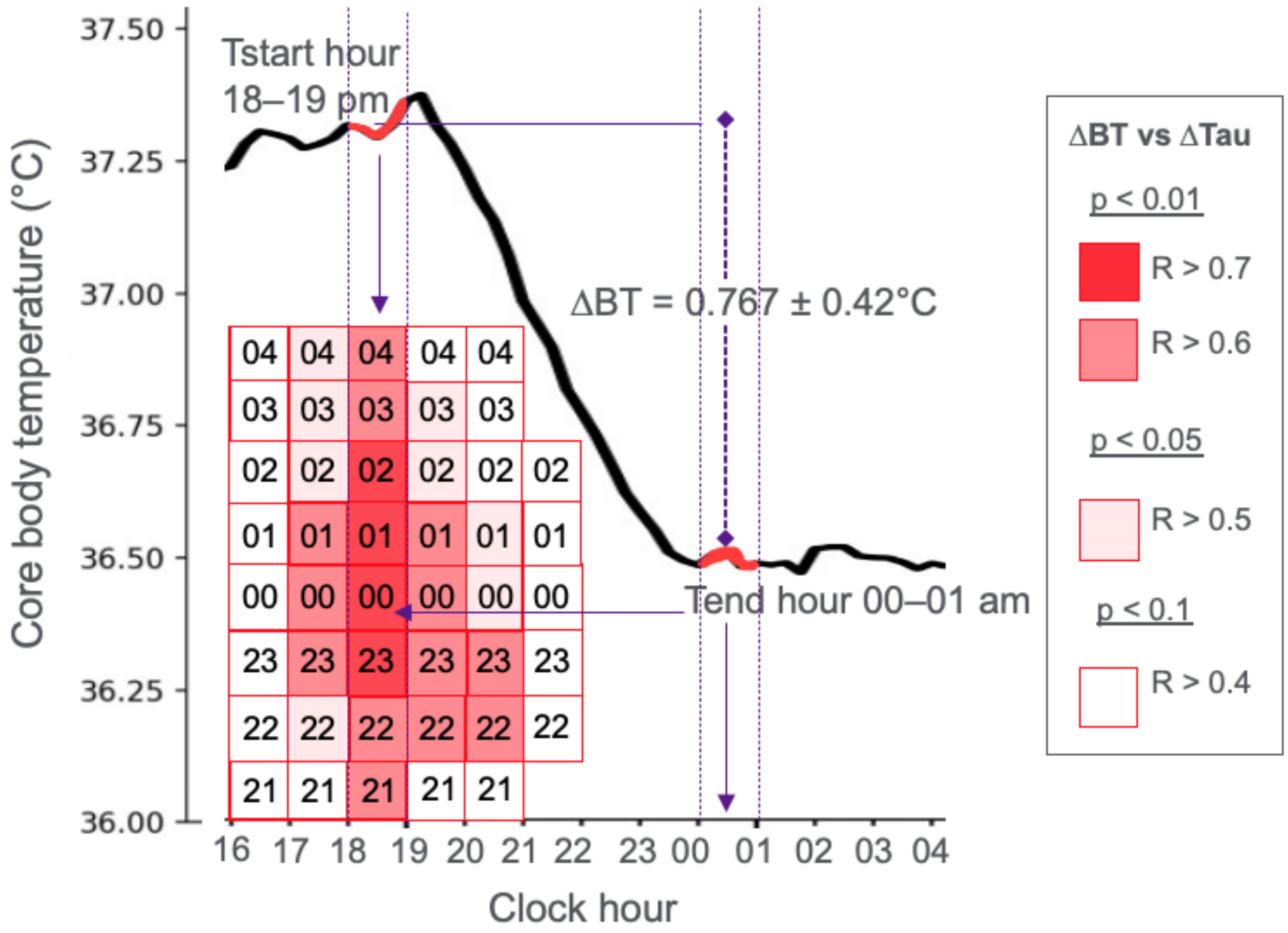

Supplemental Figure 8. ΔTau-ΔBT correlations by ΔBT clock interval.
ΔTau vs ΔBT correlation grid superimposed upon group average BT per clock hour. Colors show strength of correlation with ΔTau for each ΔBT value. ΔBT is specified by Tstart hour (x axis) and Tend hour (value in grid). Max R value (R=0.721, $p < 0.001$) was at ΔBT Tstart 18-19 pm, Tend 00-01 pm (shown). Strong correlations (R > 0.6) were seen for multiple adjacent intervals.

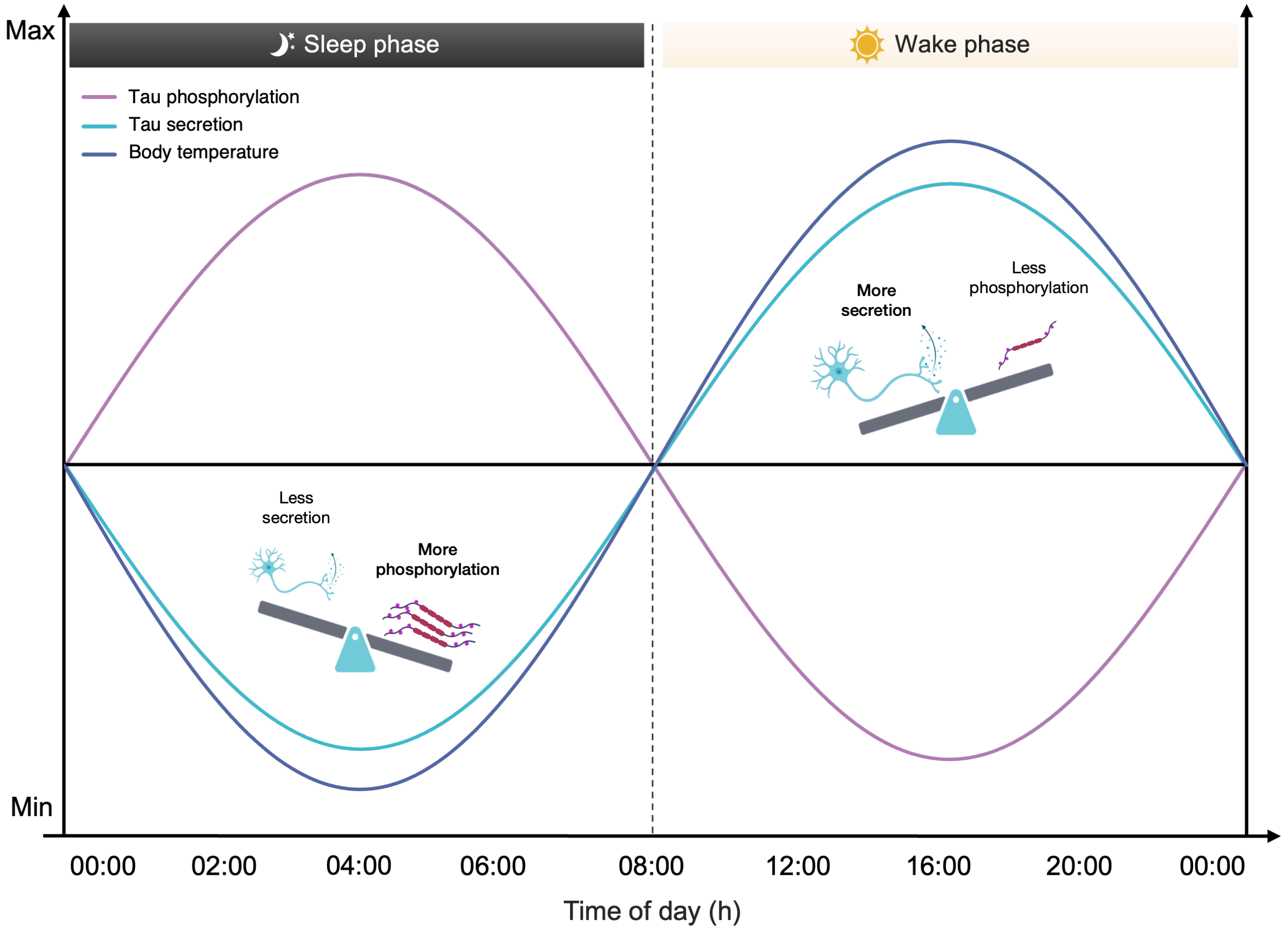

Supplemental Figure 9.

Integrative graphical representation of tau phosphorylation and secretion synchronized with the physiological changes in core BT during the sleep-wake cycle. The periods of increased tau phosphorylation and reduced secretion during the sleep phase, contrasted with heightened secretion and decreased phosphorylation during the wake phase, are represented. This is a simplified sinusoidal representation of the variations of the three parameters; actual measurements are likely to be more complex. Time of day is indicated on the x-axis, with the y-axis reflecting the relative levels of each process from minimum to maximum.

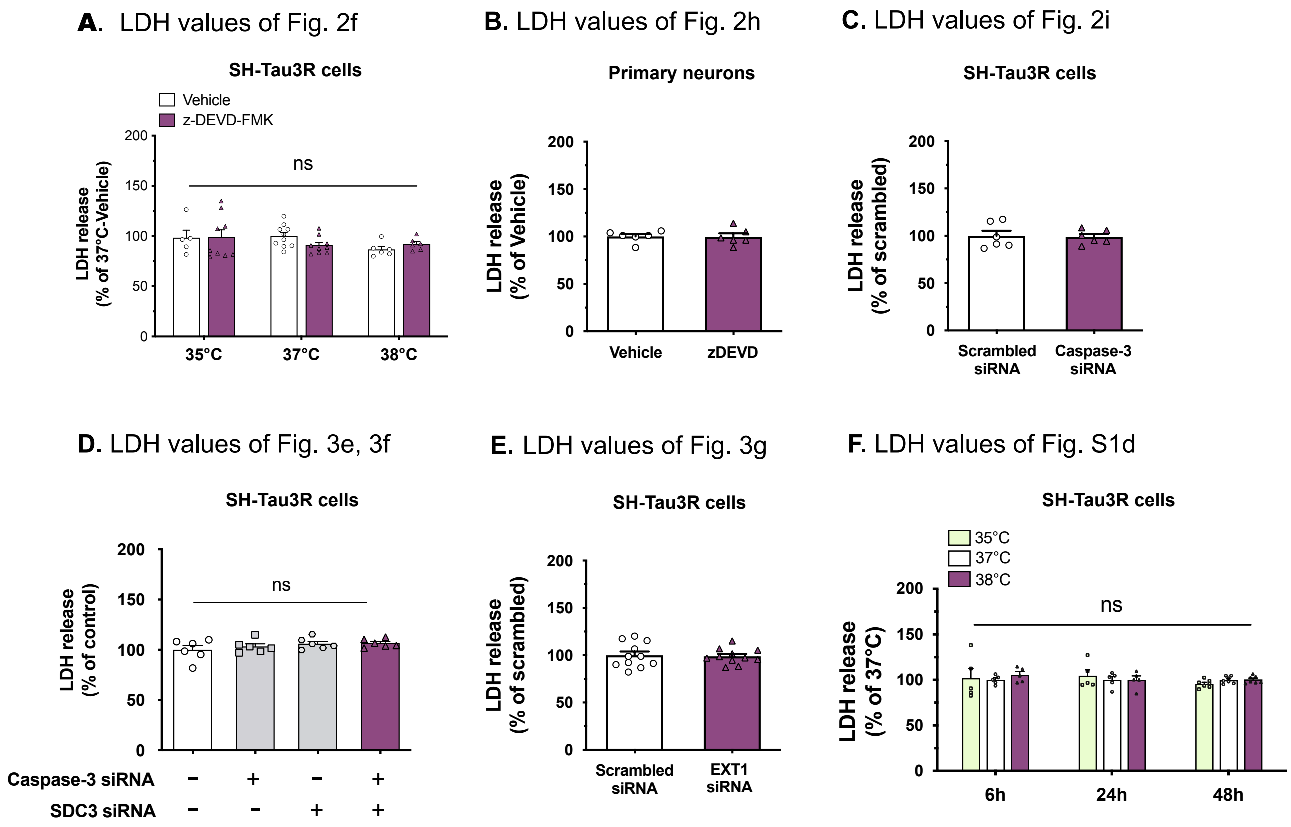

Supplemental Figure 10. Respective LDH values obtained for each in vitro experiment.
(**A-F**) The corresponding experiences are mentioned above each graph. ns: non-significant

Supplemental Table 1:
Antibodies used in Western blot, Dot blot, co-immunoprecipitation and immunocytochemistry experiments

Protein	Dilution (WB/DB)	Dilution (ICC)	Dilution (co-IP)	Ref.	Supplier
**Mouse primary antibodies**					
AT100 (pThr212/pSer214)	1/1000	/		MN1060	ThermoFisher
AT270 (pThr181)	1/1000	/		MN1050	ThermoFisher
CP13 (pSer202)	1/1000	/		-	Peter Davies
DA9 (amino acids 102-140)	1/1000	/	1/100	-	Peter Davies
MAP2	1/1000	/		MAB3418	Millipore
MC6 (pSer235)	1/1000	/		-	Peter Davies
PHF1 (pSer396/pSer404)	1/1000	/		-	Peter Davies
PIP2 (2C11)	1/1000	/	4 µg	Sc-53412	Santa Cruz
Tau 3-repeat isoform (RD3)	1/1000	/		05-803	Millipore
Tau12 (amino acids 9-18)	1/1000	/		ab74137	abcam
Tau46 (amino acids 404-441)	1/1000	/	1/100	ab203179	abcam
TauC3 (cleaved Asp421)	1/1000	1/100	3 µg	AHB0061	ThermoFisher
β-actin	1/10000	/		A2628	Sigma-Aldrich
**Rabbit primary antibodies**					
Caspase-1	1/1000	/		3866S	Cell Signaling
Caspase-3	1/1000	/		9662S	Cell Signaling
FGF2	1/1000	/		ab208687	abcam
Neurofilament-L (NfL)	1/1000	/		2837S	Cell Signaling
pSer199	1/1000	/		44734G	ThermoFisher
pSer422	1/1000	/		44774G	ThermoFisher
pThr205	1/1000	/		44738G	ThermoFisher
Syndecan-3 (HSPG)	1/1000	1/100		10886-1-AP	ThermoFisher
TauC (amino acids 243-441)	1/10000	/		A0024	Dako Cytomation
α-synuclein	1/1000	/		2642S	Cell Signaling
**Secondary antibodies**					
HRP conjugate goat anti-mouse IgG (HC+LC)	1/5000	/		115-035-003	Jackson laboratories
HRP conjugate goat anti-mouse IgG (LC specific)	1/5000	/		AP200P	Millipore
HRP conjugate goat anti-rabbit IgG (HC+LC)	1/5000	/		111-035-144	Jackson laboratories
Alexa Fluor 488 goat anti-mouse IgG (HC+LC)	/	1/1000		A-11029	ThermoFisher
Alexa Fluor 633 goat anti-rabbit IgG (HC+LC)	/	1/1000		A-11036	ThermoFisher

List of primers used for RT-qPCR

Gene Name	Gene code	Species	Forward primer (5' - 3')	Reverse primer (5' - 3')	Product length	Exon Location
Caspase-3 (CASP3)	NM_004346	Human	CACGGATACACAGCCACAG	CGGATGGGTGCTATTGTGAG	39	1-2
Syndecan-3 (SDC3)	NM_014654	Human	GTAGCTGCCCTCATCCTTT	CTGCCTCAGAAGAGTATCCTG	40	4-5
Exostosin-1 (EXT1)	NM_000127	Human	CAGGAATCTGAAGGACCCAAG	TGACAGAGACAACACCGAGTA	42	1-2

<u>Supplemental Table 2:</u> Individual data of oral temperature, CSF tau levels and CSF NfL levels

Fig.6a	Oral temperature (°C) at 8AM	Oral temperature (°C) at 4PM	Δ temperature (°C)	CSF tau (pg/ml) at 8AM	CSF tau (pg/ml) at 4PM	Δ CSF tau (pg/ml)
Participant 1	36,1	36,3	0,2	1516,759	1731,063	214,304
Participant 2	36,1	36,1	0,0	709,693	844,842	135,149
Participant 3	36,2	36,6	0,4	1129,545	1368,339	238,794
Participant 4	36,8	37,0	0,2	1659,087	1806,234	147,147
Participant 5	36,5	36,9	0,4	724,887	983,029	258,142
Participant 6	36,5	36,6	0,1	1560,753	1539,916	-20,836
Participant 7	37,0	36,9	-0,1	981,481	1147,518	166,037
Participant 8	36,8	36,8	0,0	1189,658	1181,240	-8,418
Participant 9	36,6	36,4	-0,2	699,585	712,338	12,752
Participant 10	36,2	36,4	0,2	1060,842	1157,617	96,775
Participant 11	36,8	36,8	0,0	393,811	427,549	33,738
Participant 12	37,1	36,6	-0,5	694,996	757,565	62,569
Participant 13	37,0	37,0	0,0	1049,208	1190,000	140,792

Fig.6b	Oral temperature (°C) at 8AM	Oral temperature (°C) at 4PM	Δ temperature (°C)	CSF NfL (pg/ml) at 8AM	CSF NfL (pg/ml) at 4PM	Δ CSF NfL (pg/ml)
Participant 1	36,1	36,3	0,2	2256,25	2207,596	-48,7
Participant 2	36,1	36,1	0,0	1000,488	951,94	-48,5
Participant 3	36,2	36,6	0,4	732,174	816,85	84,7
Participant 4	36,2	36,7	0,5	861,198	849,644	-11,6
Participant 5	36,5	36,9	0,4	907,118	971,764	64,6
Participant 6	36,5	36,6	0,1	1327,746	1298,478	-29,3
Participant 7	37	36,9	-0,1	1317,66	1370,886	53,2
Participant 8	36,8	36,8	0,0	1056,076624	1104,25169	48,2
Participant 9	36,6	36,4	-0,2	1347,956	1347,956	0,0
Participant 10	36,2	36,4	0,2	1076,154	1169,258	93,1
Participant 11	36,8	36,8	0,0	756,852	949,632	192,8

Paired samples statistics

	N	Mean	SD	SEM
CSF tau 8AM	13	1028	384.6	106.7
CSF tau 4PM	13	1142	404	112

Mean of differences (4PM - 8AM)	SD of differences	SEM of differences	95% confidence interval	R squared (partial eta squared)	Correlation coefficient (r)	P value (one tailed)	P value summary
113.6	93.18	25.84	57.30 to 169.9	0.6169	0.9733	<0.0001	****

<u>Supplemental Table 3:</u> Pairwise comparisons and individual data of core body temperature and plasma tau levels

Fig.6c

Timepoint	n	Mean ± SD	Pairwise comparison, n	Wilcoxon related samples signed rank test
Day 1 7 am	19	4.22 ± 1.0	Day 1 7 am vs 7 pm, n=19	p = 0.006
Day 1 7 pm	19	4.81 ± 1.4		
Day 1 7 am	16	4.10 ± 1.0	Day 2 7 am vs 7 pm, n=16	p = 0.011
Day 1 7 pm	16	4.68 ± 1.4		

Subject ID	Age	Gender	Δ temperature 6PM minus 1AM (°C)	Δ plasma tau 7PM minus 7 AM (pg/ml)
Participant 1	71	M	0.6	0.3
Participant 2	74	M	1.0	0.8
Participant 7	74	M	1.2	0.9
Participant 9	77	F	1.2	2.1
Participant 11	60	F	-0.1	-1.0
Participant 14	68	M	0.1	0.7
Participant 27	75	F	0.6	-0.3
Participant 28	65	F	1.3	1.8
Participant 51	66	F	1.2	0.8
Participant 64	75	M	0.7	0.4
Participant 68	65	F	0.1	0.5
Participant 74	66	M	0.72	0.47
Participant 76	63	M	-0.23	-0.42
Participant 83	66	F	1.25	0.44
Participant 89	62	F	1.35	0.78

Supplemental Table 4: Detailed statistical information

Fig. 1	Description	Normality test	Statistical analysis	Post-test
Fig. 1C	ELISA - 72 hours (35-38°C) SH-Tau3R	Kologorov-Smirnov	One-way ANOVA $F_{2,15}$ = 190.4 (p< 0.0001)	Tukey's Multiple Comparison Test
Fig. 1E	Dot blot Tau3R SH-Tau3R	Kologorov-Smirnov	One-way ANOVA $F_{2,31}$ = 24.25 (p< 0.0001)	Tukey's Multiple Comparison Test
	Dot blot TauC SH-Tau3R	Kologorov-Smirnov	One-way ANOVA $F_{2,31}$ = 12.68 (p< 0.0001)	Tukey's Multiple Comparison Test
	Dot blot DA9 SH-Tau3R	Kologorov-Smirnov	One-way ANOVA $F_{2,33}$ = 6.67 (p< 0.01)	Tukey's Multiple Comparison Test
	Dot blot Tau12 SH-Tau3R	Kologorov-Smirnov	One-way ANOVA $F_{2,31}$ = 9.085 (p< 0.001)	Tukey's Multiple Comparison Test
Fig. 1G	Dot blot AT270 SH-Tau3R	Kologorov-Smirnov	One-way ANOVA $F_{2,30}$ = 14.08 (p< 0.0001)	Tukey's Multiple Comparison Test
	Dot blot pS199 SH-Tau3R	Kologorov-Smirnov	One-way ANOVA $F_{2,30}$ = 11.92 (p< 0.001)	Tukey's Multiple Comparison Test
	Dot blot CP13 SH-Tau3R	Kologorov-Smirnov	One-way ANOVA $F_{2,32}$ = 27.66 (p< 0.0001)	Tukey's Multiple Comparison Test
	Dot blot pT205 SH-Tau3R	Kologorov-Smirnov	One-way ANOVA $F_{2,36}$ = 39.53 (p< 0.0001)	Tukey's Multiple Comparison Test
	Dot blot AT100 SH-Tau3R	Kologorov-Smirnov	One-way ANOVA $F_{2,27}$ = 6.916 (p< 0.01)	Tukey's Multiple Comparison Test
	Dot blot MC6 SH-Tau3R	Kologorov-Smirnov	One-way ANOVA $F_{2,22}$ = 79.55 (p< 0.0001)	Tukey's Multiple Comparison Test
	Dot blot PHF1 SH-Tau3R	Kologorov-Smirnov	One-way ANOVA $F_{2,31}$ = 114.5 (p< 0.0001)	Tukey's Multiple Comparison Test
Fig. 1I	ELISA assays of mouse tau Primary neurons	Kologorov-Smirnov	One-way ANOVA $F_{2,15}$ = 21.84 (p< 0.0001)	Dunnett's Multiple Comparison Test
Fig. 1K	Dot blot of total tau Primary neurons	Kologorov-Smirnov	One-way ANOVA $F_{2,32}$ = 11.38 (p< 0.001)	Tukey's Multiple Comparison Test
	Dot blot of pS199 tau Primary neurons	Kologorov-Smirnov	One-way ANOVA $F_{2,32}$ = 13.70 (p< 0.0001)	Tukey's Multiple Comparison Test

	Description	Normality test	Statistical analysis	Post-test
	Dot blot of pT205 tau<br>Primary neurons	Kologorov-Smirnov	One-way ANOVA<br>$F_{2,32}$ = 39.36 (p< 0.0001)	Tukey's Multiple Comparison Test

Fig. 2	Description	Normality test	Statistical analysis	Post-test
**Fig. 2A**	Caspase-3 activity assay<br>SH-Tau3R	Kologorov-Smirnov	One-way ANOVA<br>$F_{2,15}$ = 11.53 (p< 0.001)	Tukey's Multiple Comparison Test
**Fig. 2C**	Western blot Caspase-3<br>SH-Tau3R	Kologorov-Smirnov	One-way ANOVA<br>$F_{2,12}$ = 24.47 (p< 0.0001)	Tukey's Multiple Comparison Test
	Western blot pTau(S422)<br>SH-Tau3R	Kologorov-Smirnov	One-way ANOVA<br>$F_{2,14}$ = 30.94 (p< 0.0001)	Tukey's Multiple Comparison Test
	Western blot TauC3<br>SH-Tau3R	Kologorov-Smirnov	One-way ANOVA<br>$F_{2,13}$ = 116.2 (p< 0.0001)	Tukey's Multiple Comparison Test
	Western blot Tau46<br>SH-Tau3R	Kologorov-Smirnov	One-way ANOVA<br>$F_{2,14}$ = 21.87 (p< 0.0001)	Tukey's Multiple Comparison Test
	Western blot Caspase-3<br>Primary neurons	Kologorov-Smirnov	One-way ANOVA<br>$F_{2,15}$ = 4.113 (p< 0.05)	Tukey's Multiple Comparison Test
	Western blot pTau(S422)<br>Primary neurons	Kologorov-Smirnov	One-way ANOVA<br>$F_{2,15}$ = 9.424 (p< 0.01)	Tukey's Multiple Comparison Test
	Western blot TauC3<br>Primary neurons	Kologorov-Smirnov	One-way ANOVA<br>$F_{2,15}$ = 5.62 (p< 0.05)	Tukey's Multiple Comparison Test
	Western blot Tau46<br>Primary neurons	Kologorov-Smirnov	One-way ANOVA<br>$F_{2,15}$ = 2.931 (ns)	Tukey's Multiple Comparison Test
**Fig. 2E**	Dot blot TauC3<br>SH-Tau3R	Kologorov-Smirnov	One-way ANOVA<br>$F_{2,30}$ = 14.89 (p< 0.0001)	Tukey's Multiple Comparison Test
	Dot blot Tau46<br>SH-Tau3R	Kologorov-Smirnov	One-way ANOVA<br>$F_{2,32}$ = 31.49 (p< 0.0001)	Tukey's Multiple Comparison Test
	Dot blot TauC3<br>Primary neurons	Kologorov-Smirnov	One-way ANOVA<br>$F_{2,30}$ = 26.00 (p< 0.0001)	Tukey's Multiple Comparison Test
	Dot blot Tau46<br>Primary neurons	Kologorov-Smirnov	One-way ANOVA<br>$F_{2,15}$ = 24.25 (p< 0.0001)	Tukey's Multiple Comparison Test
**Fig. 2F**	35°C - Dot blot total tau<br>(zDEVD)	Kologorov-Smirnov	Unpaired t test with Welch's correction	N/A

	SH-Tau3R		two-tailed P value<br>p=0.4750, non-significant (ns)	
	37°C - Dot blot total tau (zDEVD)<br>SH-Tau3R	Kologorov-Smirnov	Unpaired t test with Welch's correction<br>two-tailed P value<br>*** p< 0.0001	N/A
	38°C - Dot blot total tau (zDEVD)<br>SH-Tau3R	Kologorov-Smirnov	Unpaired t test with Welch's correction<br>two-tailed P value<br>*** p< 0.001	N/A
	35°C - Dot blot TauC3 (zDEVD)<br>SH-Tau3R	N/A	Unpaired t test with Welch's correction<br>two-tailed P value<br>** p< 0.01	N/A
	37°C - Dot blot TauC3 (zDEVD)<br>SH-Tau3R	Kologorov-Smirnov	Unpaired t test with Welch's correction<br>two-tailed P value<br>** p< 0.01	N/A
	38°C - Dot blot TauC3 (zDEVD)<br>SH-Tau3R	Kologorov-Smirnov	Unpaired t test with Welch's correction<br>two-tailed P value<br>** p< 0.01	N/A
**Fig. 2G**	ELISA assays of mouse tau (zDEVD)<br>Primary neurons	Kologorov-Smirnov	Unpaired t test with Welch's correction<br>two-tailed P value<br>* p< 0.05	N/A
**Fig. 2H**	Dot blot total tau (z-DEVD)<br>Primary neurons	Kologorov-Smirnov	Unpaired t test with Welch's correction<br>two-tailed P value<br>* p< 0.05	N/A
	Dot blot TauC3 (z-DEVD)<br>Primary neurons	Kologorov-Smirnov	Unpaired t test with Welch's correction<br>two-tailed P value<br>* p< 0.05	N/A
**Fig. 2I**	Dot blot total tau (siRNA Caspase-3)<br>SH-Tau3R	Kologorov-Smirnov	Unpaired t test with Welch's correction<br>two-tailed P value<br>** p< 0.01	N/A
	Dot blot TauC3 (siRNA Caspase-3)	Kologorov-Smirnov	Unpaired t test with Welch's correction	N/A

	SH-Tau3R		two-tailed P value<br>** p< 0.01	

Fig. 3	Description	Normality test	Statistical analysis	Post-test
**Fig. 3B**	Western blot SDC3<br>SH-Tau3R cells	Kologorov-Smirnov	One-way ANOVA<br>$F_{2,13}$ = 20.42 (p< 0.0001)	Tukey's Multiple Comparison Test
	Western blot PIP2<br>SH-Tau3R cells	Kologorov-Smirnov	One-way ANOVA<br>$F_{2,15}$ = 25.16 (p< 0.0001)	Tukey's Multiple Comparison Test
	Western blot SDC3<br>Primary neurons	Kologorov-Smirnov	One-way ANOVA<br>$F_{2,13}$ = 13.56 (p< 0.001)	Tukey's Multiple Comparison Test
	Western blot PIP2<br>Primary neurons	Kologorov-Smirnov	One-way ANOVA<br>$F_{2,15}$ = 10.04 (p< 0.01)	Tukey's Multiple Comparison Test

Fig. 4	Description	Normality test	Statistical analysis	Post-test
**Fig. 4A**	Dot blot Total tau (siRNAs)<br>SH-Tau3R cells	Kologorov-Smirnov	One-way ANOVA<br>$F_{2,20}$ = 17.29 (p< 0.0001)	Tukey's Multiple Comparison Test
**Fig. 4B**	Dot blot TauC3 (siRNAs)<br>SH-Tau3R cells	Kologorov-Smirnov	One-way ANOVA<br>$F_{2,19}$ = 12.88 (p< 0.0001)	Tukey's Multiple Comparison Test
**Fig. 4C**	Dot blot total tau (siRNA EXT1)<br>SH-Tau3R cells	Kologorov-Smirnov	Unpaired t test with Welch's correction<br>two-tailed P value<br>**** p< 0.0001	N/A
	Dot blot TauC3 (siRNA EXT1)<br>SH-Tau3R cells	Kologorov-Smirnov	Unpaired t test with Welch's correction<br>two-tailed P value<br>** p< 0.01	N/A
**Fig. 4E**	Membrane fluidity assay<br>SH-Tau3R cells	Kologorov-Smirnov	One-way ANOVA<br>$F_{2,21}$ = 22.98 (p< 0.0001)	Tukey's Multiple Comparison Test
**Fig. 4I**	Western blot Tau3R (PIP2 binding)<br>SH-Tau3R cells	N/A	Kruskal-Wallis test (ANOVA)<br>ns, p= 0.7910	Dunn's Multiple Comparison Test

**Fig. 4J**	Western blot TauC3 (PIP2 binding)<br>SH-Tau3R cells	N/A	Kruskal-Wallis test (ANOVA)<br>** p< 0.01	Dunn's Multiple Comparison Test

**Fig.5**	**Description**	**Normality test**	**Statistical analysis**	**Post-test**
**Fig. 5A**	Rectal temperature (°C)<br>Wild-type mice<br>Awake vs. Sleep	Kologorov-Smirnov	Unpaired t test with Welch's correction<br>two-tailed P value, n=6 per group<br>** p< 0.01	N/A
**Fig. 5D**	WB Caspase-3<br>Wild-type mice<br>Awake vs sleep	Kologorov-Smirnov	Unpaired t test with Welch's correction<br>two-tailed P value, n=10 per group<br>** p< 0.01	N/A
	WB pTau(S422)<br>Wild-type mice<br>Awake vs sleep	Kologorov-Smirnov	Unpaired t test with Welch's correction<br>two-tailed P value, n=10 per group<br>** p< 0.01	N/A
	WB TauC3<br>Wild-type mice<br>Awake vs sleep	Kologorov-Smirnov	Unpaired t test with Welch's correction<br>two-tailed P value, n=10 per group<br>* p< 0.05	N/A
	WB Tau46<br>Wild-type mice<br>Awake vs sleep	Kologorov-Smirnov	Unpaired t test with Welch's correction<br>two-tailed P value, n=10 per group<br>p = 0.3166, ns	N/A
	WB SDC3<br>Wild-type mice<br>Awake vs sleep	Kologorov-Smirnov	Unpaired t test with Welch's correction<br>two-tailed P value, n=10 per group<br>** p< 0.01	N/A
	WB PIP2<br>Wild-type mice<br>Awake vs sleep	Kologorov-Smirnov	Unpaired t test with Welch's correction<br>two-tailed P value, n=10 per group<br>* p< 0.05	N/A
**Fig. 5F**	Body temperature (°C)<br>Sleep deprived mice	Kologorov-Smirnov	One-way ANOVA<br>$F_{5,24}$ = 13.59 (p< 0.0001)	Tukey's Multiple Comparison Test
**Fig. 5H**	WB Caspase-3<br>Sleep deprived mice	Kologorov-Smirnov	Unpaired t test with Welch's correction<br>two-tailed P value<br>* p< 0.05	N/A
	WB pTau(S422)	Kologorov-Smirnov	Unpaired t test with Welch's correction	N/A

	Sleep deprived mice		two-tailed P value<br>** $p< 0.01$	
	WB TauC3<br>Sleep deprived mice	Kologorov-Smirnov	Unpaired t test with Welch's correction<br>two-tailed P value<br>* $p< 0.05$	N/A
	WB Tau46<br>Sleep deprived mice	Kologorov-Smirnov	Unpaired t test with Welch's correction<br>two-tailed P value<br>* $p< 0.05$	N/A
	WB SDC3<br>Sleep deprived mice	Kologorov-Smirnov	Unpaired t test with Welch's correction<br>two-tailed P value<br>$p = 0.4618$, ns	N/A
	WB PIP2<br>Sleep deprived mice	Kologorov-Smirnov	Unpaired t test with Welch's correction<br>two-tailed P value<br>* $p< 0.05$	N/A
**Fig. 5J**	Rectal temperature (°C)<br>hTau mice<br>Hypo/Normo/hyperthermic	Shapiro-Wilk	Two-way ANOVA<br>$F_{2,14} = 75.21$ for group, **** $p< 0.0001$<br>$F_{1,14} = 18.60$ for time, *** $p< 0.001$<br>$F_{2,14} = 116.9$ for interaction, **** $p< 0.0001$	Šĺdák's Multiple Comparison Test
**Fig. 5K**	CSF tau (pg/ml) - ELISA<br>hTau mice<br>Hypo/Normo/hyperthermic	Shapiro-Wilk	One-Way ANOVA<br>$F_{2,11} = 5.238$ ($p< 0.05$)	Dunnet's Multiple Comparison Test
**Fig. 5K**	Correlation<br>CSF tau & rectal temperature	N/A	Correlation with Pearson coefficient (r=0.6498)<br>* $p< 0.05$	N/A
**Fig. 5L**	Plasma tau (pg/ml) - ELISA<br>hTau mice<br>Hypo/Normo/hyperthermic	Shapiro-Wilk	One-Way ANOVA<br>$F_{2,12} = 3.314$ (ns)	Dunnet's Multiple Comparison Test
**Fig. 5L**	Correlation<br>Plasma tau & rectal temperature	N/A	Correlation with Pearson coefficient (r=0.6032)<br>* $p< 0.05$	N/A

Fig. 6	Description	Normality test	Statistical analysis	Post-test
**Fig. 6A**	Correlation<br>CSF tau & oral temperature (8AM-4PM)	Kologorov-Smirnov	Correlation with Pearson coefficient (r=0.5810)<br>* $p < 0.05$	N/A
**Fig. 6B**	Correlation<br>CSF NfL & oral temperature (8AM-4PM)	Kologorov-Smirnov	Correlation with Pearson coefficient (r=0.0012)<br>ns	N/A
**Fig. 6C**	Correlation<br>plasma tau (7AM-7PM) & body temperature (6PM-1AM)	Kologorov-Smirnov	Correlation with Pearson coefficient (r=0.7145)<br>** $p < 0.01$	N/A

Supp. Fig. 1	Description	Normality test	Statistical analysis	Post-test
**Supp. Fig. 1A**	ELISA assays of total tau<br>SH-Tau3R	Kologorov-Smirnov	One-way ANOVA<br>$F_{2,15} = 16.59$ ($p < 0.001$)	Dunnett's Multiple Comparison Test
**Supp. Fig. 1B**	ELISA assays of pS199 tau<br>SH-Tau3R	Kologorov-Smirnov	One-way ANOVA<br>$F_{2,15} = 8.47$ ($p < 0.01$)	Tukey's Multiple Comparison Test
	ELISA assays of pT231 tau<br>SH-Tau3R	Kologorov-Smirnov	One-way ANOVA<br>$F_{2,15} = 13.22$ ($p < 0.001$)	Tukey's Multiple Comparison Test
	ELISA assays of pS396 tau<br>SH-Tau3R	Kologorov-Smirnov	One-way ANOVA<br>$F_{2,15} = 7.21$ ($p < 0.01$)	Tukey's Multiple Comparison Test
**Supp. Fig. 1C**	LDH - 72 hours (35-39°C)<br>SH-Tau3R	Kologorov-Smirnov	One-way ANOVA<br>$F_{3,28} = 94.16$ ($p < 0.0001$)	Tukey's Multiple Comparison Test
**Supp. Fig. 1D**	LDH - 72 hours (35-38°C)<br>Mouse primary neurons	Kologorov-Smirnov	One-way ANOVA<br>$F_{2,33} = 0.7683$ (ns)	Tukey's Multiple Comparison Test
**Supp. Fig. 1E**	Dot blot total tau - 6 hours of exposure	N/A	Kruskal-Wallis test (ANOVA)	Dunn's Multiple Comparison Test

	SH-Tau3R		** p< 0.01	
	Dot blot total tau - 24 hours of exposure<br>SH-Tau3R	Kologorov-Smirnov	One-way ANOVA<br>$F_{2,12} = 45.92$ ($p < 0.0001$)	Tukey's Multiple Comparison Test
	Dot blot total tau - 48 hours of exposure<br>SH-Tau3R	Kologorov-Smirnov	One-way ANOVA<br>$F_{2,18} = 13.59$ ($p < 0.001$)	Tukey's Multiple Comparison Test

**Supp. Fig. 1G**	WB total tau<br>SH-Tau3R	Kologorov-Smirnov	One-way ANOVA<br>$F_{2,12} = 2.774$ (ns)	Tukey's Multiple Comparison Test
	WB AT270<br>SH-Tau3R	Kologorov-Smirnov	One-way ANOVA<br>$F_{2,12} = 95.87$ ($p < 0.0001$)	Tukey's Multiple Comparison Test
	WB pS199<br>SH-Tau3R	Kologorov-Smirnov	One-way ANOVA<br>$F_{2,12} = 20.08$ ($p < 0.001$)	Tukey's Multiple Comparison Test
	WB CP13<br>SH-Tau3R	Kologorov-Smirnov	One-way ANOVA<br>$F_{2,12} = 90.31$ ($p < 0.0001$)	Tukey's Multiple Comparison Test
	WB pT205<br>SH-Tau3R	Kologorov-Smirnov	One-way ANOVA<br>$F_{2,12} = 25.35$ ($p < 0.0001$)	Tukey's Multiple Comparison Test
	WB AT100<br>SH-Tau3R	Kologorov-Smirnov	One-way ANOVA<br>$F_{2,12} = 21.06$ ($p < 0.0001$)	Tukey's Multiple Comparison Test
	WB MC6<br>SH-Tau3R	Kologorov-Smirnov	One-way ANOVA<br>$F_{2,12} = 204.6$ ($p < 0.0001$)	Tukey's Multiple Comparison Test
	WB PHF1<br>SH-Tau3R	Kologorov-Smirnov	One-way ANOVA<br>$F_{2,12} = 96.89$ ($p < 0.0001$)	Tukey's Multiple Comparison Test

**Supp. Fig. 1I**	Dot blot MAP2<br>SH-Tau3R	Kologorov-Smirnov	One-way ANOVA<br>$F_{2,12} = 0.1933$ (ns)	Dunnet's Multiple Comparison Test
	Dot blot alpha-synuclein<br>SH-Tau3R	Kologorov-Smirnov	One-way ANOVA<br>$F_{2,15} = 0.272$ (ns)	Dunnet's Multiple Comparison Test
	Dot blot FGF2<br>SH-Tau3R	Kologorov-Smirnov	One-way ANOVA<br>$F_{2,15} = 0.6294$ (ns)	Dunnet's Multiple Comparison Test
	Dot blot Caspase-1<br>SH-Tau3R	Kologorov-Smirnov	One-way ANOVA<br>$F_{2,13} = 1.539$ (ns)	Dunnet's Multiple Comparison Test
	Dot blot NfL<br>SH-Tau3R	Kologorov-Smirnov	One-way ANOVA<br>$F_{2,13} = 1.512$ (ns)	Dunnet's Multiple Comparison Test

**Supp. Fig. 1J**	WB FGF2<br>SH-Tau3R	Kologorov-Smirnov	One-way ANOVA<br>$F_{2,15} = 0.4731$ (ns)	Dunnet's Multiple Comparison Test

**Supp Fig. 3**	**Description**	**Normality test**	**Statistical analysis**	**Post-test**
**Supp Fig. 3**	qPCR Caspase-3<br>SH-Tau3R cells	Kologorov-Smirnov	One-way ANOVA<br>$F_{2,12} = 1.53$ (ns)	Tukey's Multiple Comparison Test
	qPCR Syndecan-3<br>SH-Tau3R cells	Kologorov-Smirnov	One-way ANOVA<br>$F_{2,12} = 7.51$ ($p < 0.01$)	Tukey's Multiple Comparison Test
	qPCR EXT1<br>SH-Tau3R cells	Kologorov-Smirnov	One-way ANOVA<br>$F_{2,12} = 6.057$ ($p < 0.05$)	Tukey's Multiple Comparison Test

**Supp. Fig. 5**	**Description**	**Normality test**	**Statistical analysis**	**Post-test**
**Supp. Fig. 5A**	Correlation<br>Caspase-3 and rectal temperature	N/A	Correlation with Pearson coefficient (r=0.6386)<br>** p< 0.01	N/A
**Supp. Fig. 5B**	Correlation<br>pTau(S422) and rectal temperature	N/A	Correlation with Pearson coefficient (r=-0.5437)<br>* p< 0.05	N/A
**Supp. Fig. 5C**	Correlation<br>TauC3 and rectal temperature	N/A	Correlation with Pearson coefficient (r=0.4251)<br>p=0.06, ns	N/A
**Supp. Fig. 5D**	Correlation<br>Tau46 and rectal temperature	N/A	Correlation with Pearson coefficient (r=-0.2761)<br>p=0.2386, ns	N/A
**Supp. Fig. 5E**	Correlation<br>SDC3 and rectal temperature	N/A	Correlation with Pearson coefficient (r=0.5688)<br>** p< 0.01	N/A
**Supp. Fig. 5F**	Correlation	N/A	Correlation with Pearson coefficient (r=-0.5933)	N/A

	PIP2 and rectal temperature		* $p < 0.05$	

Supp. Fig. 6	Description	Normality test	Statistical analysis	Post-test
**Supp. Fig. 6B**	Correlation<br>Brain and body temperatures	N/A	Correlation with Pearson coefficient (r=0.8954)<br><br>*** $p < 0.0001$	N/A
**Supp. Fig. 6D**	WB Caspase-3<br>Hypo/Normo/hyperthermic - hTau	Kologorov-Smirnov	One-way ANOVA<br><br>$F_{2,13} = 6.71$ ($p < 0.01$)	Tukey's Multiple Comparison Test
	WB pTau(S422)<br>Hypo/Normo/hyperthermic - hTau	Kologorov-Smirnov	One-way ANOVA<br><br>$F_{2,14} = 21.81$ ($p < 0.0001$)	Tukey's Multiple Comparison Test
	WB TauC3<br>Hypo/Normo/hyperthermic - hTau	Kologorov-Smirnov	One-way ANOVA<br><br>$F_{2,14} = 5.52$ ($p < 0.05$)	Tukey's Multiple Comparison Test
	WB Tau46<br>Hypo/Normo/hyperthermic - hTau	Kologorov-Smirnov	One-way ANOVA<br><br>$F_{2,14} = 5.66$ ($p < 0.05$)	Tukey's Multiple Comparison Test
	WB SDC3<br>Hypo/Normo/hyperthermic - hTau	Kologorov-Smirnov	One-way ANOVA<br><br>$F_{2,14} = 15.85$ ($p < 0.001$)	Tukey's Multiple Comparison Test
	WB PIP2<br>Hypo/Normo/hyperthermic - hTau	Kologorov-Smirnov	One-way ANOVA<br><br>$F_{2,14} = 46.96$ ($p < 0.0001$)	Tukey's Multiple Comparison Test
**Supp. Fig. 6E**	Correlation<br>Caspase-3 and rectal temperature	N/A	Correlation with Pearson coefficient (r=0.6396)<br><br>** $p < 0.01$	N/A
**Supp. Fig. 6F**	Correlation<br>pTau(S422) and rectal temperature	N/A	Correlation with Pearson coefficient (r=-0.7962)<br><br>*** $p < 0.0001$	N/A
**Supp. Fig. 6G**	Correlation<br>TauC3 and rectal temperature	N/A	Correlation with Pearson coefficient (r=0.6212)<br><br>** $p < 0.01$	N/A

**Supp. Fig. 6H**	Correlation Tau46 and rectal temperature	N/A	Correlation with Pearson coefficient (r=-0.6786) ** p< 0.01	N/A
**Supp. Fig. 6I**	Correlation SDC3 and rectal temperature	N/A	Correlation with Pearson coefficient (r=0.3698) p=0.1441, ns	N/A
**Supp. Fig. 6J**	Correlation PIP2 and rectal temperature	N/A	Correlation with Pearson coefficient (r=-0.5039) * p< 0.05	N/A

**Supp. Fig. 7**	**Description**	**Normality test**	**Statistical analysis**	**Post-test**
**Supp. Fig. 7A**	Correlation CSF tau & oral temperature (8AM-8PM)	Kologorov-Smirnov	Correlation with Pearson coefficient (r=0.41) ns, p=0.17	N/A
**Supp. Fig. 7B**	Correlation CSF NfL & oral temperature (8AM-8PM)	Kologorov-Smirnov	Correlation with Pearson coefficient (r=0.05) ns	N/A
**Supp. Fig. 7C**	Correlation plasma tau (7AM-7PM) & body temperature (7PM-7AM)	Kologorov-Smirnov	Correlation with Pearson coefficient (r=0.46) ns, p=0.08	N/A

**Supp. Fig. 10**	**Description**	**Normality test**	**Statistical analysis**	**Post-test**
**Supp. Fig. 10A**	LDH values of Fig. 2F	Kologorov-Smirnov	Two-way ANOVA<br>$F_{2,39}$ = 1.5 for temperature, ns<br>$F_{1,39}$ = 0.06 for treatment, ns<br>$F_{2,39}$ = 1.2 for interaction, ns	Šĺdák's Multiple Comparison Test
**Supp. Fig. 10B**	LDH values of Fig. 2H	Kologorov-Smirnov	Unpaired t test with Welch's correction<br>two-tailed P value	Šĺdák's Multiple Comparison Test

			P=0.96, ns	
**Supp. Fig. 10C**	LDH values of Fig. 2I	Kologorov-Smirnov	Unpaired t test with Welch's correction<br>two-tailed P value<br>P=0.87, ns	N/A
**Supp. Fig. 10D**	LDH values of Fig. 3E/3F	Kologorov-Smirnov	One-way ANOVA<br>$F_{2,20}$ = 1.37 (p=0.28, ns)	Tukey's Multiple Comparison Test
**Supp. Fig. 10E**	LDH values of Fig. 3G	Kologorov-Smirnov	Unpaired t test with Welch's correction<br>two-tailed P value<br>P=0.80, ns	N/A
**Supp. Fig. 10F**	LDH values of Fig. S1D	Kologorov-Smirnov	Two-way ANOVA<br>$F_{4,42}$ = 0.51 for group, ns<br>$F_{2,42}$ = 0.71 for time, ns<br>$F_{2,42}$ = 0.14 for interaction, ns	Tukey's Multiple Comparison Test
