# Supplementary material for: Sleep-wake variation in body temperature regulates tau secretion and correlates with CSF and plasma tau
Source: J Clin Invest. 2025 Feb 4;135(7):e182931. doi: 10.1172/JCI182931 (PMC11957704; doi:10.1172/JCI182931)

Raw membranes used in Western blot and Dot blot for

Sleep-wake body temperature variation regulates tau secretion and correlates with CSF and plasma tau

Geoffrey Canet[1,2], Felipe Da Gama Monteiro[1,3], Emma Rocaboy[2], Sofia Diego-Diaz[2], Boutheyna Khelaifia[1,2], Kelly Godbout[2], Aymane Lachhab[2], Jessica Kim[4], Daphne I. Valencia[5], Audrey Yin[4], Hau-Tieng Wu[4], Jordan Howell[4], Emily Blank[4], Francis Laliberté[1], Nadia Fortin[1], Emmanuelle Boscher[1,2], Parissa Fereydouni-Forouzandeh[2], Stéphanie Champagne[2], Isabelle Guisle[1,2], Sébastien S. Hébert[1,2], Vincent Pernet[1,2,6], Haiyan Liu[7], William Lu[7], Ludovic Debure[4], David M. Rapoport[5], Indu Ayappa[5], Andrew W. Varga[5], Ankit Parekh[5], Ricardo S. Osorio[4], Steve Lacroix[1,3], Mark P. Burns[8], Brendan P. Lucey[7], Esther M. Blessing[4*], Emmanuel Planel[1,2*]*

FIGURE 1

Figure 1B

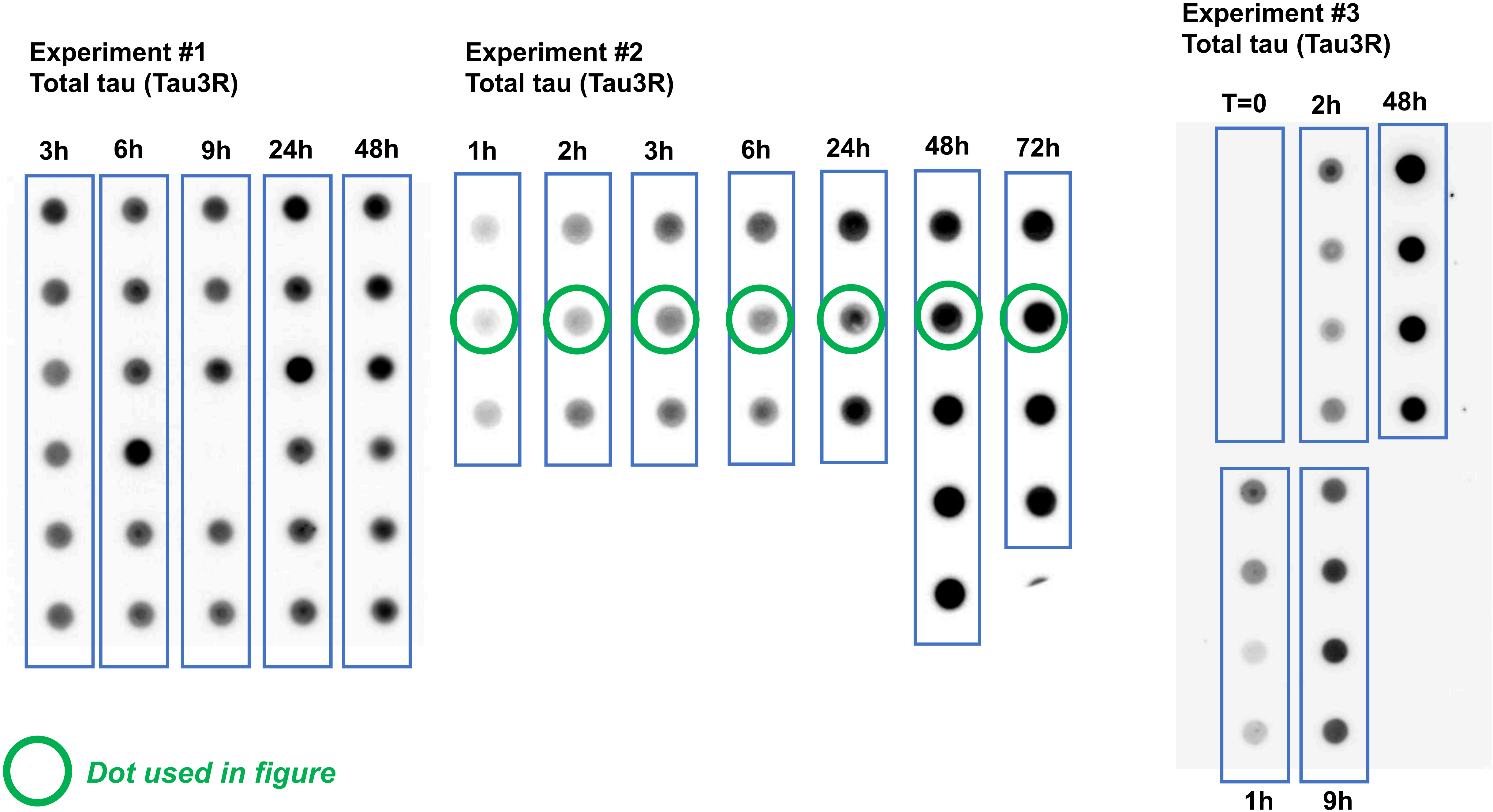
Experiment #1
Total tau (Tau3R)
3h
6h
9h
24h
48h
Experiment #2
Total tau (Tau3R)
1h
2h
3h
6h
24h
48h
72h
Experiment #3
Total tau (Tau3R)
T=0
2h
48h
1h
9h
Dot used in figure

Figure 1D, E

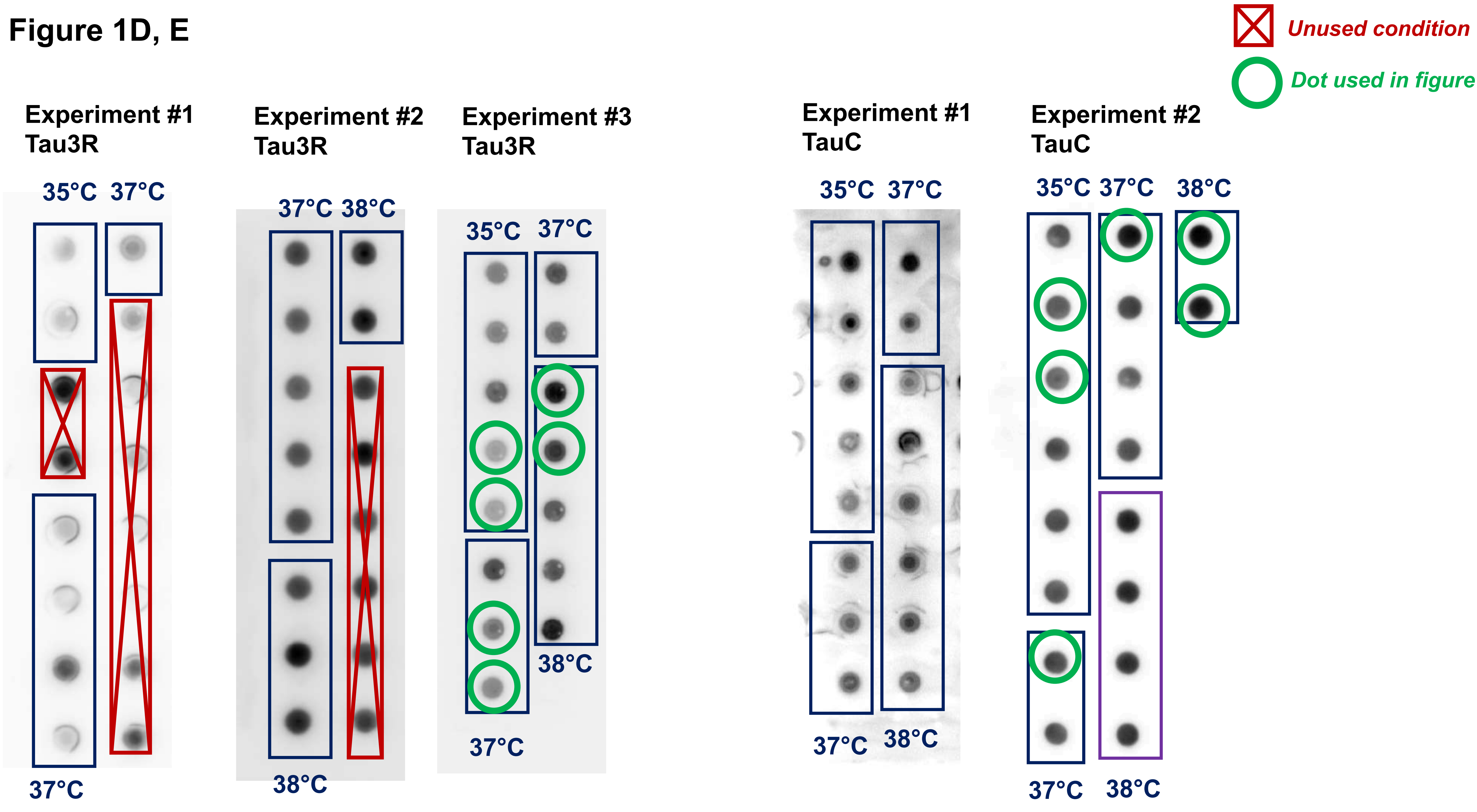

Unused condition
Dot used in figure
Experiment #1
Tau3R
35°C 37°C
37°C
Experiment #2
Tau3R
37°C 38°C
38°C
Experiment #3
Tau3R
35°C 37°C
38°C
37°C
Experiment #1
TauC
35°C 37°C
37°C 38°C
Experiment #2
TauC
35°C 37°C 38°C
37°C 38°C

Figure 1 D,E

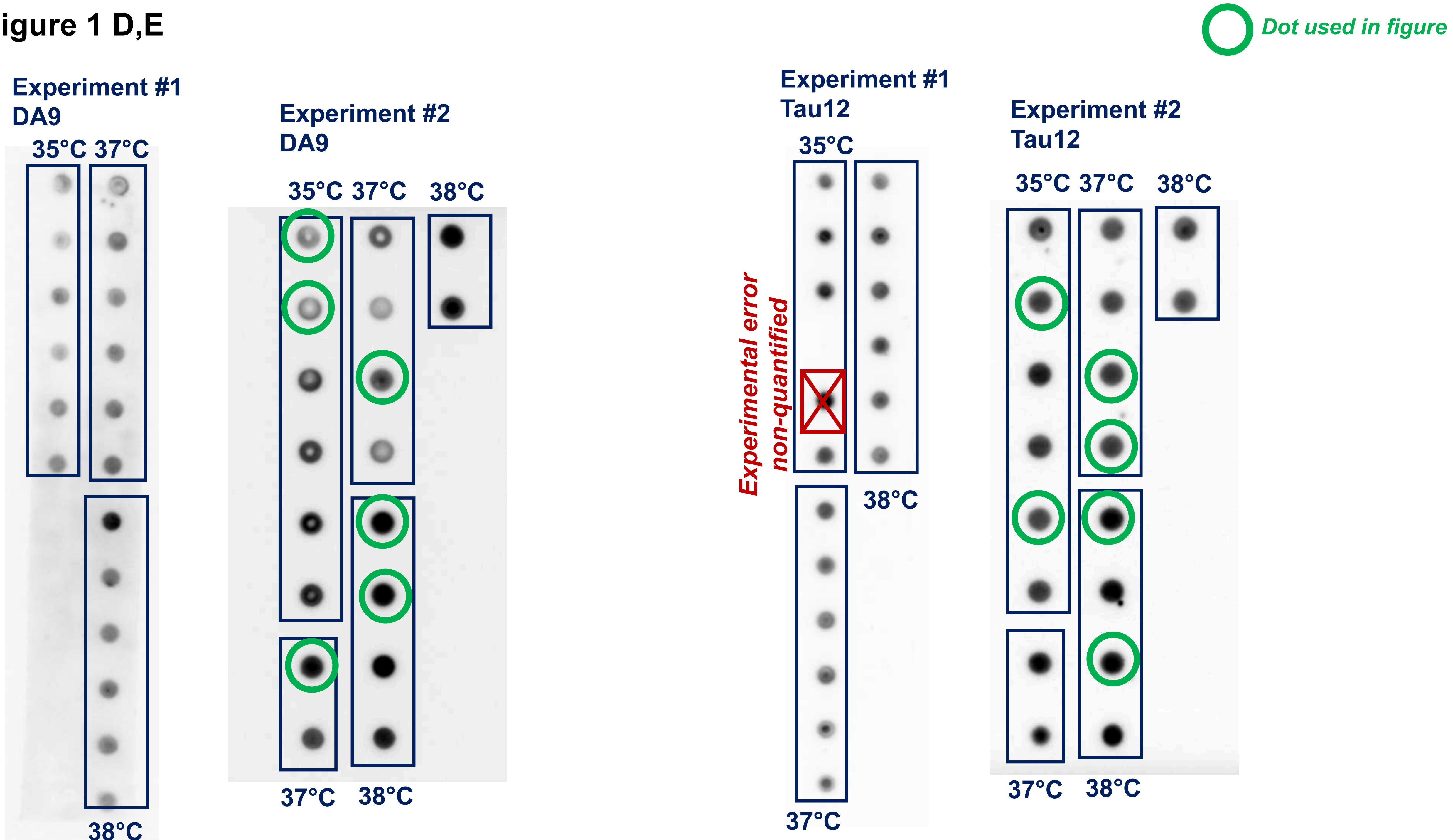

Dot used in figure
Experiment #1
DA9
35°C 37°C
38°C
Experiment #2
DA9
35°C 37°C 38°C
37°C 38°C
Experiment #1
Tau12
35°C
Experimental error
non-quantified
38°C
37°C
Experiment #2
Tau12
35°C 37°C 38°C
37°C 38°C

Figure 1 F,G

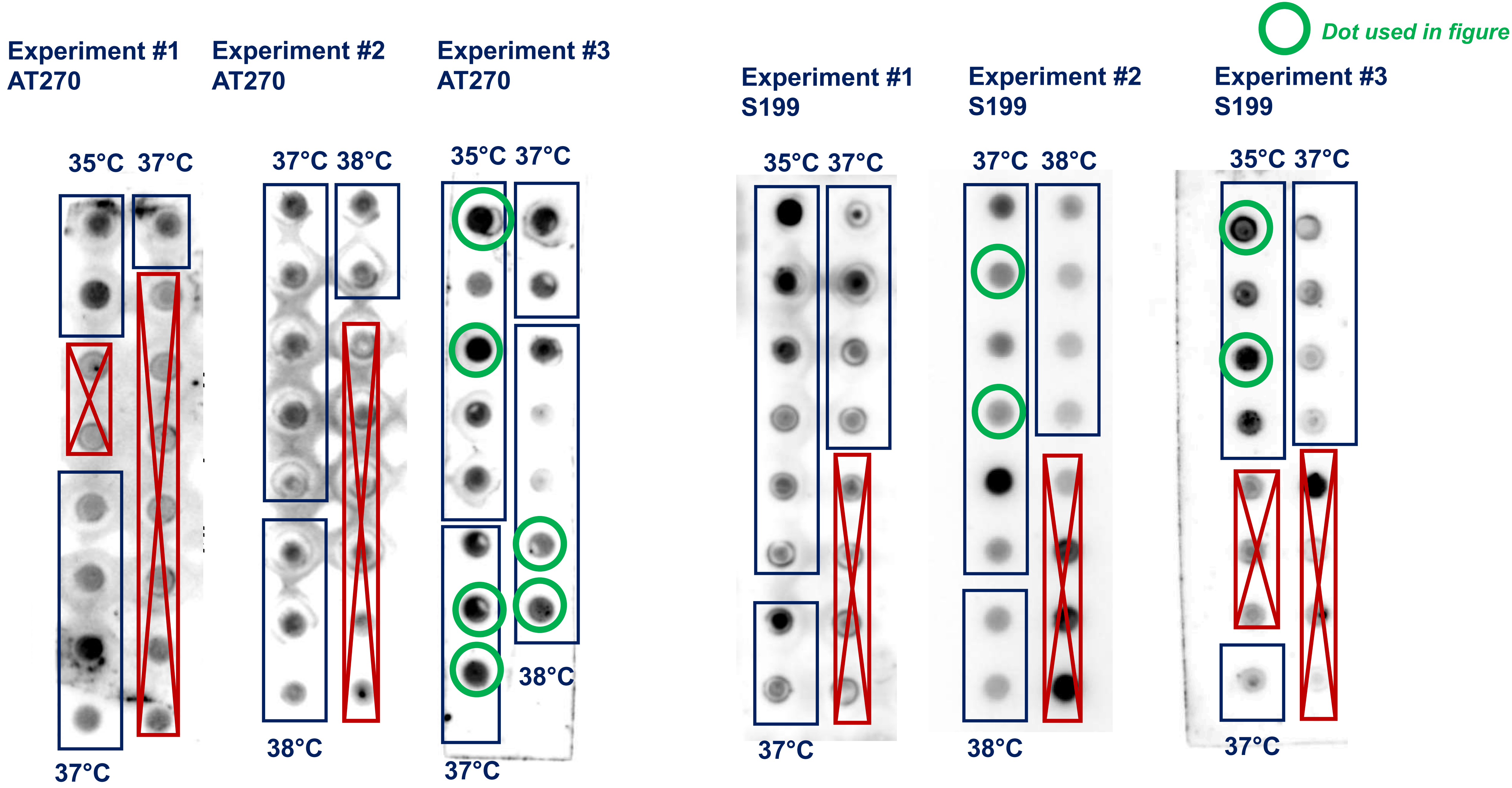

Unused condition
Dot used in figure
Experiment #1
AT270
Experiment #2
AT270
Experiment #3
AT270
Experiment #1
S199
Experiment #2
S199
Experiment #3
S199
35°C 37°C
37°C
37°C 38°C
38°C
35°C 37°C
38°C
37°C
35°C 37°C
37°C
37°C 38°C
38°C
35°C 37°C
37°C

Figure 1 F,G

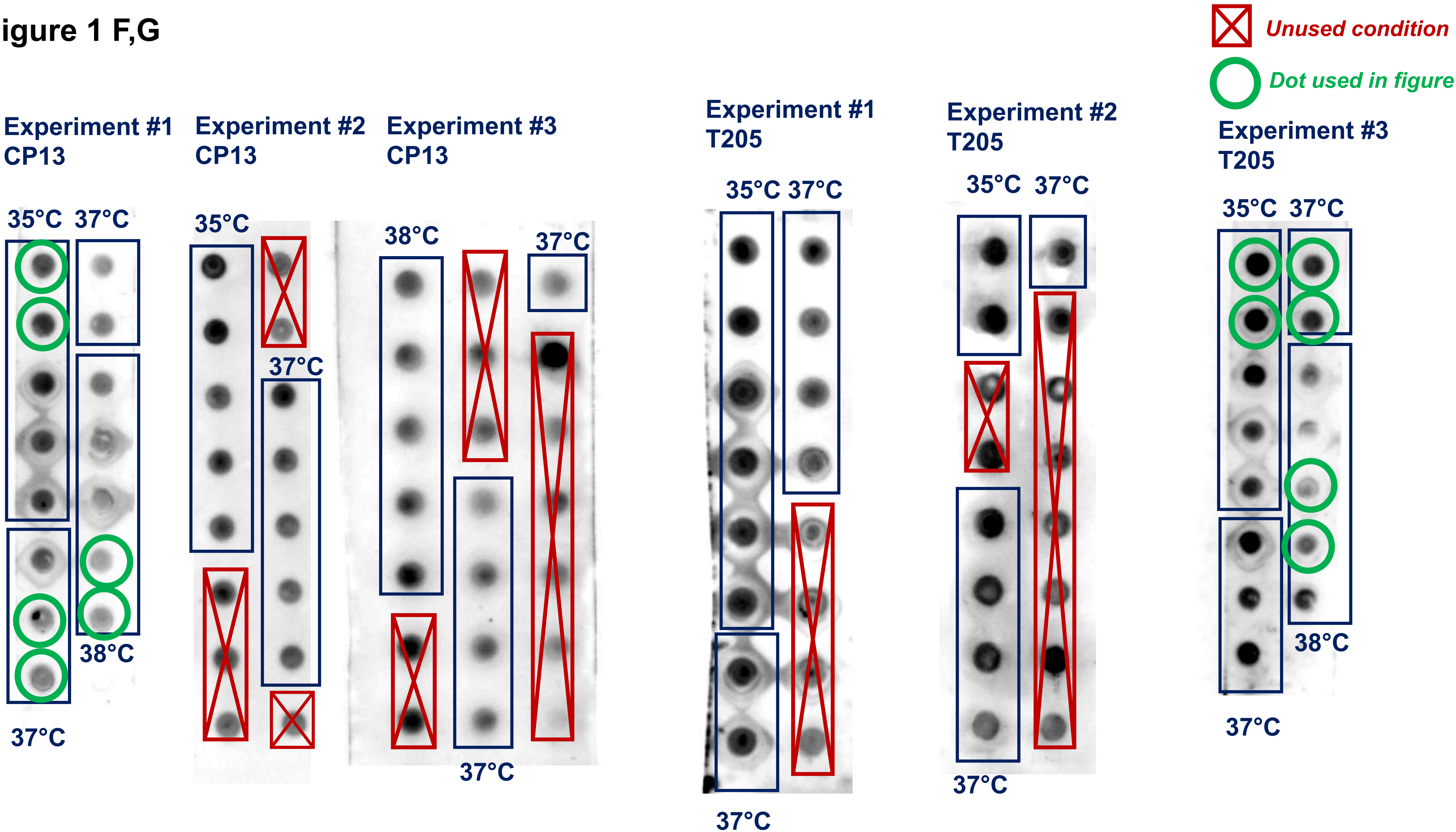

Unused condition
Dot used in figure
Experiment #1
CP13
35°C 37°C
37°C
38°C
Experiment #2
CP13
35°C
37°C
Experiment #3
CP13
38°C
37°C
37°C
Experiment #1
T205
35°C 37°C
37°C
Experiment #2
T205
35°C 37°C
37°C
Experiment #3
T205
35°C 37°C
38°C
37°C

Figure 1 F,G

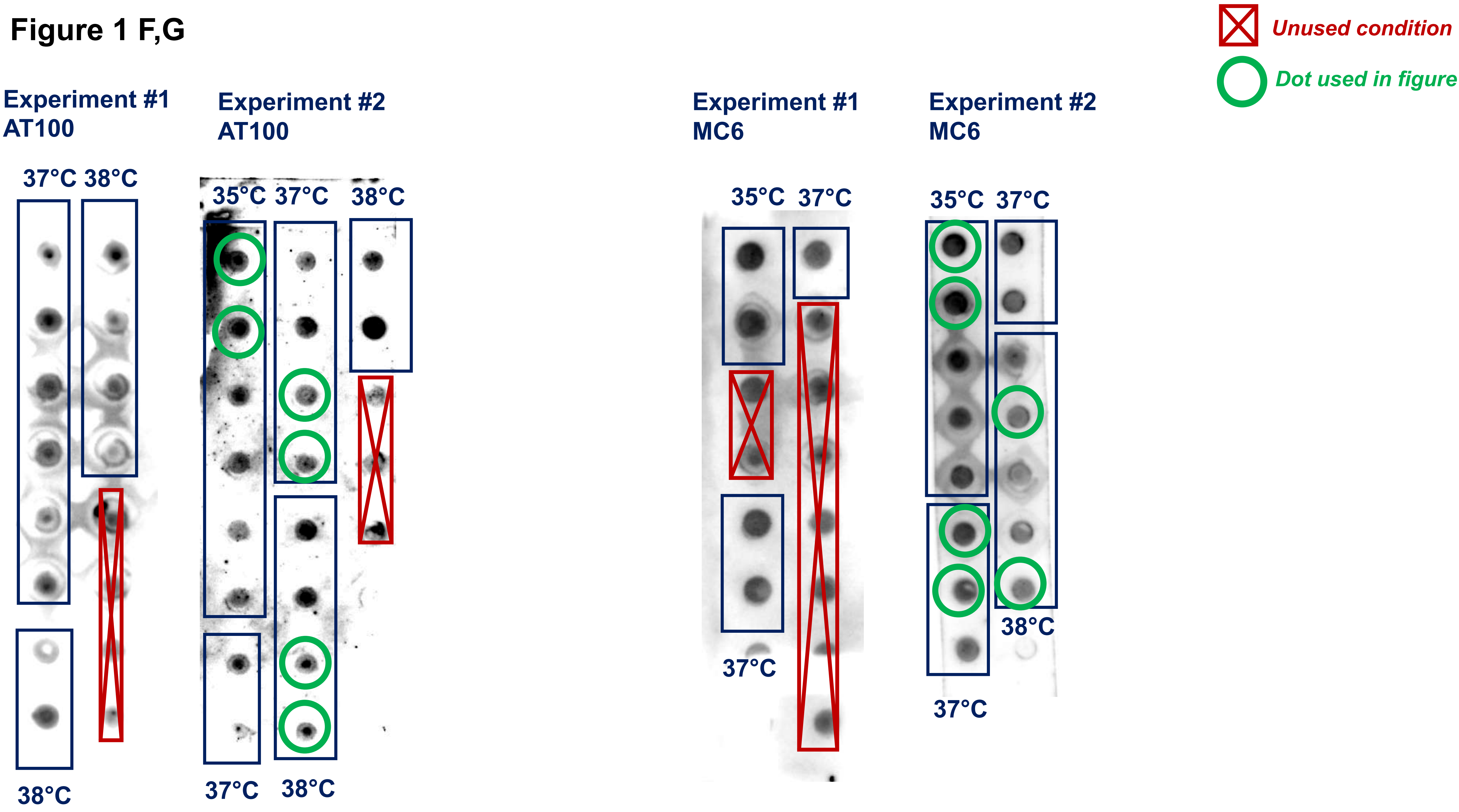

Unused condition
Dot used in figure
Experiment #1
AT100
37°C 38°C
38°C
Experiment #2
AT100
35°C 37°C 38°C
37°C 38°C
Experiment #1
MC6
35°C 37°C
37°C
Experiment #2
MC6
35°C 37°C
38°C
37°C

Figure 1 F,G

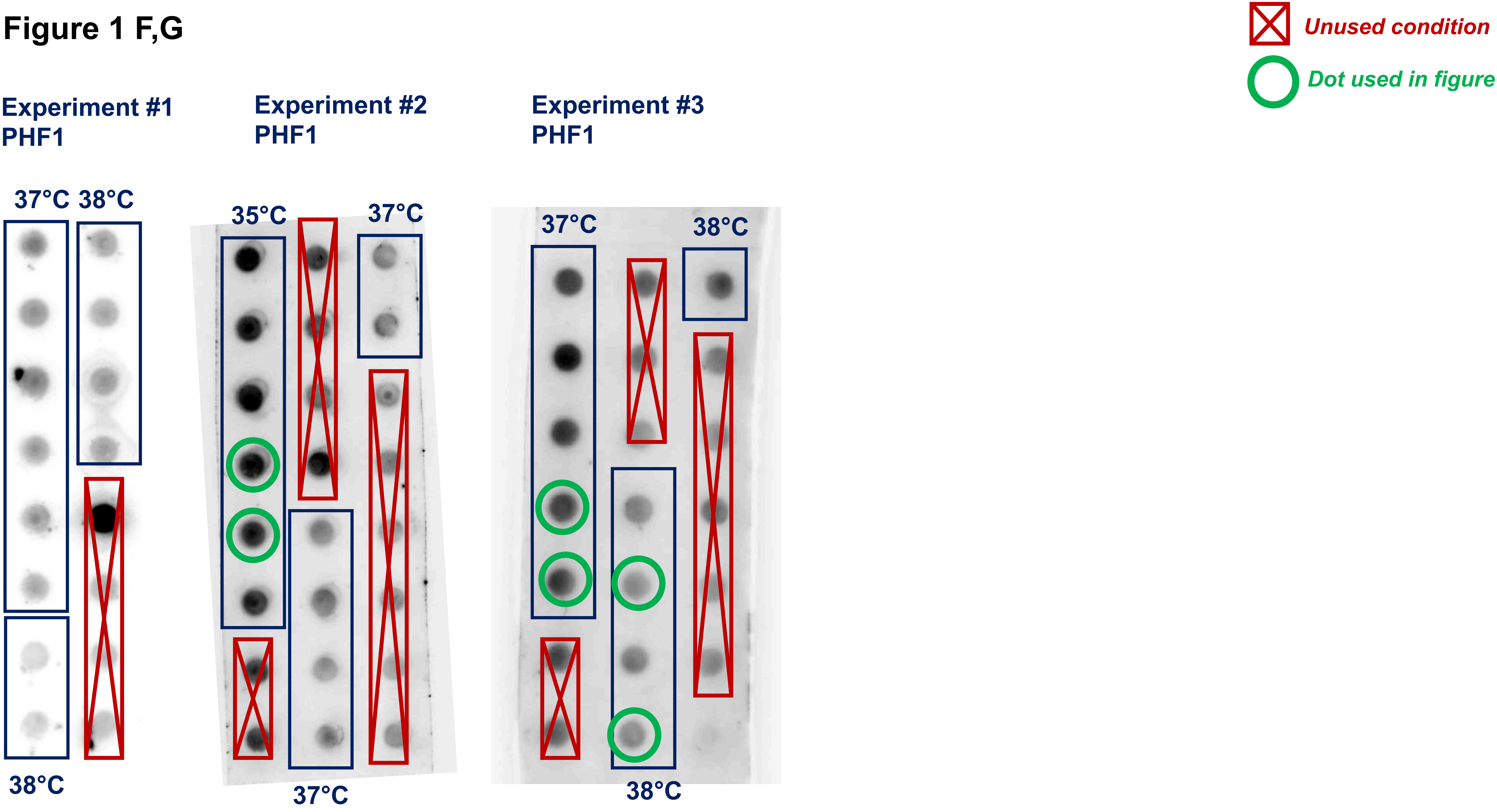

Unused condition
Dot used in figure
Experiment #1
PHF1
37°C 38°C
38°C
Experiment #2
PHF1
35°C
37°C
37°C
Experiment #3
PHF1
37°C
38°C
38°C

Figure 1 J,K

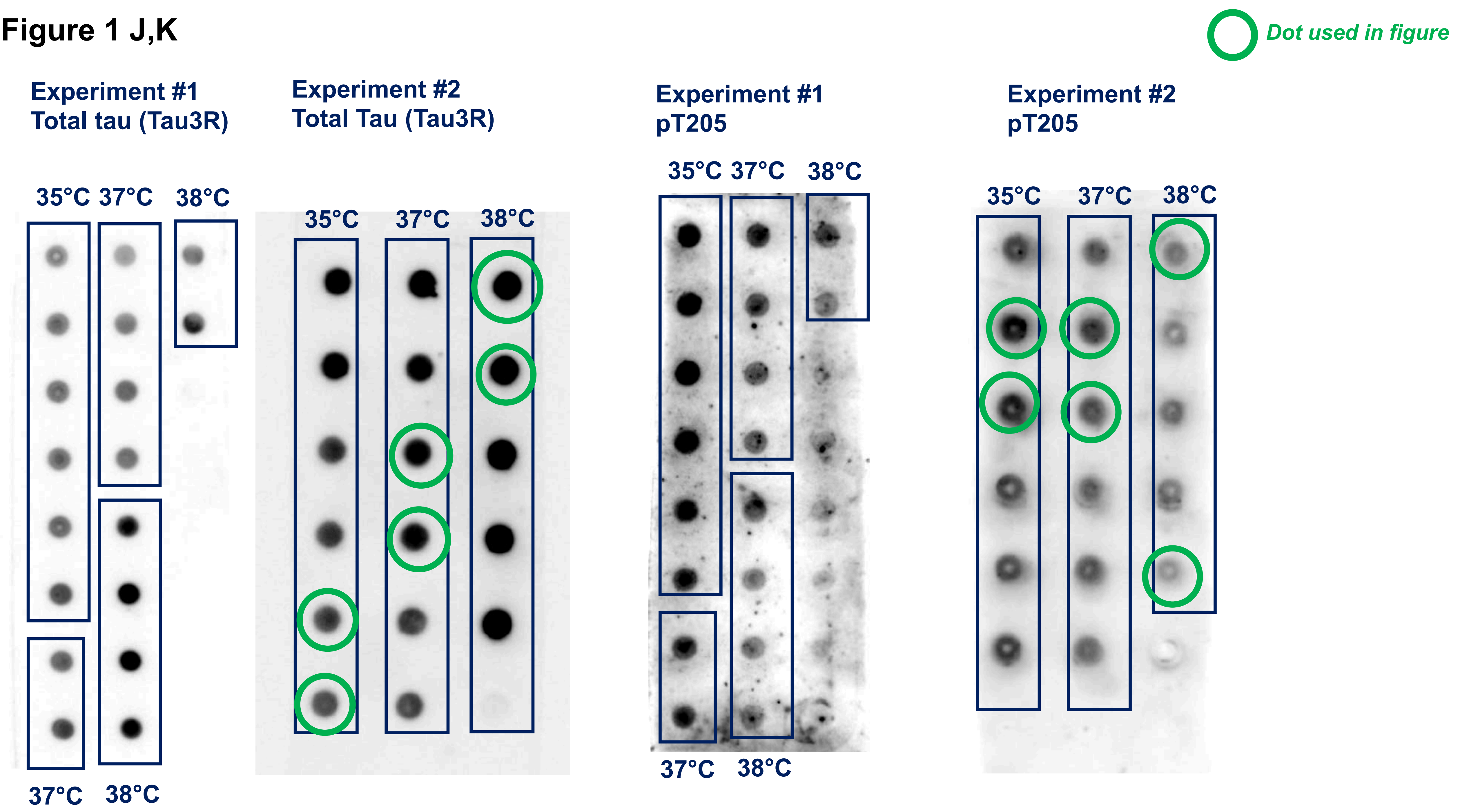
Dot used in figure
Experiment #1
Total tau (Tau3R)
35°C 37°C 38°C
37°C 38°C
Experiment #2
Total Tau (Tau3R)
35°C 37°C 38°C
Experiment #1
pT205
35°C 37°C 38°C
37°C 38°C
Experiment #2
pT205
35°C 37°C 38°C

Figure 1 J,K

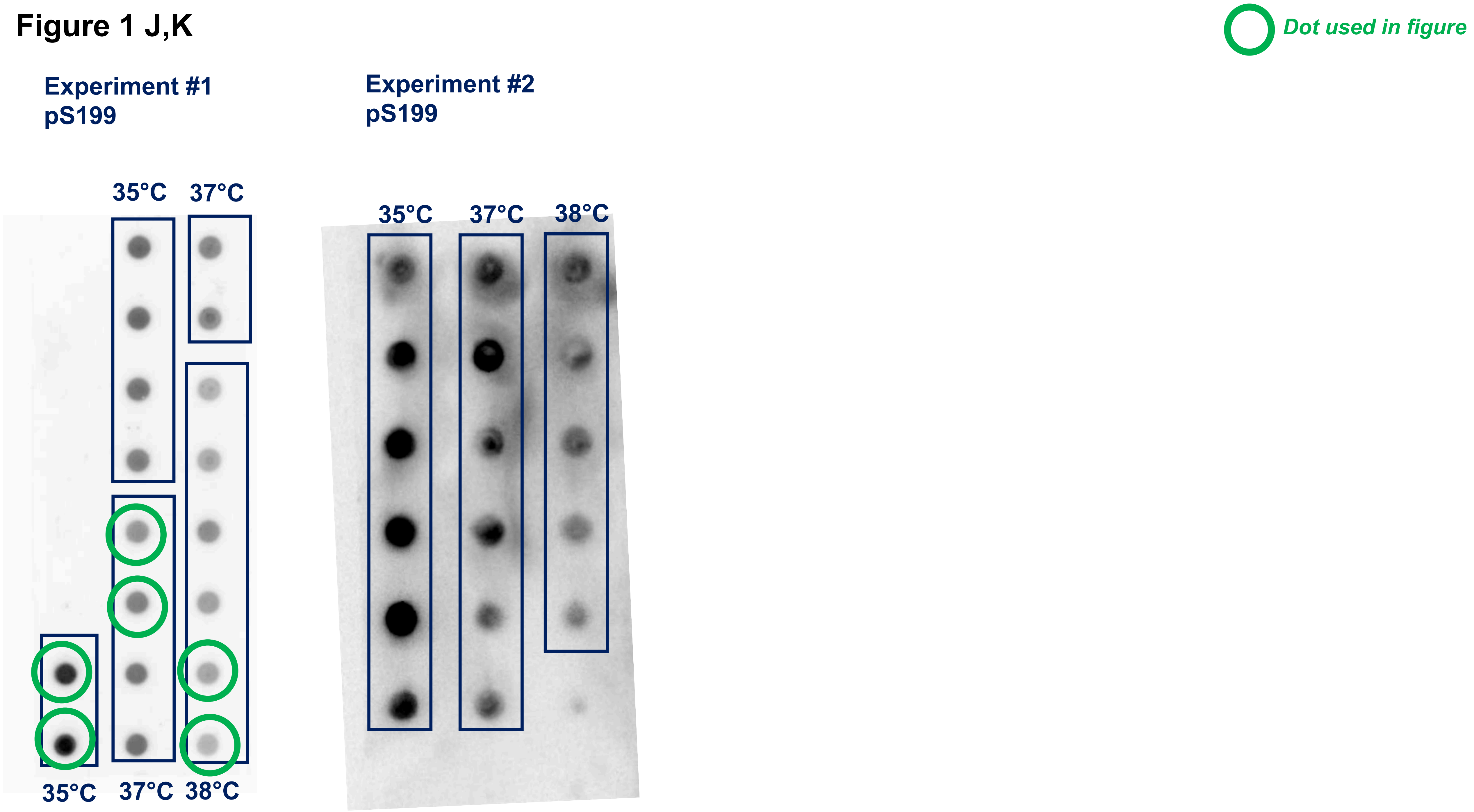

Dot used in figure
Experiment #1
pS199
35°C
37°C
35°C
37°C
38°C
Experiment #2
pS199
35°C
37°C
38°C

FIGURE 2

Figure 2B

Caspase-3 - SH-Tau3R

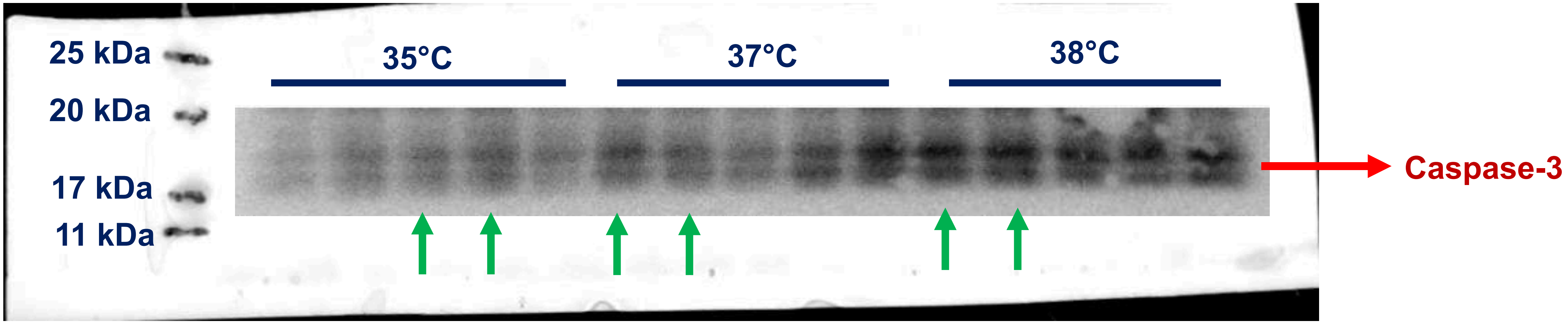

Corresponding Actin

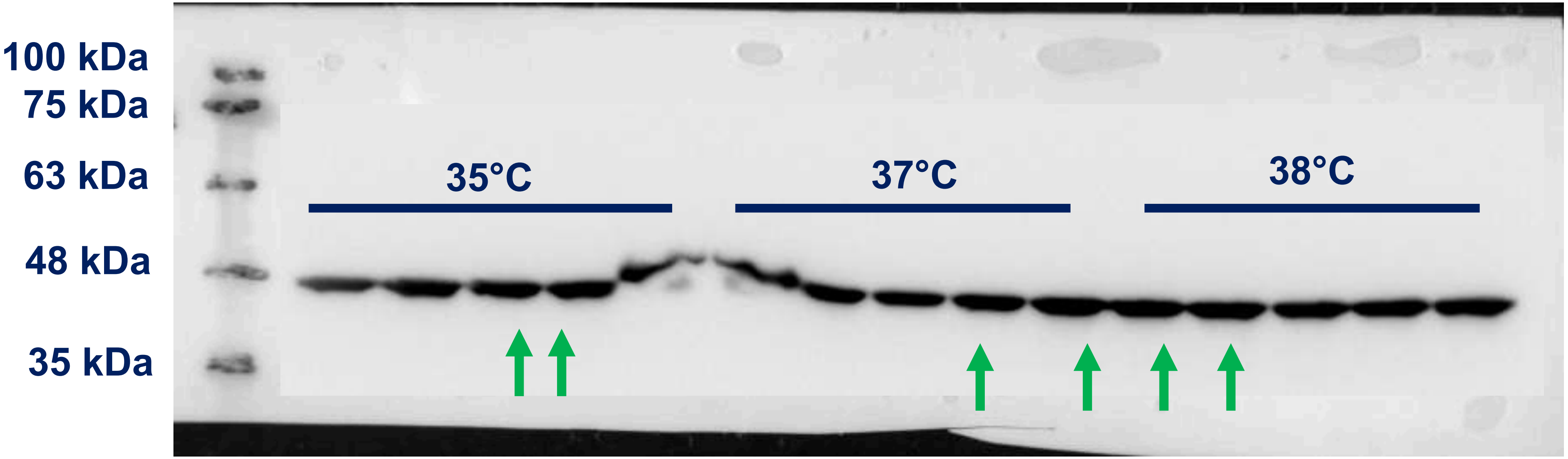

Figure 2B

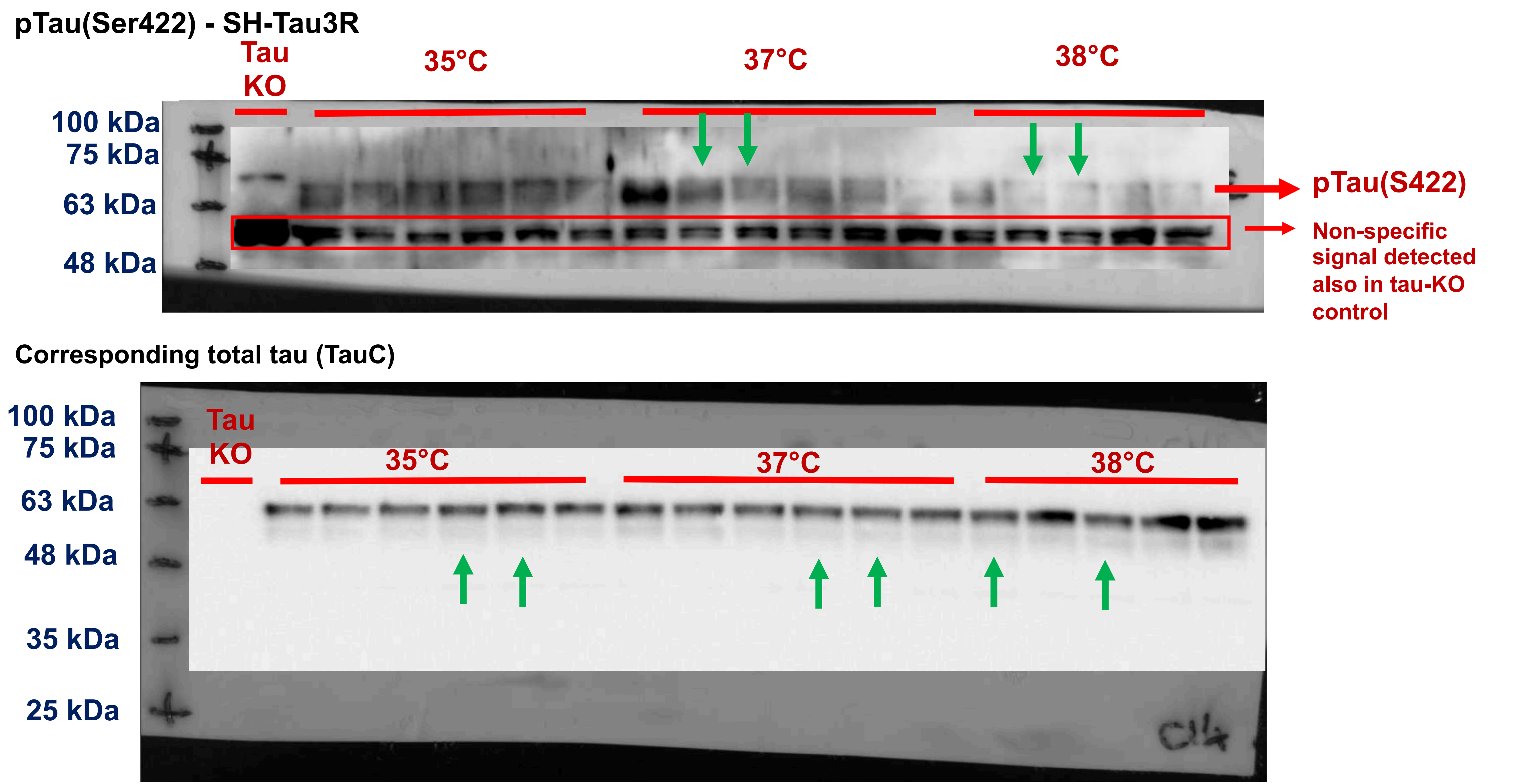

Dot used in figure
pTau(Ser422) - SH-Tau3R
Tau KO
35°C
37°C
38°C
100 kDa
75 kDa
63 kDa
48 kDa
pTau(S422)
Non-specific signal detected also in tau-KO control
Corresponding total tau (TauC)
100 kDa
75 kDa
63 kDa
48 kDa
35 kDa
25 kDa
Tau KO
35°C
37°C
38°C

Figure 2B

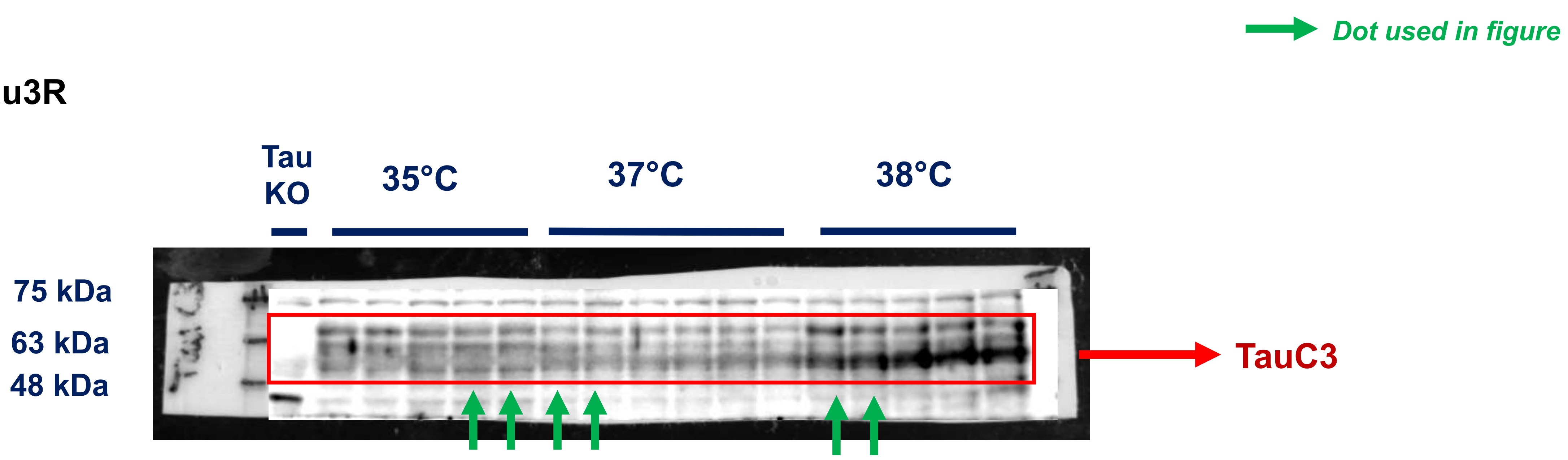

Dot used in figure
TauC3 - SH-Tau3R
Tau KO
35°C
37°C
38°C
75 kDa
63 kDa
48 kDa
TauC3

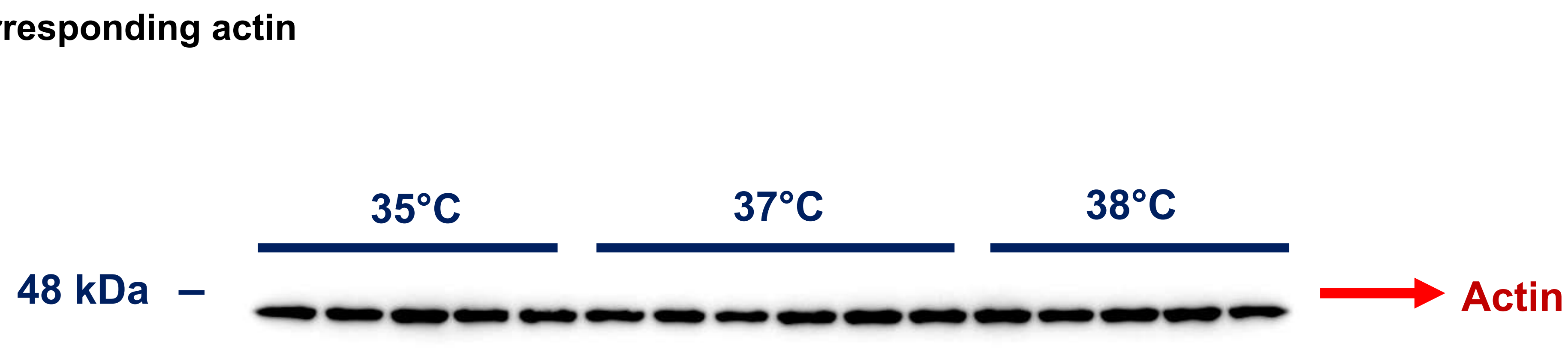

Corresponding actin
35°C
37°C
38°C
48 kDa
Actin

Figure 2B

Tau46 - SH-Tau3R

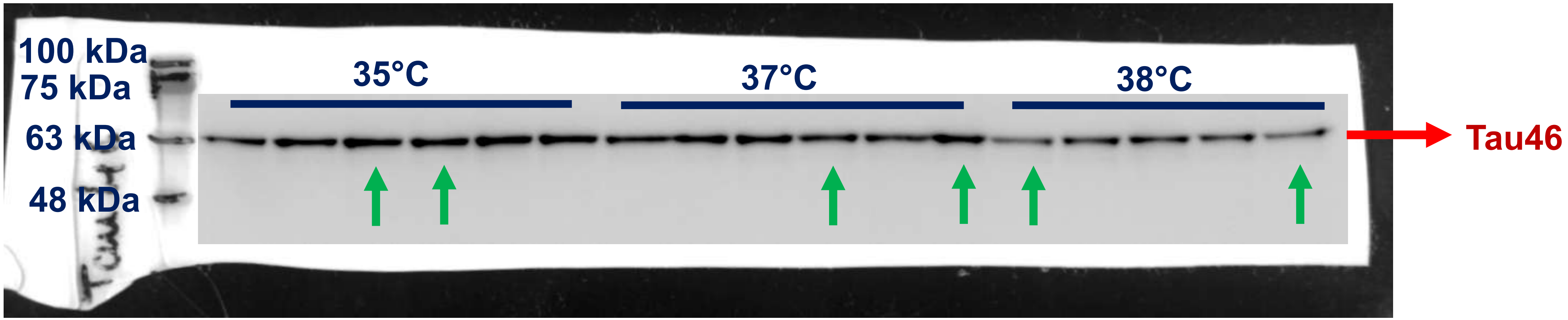

Corresponding Actin

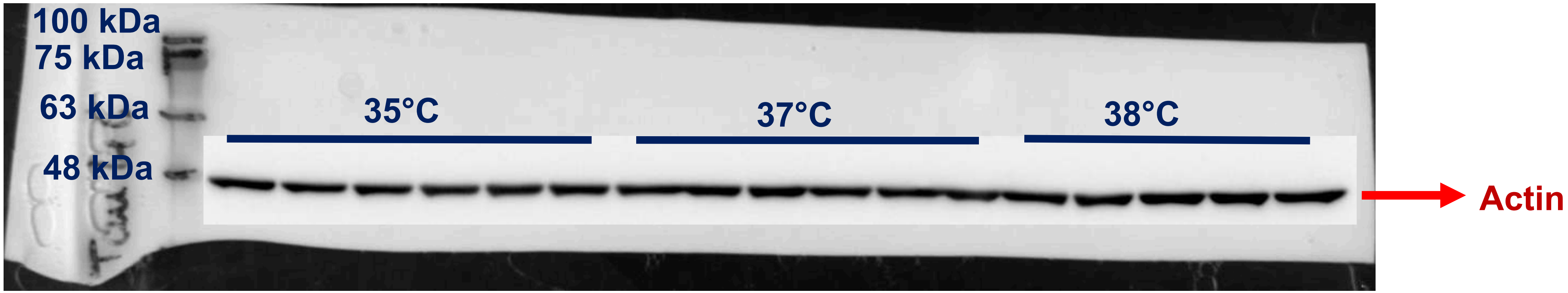

Figure 2B

Dot used in figure

Caspase-3 – Primary neurons

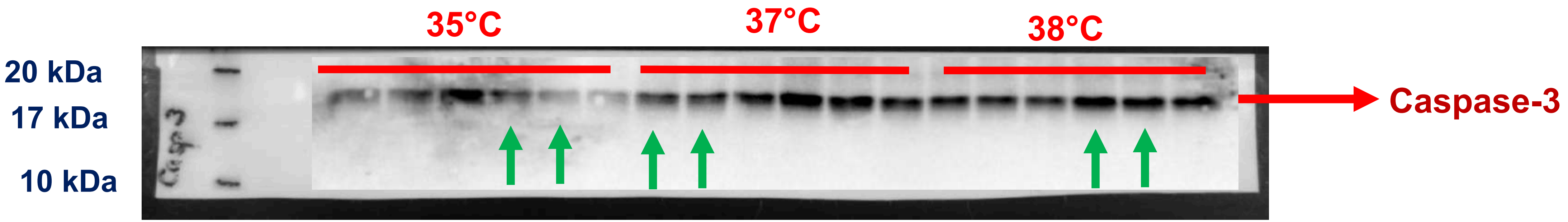

Corresponding actin

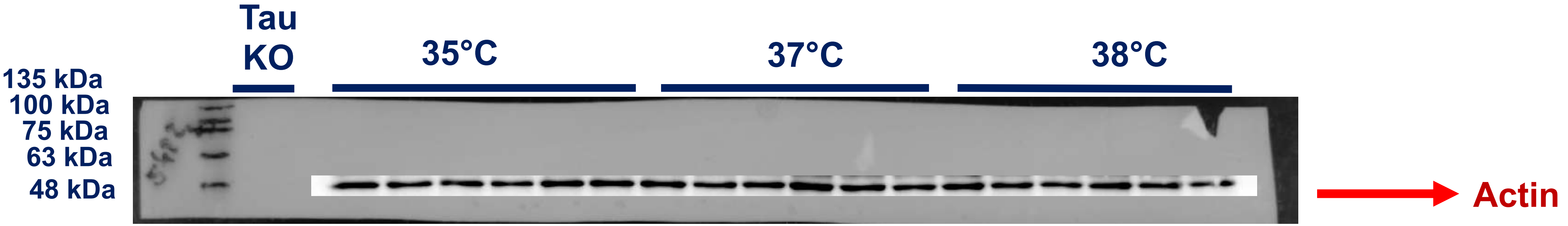

Figure 2B

Dot used in figure

TauC3 – Primary neurons

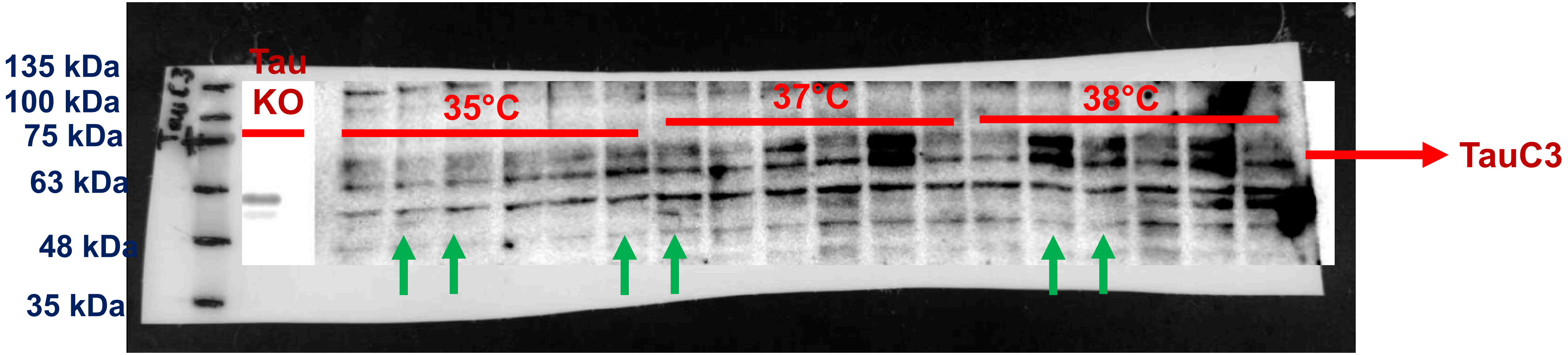

Corresponding actin

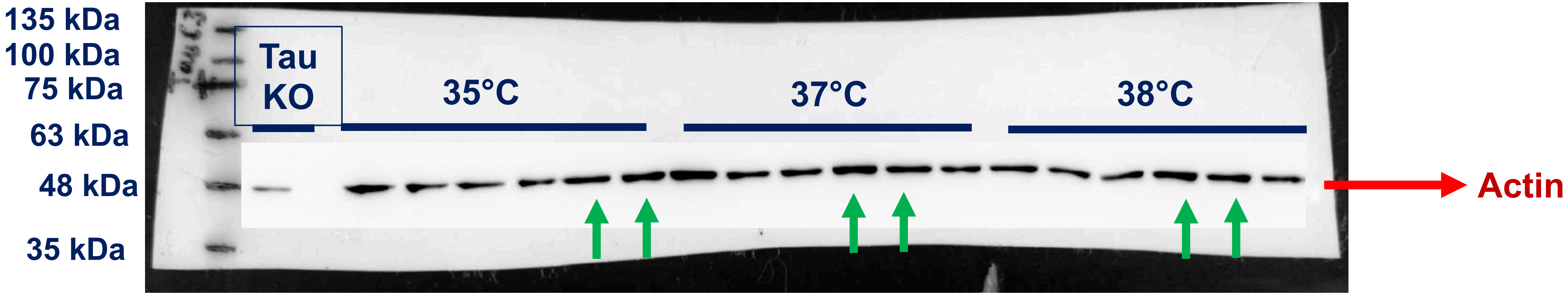

Figure 2B

S422 – Primary neurons

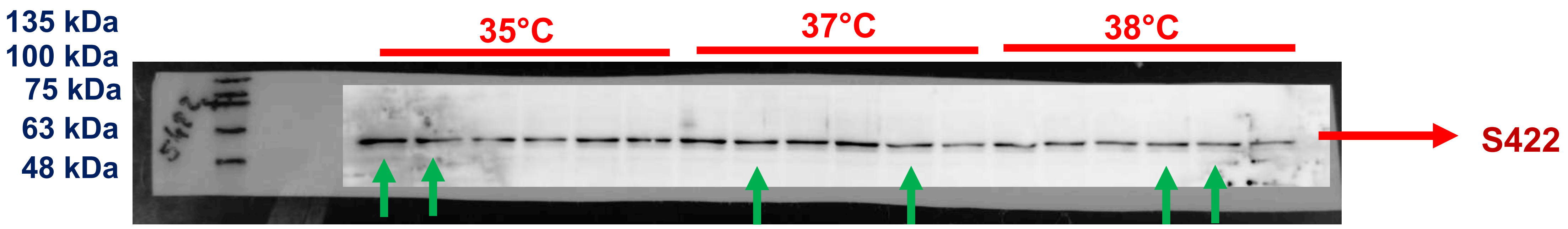

Corresponding Total tau

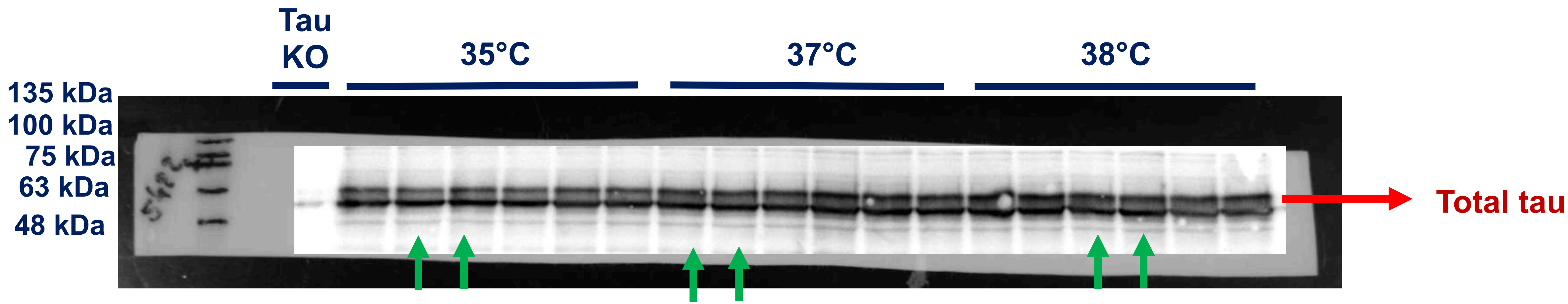

Figure 2B

Dot used in figure

Tau46 – Primary neurons

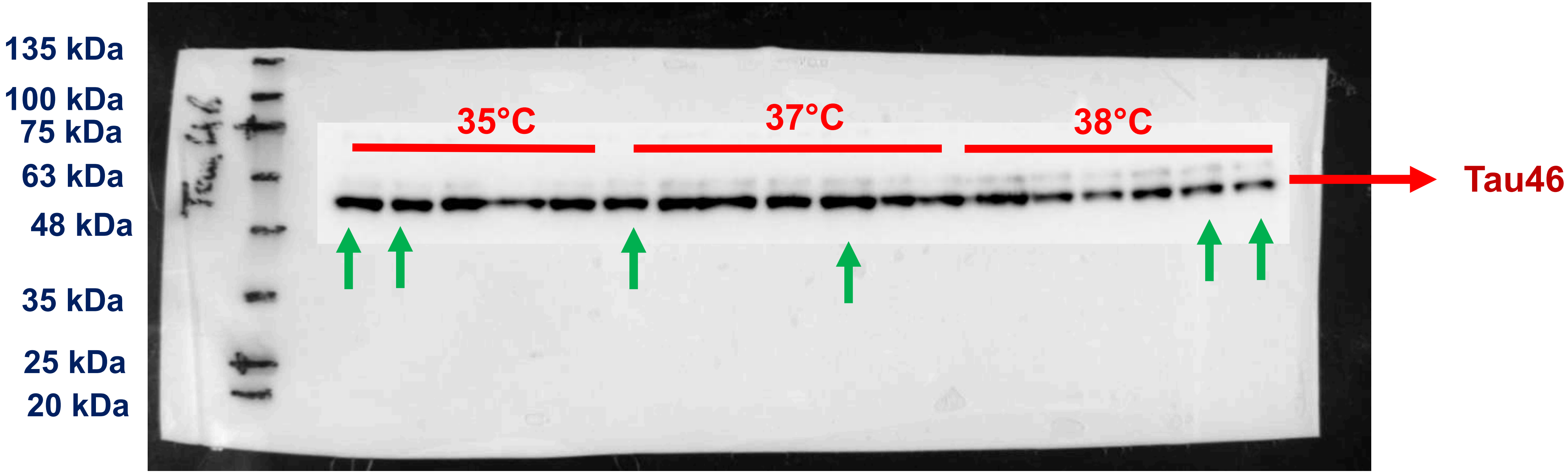

Corresponding actin

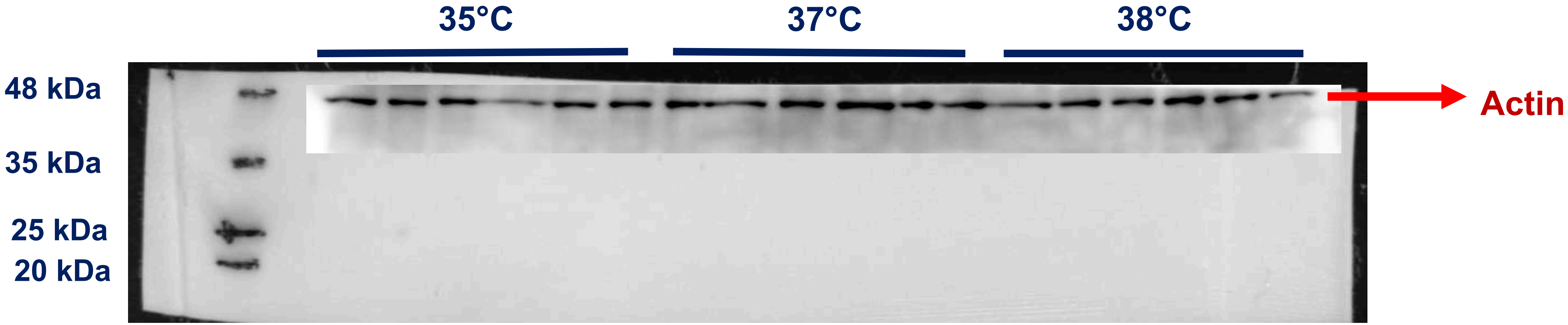

Figure 2D

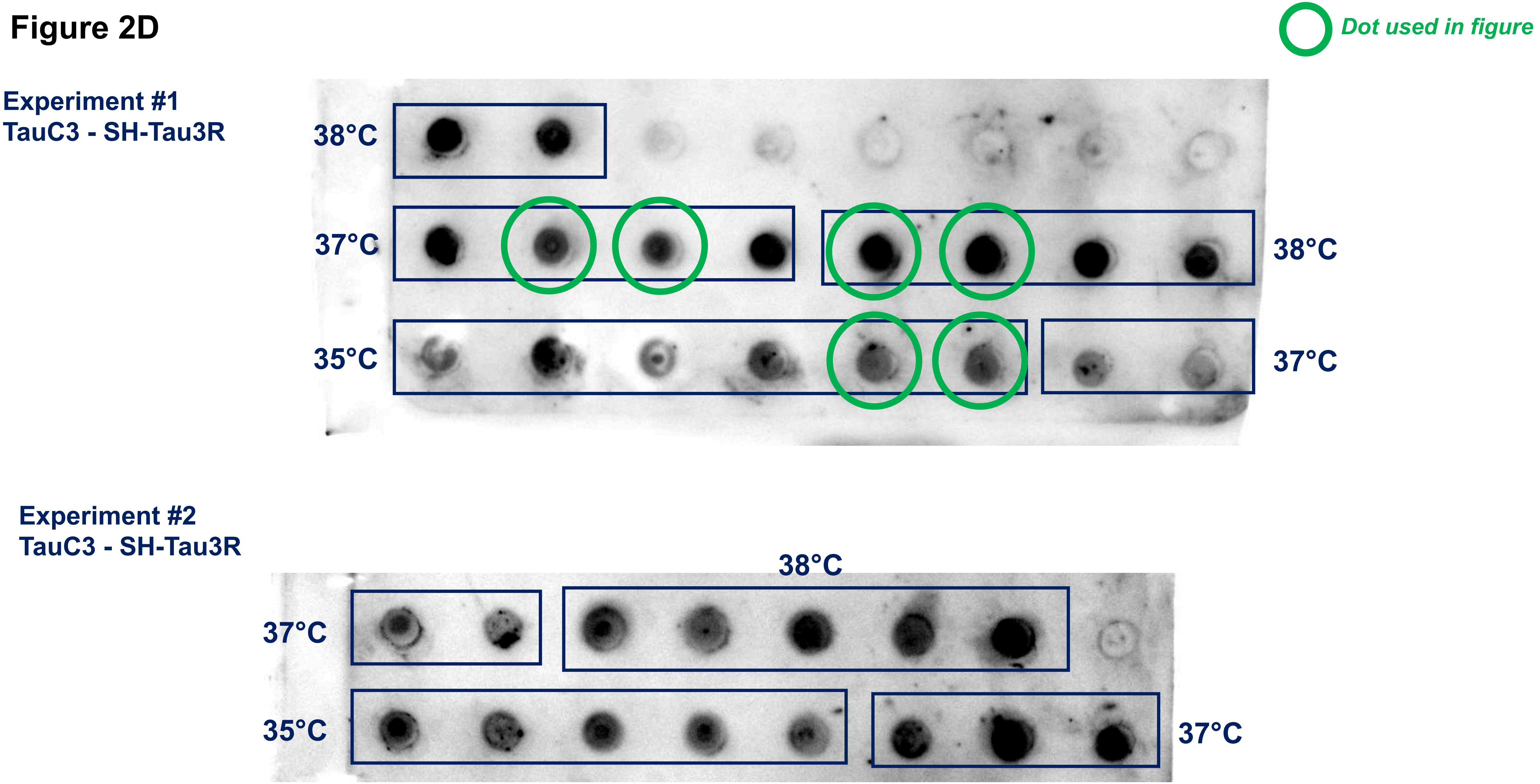

Dot used in figure
Experiment #1
TauC3 - SH-Tau3R
38°C
37°C
38°C
35°C
37°C
Experiment #2
TauC3 - SH-Tau3R
38°C
37°C
35°C
37°C

Figure 2D

Dot used in figure

Experiment #1
Tau46 – SH-Tau3R

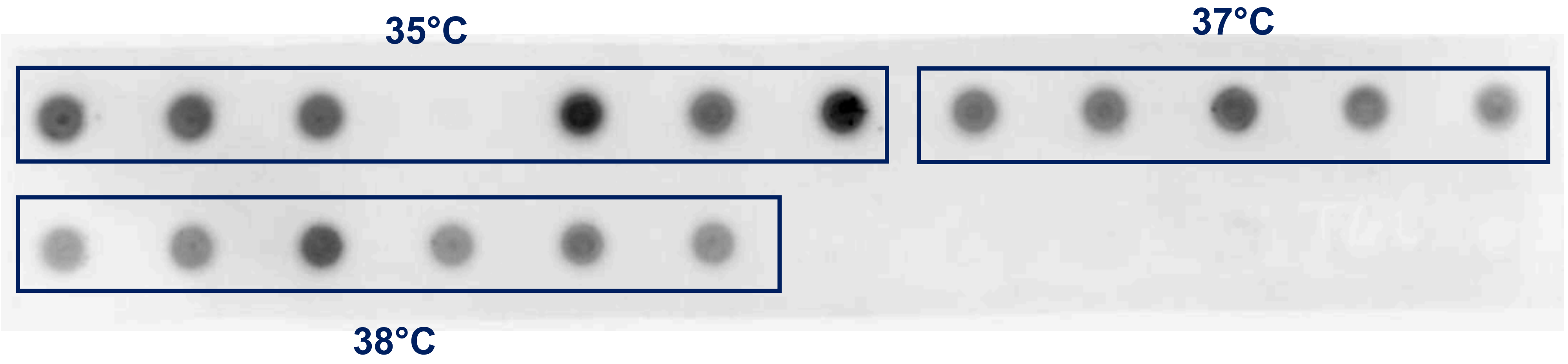

Experiment #2
Tau46 – SH-Tau3R

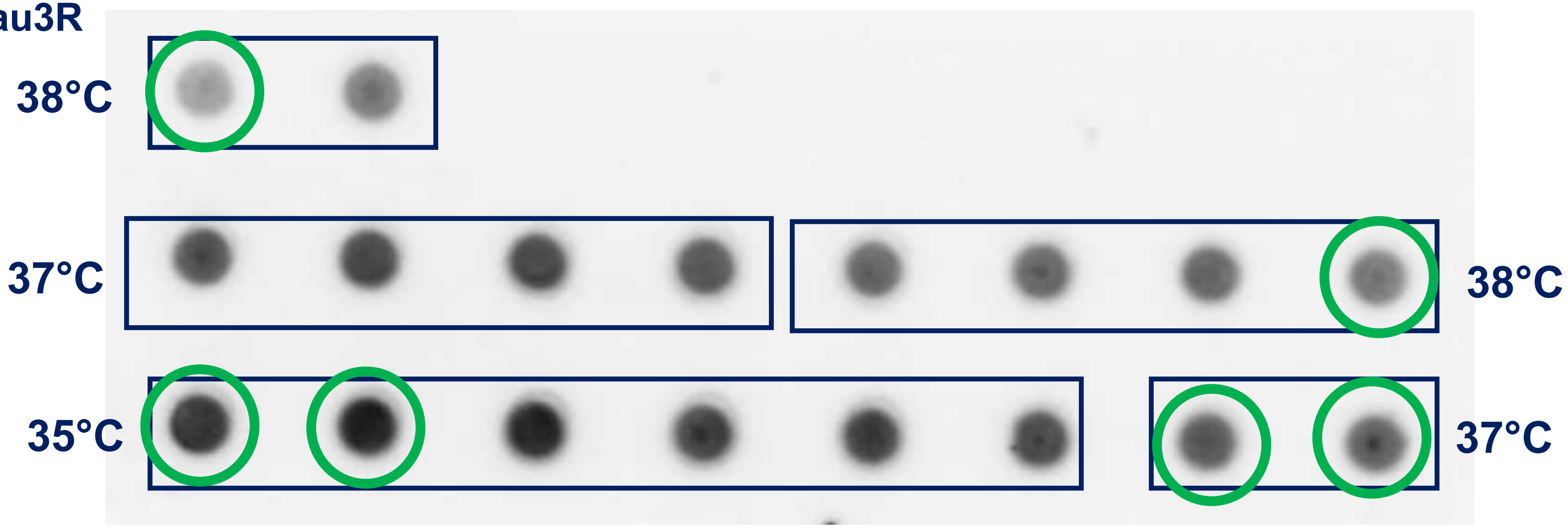

Figure 2D

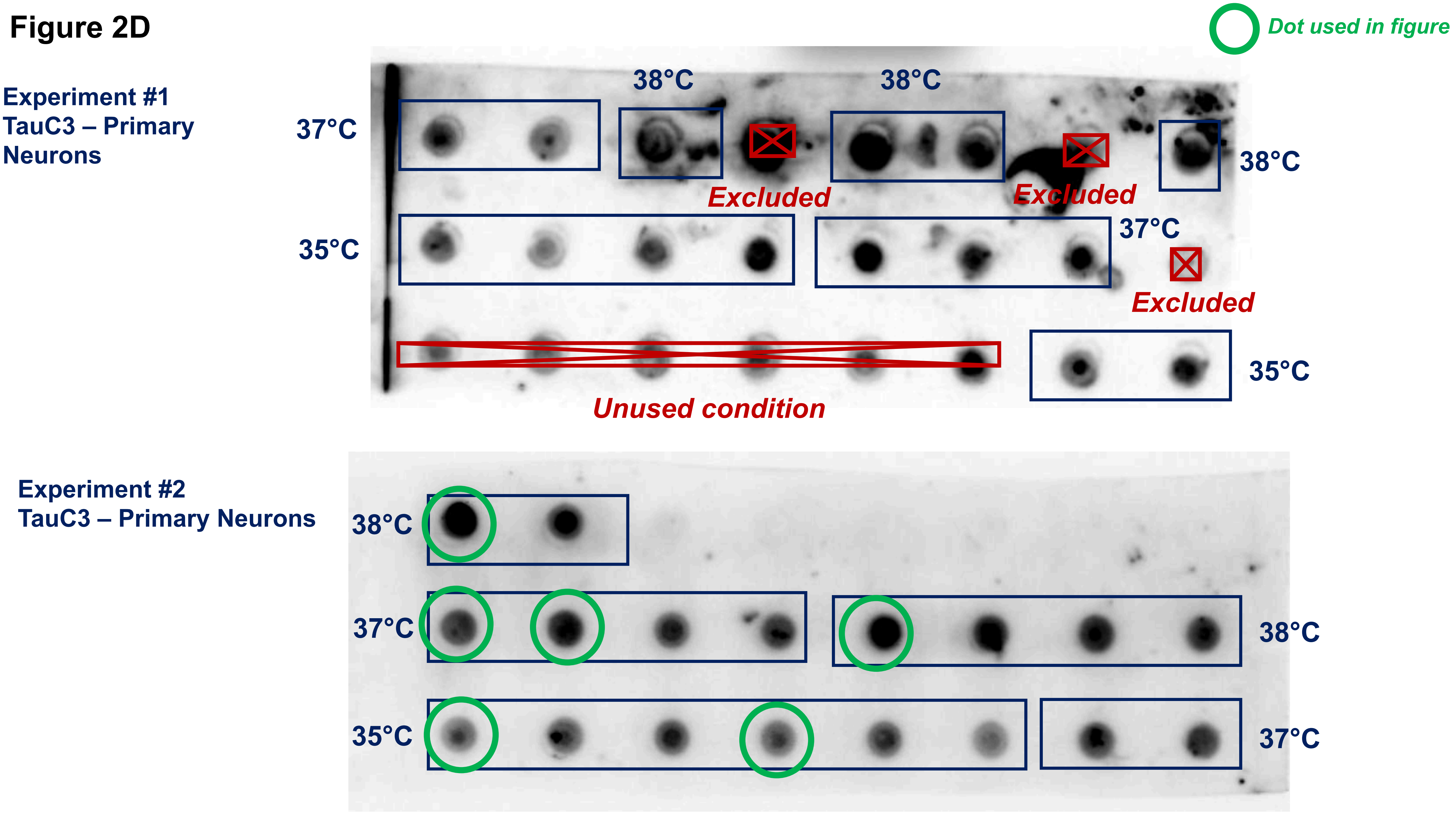

Dot used in figure
Experiment #1
TauC3 – Primary Neurons
37°C
38°C
38°C
38°C
Excluded
Excluded
35°C
37°C
Excluded
Unused condition
35°C
Experiment #2
TauC3 – Primary Neurons
38°C
37°C
38°C
35°C
37°C

Figure 2D

Tau46 – Primary Neurons

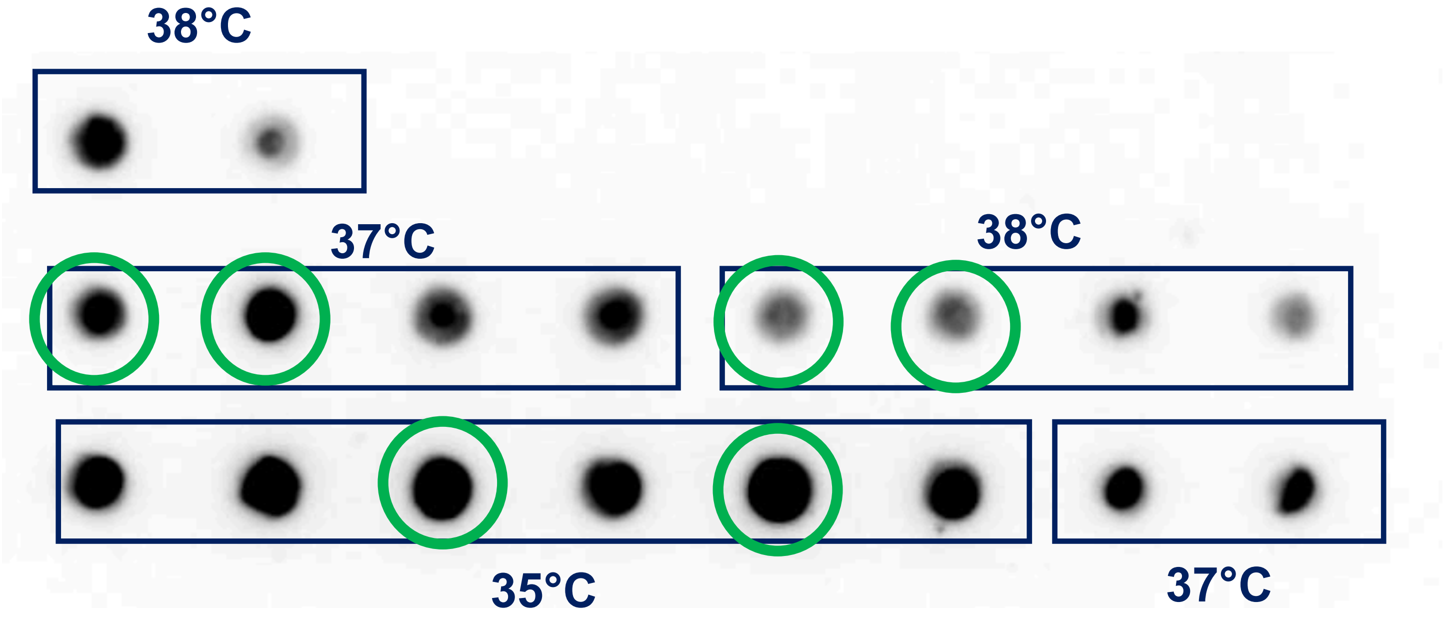

Figure 2F

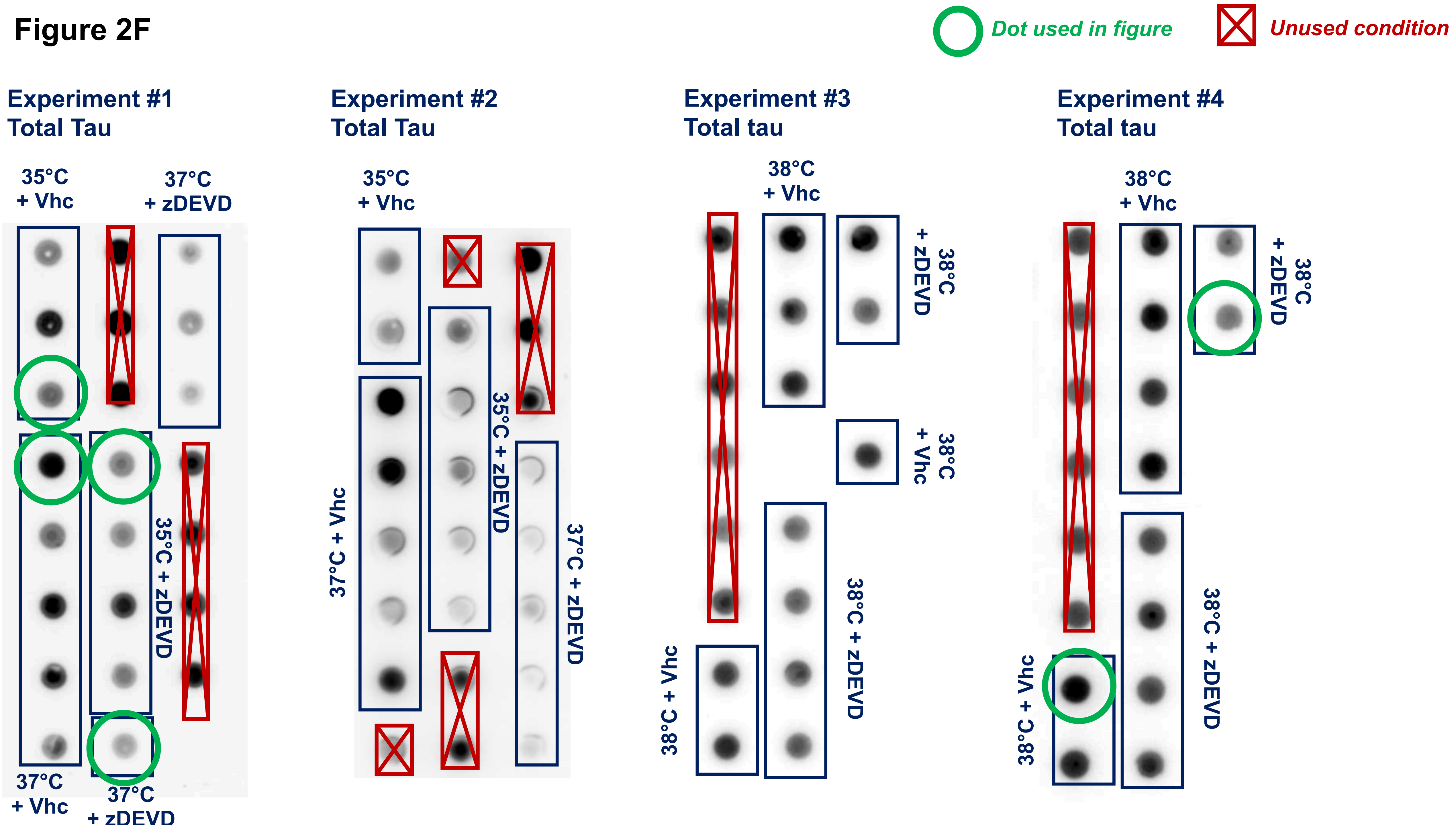

Dot used in figure
Unused condition
Experiment #1
Total Tau
35°C + Vhc
37°C + zDEVD
35°C + zDEVD
37°C + Vhc
37°C + zDEVD
Experiment #2
Total Tau
35°C + Vhc
35°C + zDEVD
37°C + Vhc
37°C + zDEVD
Experiment #3
Total tau
38°C + Vhc
38°C + zDEVD
38°C + Vhc
38°C + Vhc
38°C + zDEVD
Experiment #4
Total tau
38°C + Vhc
38°C + zDEVD
38°C + Vhc
38°C + zDEVD

Figure 2F

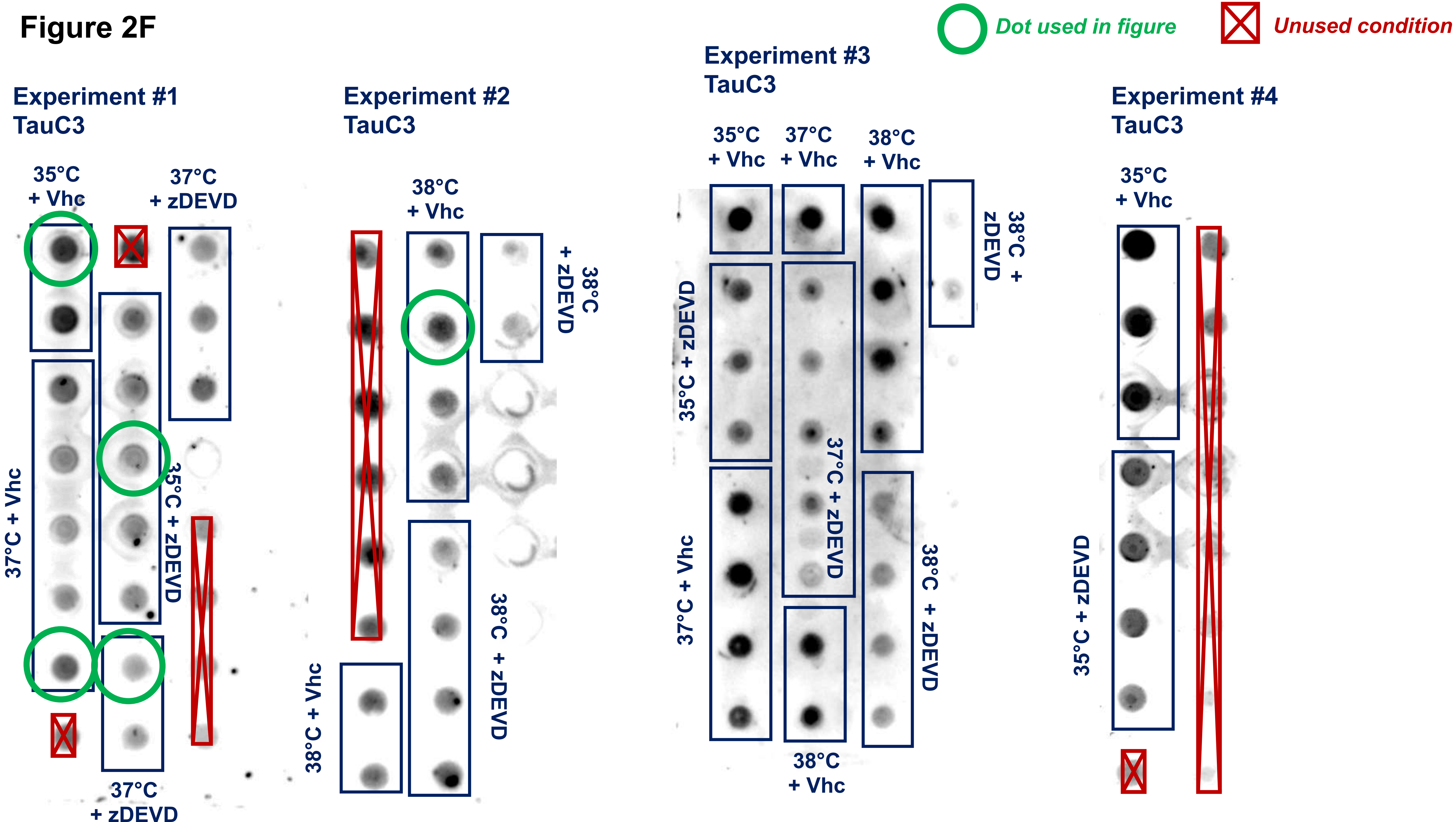
Dot used in figure
Unused condition
Experiment #1
TauC3
35°C
+ Vhc
37°C
+ zDEVD
37°C + Vhc
35°C + zDEVD
37°C
+ zDEVD
Experiment #2
TauC3
38°C
+ Vhc
38°C
+ zDEVD
38°C + zDEVD
38°C + Vhc
Experiment #3
TauC3
35°C
+ Vhc
37°C
+ Vhc
38°C
+ Vhc
38°C +
zDEVD
35°C + zDEVD
37°C + zDEVD
37°C + Vhc
38°C + zDEVD
38°C
+ Vhc
Experiment #4
TauC3
35°C
+ Vhc
35°C + zDEVD

Figure 2H

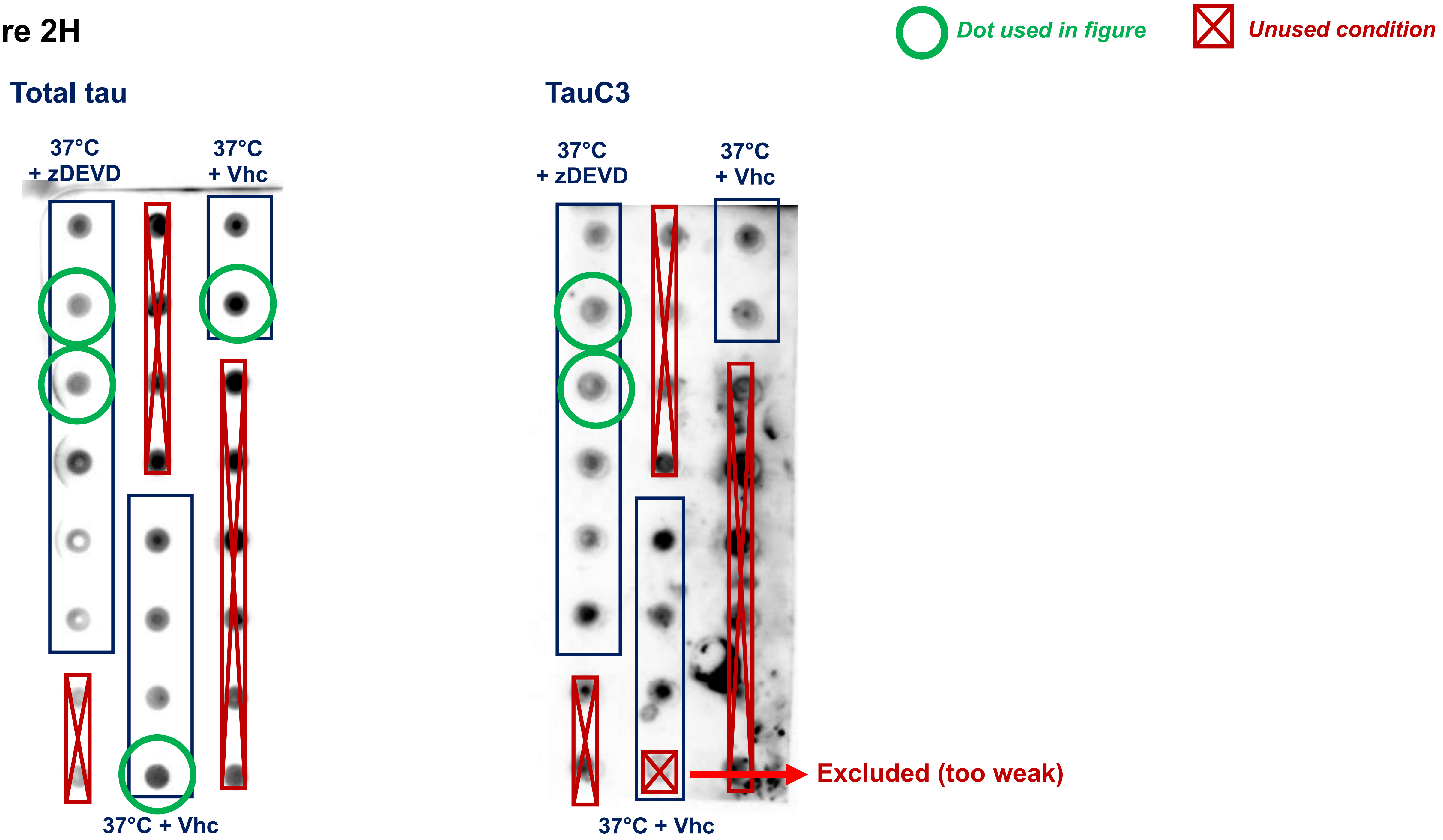
Dot used in figure
Unused condition
Total tau
37°C
+ zDEVD
37°C
+ Vhc
37°C + Vhc
TauC3
37°C
+ zDEVD
37°C
+ Vhc
37°C + Vhc
Excluded (too weak)

Figure 2I

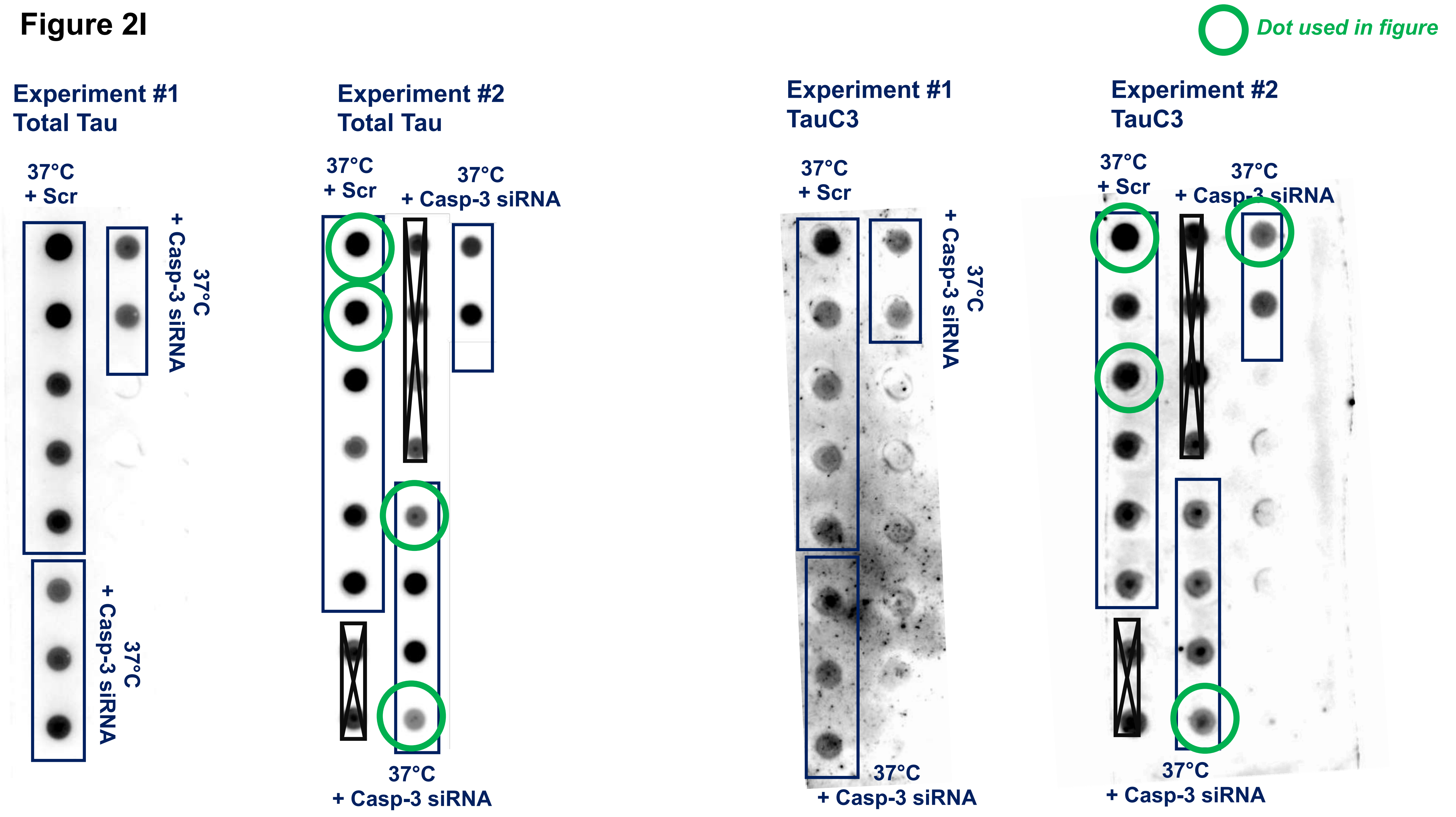
Dot used in figure
Experiment #1
Total Tau
37°C
+ Scr
37°C
+ Casp-3 siRNA
37°C
+ Casp-3 siRNA
Experiment #2
Total Tau
37°C
+ Scr
37°C
+ Casp-3 siRNA
37°C
+ Casp-3 siRNA
Experiment #1
TauC3
37°C
+ Scr
37°C
+ Casp-3 siRNA
37°C
+ Casp-3 siRNA
Experiment #2
TauC3
37°C
+ Scr
37°C
+ Casp-3 siRNA
37°C
+ Casp-3 siRNA

FIGURE 3

Figure 3A,B

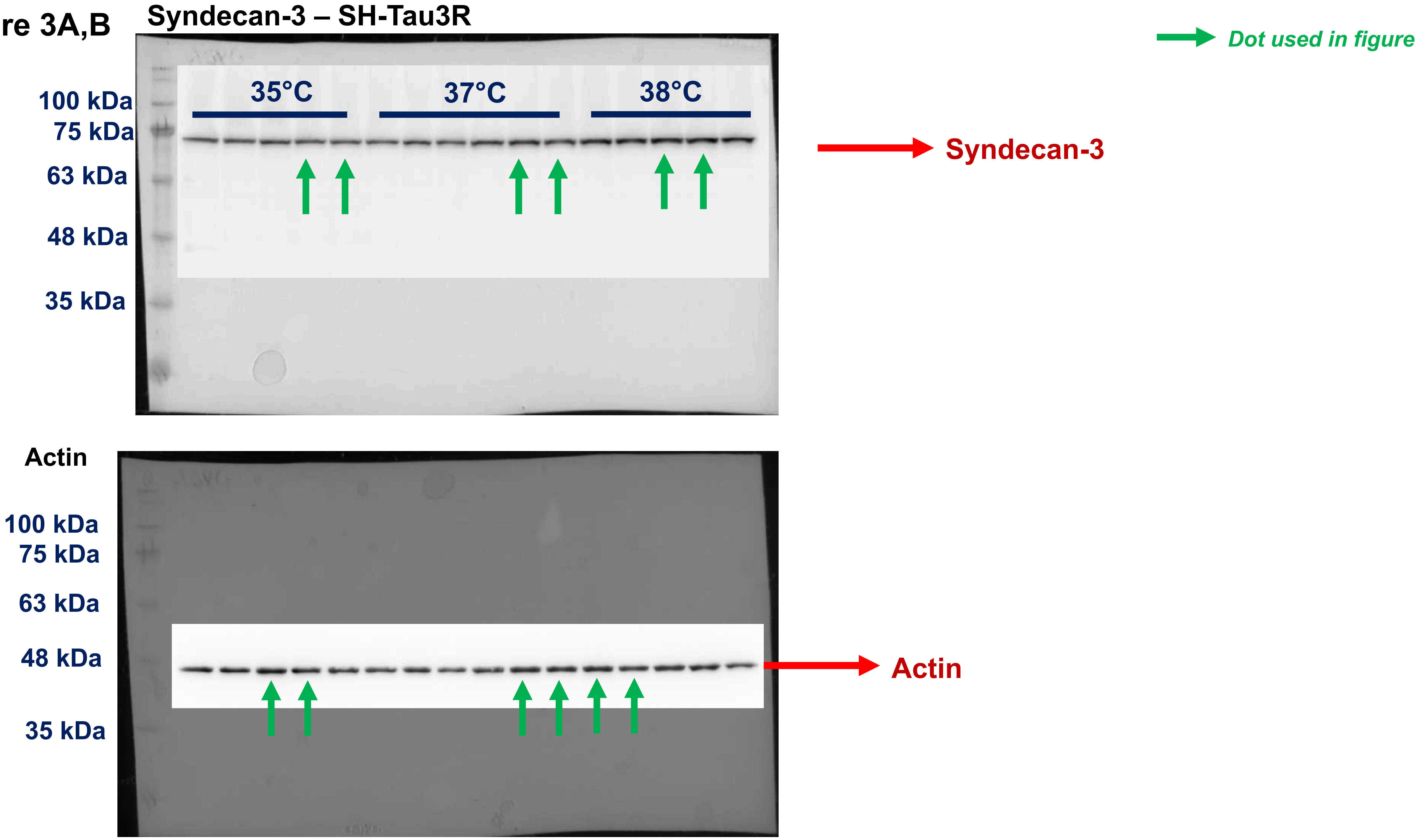

Syndecan-3 – SH-Tau3R
Dot used in figure
100 kDa
75 kDa
63 kDa
48 kDa
35 kDa
35°C
37°C
38°C
Syndecan-3
Actin
100 kDa
75 kDa
63 kDa
48 kDa
35 kDa
Actin

Figure 3A,B

Dot used in figure

PIP2 – SH-Tau3R

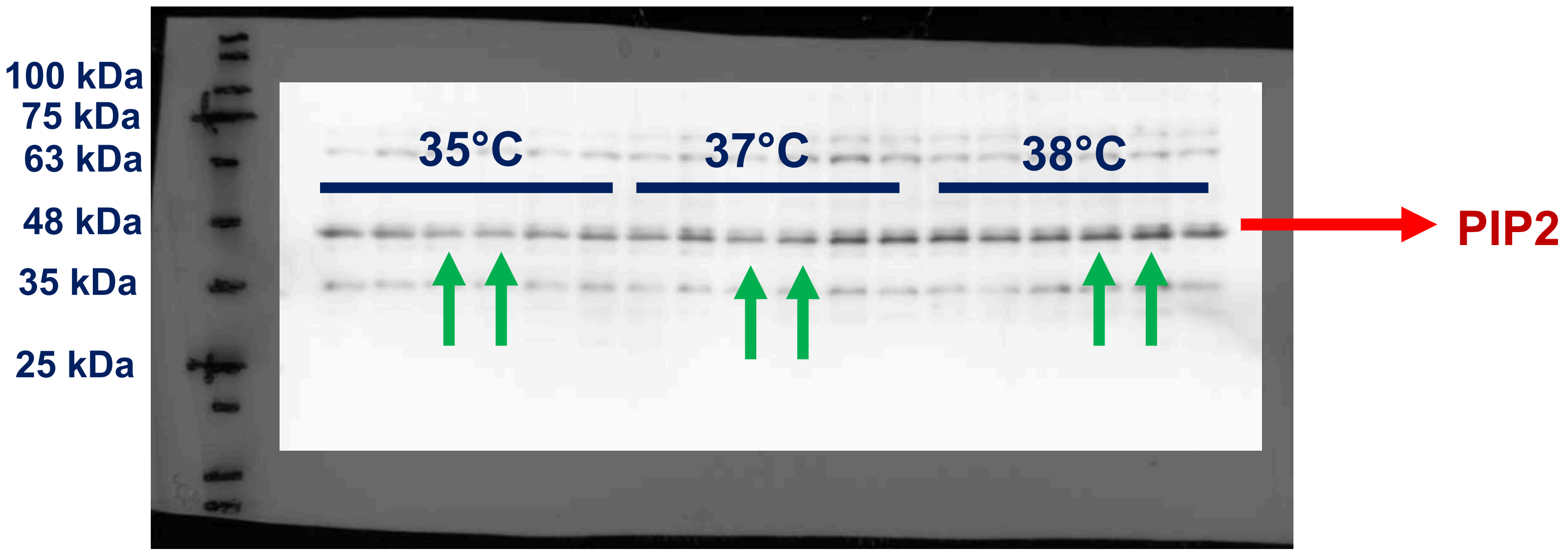

Actin

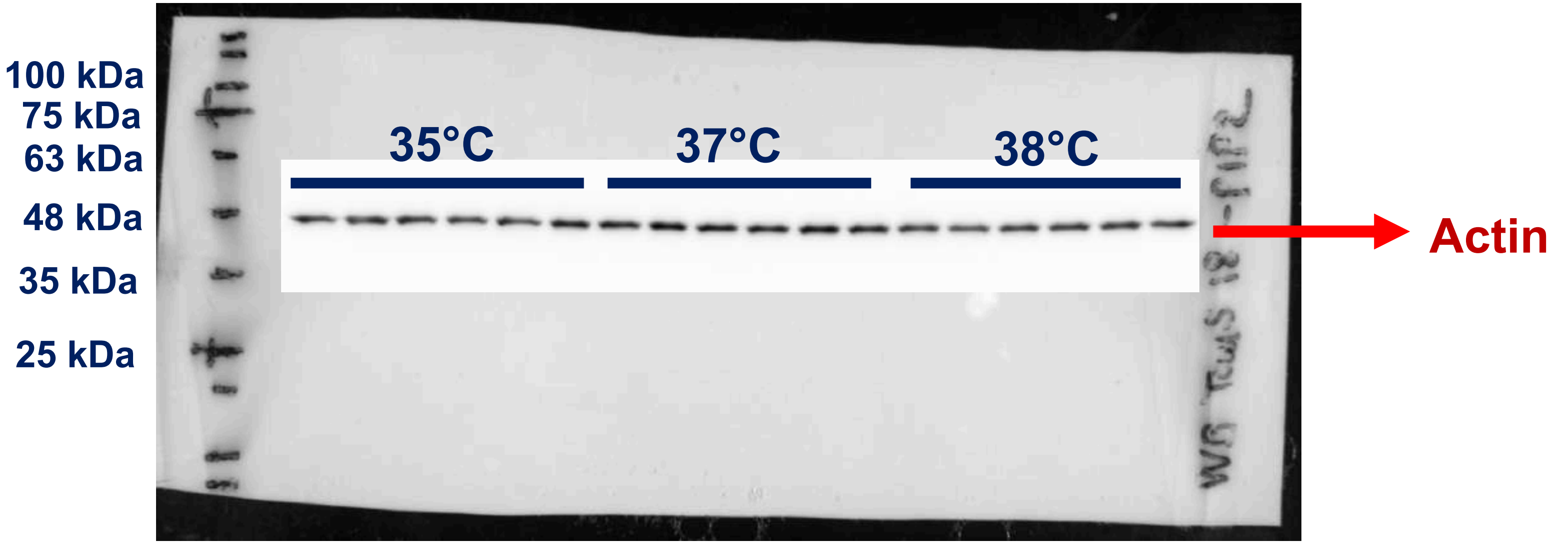

Figure 3A,B

Syndecan-3 – Primary neurons

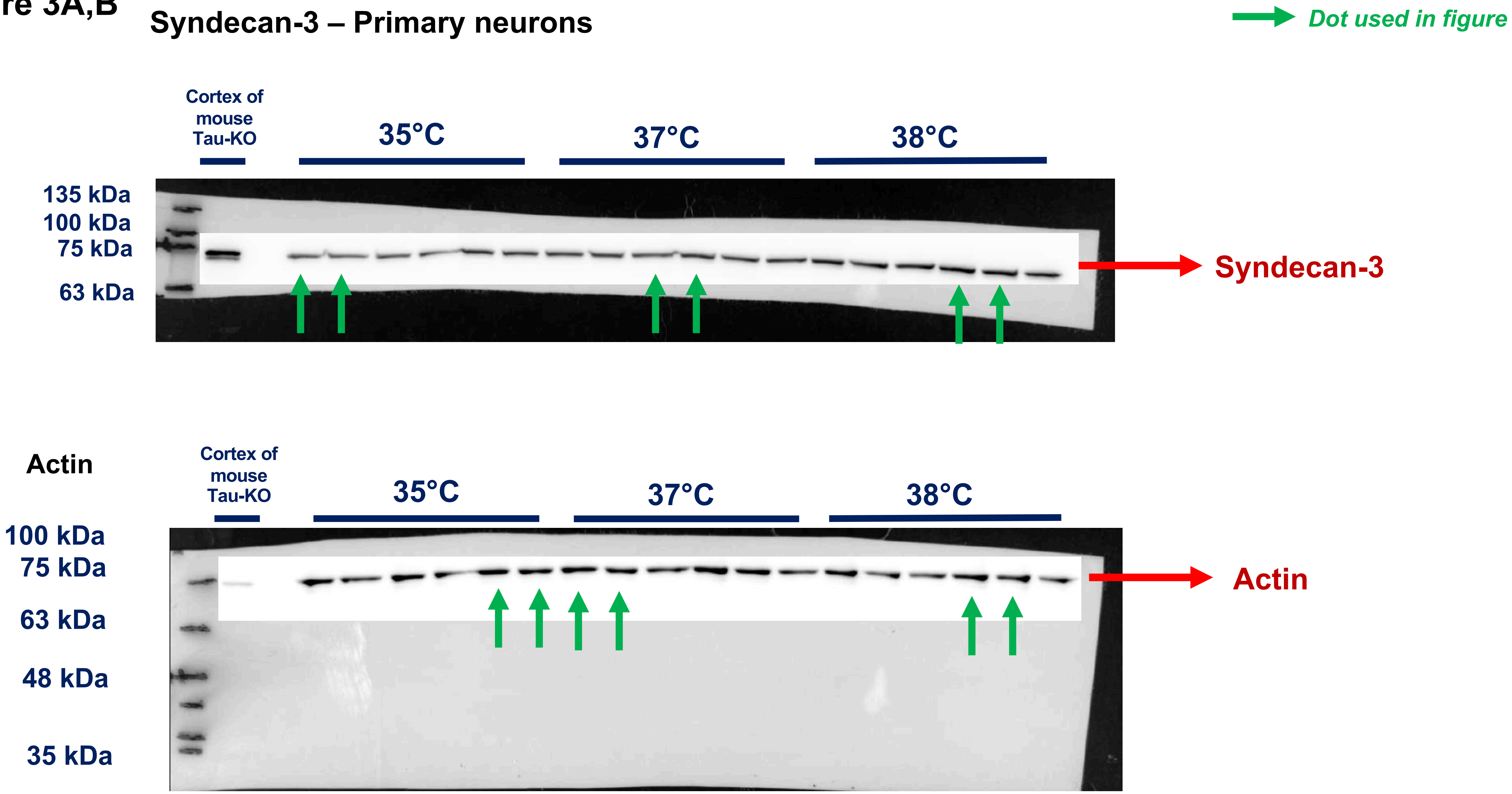

Figure 3A,B

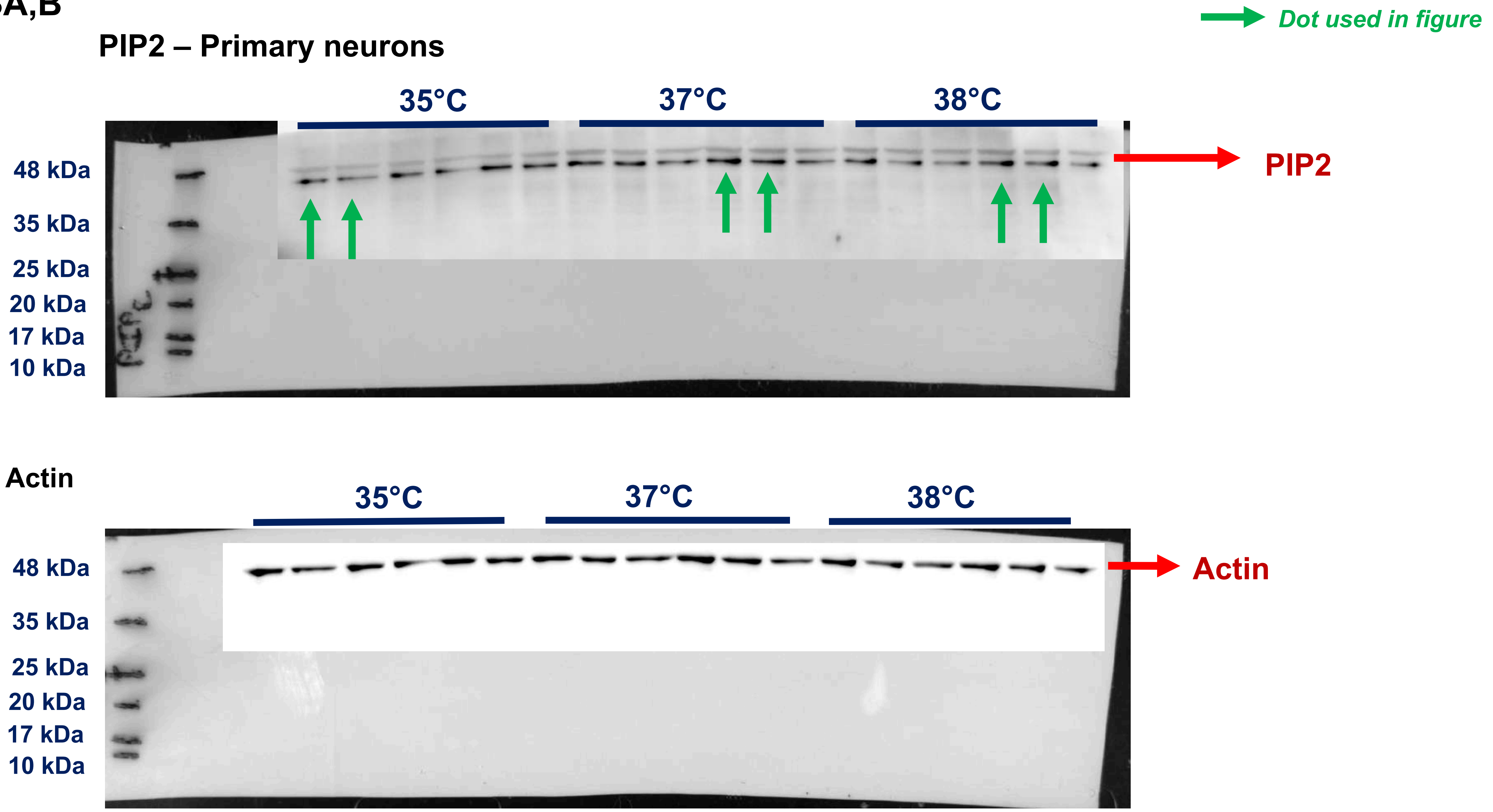
Dot used in figure
PIP2 – Primary neurons
35°C
37°C
38°C
48 kDa
35 kDa
25 kDa
20 kDa
17 kDa
10 kDa
PIP2
Actin
35°C
37°C
38°C
48 kDa
35 kDa
25 kDa
20 kDa
17 kDa
10 kDa
Actin

FIGURE 4

Figure 4A

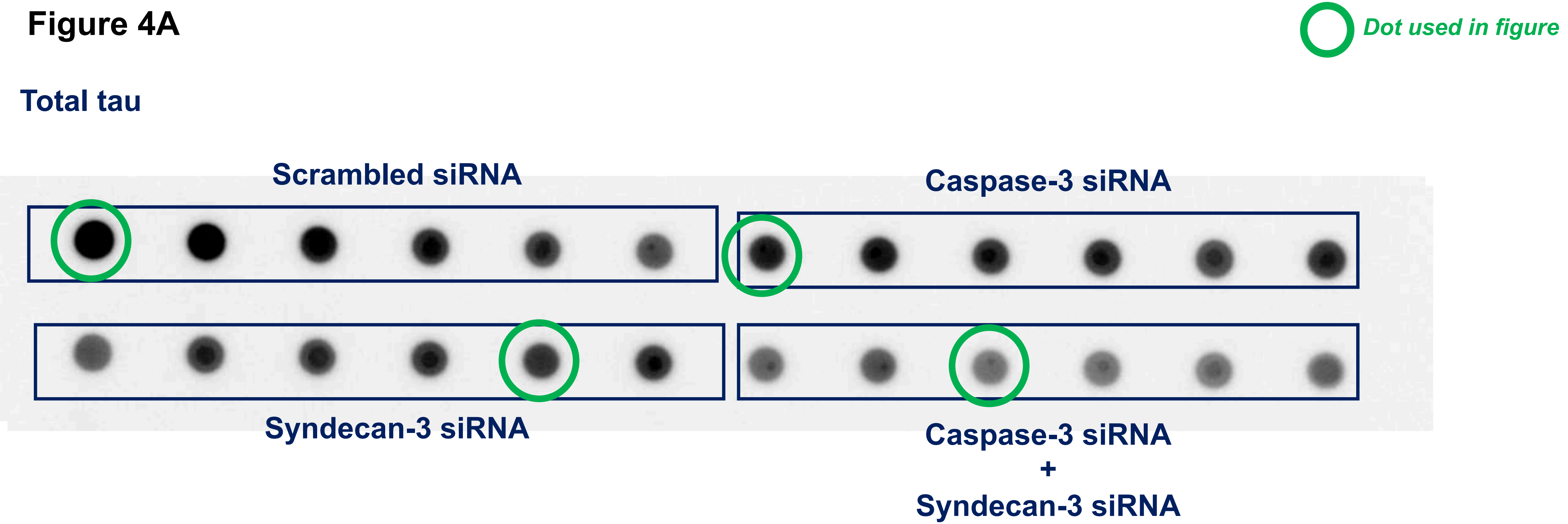
Dot used in figure
Total tau
Scrambled siRNA
Caspase-3 siRNA
Syndecan-3 siRNA
Caspase-3 siRNA
+
Syndecan-3 siRNA

Figure 4B

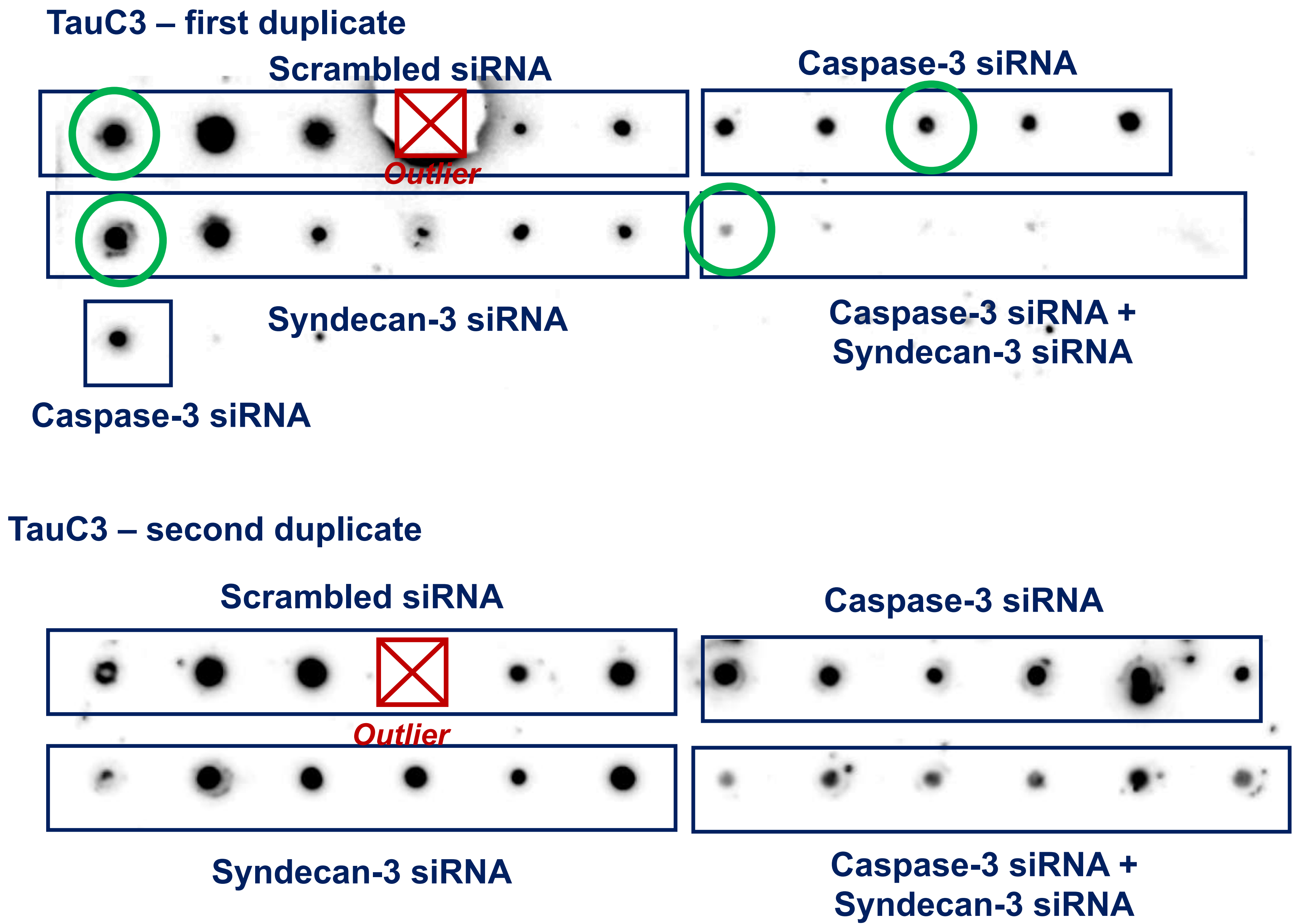
Dot used in figure
TauC3 – first duplicate
Scrambled siRNA
Outlier
Caspase-3 siRNA
Syndecan-3 siRNA
Caspase-3 siRNA
Caspase-3 siRNA +
Syndecan-3 siRNA
TauC3 – second duplicate
Scrambled siRNA
Outlier
Caspase-3 siRNA
Syndecan-3 siRNA
Caspase-3 siRNA +
Syndecan-3 siRNA

Figure 4C

Dot used in figure

Total tau

Experiment #1

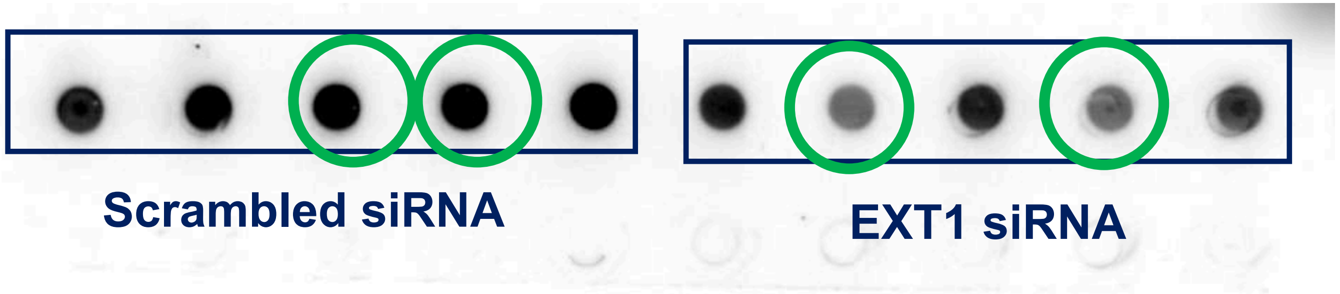

Experiment #2

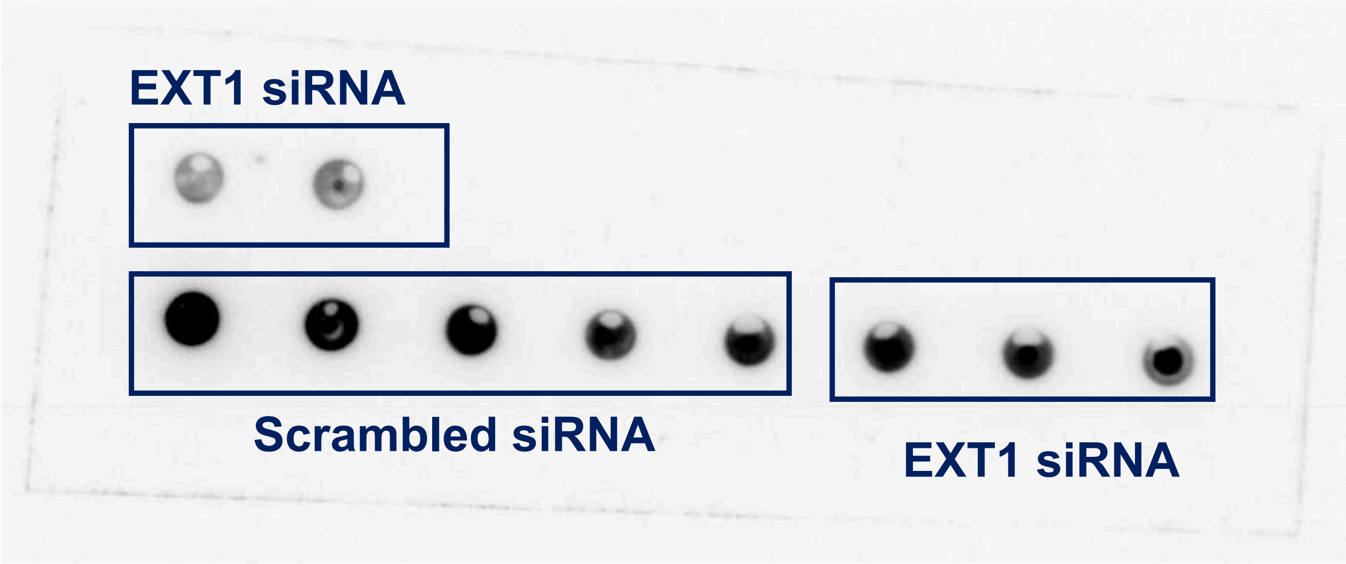

Figure 4C

Dot used in figure

TauC3

Experiment #1

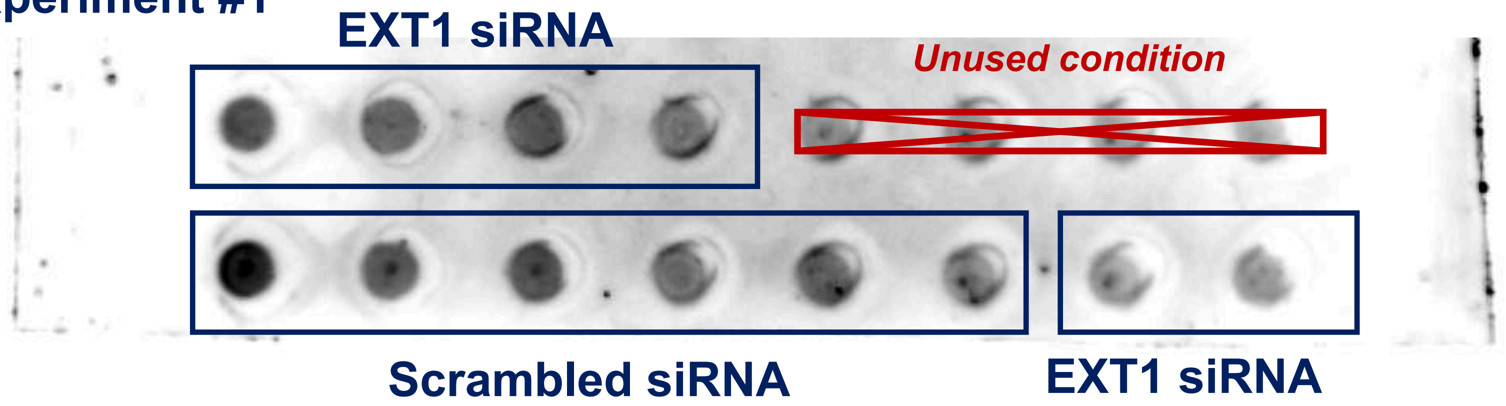

Experiment #2

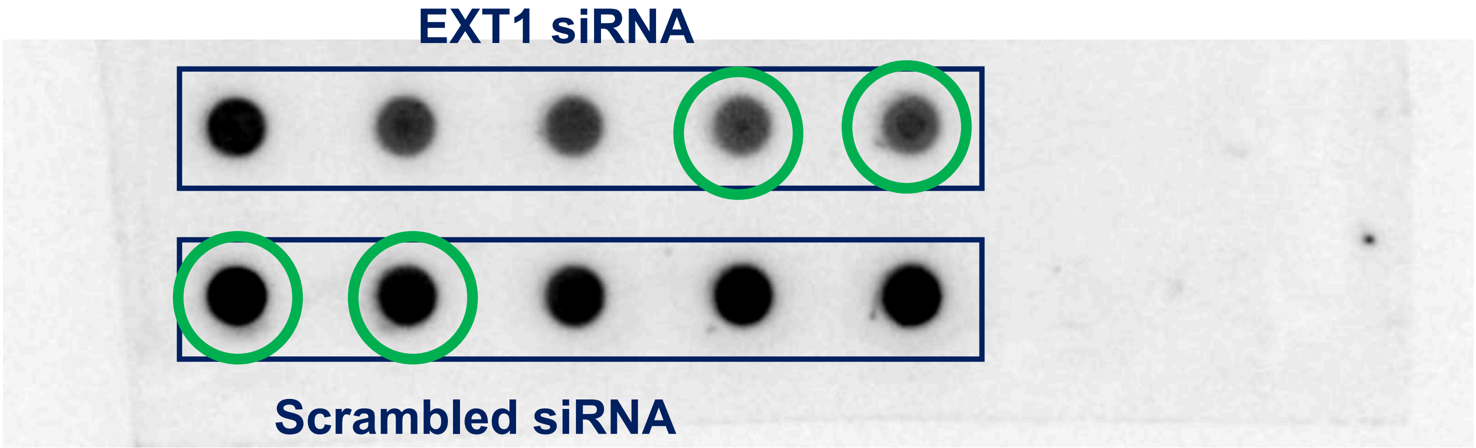

Figure 4F

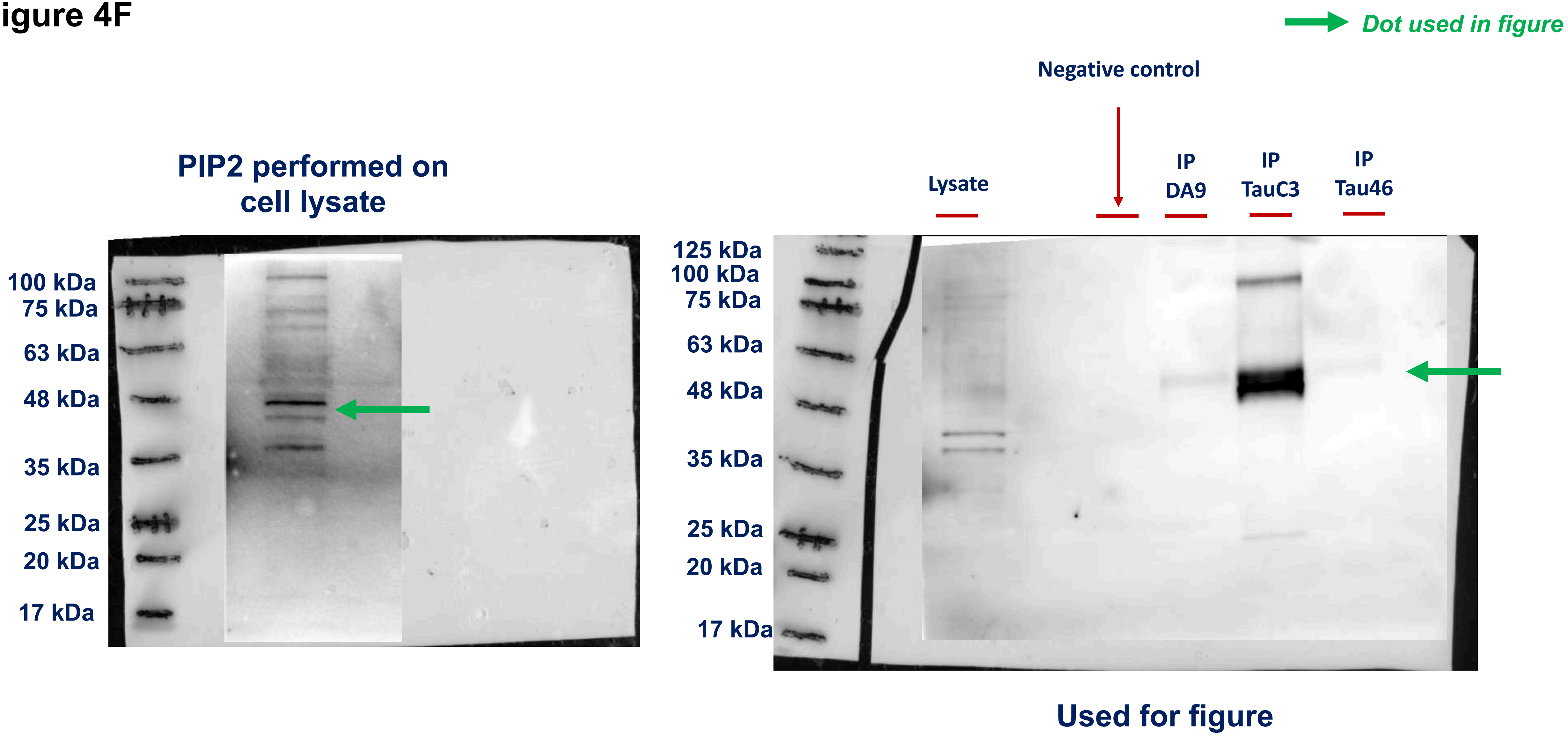

Dot used in figure
PIP2 performed on cell lysate
100 kDa
75 kDa
63 kDa
48 kDa
35 kDa
25 kDa
20 kDa
17 kDa
Negative control
Lysate
IP DA9
IP TauC3
IP Tau46
125 kDa
100 kDa
75 kDa
63 kDa
48 kDa
35 kDa
25 kDa
20 kDa
17 kDa
Used for figure

Figure 4G

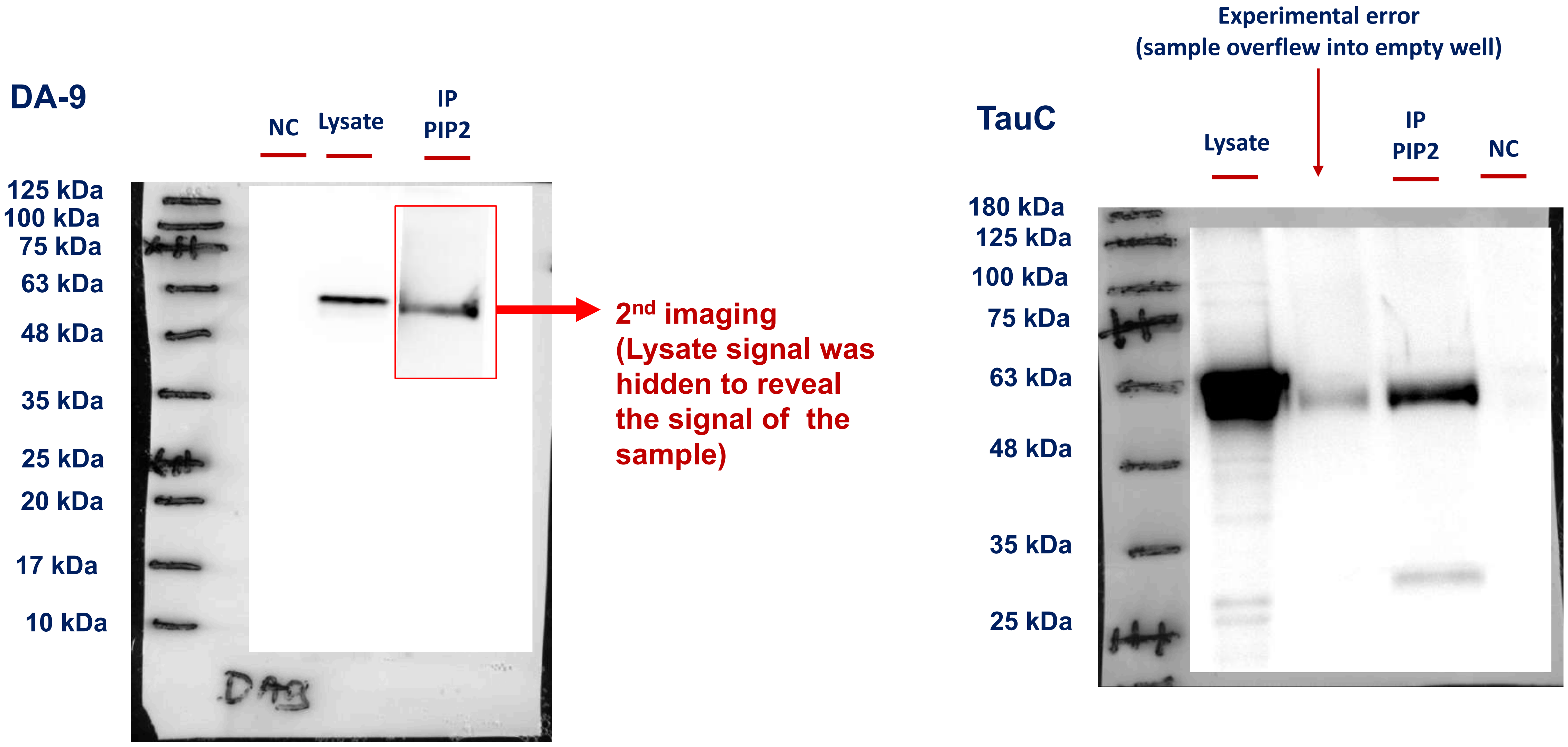
DA-9
NC
Lysate
IP
PIP2
125 kDa
100 kDa
75 kDa
63 kDa
48 kDa
35 kDa
25 kDa
20 kDa
17 kDa
10 kDa
2nd imaging
(Lysate signal was hidden to reveal the signal of the sample)
Experimental error
(sample overflew into empty well)
TauC
Lysate
IP
PIP2
NC
180 kDa
125 kDa
100 kDa
75 kDa
63 kDa
48 kDa
35 kDa
25 kDa

Figure 4G

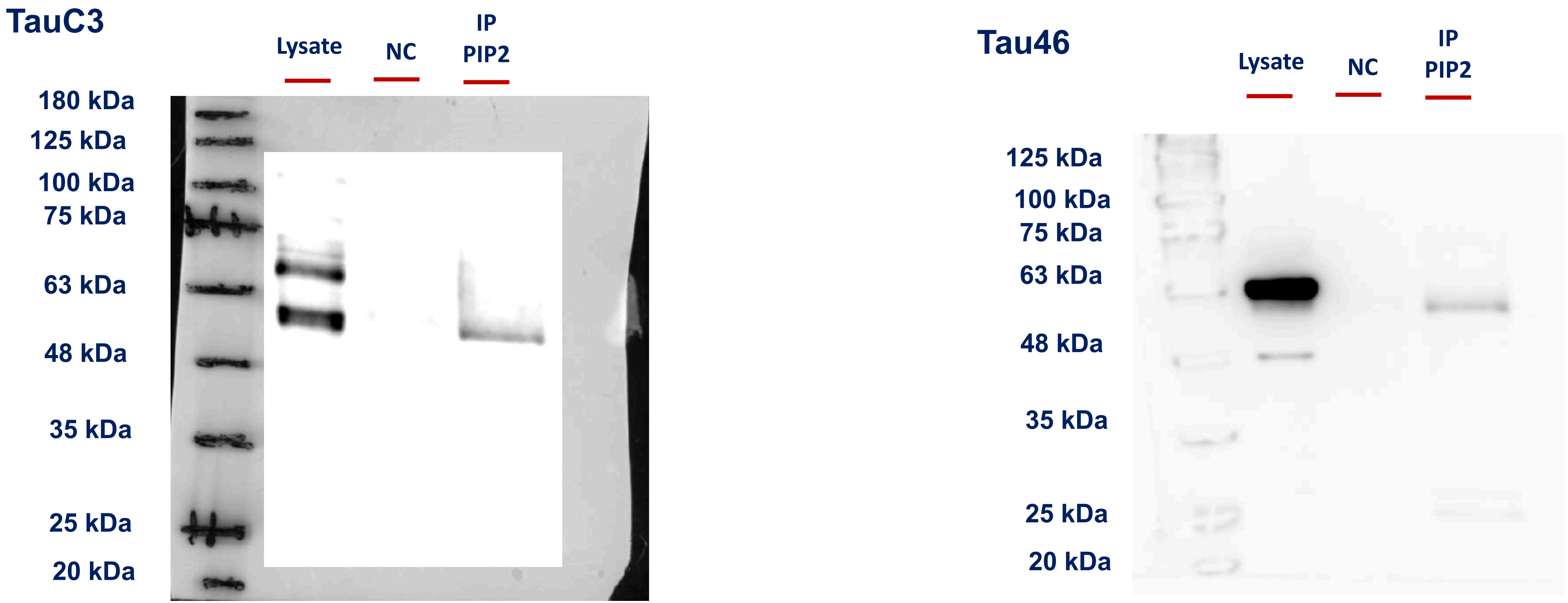
TauC3
Lysate
NC
IP
PIP2
180 kDa
125 kDa
100 kDa
75 kDa
63 kDa
48 kDa
35 kDa
25 kDa
20 kDa
Tau46
Lysate
NC
IP
PIP2
125 kDa
100 kDa
75 kDa
63 kDa
48 kDa
35 kDa
25 kDa
20 kDa

Figure 4G

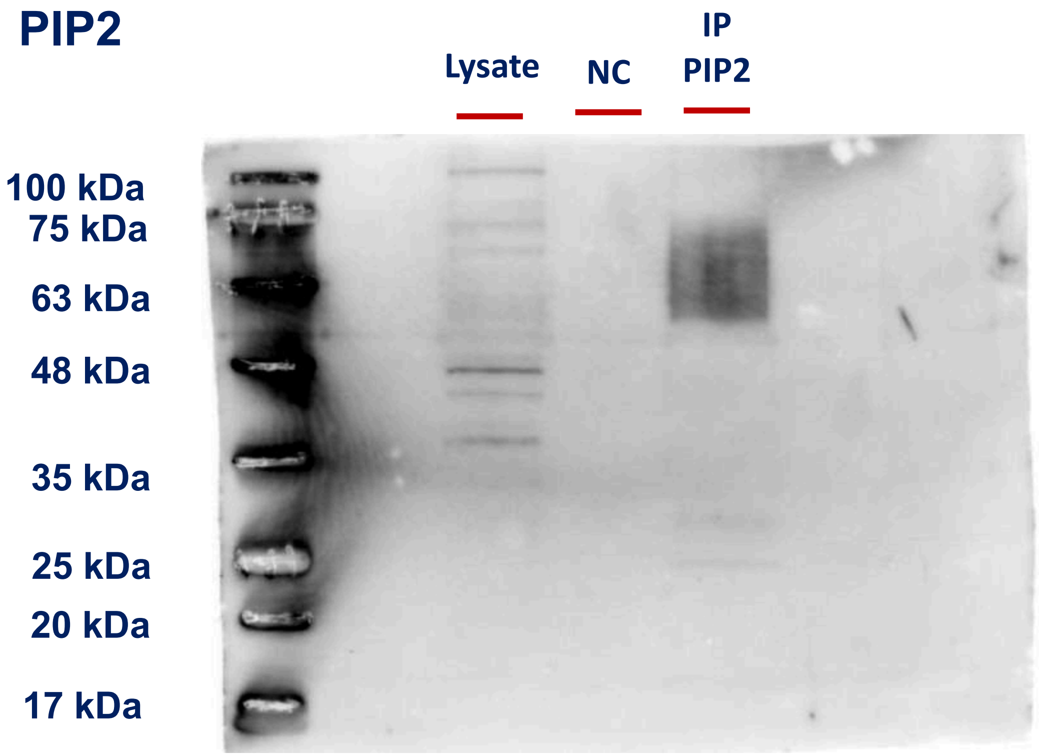

PIP2
Lysate
NC
IP
PIP2
100 kDa
75 kDa
63 kDa
48 kDa
35 kDa
25 kDa
20 kDa
17 kDa

Figure 4H

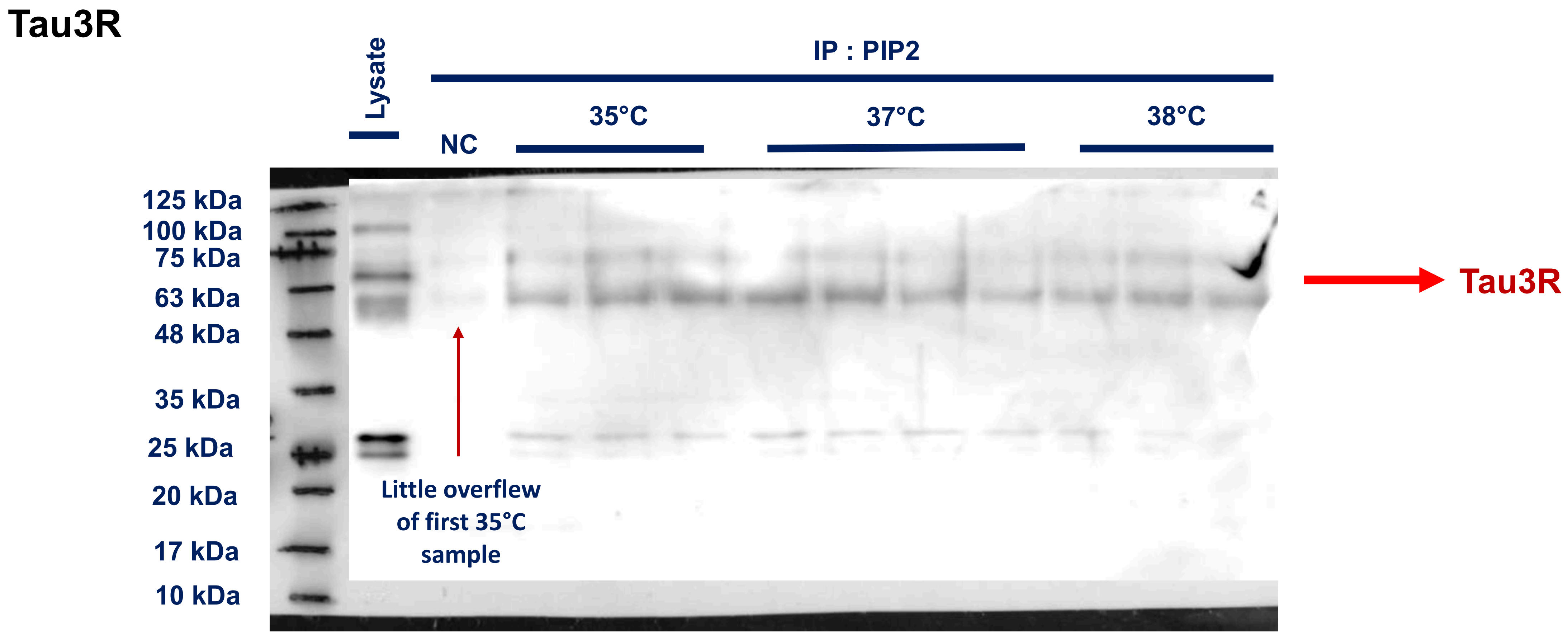

Tau3R
Lysate
IP : PIP2
35°C
37°C
38°C
NC
125 kDa
100 kDa
75 kDa
63 kDa
48 kDa
35 kDa
25 kDa
20 kDa
17 kDa
10 kDa
Little overflew
of first 35°C
sample
Tau3R

Figure 4H

TauC3

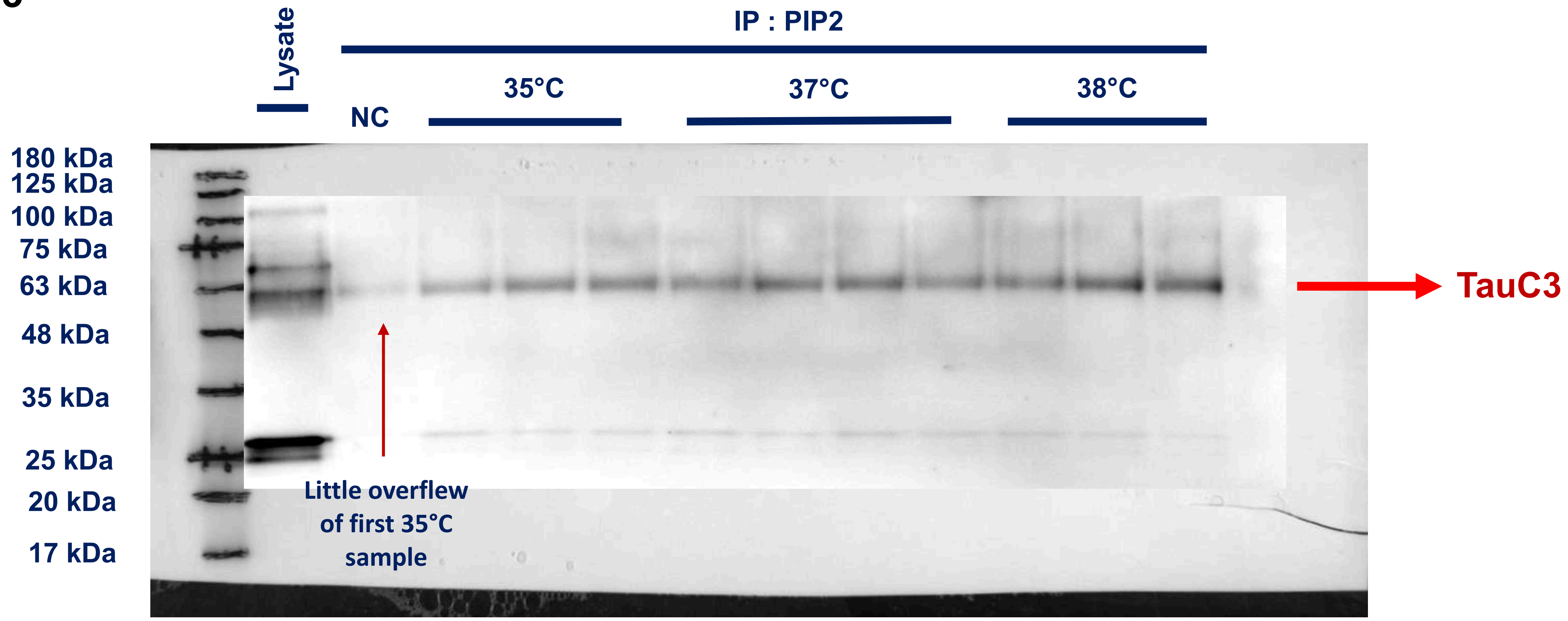

FIGURE 5

Figure 5C,D

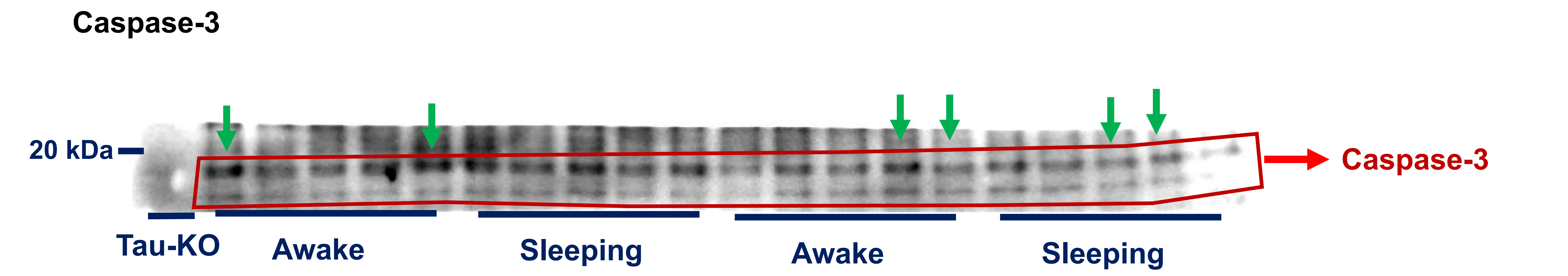
Dot used in figure
Caspase-3
20 kDa
Caspase-3
Tau-KO
Awake
Sleeping
Awake
Sleeping

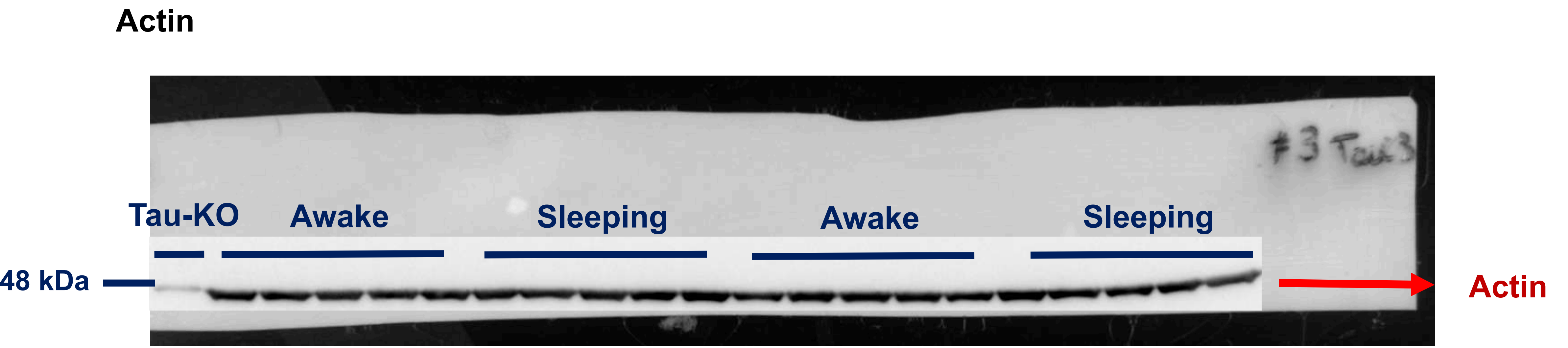
Actin
#3 Tau3
Tau-KO
Awake
Sleeping
Awake
Sleeping
48 kDa
Actin

Figure 5C,D

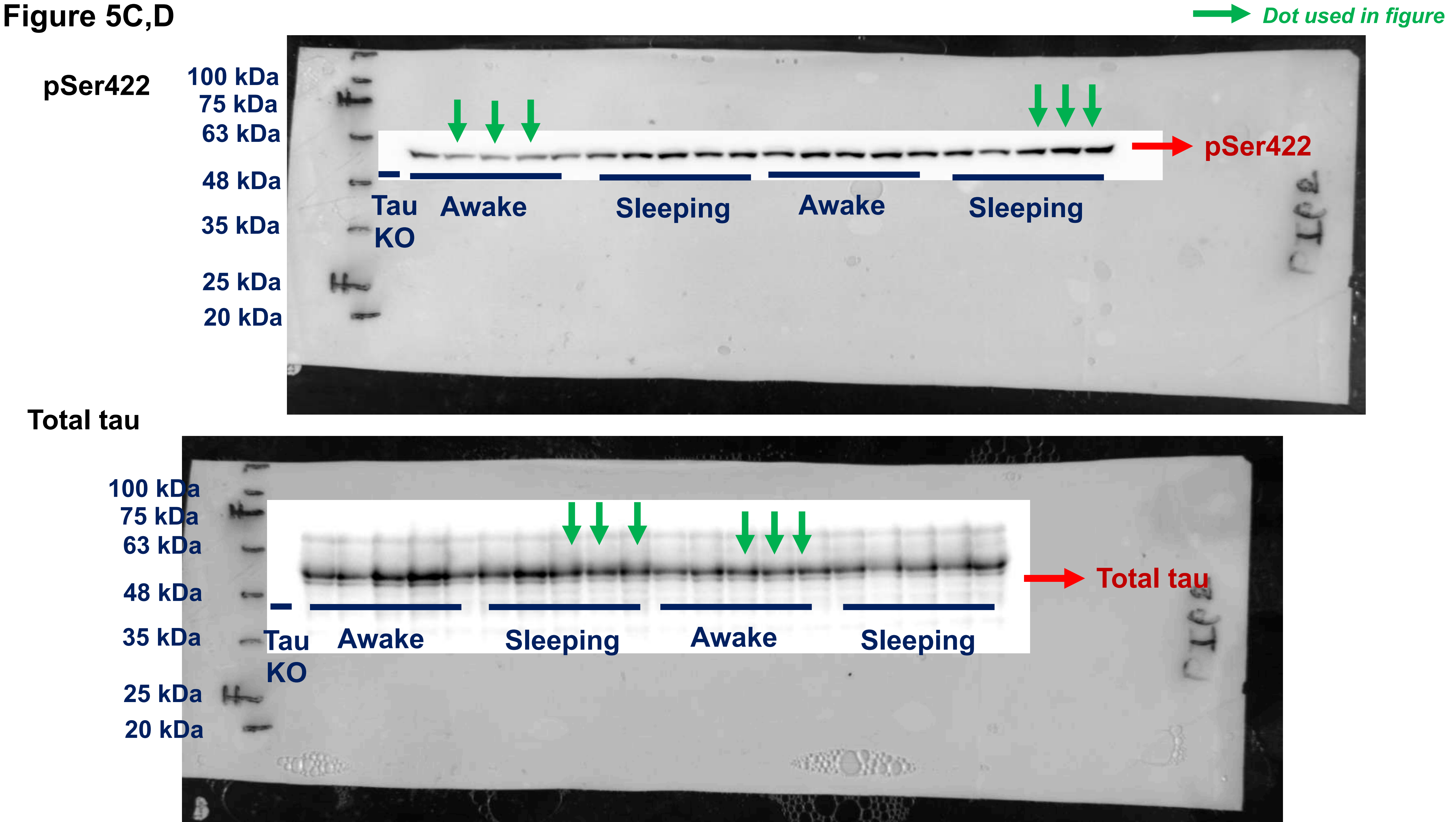

Dot used in figure
pSer422
100 kDa
75 kDa
63 kDa
48 kDa
35 kDa
25 kDa
20 kDa
Tau KO
Awake
Sleeping
Awake
Sleeping
pSer422
Total tau
100 kDa
75 kDa
63 kDa
48 kDa
35 kDa
25 kDa
20 kDa
Tau KO
Awake
Sleeping
Awake
Sleeping
Total tau

Figure 5C,D

Dot used in figure

TauC3

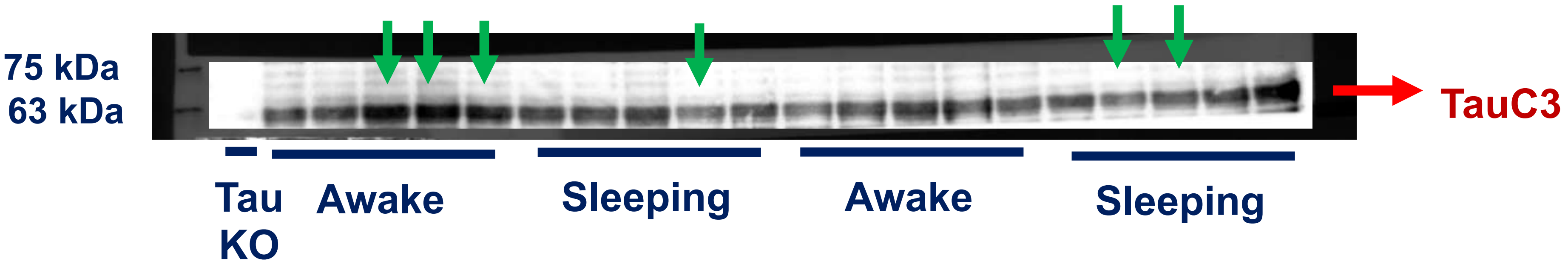

Actin

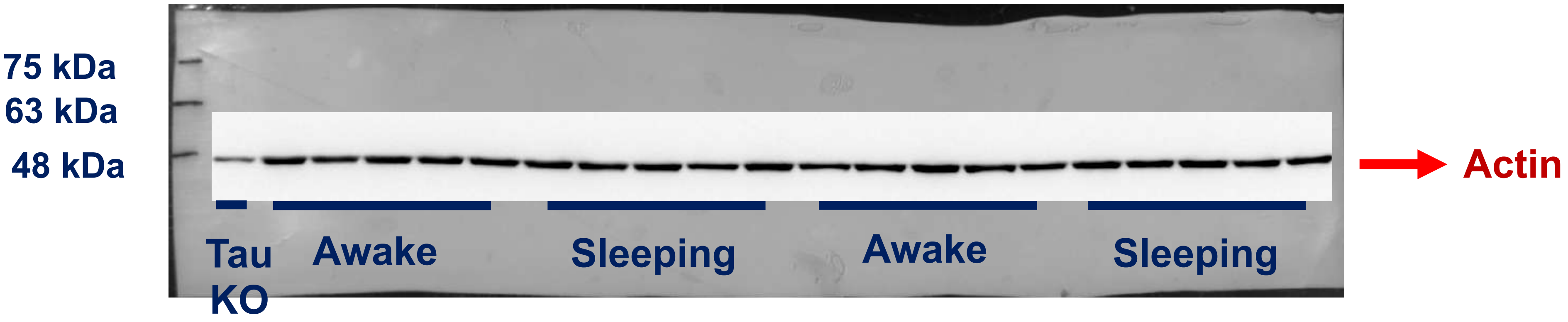

Figure 5C,D

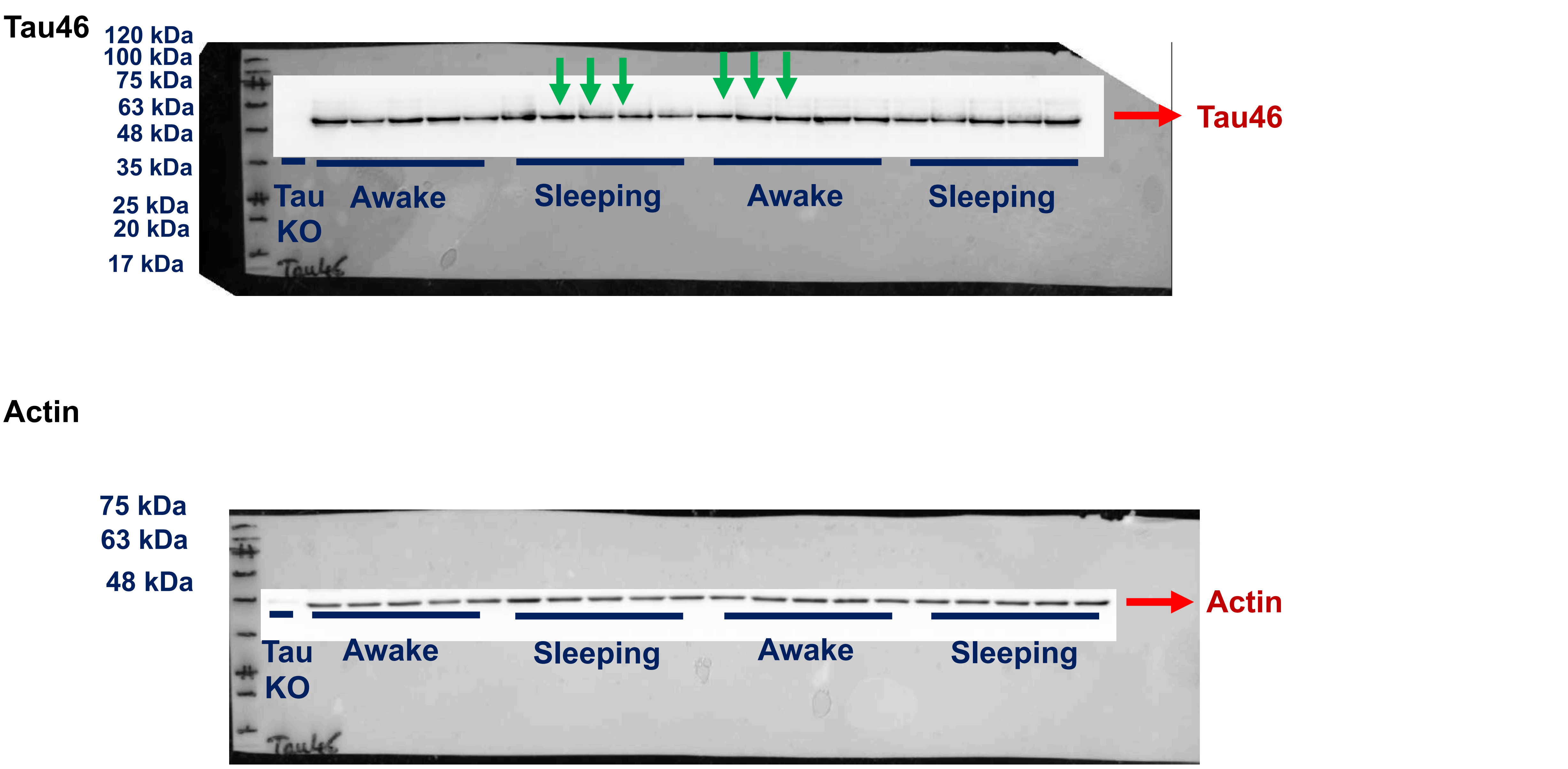
Dot used in figure
Tau46
120 kDa
100 kDa
75 kDa
63 kDa
48 kDa
35 kDa
25 kDa
20 kDa
17 kDa
Tau KO
Awake
Sleeping
Awake
Sleeping
Tau46
Actin
75 kDa
63 kDa
48 kDa
Tau KO
Awake
Sleeping
Awake
Sleeping
Actin

Figure 5C,D

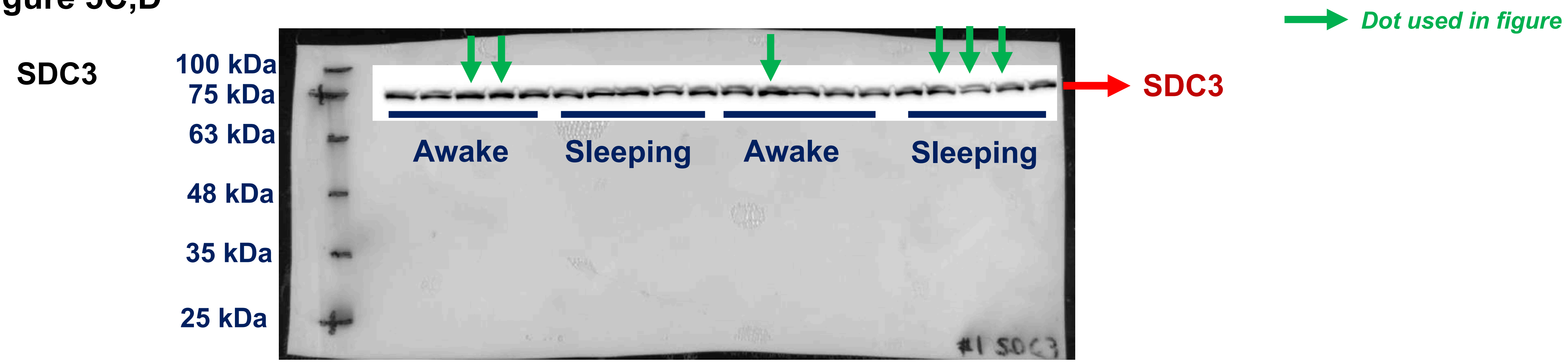

Dot used in figure
SDC3
100 kDa
75 kDa
63 kDa
48 kDa
35 kDa
25 kDa
Awake
Sleeping
Awake
Sleeping
SDC3
#1 SDC3

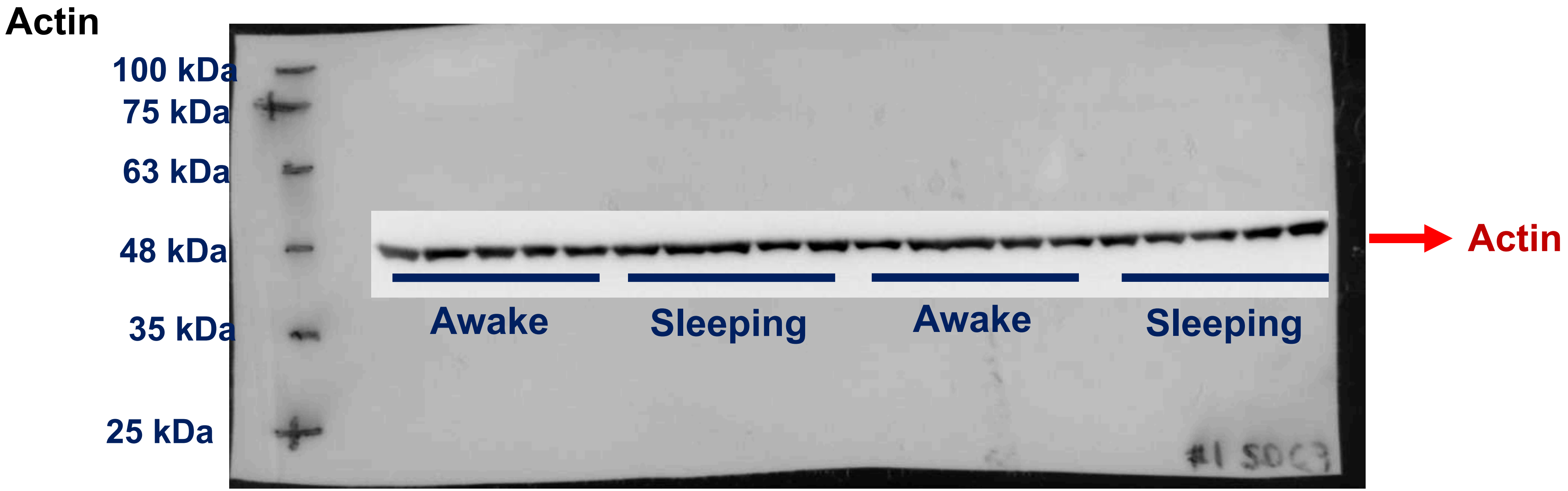

Actin
100 kDa
75 kDa
63 kDa
48 kDa
35 kDa
25 kDa
Awake
Sleeping
Awake
Sleeping
Actin
#1 SDC3

Figure 5C,D

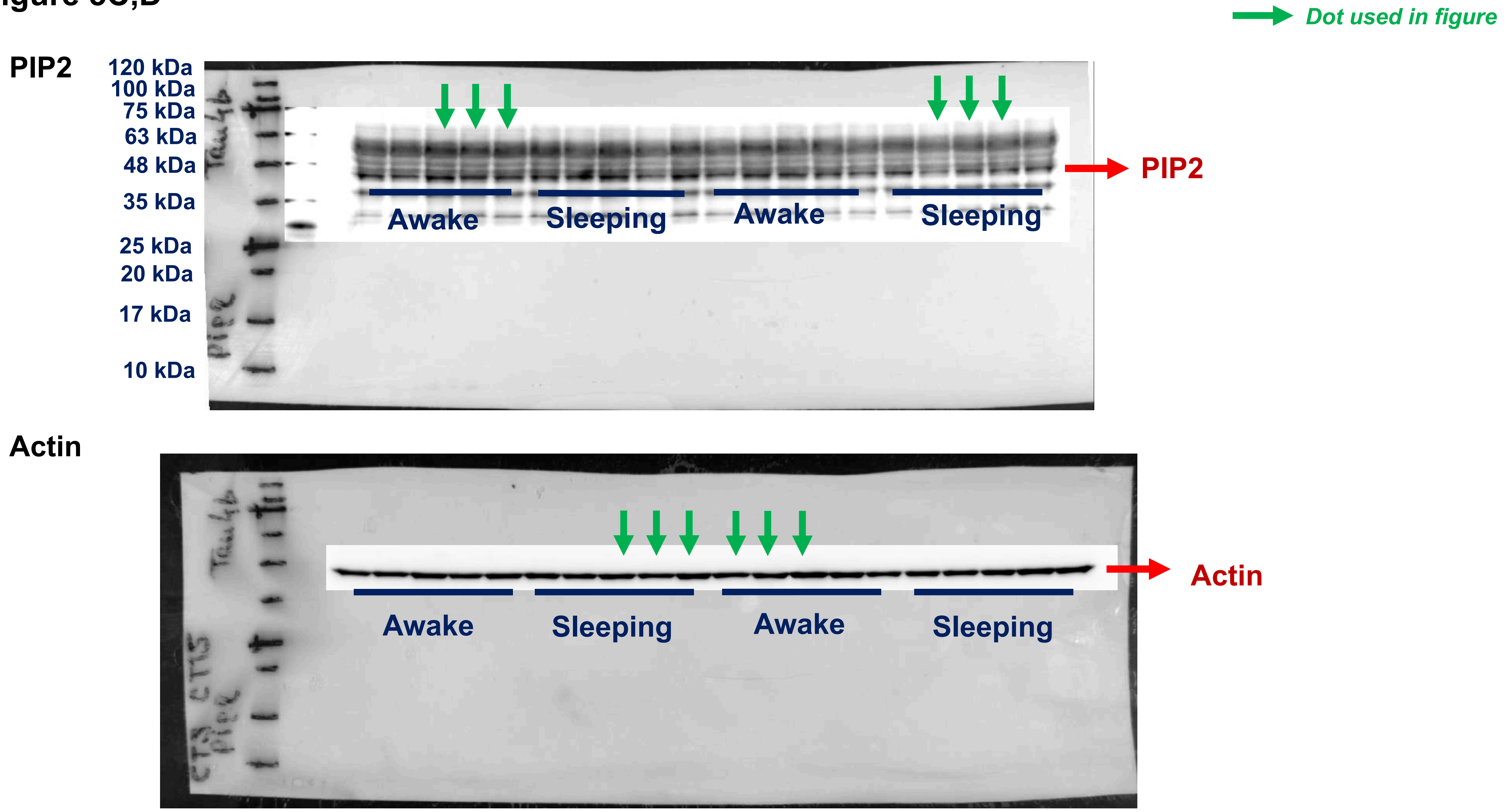

Dot used in figure
PIP2
120 kDa
100 kDa
75 kDa
63 kDa
48 kDa
35 kDa
25 kDa
20 kDa
17 kDa
10 kDa
Awake
Sleeping
Awake
Sleeping
PIP2
Actin
Awake
Sleeping
Awake
Sleeping
Actin

Figure 5G,H

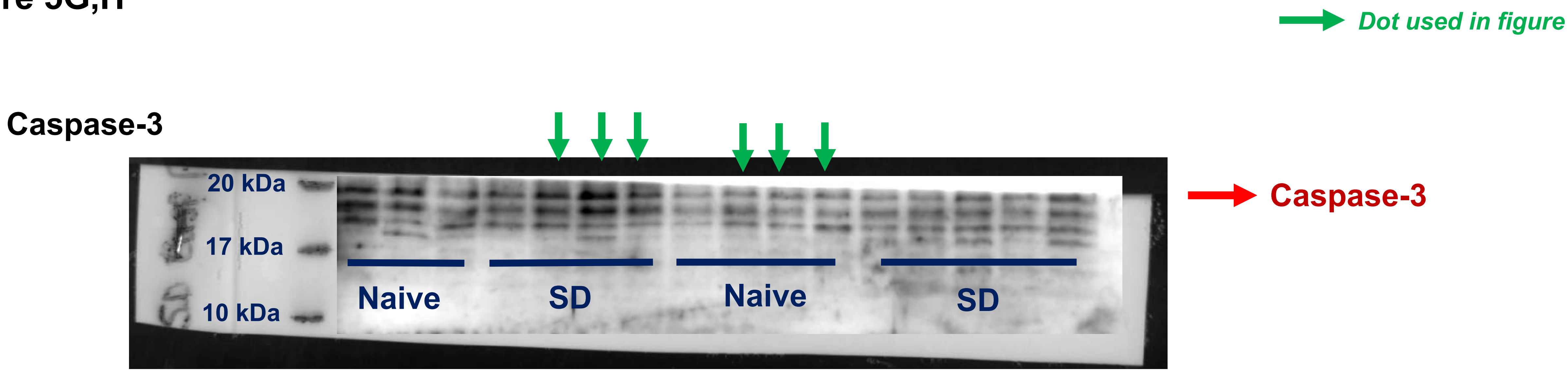

Dot used in figure
Caspase-3
20 kDa
17 kDa
10 kDa
Naive
SD
Naive
SD
Caspase-3

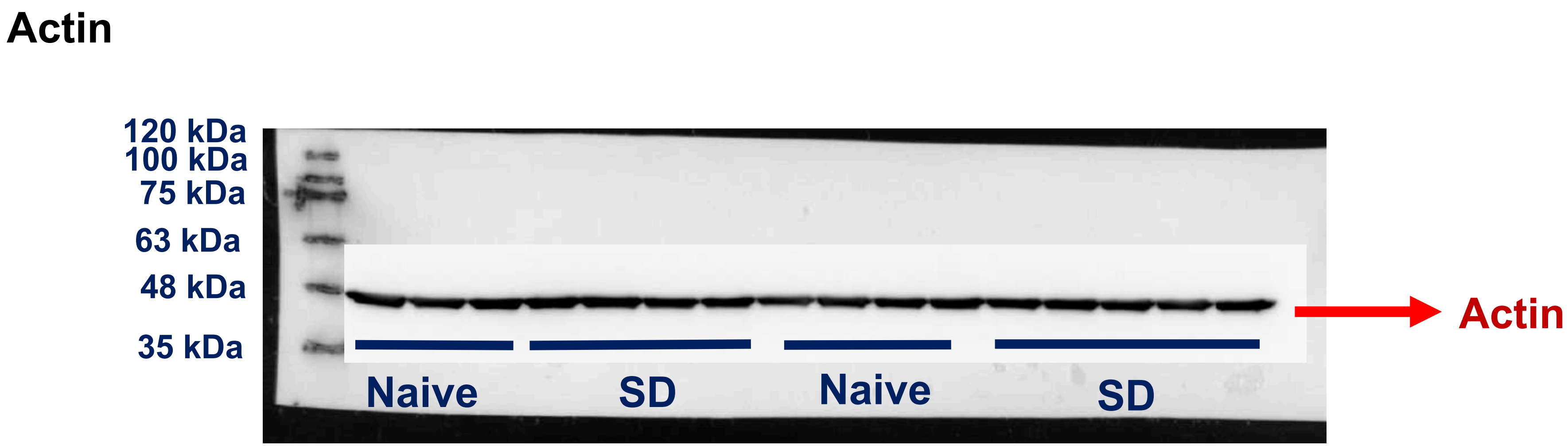

Actin
120 kDa
100 kDa
75 kDa
63 kDa
48 kDa
35 kDa
Naive
SD
Naive
SD
Actin

Figure 5G,H

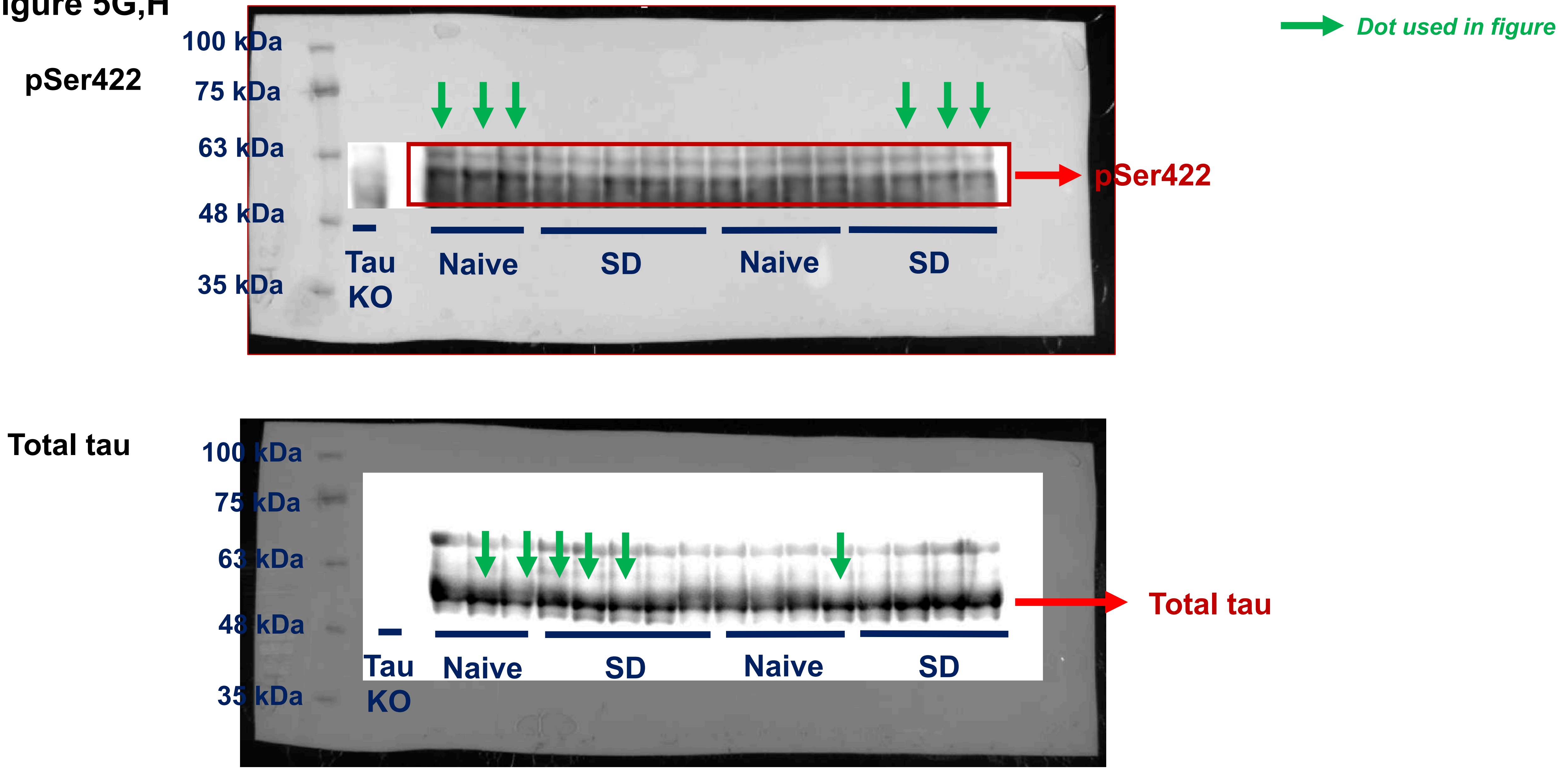

Dot used in figure
pSer422
100 kDa
75 kDa
63 kDa
48 kDa
35 kDa
Tau KO
Naive
SD
Naive
SD
pSer422
Total tau
100 kDa
75 kDa
63 kDa
48 kDa
35 kDa
Tau KO
Naive
SD
Naive
SD
Total tau

Figure 5 G,H

Dot used in figure

TauC3

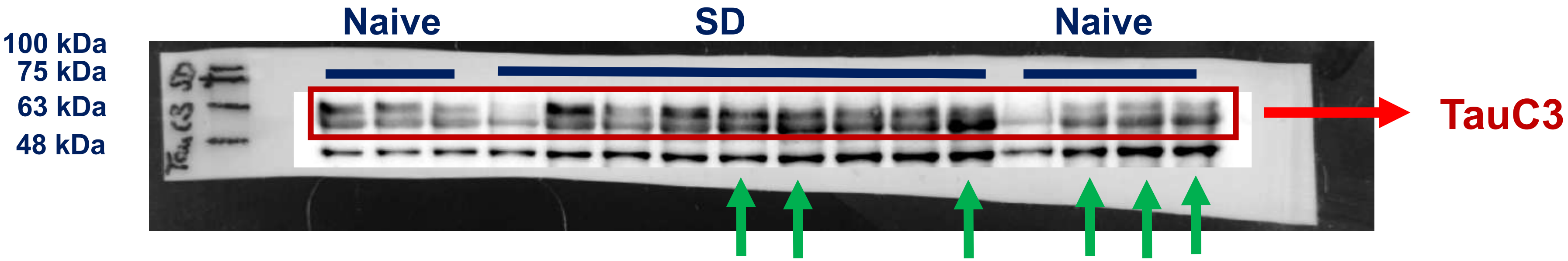

Actin

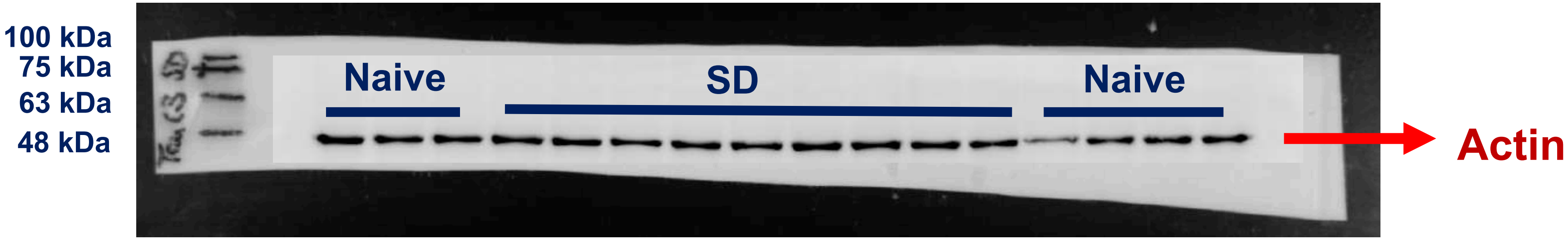

Figure 5 G,H

Dot used in figure

Tau46

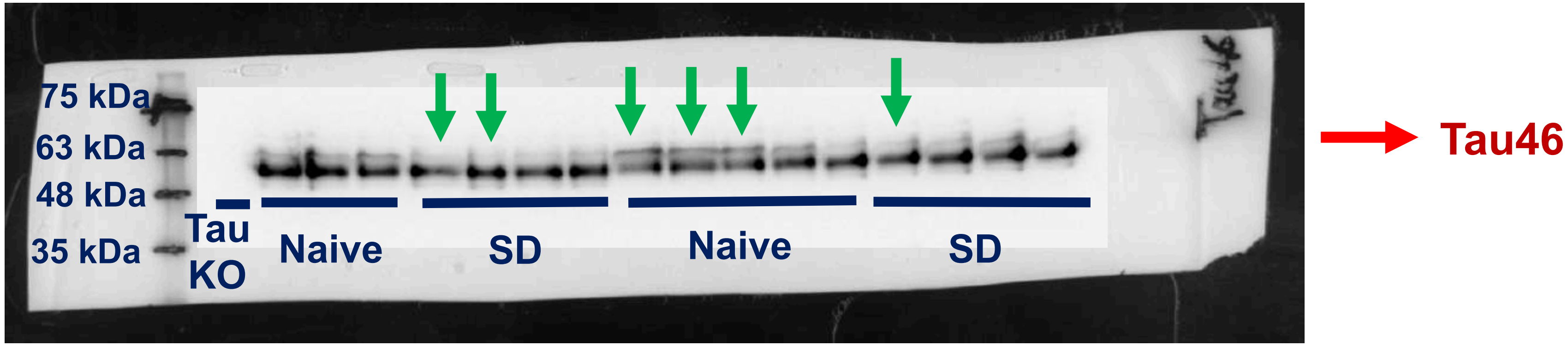

Actin

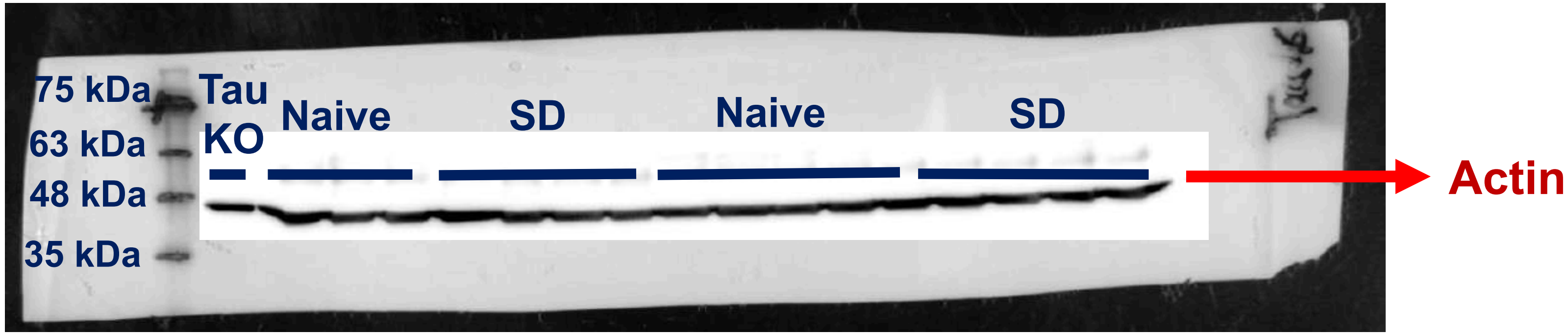

Figure 5 G,H

SDC3

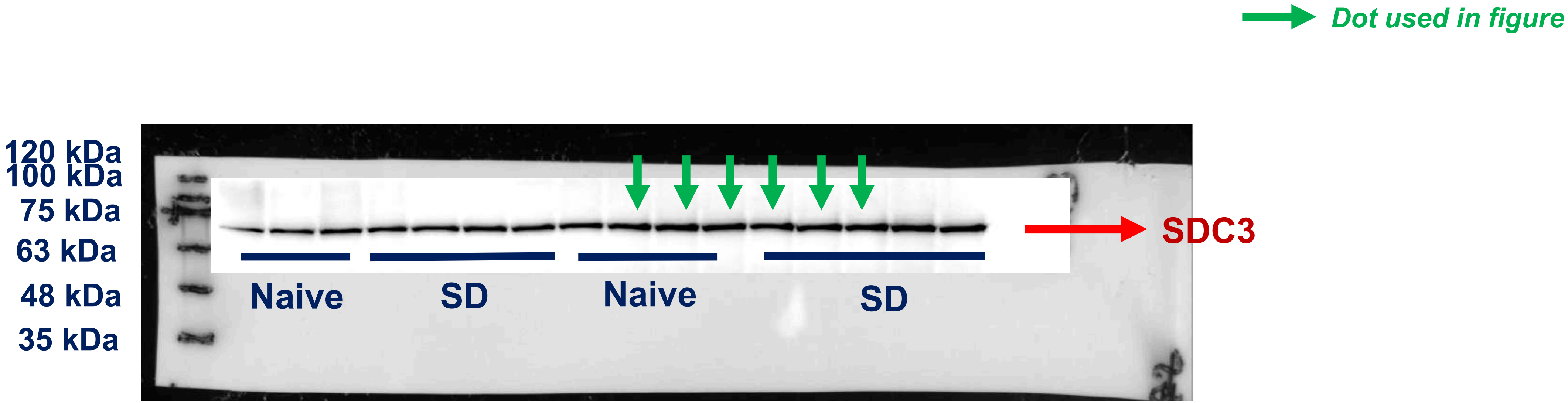

Actin

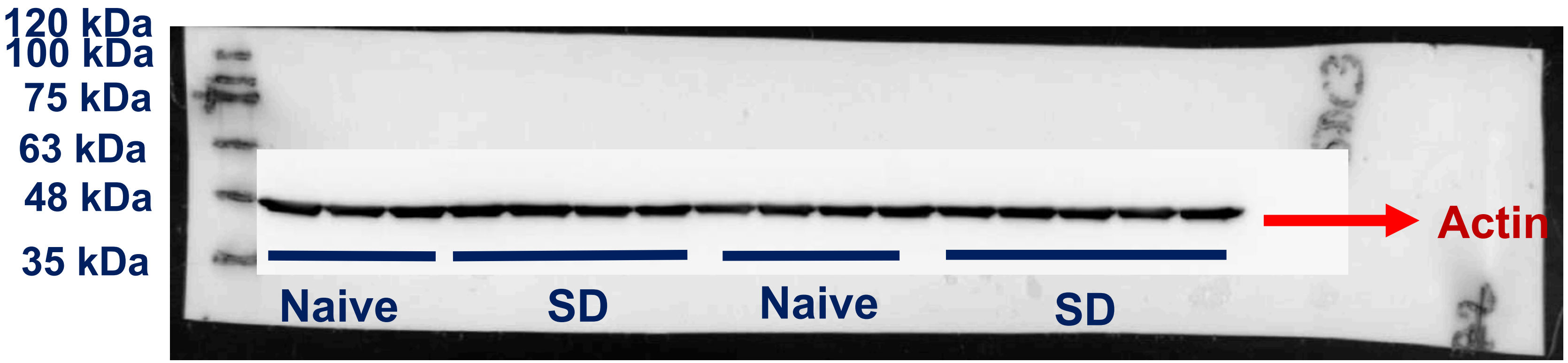

Figure 5 G,H

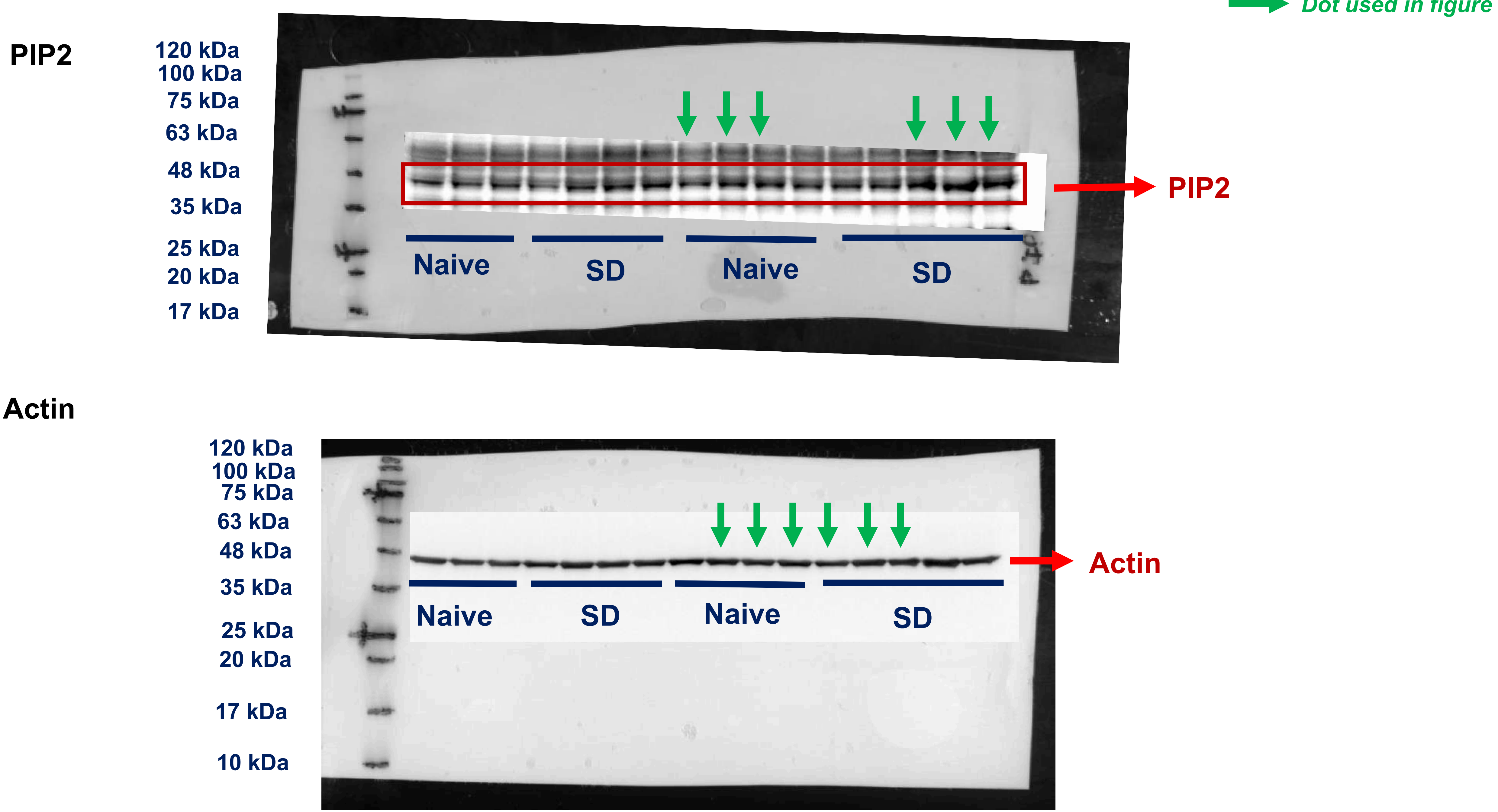

Dot used in figure
PIP2
120 kDa
100 kDa
75 kDa
63 kDa
48 kDa
35 kDa
25 kDa
20 kDa
17 kDa
Naive
SD
Naive
SD
PIP2
Actin
120 kDa
100 kDa
75 kDa
63 kDa
48 kDa
35 kDa
25 kDa
20 kDa
17 kDa
10 kDa
Naive
SD
Naive
SD
Actin

FIGURE S1

Supp Figure 1 E

Dot used in figure

Experiment #1
Total tau

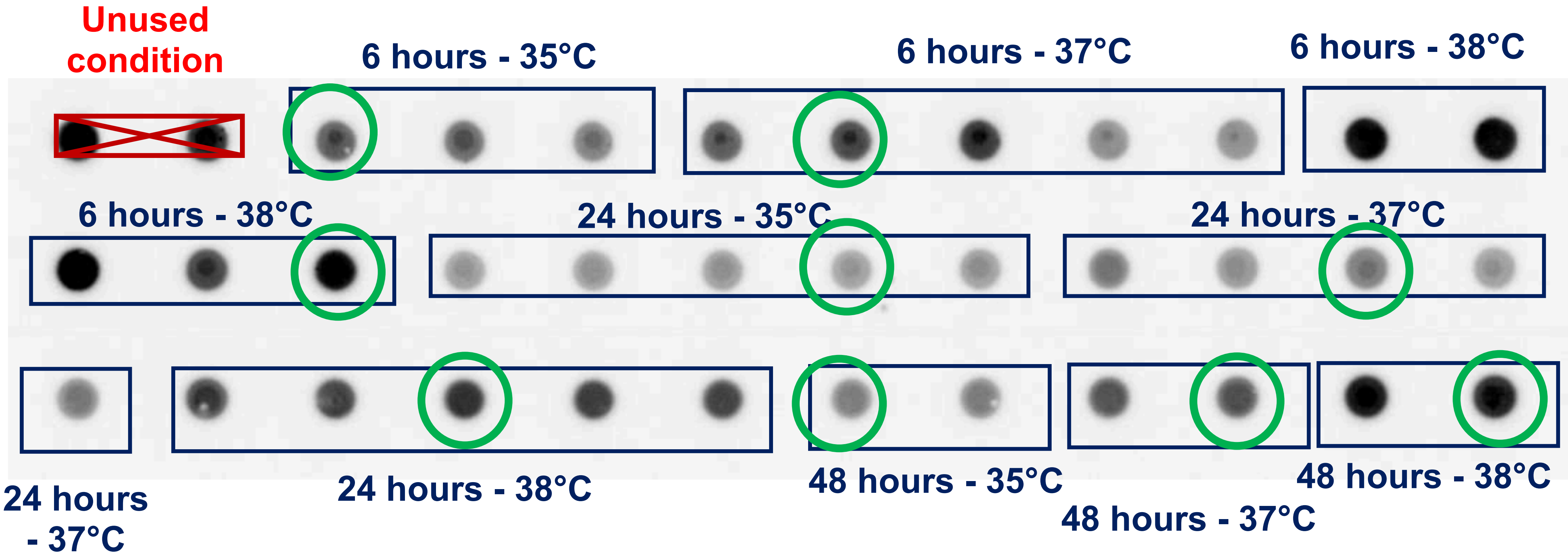

Experiment #1
Total tau

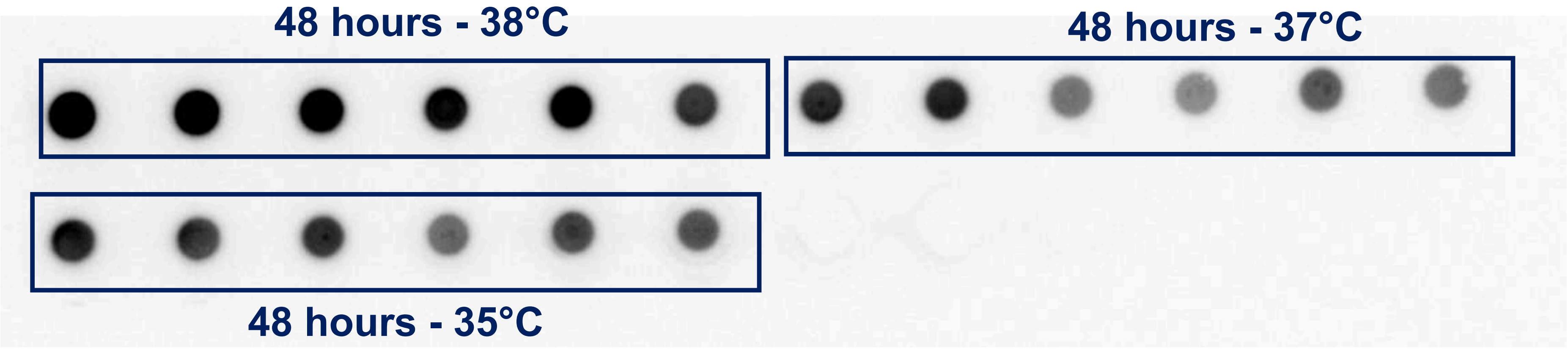

Supp Figure 1F

Dot used in figure

Total tau - SH-Tau3R

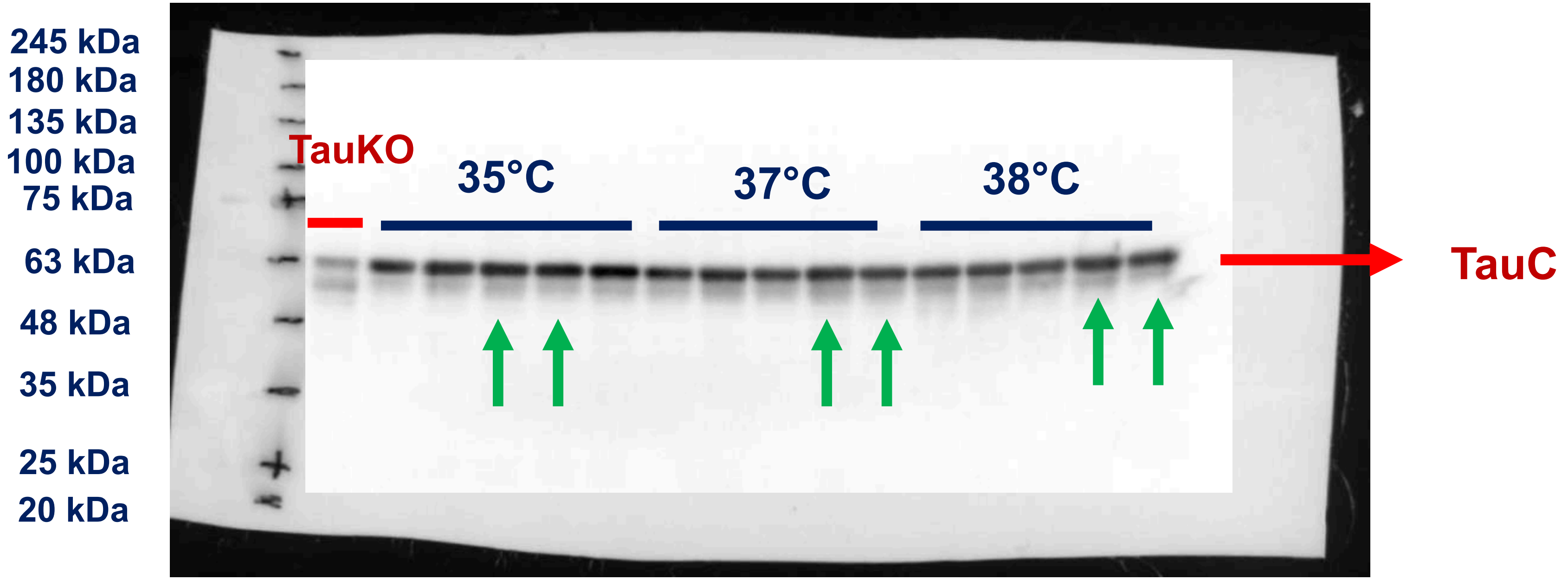

Corresponding Actin

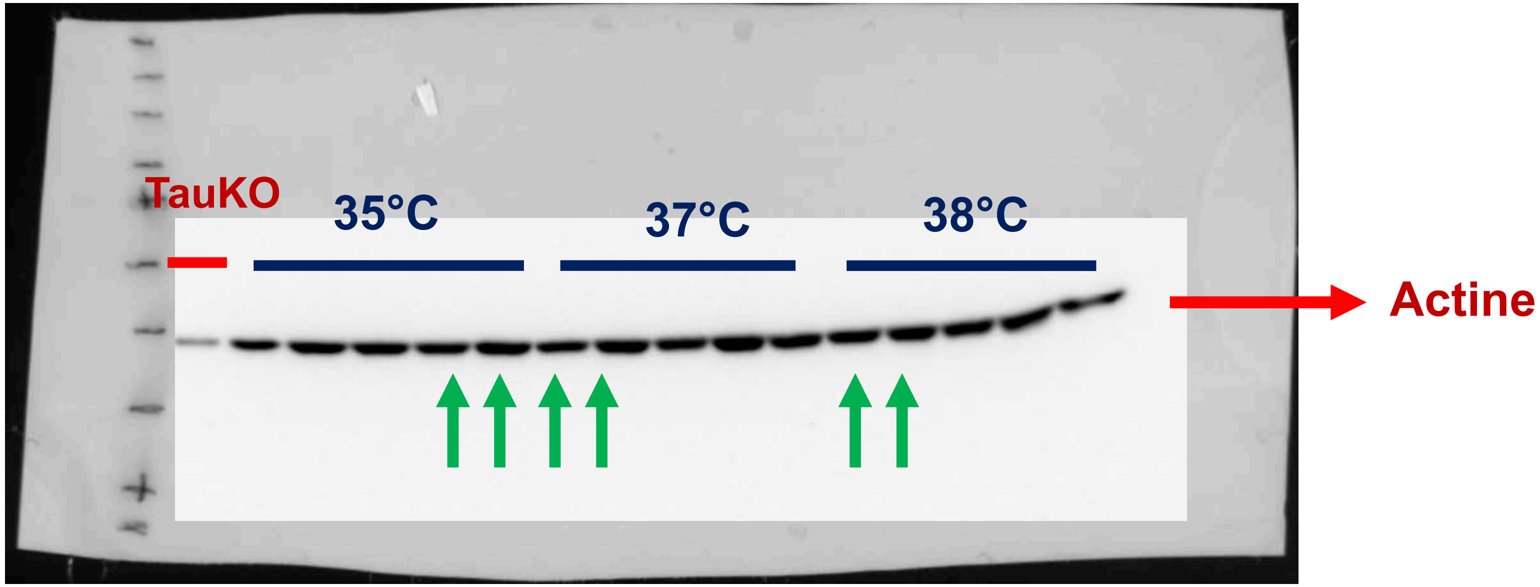

Supp Figure 1F

AT270 - SH-Tau3R

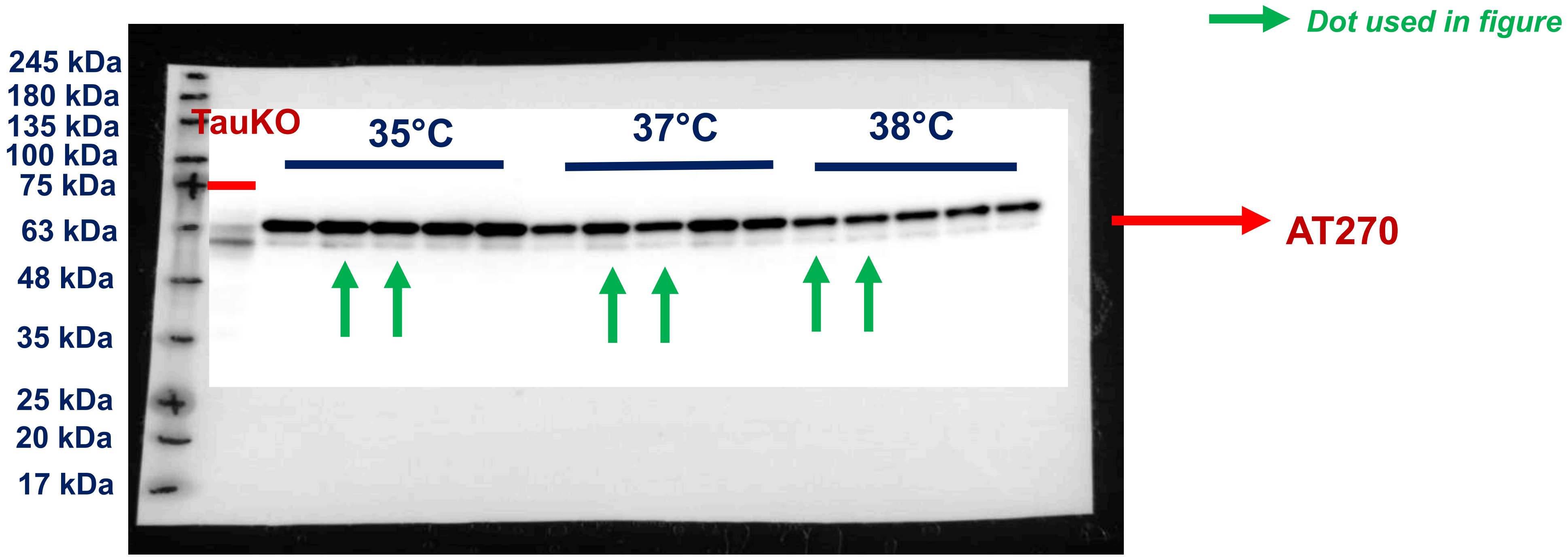

Corresponding Total tau

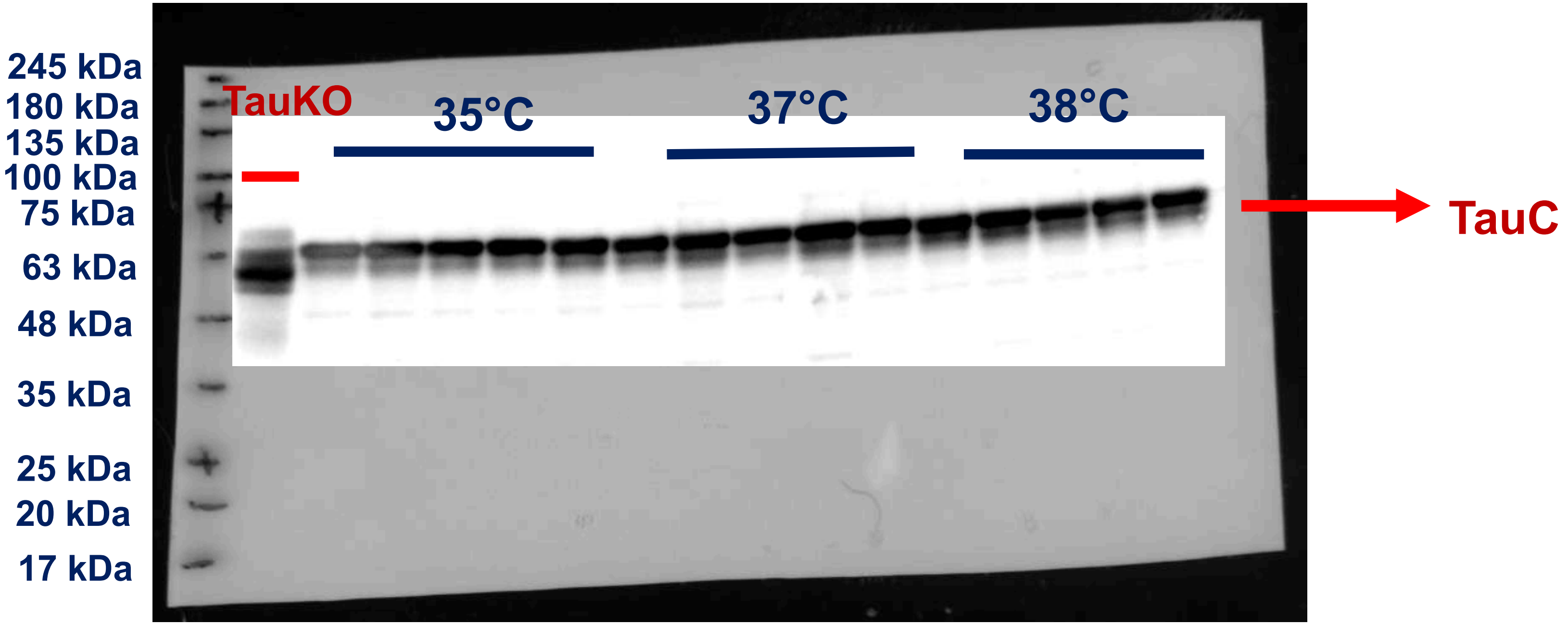

Supp Figure 1F

Dot used in figure

S199 - SH-Tau3R

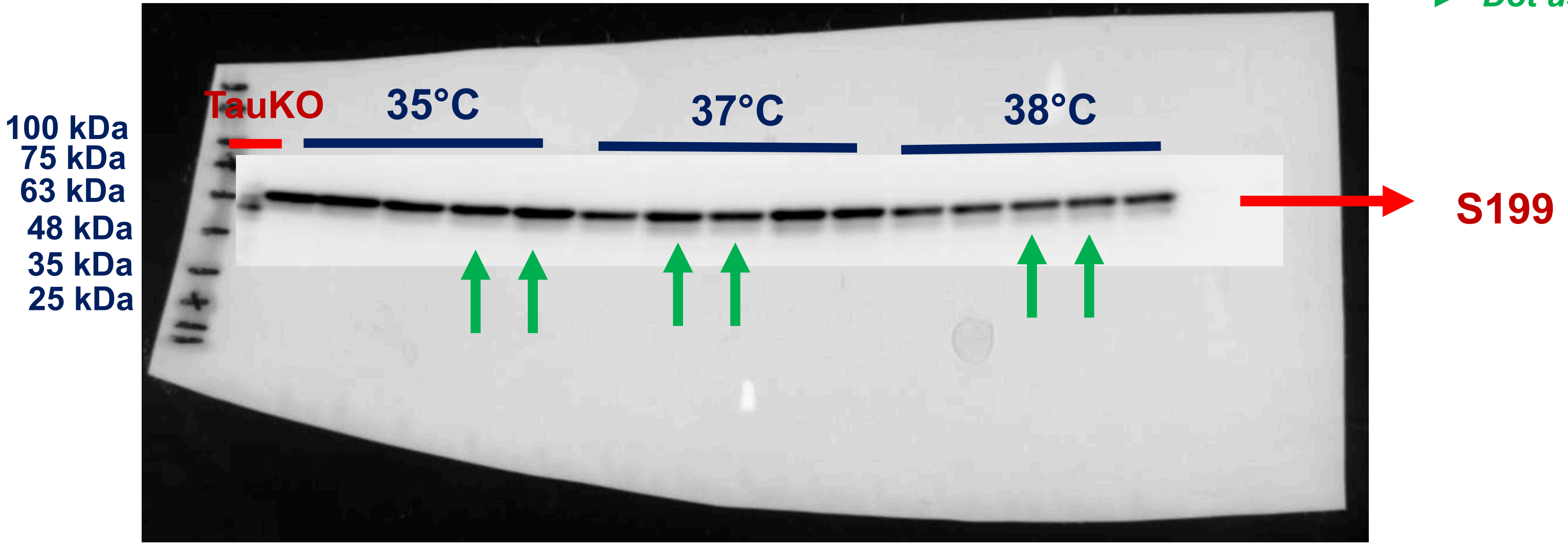

Corresponding Total tau

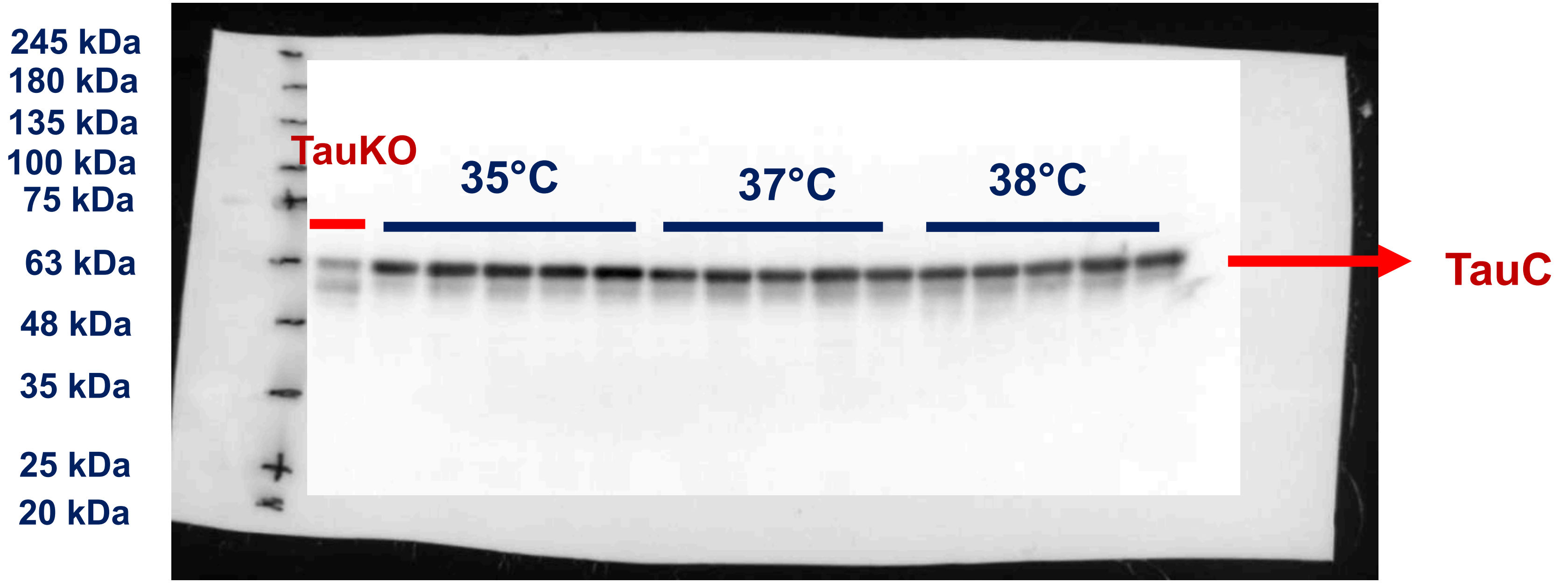

Supp Figure 1F

CP13 - SH-Tau3R

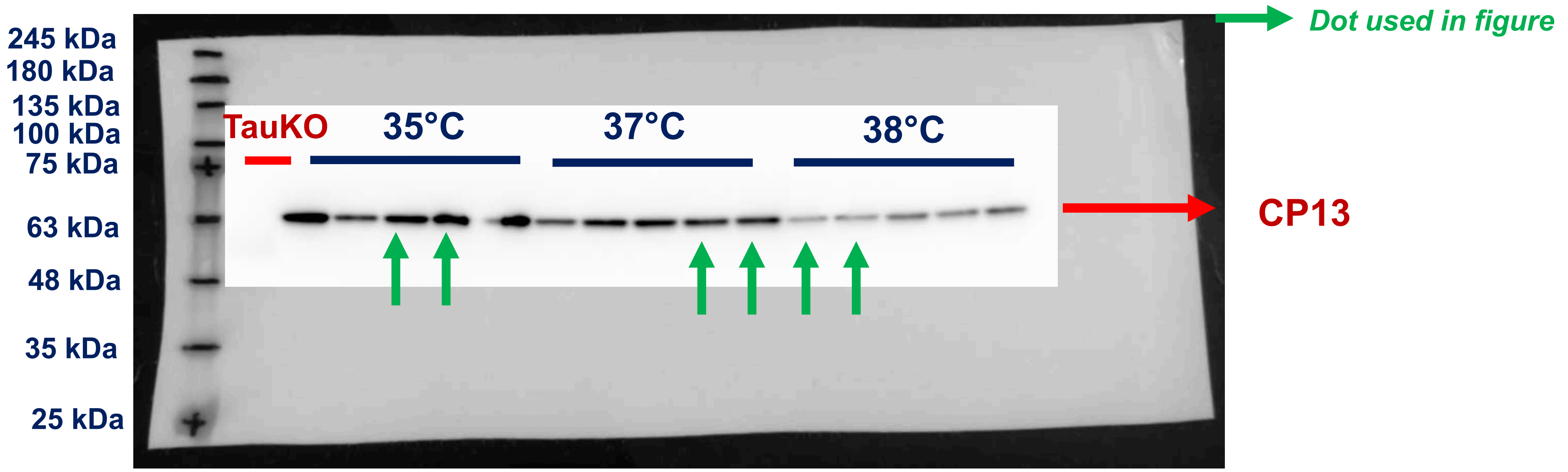

Corresponding Total tau

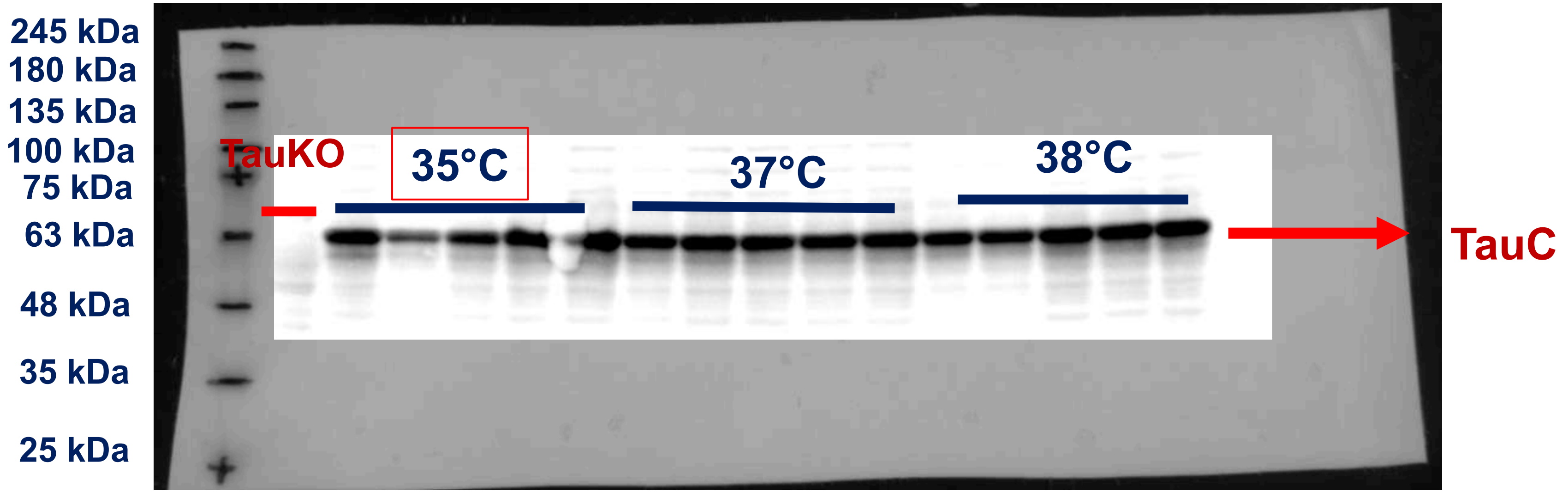

Supp Figure 1F

T205 - SH-Tau3R

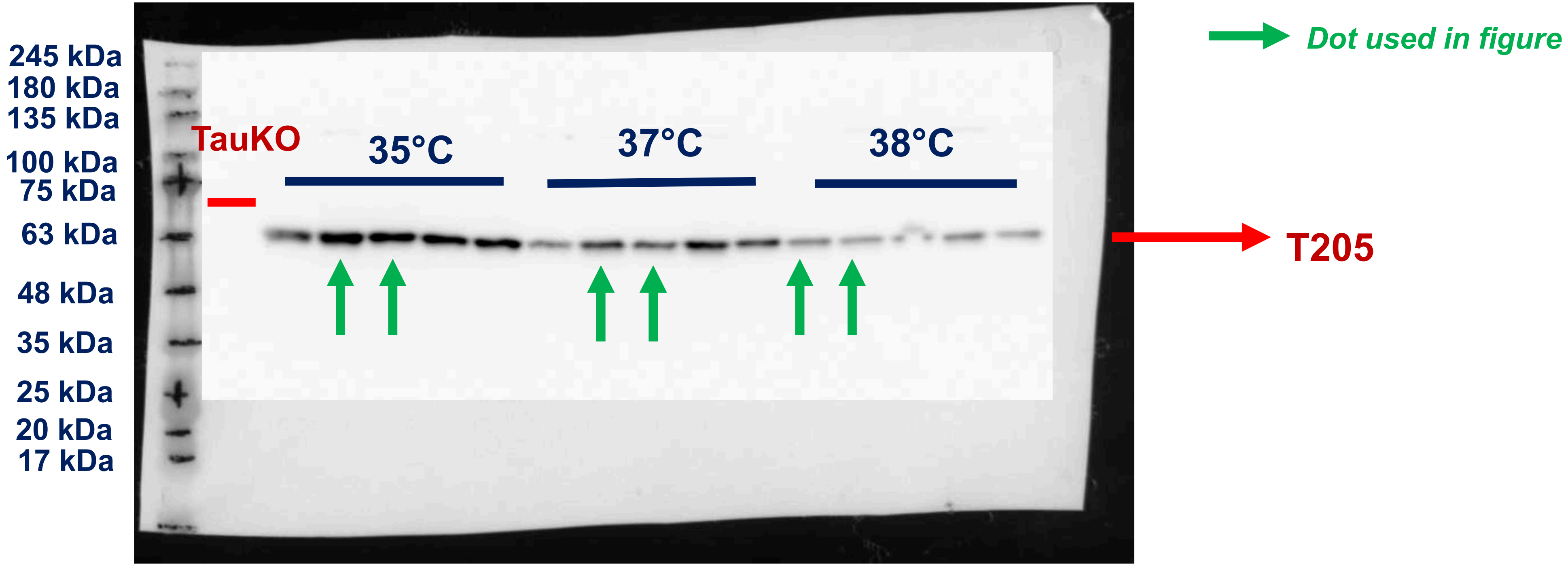

Corresponding Total tau

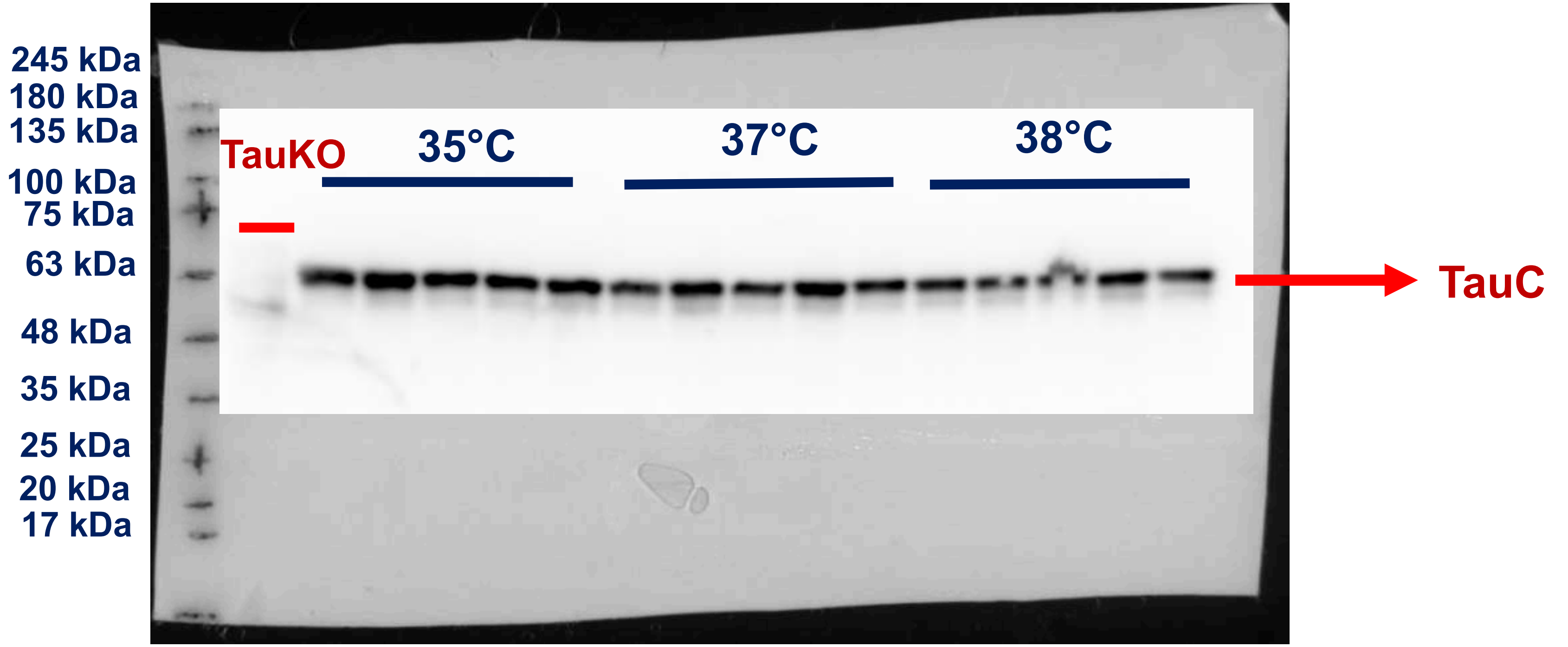

Supp Figure 1F

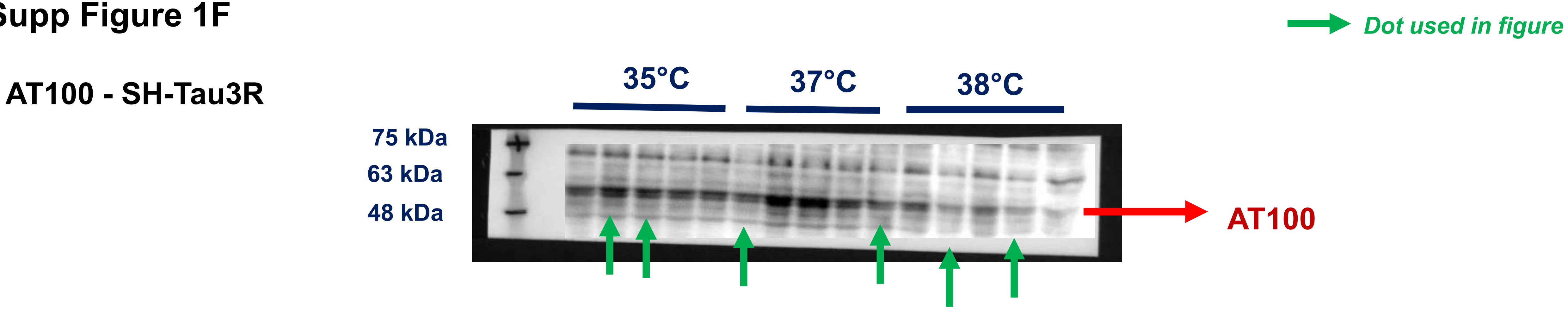

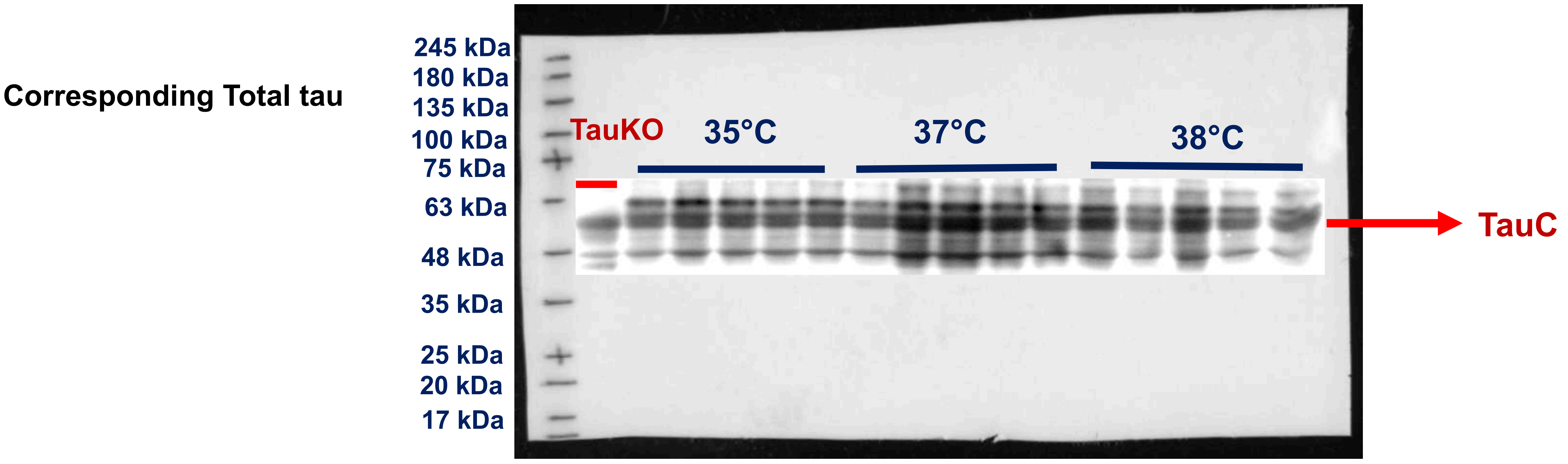

Supp Figure 1F

Dot used in figure

MC6 - SH-Tau3R

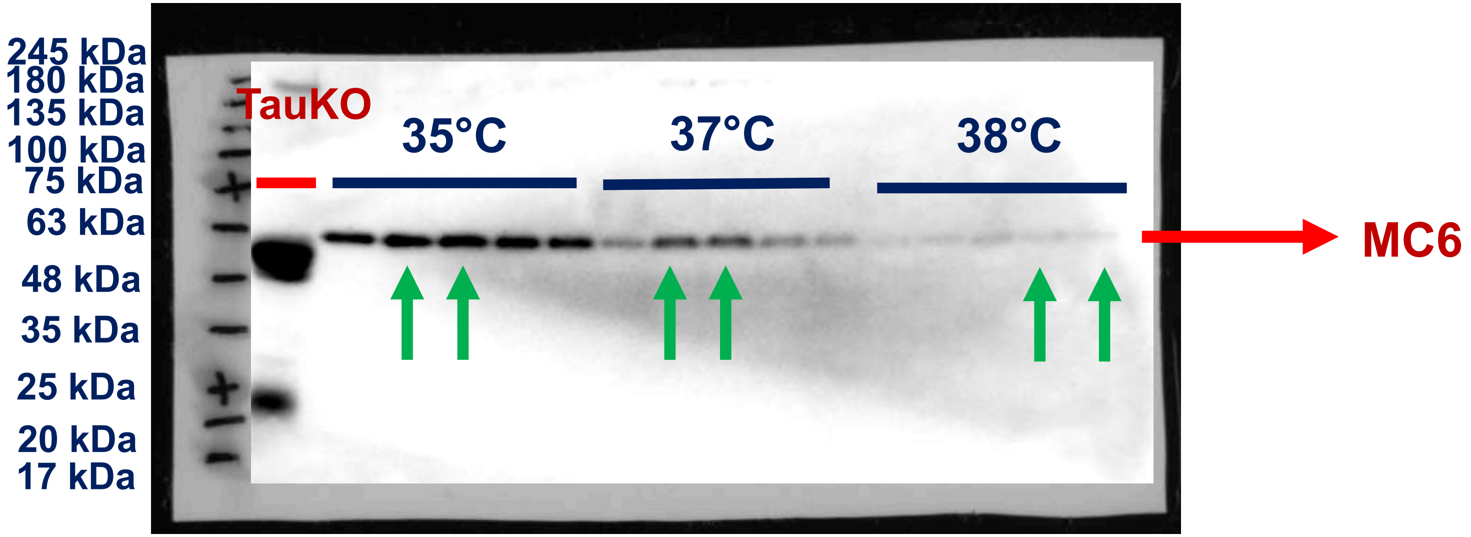

Corresponding Total tau

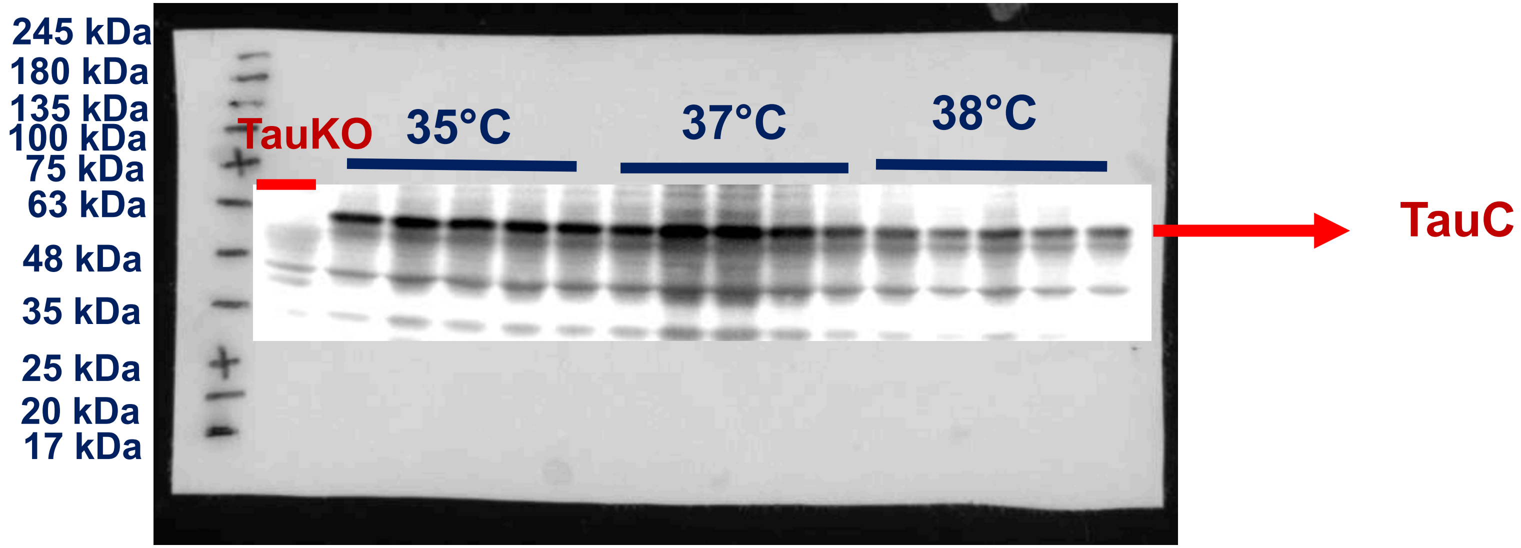

Supp Figure 1F

PHF1 - SH-Tau3R

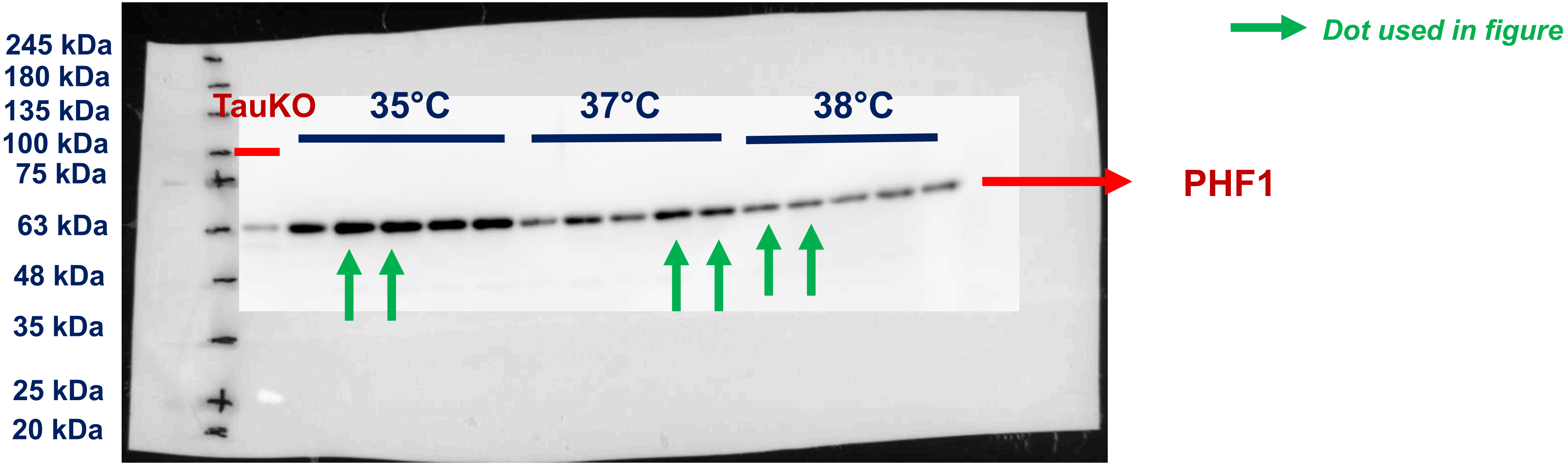

Corresponding Total tau

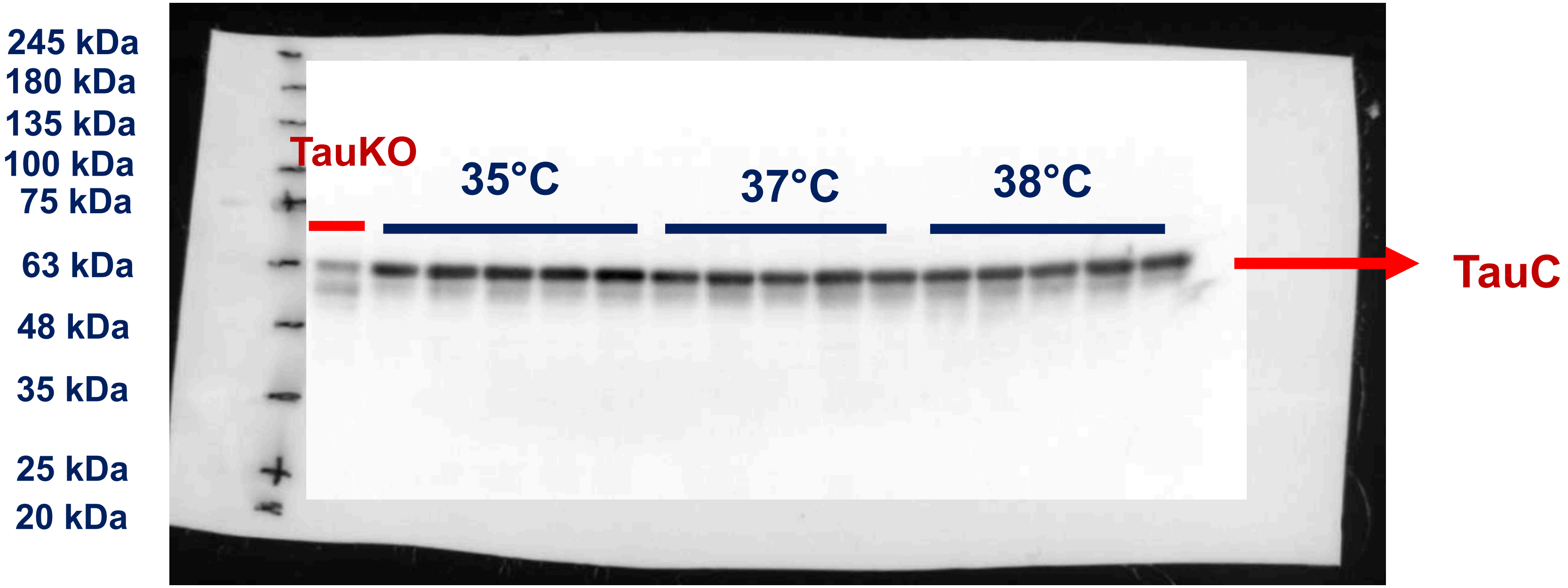

Supp Figure 1H

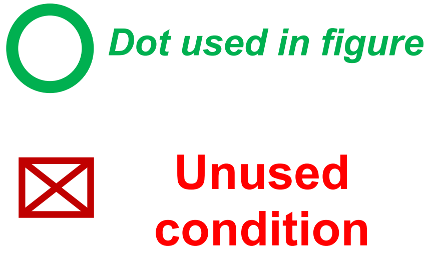

MAP2

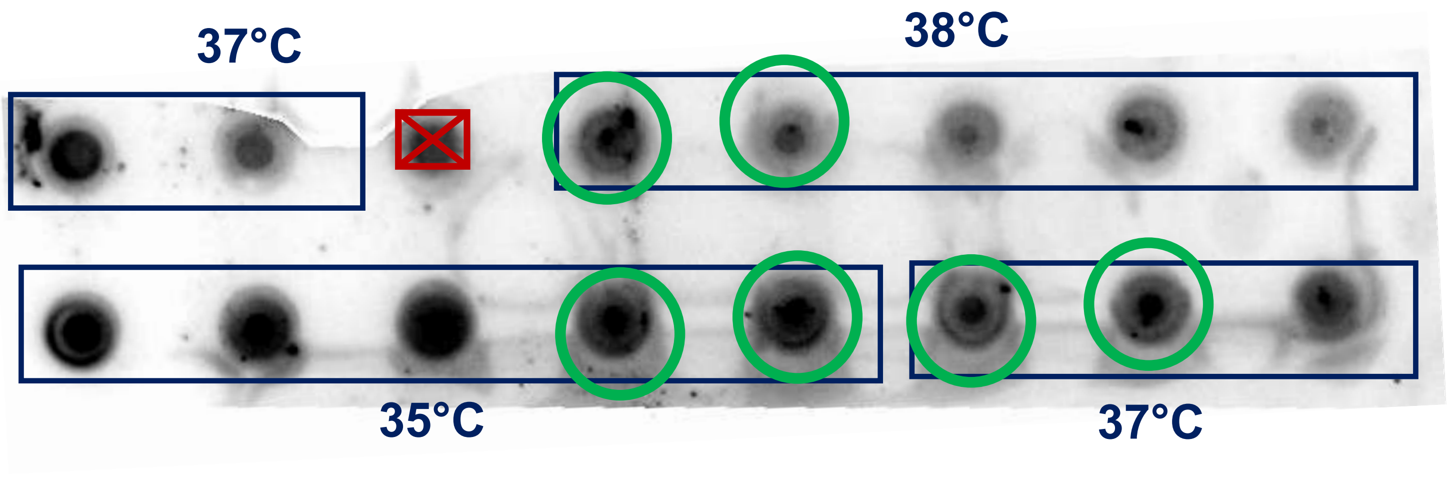

Alpha-synuclein

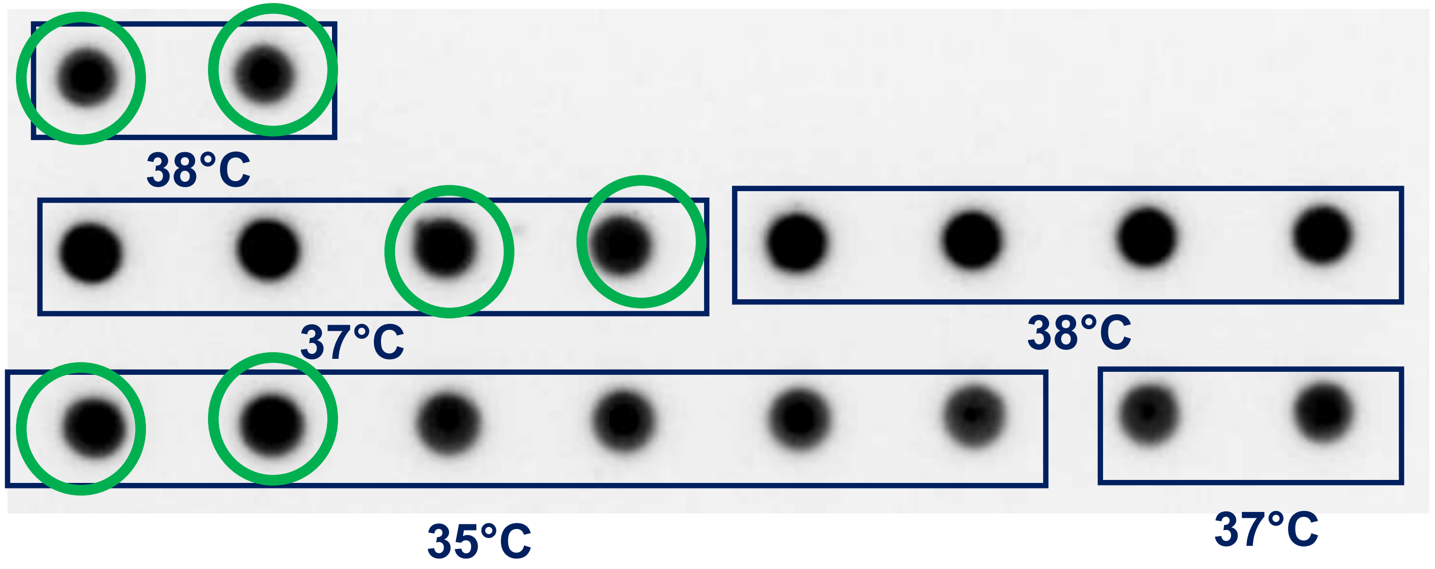

Supp Figure 1H

Dot used in figure

FGF2

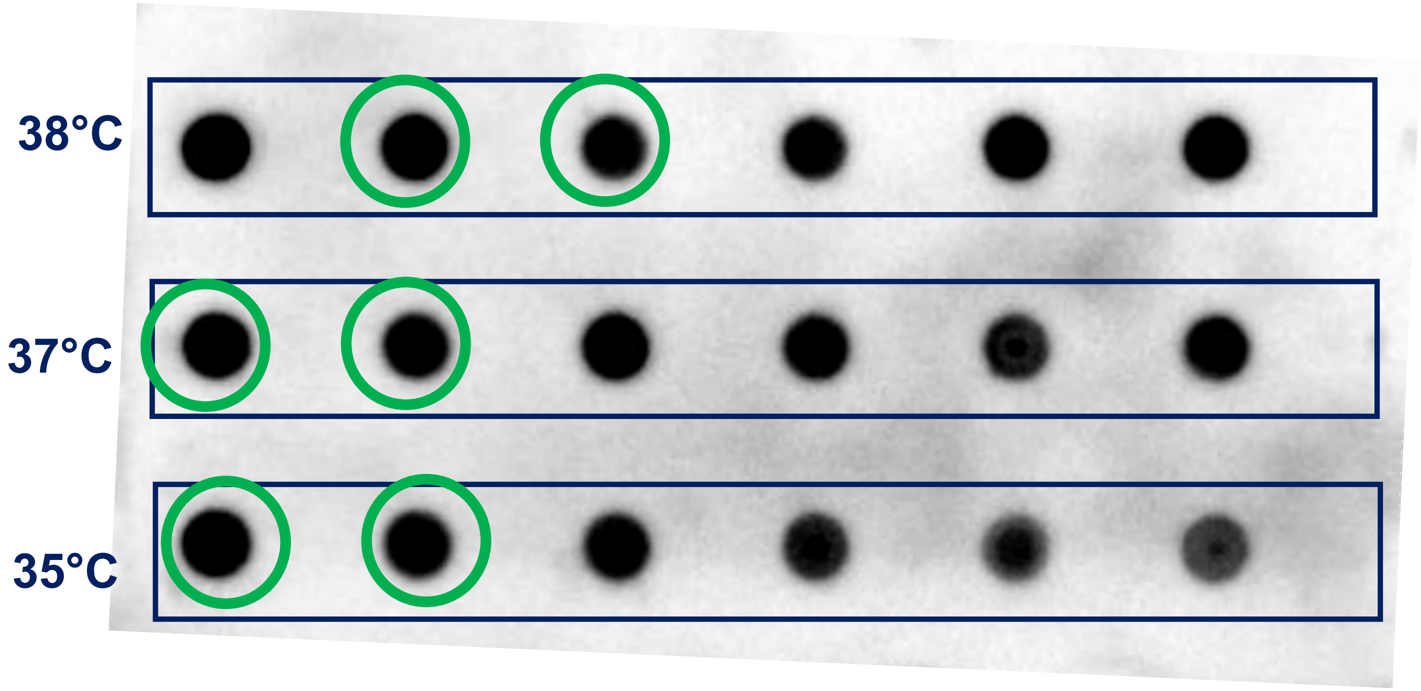

Caspase-1

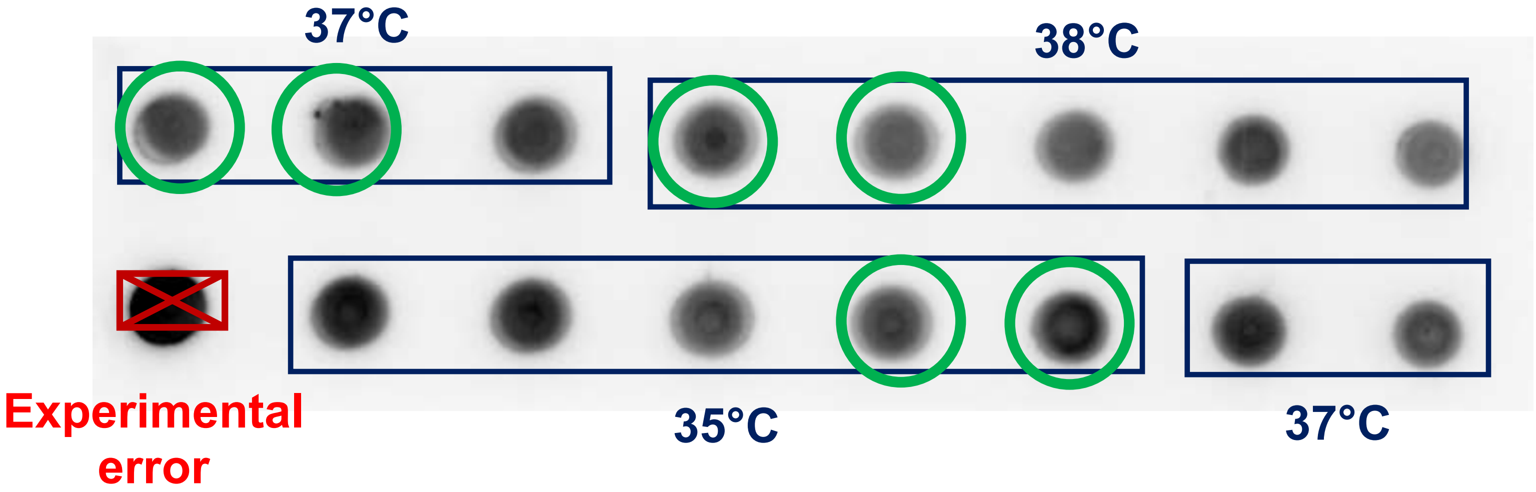

Supp Figure 1H

Dot used in figure

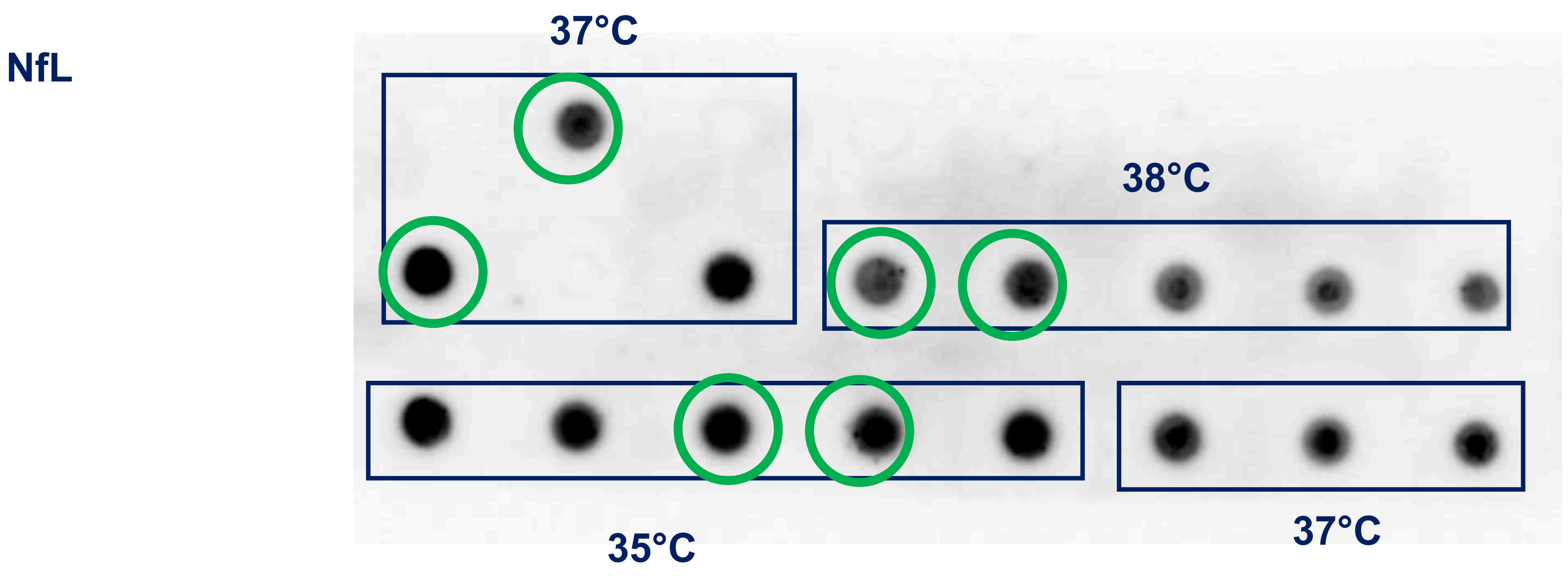

Supp Figure 1J

Dot used in figure

FGF2 - SH-Tau3R

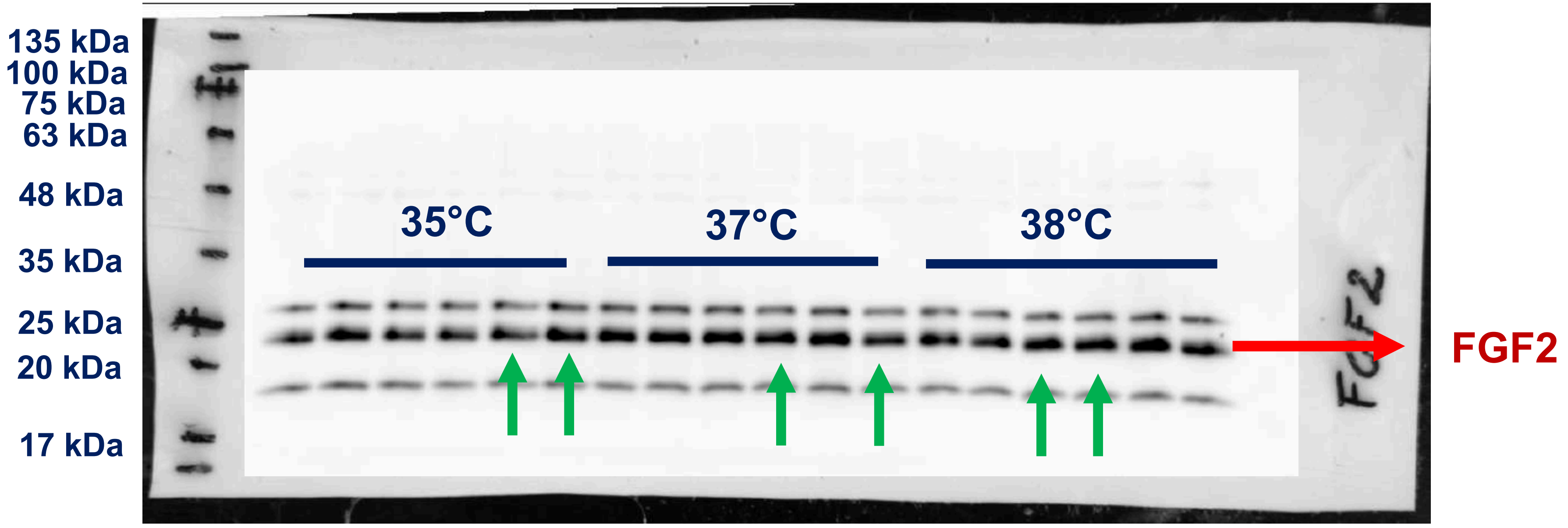

Corresponding Actin

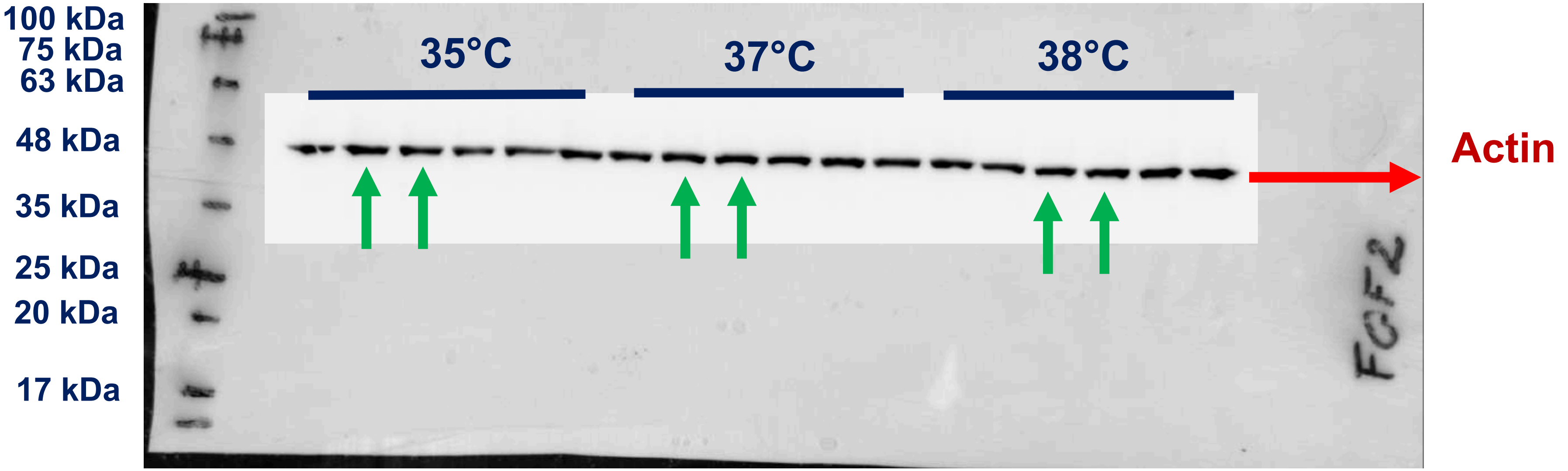

FIGURE S6

Supp Figure 6C

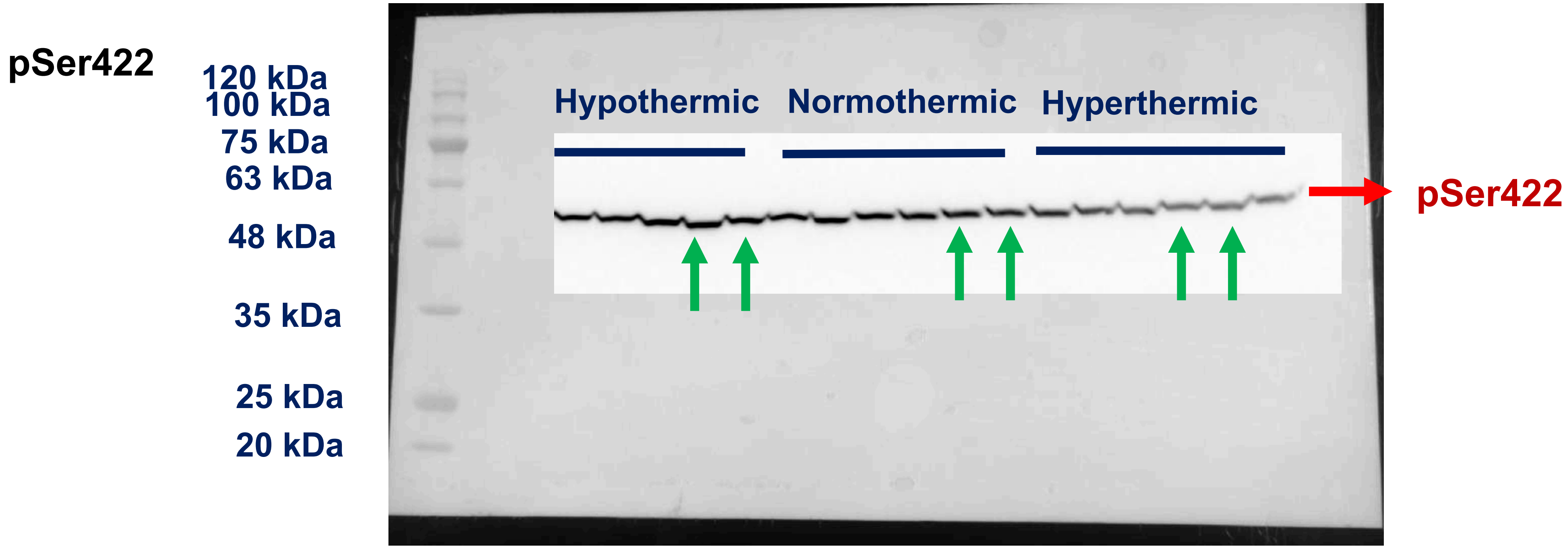

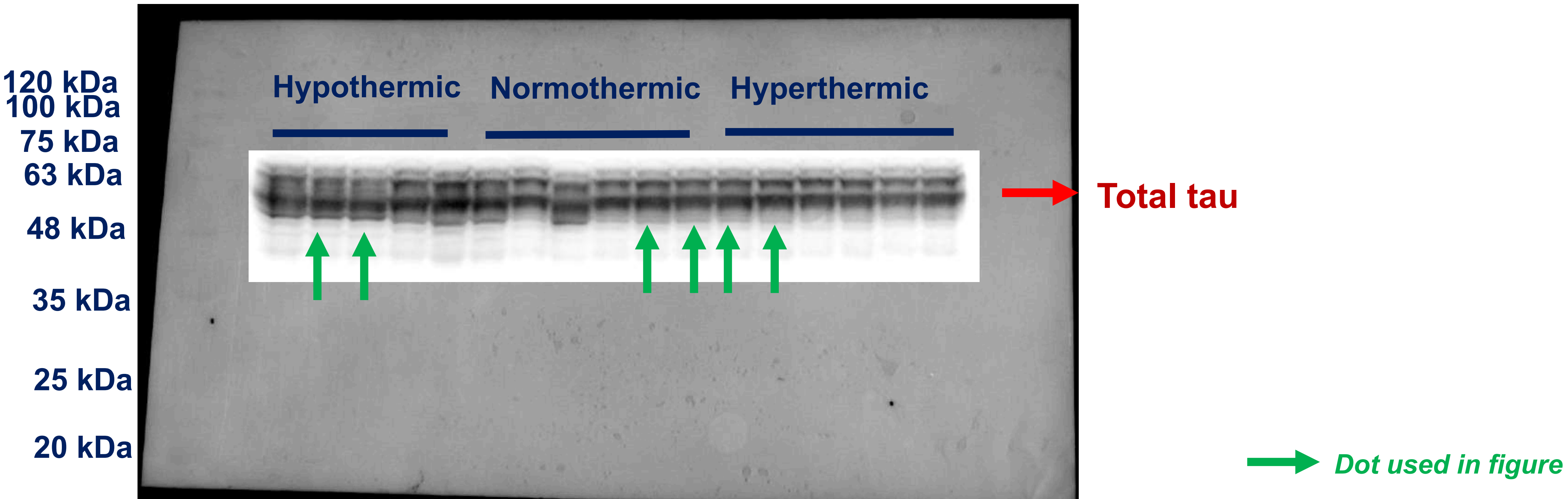

Supp Figure 6C

Dot used in figure

TauC3

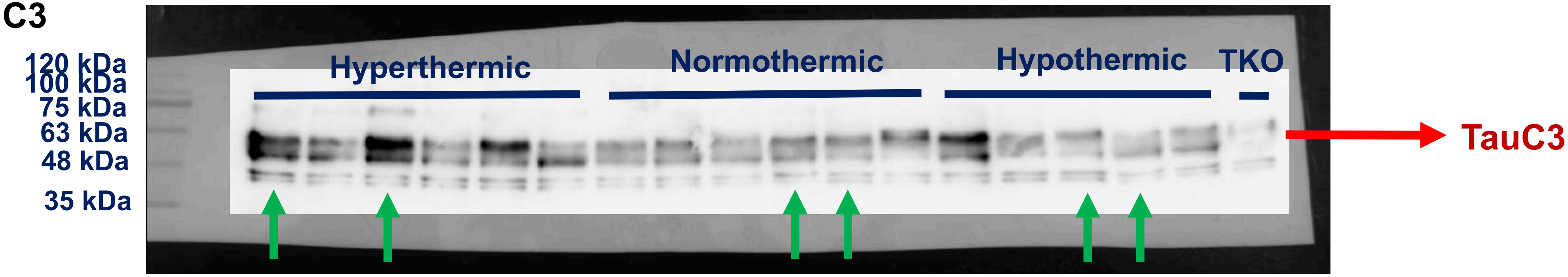

Actin

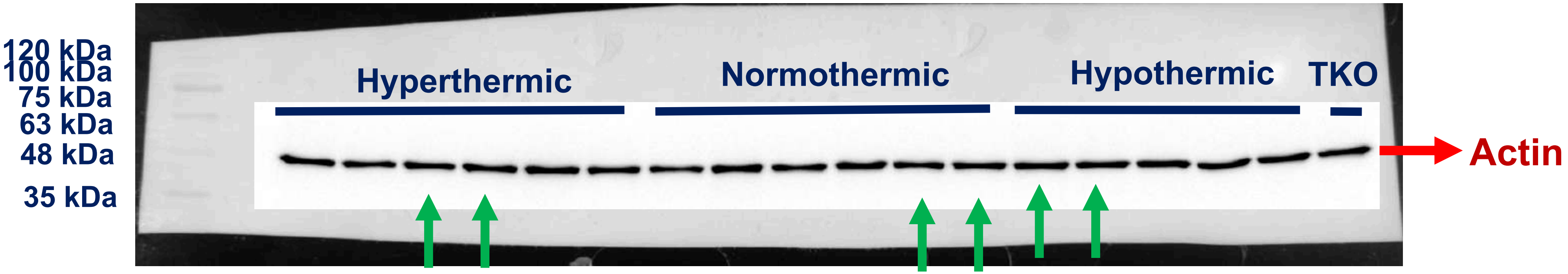

Supp Figure 6C

Tau46

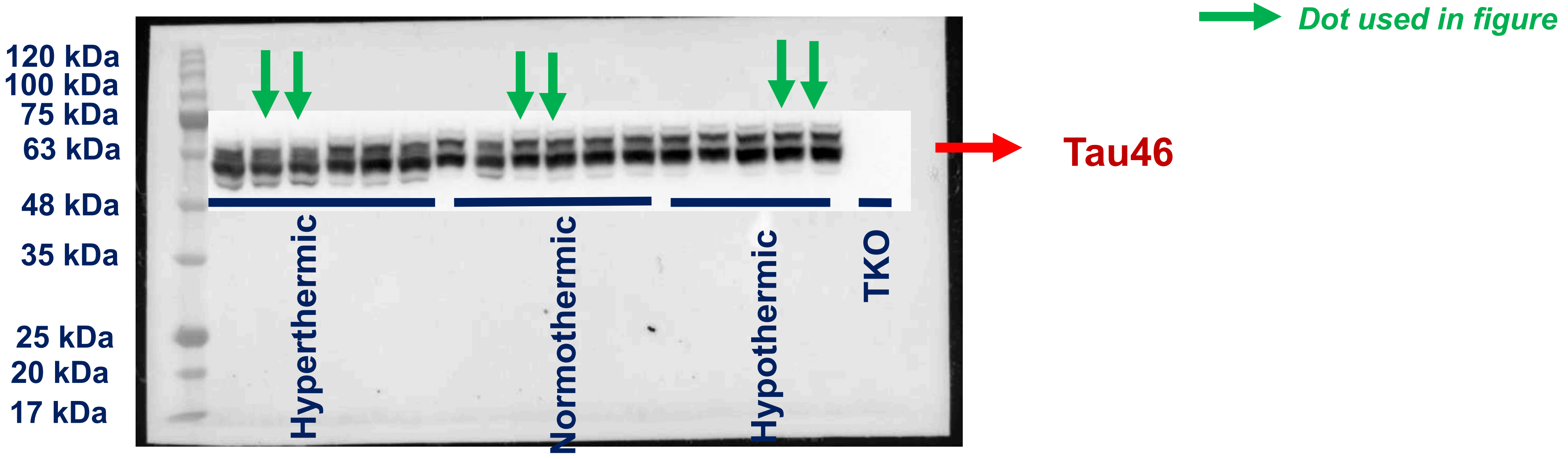

Actin

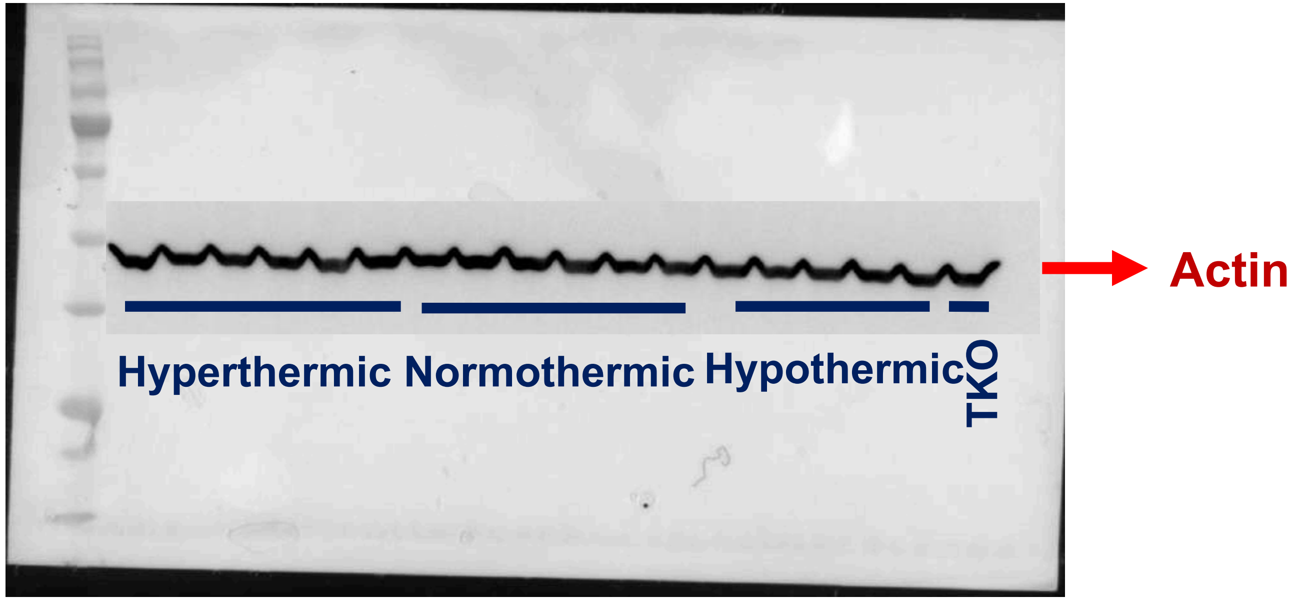

Supp Figure 6C

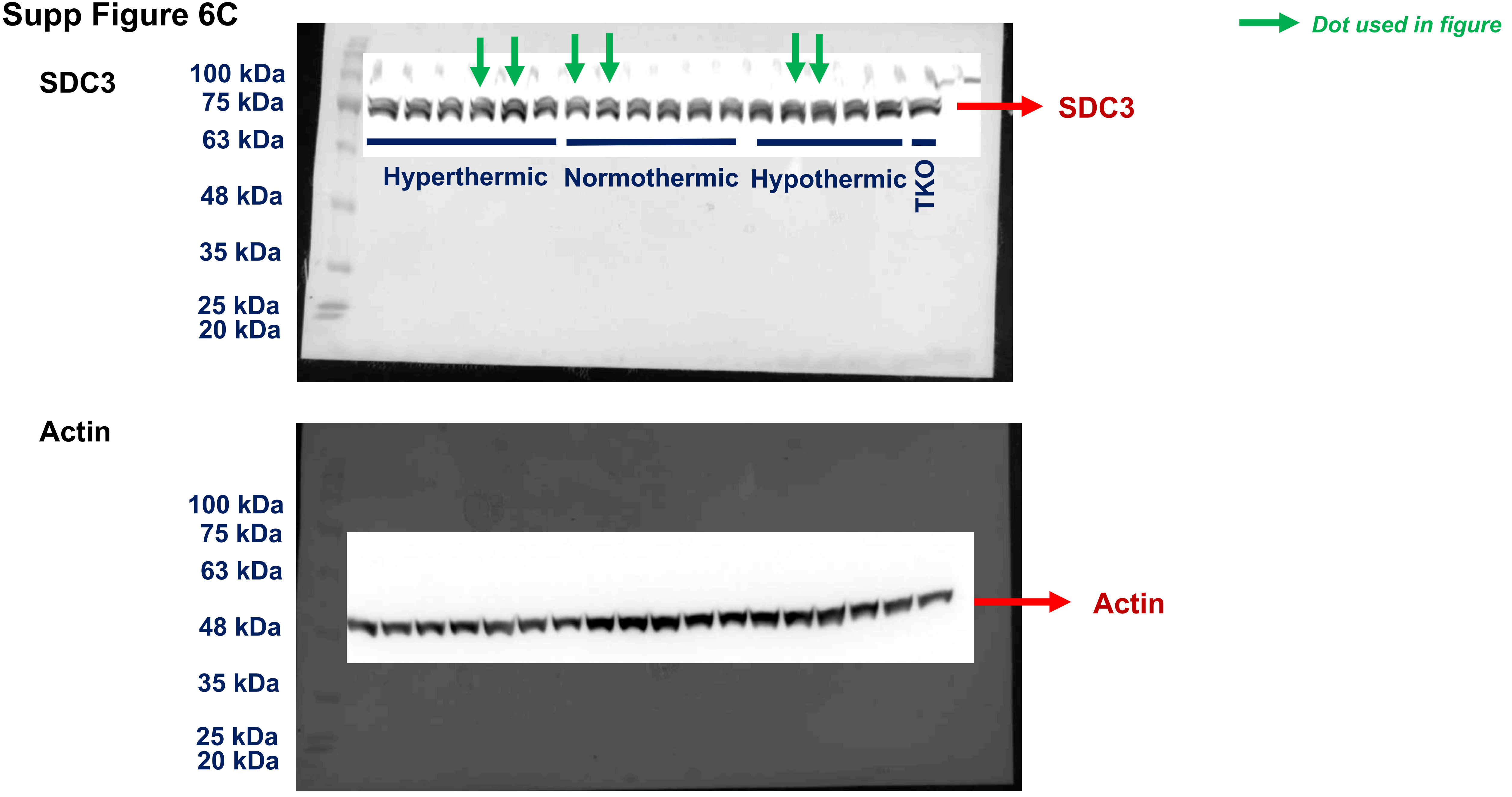

Supp Figure 6C

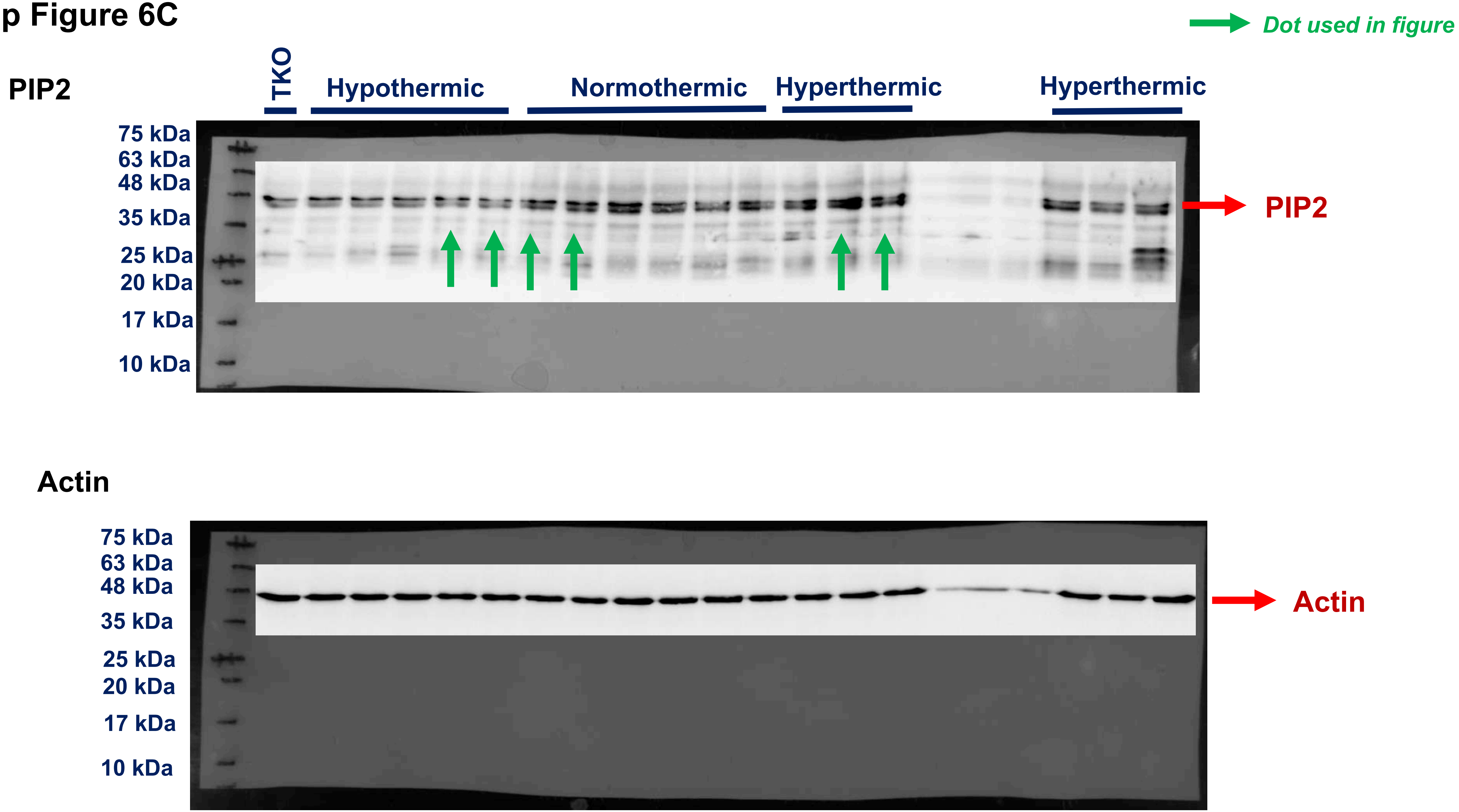

Supplement: Unedited blot and gel images [file jci-135-182931-s041.pdf]
